# Supplementary material for: Challenges and facilitators for health professionals providing primary healthcare for refugees and asylum seekers in high-income countries: a systematic review and thematic synthesis of qualitative research
Source: BMJ Open. 2017 Aug 4;7(8):e015981. doi: 10.1136/bmjopen-2017-015981 (PMC5629684; doi:10.1136/bmjopen-2017-015981)
Supplement: Supplementary material 1 [file bmjopen-2017-015981supp001.pdf]

Supplement 1: Database search strategy

MEDLINE	EMBASE	CINAHL	WEB OF SCIENCE	PSYCINFO
1. refugee/<br>2. asylum Seek$.mp.<br>3. refugee$.mp.	1. refugee/<br>2. asylum seeker/<br>3. asylum seek*.mp.<br>4. refugee*.mp.	1. MH “Refugees”<br>2. “refugee*”<br>3. ”asylum seek*”	1. refugee*<br>2. asylum seek*	1. exp refugees/<br>2. asylum seek*.mp.<br>3. refugee*.mp.
4. 1 or 2 or 3	5. 1 or 2 or 3 or 4	4. 1 or 2 or 3	3. 1 or 2	4. 1 or 2 or 3
5. exp Primary Healthcare/<br>6. exp health services/<br>7. exp health personnel/<br>8. nurs$.mp.<br>9. pharmacist$.mp.<br>10. health care.mp.<br>11. midwi$.mp.<br>12. general practi$.mp.<br>13. service provi$.mp.<br>14. care prov$.mp.<br>15. healthcare.mp.	6. exp primary health care/<br>7. exp health service/<br>8. exp health care personnel/<br>9. healthcare.mp.<br>10. health care.mp.<br>11. nurs*.mp.<br>12. pharmacist*.mp.<br>13. midwi*.mp.<br>14. general practi*.mp.<br>15. service prov*.mp.<br>16. care prov*.mp.	5. MH “Facilities Manpower and Services+”<br>6. MH “Health Personnel+”<br>7. “healthcare”<br>8. “health care”<br>9. “service prov*”<br>10. “care prov*”<br>11. “nurs*”<br>12. “pharmacist*”<br>13. “midwi*”<br>14. “general practi*”	4. healthcare<br>5. health care<br>6. service prov*<br>7. care prov*<br>8. nurs*<br>9. pharmacist*<br>10. midwi*<br>11. general practi*	5. exp Health Care Services/<br>6. exp primary health care/<br>7. exp Health Personnel/<br>8. health care.mp.<br>9. healthcare.mp.<br>10. care prov*.mp.<br>11. service prov*.mp.<br>12. nurs*.mp.<br>13. pharmacist*.mp.<br>14. midwi*.mp.<br>15. general practi*.mp.
16. 5 or 6 or 7 or 8 or 9 or 10 or 11 or 12 or 13 or 14 or 15	17. 6 or 7 or 8 or 9 or 10 or 11 or 12 or 13 or 14 or 15 or 16	15. 5 or 6 or 7 or 8 or 9 or 10 or 11 or 12 or 13 or 14	12. 4 or 5 or 6 or 7 or 8 or 9 or 10 or 11	16. 5 or 6 or 7 or 8 or 9 or 10 or 11 or 12 or 13 or 14 or 15
17. qualitative.mp.<br>18. qualitative research/<br>19. mixed method$.mp.<br>20. experienc$.mp.<br>21. perception$.mp.<br>22. attitude$.mp.<br>23. Perspective$.mp.<br>24. challenge$.mp.<br>25. barrier$.mp.<br>26. facilitator$.mp.	18. qualitative research/<br>19. qualitative.mp.<br>20. mixed method*.mp.<br>21. experienc*.mp.<br>22. perception*.mp.<br>23. attitude*.mp.<br>24. perspective*.mp.<br>25. challeng*.mp.<br>26. facilitator*.mp.<br>27. barrier*.mp.	16. MH “Qualitative Studies+”<br>17. “qualitative*”<br>18. “mixed method*”<br>19. “experienc*”<br>20. “perception*”<br>21. “attitude*”<br>22. “perspective*”<br>23. “challeng*”<br>24. “facilitator*”<br>25. “barrier*”	13. qualitative<br>14. mixed method*<br>15. experienc*<br>16. perception*<br>17. attitude*<br>18. perspective*<br>19. challeng*<br>20. facilitator*<br>21. barrier*	17. exp Qualitative Research/<br>18. qualitative.mp.<br>19. mixed method*.mp.<br>20. experienc*.mp.<br>21. perception*.mp.<br>22. attitude*.mp.<br>23. perspective*.mp.<br>24. challeng*.mp.<br>25. facilitator*.mp.<br>26. barrier*.mp.
27. 17 or 18 or 19 or 20 or 21 or 22 or 23 or 24 or 25 or 26	28. 18 or 19 or 20 or 21 or 22 or 23 or 24 or 25 or 26 or 27	26. 16 or 17 or 18 or 19 or 20 or 21 or 22 or 23 or 24 or 25	22. 13 or 14 or 15 or 16 or 17 or 18 or 19 or 20 or 21	27. 17 or 18 or 19 or 20 or 21 or 22 or 23 or 24 or 25 or 26
28. 4 and 16 and 27	29. 5 and 17 and 28	27. 4 and 15 and 26	23. 3 and 12 and 22	28. 4 and 16 and 27
**1377**	**1909**	**954**	**875**	**855**

**Total from database searches**	**5,970**
