# Supplementary material for: Challenges and facilitators for health professionals providing primary healthcare for refugees and asylum seekers in high-income countries: a systematic review and thematic synthesis of qualitative research
Source: BMJ Open. 2017 Aug 4;7(8):e015981. doi: 10.1136/bmjopen-2017-015981 (PMC5629684; doi:10.1136/bmjopen-2017-015981)
Supplement: Supplementary material 2 [file bmjopen-2017-015981supp002.pdf]

Supplement 2: Selection criteria

Study type:

Include	Exclude
Primary qualitative research studies	Theses/Dissertations
Interviews/focus groups	Opinion articles
Peer reviewed	Case studies
	Surveys (quantitative)
	Organisation reports
	Reviews

Primary health care professionals:

Include	Exclude
Nurses	Obstetricians
General practitioners	Psychologists
Midwives	Psychotherapists
Health visitors (nurse/midwives)	Physiotherapists
Pharmacists	Counsellors
	Social workers
	Managers
	Interpreters
	Volunteers
	Unqualified health professional (e.g student nurse)
	Unspecified staff within service providers

Health care service users:

Include	Exclude
Refugees	Migrants
Asylum seekers	Immigrants
Forced/Involuntary migrants	Undocumented migrant
Refugee claimant	Illegal immigrant

Setting of practice of health professionals:

Include	Exclude
Community	Asylum seeker detention centre
Community health centres	Hospitals- acute care
General practices	Specialist centres: referral from primary care
Community clinics	
Refugee/asylum centres	

High-income countries (World Bank classification 2015[1]):

Include:

Andorra	Guam	Saudi Arabia
Antigua and Barbuda	Hong Kong SAR, China	Seychelles
Argentina	Hungary	Singapore
Aruba	Iceland	Sint Maarten (Dutch part)
Australia	Ireland	Slovak Republic
Austria	Isle of Man	Slovenia
Bahamas, The	Israel	Spain
Bahrain	Italy	St. Kitts and Nevis
Barbados	Japan	St. Martin (French part)
Belgium	Korea, Rep.	Sweden
Bermuda	Kuwait	Switzerland
Brunei Darussalam	Latvia	Taiwan, China
Canada	Liechtenstein	Trinidad and Tobago
Cayman Islands	Lithuania	Turks and Caicos Islands
Channel Islands	Luxembourg	United Arab Emirates
Chile	Macao SAR, China	United Kingdom
Croatia	Malta	United States
Curaçao	Monaco	Uruguay
Cyprus	Netherlands	Venezuela, RB
Czech Republic	New Caledonia	Virgin Islands (U.S.)
Denmark	New Zealand	
Equatorial Guinea	Northern Mariana Islands	
Estonia	Norway	
Faeroe Islands	Oman	
Finland	Poland	
France	Portugal	
French Polynesia	Puerto Rico	
Germany	Qatar	
Greece	Russian Federation	
Greenland	San Marino	

Focus of study:

**Include**	**Exclude**
Experiences providing primary healthcare for refugees and asylum seekers	Experiences treating a specific condition common in refugees and asylum seekers, but no focus on healthcare interactions.<br>Experiences of a particular service or organisation for refugees and asylum seekers<br>HCP's perspectives on refugees and asylum seekers' experiences

[1] The World Bank, World Bank list of economies (July 2015) [Data file]. Retrieved from http://data.worldbank.org/about/country-and-lending-groups
