# Supplementary material for: Challenges and facilitators for health professionals providing primary healthcare for refugees and asylum seekers in high-income countries: a systematic review and thematic synthesis of qualitative research
Source: BMJ Open. 2017 Aug 4;7(8):e015981. doi: 10.1136/bmjopen-2017-015981 (PMC5629684; doi:10.1136/bmjopen-2017-015981)
Supplement: Supplementary material 3 [file bmjopen-2017-015981supp003.pdf]

Supplement 3: Data extraction of studies included in the thematic synthesis

Study Reference	Country of study	Study type	Total Participants in Study	Health professional practice setting	Service users	Data collection method	Analysis method	Study aims/purpose (Author's words)	Reported results (Themes/main headings)	Recommendations/applications (Author's words)
Begg H, Gill PS. Views of general practitioners towards refugees and asylum seekers: an interview study. Diversity Health Soc Care 2005 12;2(4):299-305 7p.	United Kingdom	Qualitative	17 general practitioners	General practice	Refugees and asylum seekers	Semi-structured interviews	Thematic framework (Ritchie and Spencer, 1993)	To identify some of the concerns of 17 general Practitioners working in an urban environment.	1.Political logistics and the asylum process<br>2 Community issues<br>3 Impact upon primary care<br>4 Resources and resource management<br>5 Training needs within primary care	> Guidelines and protocols for practice ...GPs would welcome those that might help them to deliver healthcare to refugees and asylum seekers.<br>>Primary care trusts need to liaise with local authorities and the Home Office to identify areas to which large numbers of asylum seekers are dispersed.
Bennett S, Scammell J. Midwives caring for asylum-seeking women: research findings. Pract Midwife 2014 Jan;17(1):9-12.	United Kingdom	Qualitative	10 midwives	Setting unclear, but includes community, rotational, specialist and delivery suite midwives.	Asylum seeking women	Semi-structured interviews	Thematic analysis (Bryman 2008)	The aim of this research was to gain an in depth analysis of the experiences of midwives and their understanding of the specific needs of asylum-seeking women. The findings would be used to inform education, practice and policy to enable more effective delivery of woman-centred care for this group locally.	1. Time<br>2. Communication	>Midwives deserve support in practice and enhanced education, and policy around asylum-seeking women would facilitate more effective, evidence-based care.<br>>It is essential that midwives (and other members of the multi-disciplinary team) have access to and training in the use of interpreting services.<br>>The additional time required to provide care to women seeking asylum should be factored into midwives' workloads.<br>>Education programmes to prepare/enhance knowledge and skills in caring for asylum seekers<br>>Web based resource with information about asylum seekers.
Burchill J. Safeguarding vulnerable families: work with refugees and asylum seekers. Community Practitioner 2011 Feb;84(2):23-26.	United Kingdom	Qualitative	14 health visitors	London borough	Refugees and asylum seekers	In-depth interviews	Thematic framework (Ritchie & Spencer, 1994)	Not clearly stated	1. Complexity of safeguarding-related needs<br>2. Sole support agent<br>3. Cultural challenges<br>4. Cycle of abuse<br>5. Disappearing from the system	> Increase awareness for effective commissioning of appropriate services for this group.<br>> Joint working may prevent the difficulties that health visitors face when working with vulnerable populations such as asylum seekers and refugees.<br>> Health visitors working with vulnerable populations need to explore opportunities to highlight concerns with their managers and commissioners.
Burchill J, Pevalin D. Barriers to effective practice for health visitors working with asylum seekers and refugees. Community Practitioner 2012 Jul;85(7):20-23.	United Kingdom	Qualitative	14 health visitors	London borough	Refugees and asylum seekers	In-depth interviews	Thematic framework (Ritchie & Spencer, 1994)	To determine the barriers to effective practice that health visitors when working with refugees and asylum seekers.	1.Ineffective engagement<br>2.Stretched resources	> Health professionals share innovative ways of working to in order to reduce the barriers experienced by refugees and asylum seekers.<br>> Increase awareness among primary health care staff of entitlement to health services for this particular client group.<br>> Commissioners should have an awareness of barriers to effective practice when deciding how to invest in services for vulnerable populations.
Burchill J, Pevalin DJ. Demonstrating cultural competence within health-visiting practice: working with refugee and asylum-seeking families. Diversity Equality Health Care 2014 06;11(2):151-159 9p.	United Kingdom	Qualitative	14 health visitors	London borough	Refugees and asylum seekers	In-depth interviews	Thematic framework (Ritchie & Spencer, 1994)	Explored the experiences of health visitors working with refugee and asylum-seeking families in central London, and assessed the dimensions of their cultural competency using Quickfall's model (Quickfall, 2004, 2010)	1.Institutional regard<br>2.Cultural awareness<br>3.Cultural sensitivity<br>4.Cultural knowledge<br>5.Cultural competence	> Health visitors need to be able to demonstrate cultural competence in their practice with refugee and asylum-seeking families.

Study Reference	Country of study	Study type	Total Participants in Study	Health professional practice setting	Service users	Data collection method	Analysis method	Study aims/purpose (Author's words)	Reported results (Themes/main headings)	Recommendations/applications (Author's words)
Carolan M, Cassar L. Pregnancy care for African refugee women in Australia: attendance at antenatal appointments. Evid Based Midwifery 2007 2007;5(2):54-58 5p.	Australia	Qualitative	2 midwives<br>10 African women*<br>1 community worker*<br>1 interpreter*<br>1 family and reproductive rights education program worker*	African women's clinic within a community health centre	African refugee women	Observational methods and Semi-structured interviews	Not explicitly stated, but think perhaps Thematic Analysis	To explore factors that facilitate or impede the uptake of antenatal care among African refugee women.	1.Staff attitudes<br>2.Availability of interpreters<br>3.Knowledge about the clinic at community level<br>4.Convenient location of the clinic	>Community midwifery clinics might offer a solution in terms of providing an acceptable and sensitive service to refugee African women. This familiar service would allow the women to meet the same carers on each visit, which would facilitate the development of trust.<br>>Opportunity for the clinic staff to tailor services to identified needs, such as the provision of interpreters in specific languages, liaison with medical and midwifery specialists with a knowledge of African disease and access to social and community workers.
Crowley P. The mental health needs of adult asylum seekers in Newcastle upon Tyne. Journal of Public Mental Health 2005;4(1):17-23.	United Kingdom	Mixed methods	10 general practitioners<br>67 asylum seekers (quantitative)*<br>? asylum seekers (qualitative)*<br>? managers*<br>? mental health service providers*<br>? housing support*<br>? agency staff*<br>? voluntary sector service providers*<br>? interpreters*	General practice and community	Asylum seekers	Interviews<br>Telephone interviews<br>Focus groups	Unspecified	To assess the mental health care needs of adult asylum seekers in Newcastle upon Tyne.	A. Quantitative<br>1. Demographic information<br>2. Mental illness prevalence in primary care<br>3. Mental illness prevalence in the general population<br>4. Mental health service use<br>B. Qualitative<br>1. Asylum seekers<br>2. Housing support workers and interpreters<br>3. Voluntary sector service providers<br>4. GP practices<br>5. Mental health service providers and managers<br>6. Regional and national agencies	> Increase opportunities for self-sufficiency; developing social support; developing peer groups; strengthening links with the host community; tackling racial harassment; improving economic well-being, and facilitating communication with families.<br>> Primary care practices need more education, training, support and resource to meet the needs of asylum seekers effectively, and to address the issue of hostility from other patients.<br>> There is a need both to improve mental health services and to strengthen social and other forms of support both within the communities to which asylum seekers belong and within host communities.<br>>In Newcastle, weaknesses in policy and practice in the mental health trust require attention in the light of the overall need to develop mental health services that best meet the need of the whole population.<br>> A greater level of sensitivity to the mental health needs of asylum seekers is required across the public sector, together with recognition of the major impact that experience in the host country has on their mental health and well-being.
Drennan VM, Joseph J. Health visiting and refugee families: issues in professional practice. J Adv Nurs 2005 01/15;49(2):155-163 9p.	United Kingdom	Qualitative	13 health visitors	2 inner London borough's	Refugees and asylum seekers	Semi-structured interviews	Framework method (Ritchie and Spencer 1994)	Describe health visitors' experiences working in Inner London and identifying and addressing the health needs of refugee woman in the first 3 months after the birth of a baby.<br>Investigate health visitors' perceptions of effective and ineffective strategies in identifying and addressing health needs of these women.<br>Investigate whether health visitors used a framework corresponding to Maslow's theory of a hierarchy of needs to prioritize their public health work.	1. Complexity of the relationship between health visitors and clients who are refugees.<br>2. Identification and prioritization of the health needs of the asylum seeking and refugee families.<br>3. Health visitors' perceptions of successful outcomes of their work.<br>4. Impact on health visitors of working with asylum seekers and refugees.	> There is a service and professional responsibility to ensure that health visiting and public health nursing practice is developed from the best evidence available and that collective knowledge and expertise are shared, rather than left for each practitioner to discover through trial and error.<br>> Both professional education providers and service providers need to pay attention to the specific health and social needs of asylum seeking women, who will unfortunately continue to arrive in the UK and other parts of the world.
Farley R, Askew D, Kay M. Caring for refugees in	Australia	Qualitative	20 general practitioners	General practice	Newly arrived refugees	Focus groups and	Thematic analysis	Explored the experiences of primary health care	1. Communication<br>2. Knowledge	> Increase range of resources available in languages other than English. Support English education for

Study Reference	Country of study	Study type	Total Participants in Study	Health professional practice setting	Service users	Data collection method	Analysis method	Study aims/purpose (Author's words)	Reported results (Themes/main headings)	Recommendations/applications (Author's words)
general practice: perspectives from the coalface. Australian Journal of Primary Health 2014;20(1):85-91.			5 practice Nurses<br>11 administrators*			semi-structured interviews		providers working with newly arrived refugees in Brisbane...focusing on the barriers and enablers they continue to experience in providing care to refugees.	3. Practice and health care systems	refugees. Support providers in understanding the linguistic backgrounds of their patients. Consider the importance of literacy in English education for refugees Improve availability and quality of visual resources. Raise awareness of refugees' limited literacy among providers. Increase interpreter service availability across all health care sectors (including allied health). Improve medical interpreter training. Provide information for providers regarding cultural differences in communication and the impact this can have on a consultation.<br>> Provide focussed education and training around important refugee health issues.<br>> Provide mental health training for providers, particularly in relation to caring for victims of past torture and trauma Improve supports available to providers working in this area, through access to trained psychologist and bicultural workers. Enhance psychologists' access to interpreters.<br>> Provide initial refugee health care in a specialised refugee health setting and ensure effective communication and support at the time of referral and beyond. Provide a forum for the exchange and transfer of experiences, information and resources between providers working in this area. Provide focussed education and training for providers, regarding the health care system as it pertains to refugee health care. Identify and adequately resource relevant support organisations. Consider methods to adequately remunerate providers (e.g. Medicare payments when interpreters are used). Provide case workers with appropriate training to assist in coordinating care.<br>> Provide education for refugees around health care within the Australian health care system.
Feldmann CT, Bensing JM, de Ruijter A. Worries are the mother of many diseases: General practitioners and refugees in the Netherlands on stress, being ill and prejudice. Patient Educ Couns Mar 2007;65(3):369-380	Netherlands	Qualitative	66 refugees*<br>24 general practitioners	General practice	Refugees (Afghan & Somali)	In-depth interviews	Not specified	To confront the views of refugee patients and general practitioners in the Netherlands, focusing on medically unexplained physical symptoms (MUPS).	1. Perspectives of refugees<br>-General narrative versus personal narratives<br>-Refugees' concepts of health and illness<br>-Causes of illness—mental worries<br>-Personal responsibility—strategies to stay healthy<br>-Expectations from doctors<br>-Refugees' problems with doctors<br>2. The general practitioners' perspective<br>-General practitioners on refugee problems<br>-How doctors deal with refugee problems<br>-Human interest strategy	>For a fruitful cooperation to develop, based on trust, GPs need to invest in the relationship with individual refugees, and avoid statements or actions based on stereotypes and prejudice. There is a heartening parallel between refugees' expectations and GPs' best practices.<br>> Direct observation, visual registration and later (qualitative) analysis of consultations between general practitioners and refugee patients, combined with eliciting refugees' expectations and level of trust before the consultation, and both the GPs' and the refugees' assessments afterwards, can help to raise awareness of possibilities for improvement in specific practices.<br>>Early investment in the relationship with new refugee patients may be crucial to establishing a basis of trust and dealing with unexplained physical symptoms effectively.<br>>Asking (refugee) patients about their situation and the way they are dealing with it, separate from the complaint that is being presented, helps to create an atmosphere of joint responsibility.

Study Reference	Country of study	Study type	Total Participants in Study	Health professional practice setting	Service users	Data collection method	Analysis method	Study aims/purpose (Author's words)	Reported results (Themes/main headings)	Recommendations/applications (Author's words)
									-Technical strategy<br>-Elements that occur in both 'human interest' and 'technical' strategies	>A physical complaint always deserves a thorough physical examination.<br>>The tendency to stereotype refugee patients may be a serious pitfall for practitioners.<br>>Critical reflection by practitioners is needed on strategies they employ for dealing with unexplained physical symptoms.<br>>Professional errors by medical practitioners have a long life circulating as part of the 'general narrative' in refugee communities, undermining trust. A more open climate when dealing with professional mistakes, especially towards the patients involved and their relatives, may help to address this phenomenon.
Furler J, Kokanovic R, Dowrick C, Newton D, Gunn J, May C. Managing depression among ethnic communities: A qualitative study. Annals of Family Medicine May-Jun 2010;8(3):231-236.	Australia	Qualitative	8 family physicians	Community health centre	Refugees with depression	Semi-structured interviews	Thematic analysis (Mays & Pope 1995)	Explores the complexities of this work [clinical care for depression] through a study of how family physicians experience working with different ethnic minority communities in recognizing, understanding, and caring for patients with depression.	1. Understanding and negotiating the problem of depression<br>2. Managing the depression<br>3.Working with the interpreter	>Highlight the need for more detailed observational research of clinical care for depression across a range of primary care settings and contexts.
Griffiths R, Emrys E, Lamb CF, Eagar S, Smith M. Operation Safe Haven: The needs of nurses caring for refugees. Int J Nurs Pract Jun 2003;9(3):183-190.	Australia	Qualitative	13 nurses<br>1 medical records clerk*<br>2 nursing managers	Refugee reception centre	Refugees	2 focus groups (13 nurses + 1 clerk), Semi-structured interviews (2 nurse managers)	Thematic analysis	To identify the skills, knowledge and support nurses require to provide holistic and competent care to refugee children and their families and the nature of support that is required to assist their transition back to mainstream health services.	1. Clinical skills and knowledge required by Safe Haven nursing staff.<br>2. Cultural competency skills<br>3. Trauma-sensitive care<br>4. Stressors impacting on Safe Haven nurses<br>5. Sources of support for Safe Haven nurses<br>6. Rewards<br>7. Return to work	>Counselling (for Nurses) should be provided by qualified, on-site counsellors with good understanding of trauma-related issues.<br>>Nursing workforce planners need to be able to employ appropriate numbers of permanent staff for extended disaster operations, avoiding the need for excessive work hours or the unsustainable practice of 'partial secondment', where nurses are expected to carry out disaster-type work and maintain their existing work responsibilities.<br>>Nursing workforce planners should undertake strategic recruitment during extended disaster operations, identifying appropriately skilled workers to form a stable workforce offering continuity of care.<br>>Disaster planners at the Area Health Service level should identify appropriate external agencies and designated health providers to assist with clinical management during extended operations, where nurses work with increased autonomy.
Jensen NK, Norredam M, Priebe S, Krasnik A. How do general practitioners experience providing care to refugees with mental health problems? A qualitative study from Denmark. BMC Family Practice 2013;14:17.	Denmark	Qualitative	9 general practitioners	Medical clinics with high proportion of immigrants	Refugees	Semi-structured interviews	Content analysis (Graneheim and Lundman 2004)	To qualitatively explore issues identified by general practitioners as important in their experiences of providing care for refugees with mental health problems.	1. Communication<br>2. Quality of care<br>3. Referral pathways<br>4. Understandings of disease and expectations of treatment.	>The findings from this study suggest that there is an increased need for general practitioners to be aware of potential traumas experienced by refugee patients, but also leave room for taking individual differences into account in the consultation. This could be attained by the development of conversational models for general practitioners including points to be aware of in the treatment of refugee patients. This may serve as a support in the health care management of refugee patients, but at the same time does not disregard the resources of individual refugee patients.

Study Reference	Country of study	Study type	Total Participants in Study	Health professional practice setting	Service users	Data collection method	Analysis method	Study aims/purpose (Author's words)	Reported results (Themes/main headings)	Recommendations/applications (Author's words)
Johnson D.R., Ziersch A.M., Burgess T. I don't think general practice should be the front line: Experiences of general practitioners working with refugees in South Australia. Australia and New Zealand Health Policy 2008;5(pagination):Arte Number: 20. ate of Pubaton: 08 Aug 2008.	Australia	Qualitative	12 general practitioners<br>3 medical directors of divisions of general practice*	General practice	Refugees	Semi-structured interviews	Template analysis	To document the existence and nature of challenges for GPs who do this work in SA.<br>To explore the ways in which these challenges could be reduced.<br>To discuss the policy implications of this in relation to optimising the initial health care for refugees.	1. Challenges for GPs<br>a)Refugee health issues<br>-GP knowledge of previous health assessments<br>- GP awareness of and experience managing health conditions unique to refugees<br>- The multiple and complex nature of refugee health conditions<br>b) GP-refugee interaction<br>- Issues related to culture<br>- Issues related to language<br>- Refugee knowledge of the Australian healthcare system<br>c) Structure of general practice<br>- GP workforce shortages<br>- Referral systems<br>- Remuneration<br>- Infrastructure supports to perform initial assessments<br>2. Challenges for Divisions assisting GPs<br>3. Ways GPs could be better supported<br>a) Providing GPs with more resources<br>b) Providing initial refugee health care via a specialist service	>Utilise a specialist service for refugees in refugees' resettlement period, which could provide initial health assessments and expertise in working with this population.<br>> If initial health assessments are provided by a specialist service, it is important that a clear, transparent and effective referral system to a nominated general practice is part of this process when initial health care needs have been met.
Kokanovic R, May C, Dowrick C, Furler J, Newton D, Gunn J. Negotiations of distress between East Timorese and Vietnamese refugees and their family doctors in Melbourne. Sociol Health Illn May 2010;32(4):511-527.	Australia	Qualitative	5 general practitioners<br>24 refugees from Vietnam and East Timor*	Community health centre	Refugees	In depth interviews	Thematic analysis	We explore a set of cultural boundaries across which depression is contested: between recent migrants to Australia from East Timor and Vietnam, and their white 'Anglo' family doctors. We are concerned with the ways that the experiences of migration and its aftermath are manifest in the lives of people from these ethnic groups; how their consequent distress is negotiated and contested in their interactions with family doctors; and how the	1. The journey and the arrival are important<br>2. Home and family: here and there<br>3.The naming of parts: manifestations of and bringing distress into the medical encounter<br>4. Illness Labels: naming distress	> Reinvestigate the way of conducting research on depression in a cross-cultural context.

Study Reference	Country of study	Study type	Total Participants in Study	Health professional practice setting	Service users	Data collection method	Analysis method	Study aims/purpose (Author's words)	Reported results (Themes/main headings)	Recommendations/applications (Author's words)
								resulting collisions affect the meaningfulness of the concept of depression.		
Kurth E, Jaeger FN, Zemp E, Tschudin S, Bischoff A. Reproductive health care for asylum-seeking women - a challenge for health professionals. BMC Public Health 2010;10:659.	Switzerland	Mixed-Methods. Quantitative element, based on patient files, explored frequencies of diagnoses and medical interventions. Qualitative element analysed data from asylum seekers patient notes and interviews with health professionals.	80 asylum seekers*<br>3 physicians<br>3 nurse/ midwife<br>1 psychologist*<br>3 interpreter*	Women's clinic	Female asylum seekers	Semi-structured Interviews with the 10 health professionals.<br>Textual data was extracted from the 80 asylum seeker's patient files.<br>The quantitative element extracted data from hospital electronic database and patient files.	Grounded theory methodology	The aim of the present study was to investigate the reproductive health care provided for women asylum seekers attending the Women's Clinic of the University Hospital in the city of Basel, Switzerland.<br>To identify the health needs of asylum seekers attending the Women's Clinic and to investigate the health care they received in a Health maintenance organisation (HMO) specifically established for asylum seekers.<br>Explored the perceptions of the health care professionals Involved about providing health care for this group in this setting.	1. Language and cultural barriers<br>2. Conflicting roles of physicians<br>Unclear how these themes were chosen from all of the data	> Specific training and support for health care providers.<br>> Training and support are needed not only because of the emotional challenges resulting from the situation, but because the patients do not only need medical care, but very often suffer from severe psychosocial problems arising from the stressful situation they are in.<br>>Attention should also be paid to stressors that could potentially affect health professionals and their work: the need for support and training of health care providers caring for vulnerable populations should be investigated further.<br>> The effect on health care providers of working in a restrictive HMO setting, where they do not only have to carry out their traditional clinical tasks but must also cope with increasing managerial responsibilities and financial restrictions, may also warrant further study.<br>> Language barriers can be overcome with the use of well-trained professional interpreters - both for the patients' sake and to avoid frustration in health care providers.
Lawrence J, Kearns R. Exploring the 'fit' between people and providers: refugee health needs and health care services in Mt Roskill, Auckland, New Zealand. Health & Social Care in the Community 2005 Sep;13(5):451-461.	New Zealand	Qualitative	5 community representatives*<br>9 refugee group representatives*<br>5 medical practitioners<br>1 manager*<br>1 administrator*	Community health centre	Refugees	Semi-structured Interviews	Thematic analysis	This paper reports on research that sought to reveal the barriers faced by refugees in accessing health services, and the challenges faced by providers in endeavouring to meet needs in an effective and culturally appropriate manner.	1. Population change within the Roskill area<br>2. Refugee perspectives on barriers to accessing health services<br>- Resettlement issues<br>-Differing cultural understanding of illnesses and health care systems<br>-Distrust of others<br>- Difficulties in communication<br>-Cost<br>-Physical access difficulties<br>3. Experiences of health practitioners in delivering health services to refugees	>The changing social landscape of larger Western cities...demands a greater attentiveness to the health needs of a population and the health services in place at a neighbourhood level<br>>In Mt Roskill...further adjustments in terms of funding, staffing, training and the style of patient/professional contact seem a necessary prerequisite for advancing health and social care in the community.<br>>There is clear need for funded health educators to provide a comprehensive orientation on such matters at the time of their registration at a service like HoP.<br>>Many of the delays and frustrations experienced by both the users and providers of services would be addressed by the funding of appropriate translation services.<br>>We advocate an enhanced commitment to developing cultural awareness through incorporating social-scientific perspectives to complement biomedical knowledge in medical education.<br>>To achieve this responsiveness [to community demographics], maintaining an elected board comprising both community and clinic representatives, as well as developing relationships with sympathetic researchers, can assist in bridging what otherwise might be a gulf between clinic and community.
Riggs E, Davis E, Gibbs L, Block K, Szwarc J,	Australia	Qualitative	87 refugee background	Maternal and child health (MCH)	Refugee background	Focus groups	Thematic analysis	This study aims to explore the utilisation	1. Facilitating access to MCH services.	> Provision of refugee focussed training for service providers and a strategically coordinated approach is

Study Reference	Country of study	Study type	Total Participants in Study	Health professional practice setting	Service users	Data collection method	Analysis method	Study aims/purpose (Author's words)	Reported results (Themes/main headings)	Recommendations/applications (Author's words)
Casey S, et al. Accessing maternal and child health services in Melbourne, Australia: Reflections from refugee families and service providers. BMC Health Serv Res 2012 01;12(1):117-117 1p.			mothers*<br>12 nurses<br>1 community worker*<br>1 community liaison*<br>5 bilingual workers*<br>3 community representatives*<br>2 managers of bilingual workers*	service	mothers	(refugees, nurses, bilingual workers, community worker, community liaison) and individual interviews (community representatives, managers of bilingual workers)		and experience of MCH services in Melbourne, Victoria for parents of refugee background from the perspective of users and providers.	2. Promoting continued engagement with the MCH service.<br>3. Language challenges.<br>4. What is working well and what could be done better?	likely to facilitate access, build rapport and ongoing engagement and retention to the service for families of refugee background.<br>> Innovative culturally competent strategies to organise individual MCH service appointments should be trialled and evaluated to develop a MCH system that promotes refugee maternal and child health.<br>> Trial a model where MCH nurses attend venues where refugees already gather to promote MCH services, provide information and build trust.<br>> The role played by bicultural workers should be recognised and utilised in a way that benefits clients and service providers.<br>> MCH services could proactively work in partnership with bilingual community workers to call clients directly to make appointments. Where these workers are not available, interpreters could also be utilised for this purpose.
Samarasinghe K, Fridlund B, Arvidsson B. Primary health care nurses' promotion of involuntary migrant families' health. Int Nurs Rev 2010;57(2):224-231.	Sweden	Qualitative	34 primary health care nurses	Various primary health care settings: maternity, child, school and community health care, and nurse-led clinics covering asthma, allergy, diabetes and hypertension	Involuntary migrants	Interviews	Contextual analysis (Phenomenography)	The aim of this study was to describe the promotion of health in involuntary migrant families in cultural transition as conceptualized by Swedish PHCNs.	1. Category I. An ethnocentric approach focusing on the physical health of the individual<br>2. Category II. An empathic approach focusing on the mental health of the individual in a family context<br>3. Category III. A holistic approach empowering the family to function well in everyday life	> In orientating families to cultural values of host country, teaching new cultural behaviours must be carried out in a respectful way so that the families do not feel subjected to forced assimilation.<br>> having family conversations with the entire family about the impact of acculturation on interpersonal relationships may be helpful in strengthening family relations.<br>> To enhance family health and family cohesion, nurses need to facilitate involuntary migrant families' cultural transition by empowering the family to be in control of acculturation.<br>>For nurses to enhance family health during cultural transition, adequate education encompassing the development of intercultural communication skills and cultural self-awareness must be available at both undergraduate as well as post-graduate level on a national basis.<br>>In clinical practice, the implementation of family-focused nursing incorporating supportive conversations about acculturation and adaptation will be useful.
Suurmond J, Rupp I, Seeleman C, Goosen S, Stronks K. The first contacts between healthcare providers and newly-arrived asylum seekers: A qualitative study about which issues need to be addressed. Public Health Jul 2013;127(7):668-673.	Netherlands	Qualitative	36 nurse practitioners<br>10 public health physicians	Asylum seeker centres	Newly arrived asylum seekers	Group interviews	Framework	To describe the tacit knowledge of Dutch healthcare providers about the care to newly arrived asylum seekers and to give insight into the specific issues that healthcare providers need to address in the first contacts with newly arrived asylum seekers.	1. Investigation of the current health condition of asylum seekers<br>2. Assessment of health risks<br>3. Providing information about the health care system<br>4.Health education	> In education and training this rough framework thus can be used as a means to reflect upon priorities in health care to asylum seekers as well as being aware of possible pitfalls, dilemmas and difficulties.<br>> Potential aspects of training: the need for good communication skills (including the skill to work with a professional interpreter) to deal with cultural differences and to deal with possible high expectations of asylum seekers.<br>>Training may help care providers reflect upon their own boundaries of their medical profession: for example, should they be the ones to assess mental health problems of asylum seekers or is it better to refer to another institution with more relevant competencies?<br>>Sufficient time is needed for a consultation when all four elements are included.

Study Reference	Country of study	Study type	Total Participants in Study	Health professional practice setting	Service users	Data collection method	Analysis method	Study aims/purpose (Author's words)	Reported results (Themes/main headings)	Recommendations/applications (Author's words)
										>Reference to other types of care, such as mental health care, need to be ascertained, before care providers assess asylum seekers' needs such as mental health needs.<br>>Different issues may be addressed by different professionals (for example, assessing mental health problems may be done by a psychologist, health education may be done by a health educator).
Suurmond J, Seeleman C, Rupp I, Goosen S, Stronks K. Cultural competence among nurse practitioners working with asylum seekers. Nurse Educ Today 2010 11;30(8):821-826 6p.	Netherlands	Qualitative	89 nurse practitioners for survey element. 36 nurse practitioners in group interviews.	Asylum seeker centres	Asylum seekers	Questionnaires and group interviews	Framework	We explored the cultural competences that nurse practitioners working with asylum seekers thought were important.	1. Training and education in cultural competence<br>2. Knowledge of the political and humanitarian situation in the country of origin<br>3. Knowledge of epidemiology and the manifestation of diseases in asylum seekers' countries of origin<br>4. Knowledge of the effects of refugeehood on health<br>5. Awareness of the juridical context in which asylum seekers live<br>6. Skills to develop a trustful relationship with an asylum seeker<br>7. Ability to ask delicate questions about traumatic events and personal problems.<br>8. Ability to explain what can be expected from health care<br>9. Improving cultural competence	> These results add more specific competences to the cultural competences that have been described in other studies.<br>> It is not merely education or training that helps nurse practitioners feel culturally competent. Equally significant is the concrete experience of working with asylum seekers. This suggests that 'learning in action' by way of adequate supervision, mutual peer supervision, and systematic feedback on the work floor may also be a key teaching instrument. Thus, experiential and didactic learning may be integrated in order to develop relevant cultural competences.<br>> Cultural competences should not be seen as a list of skills that are acquired and ticked off one at a time, resulting in a person who is culturally competent. Acquiring cultural competence is an ongoing process, driven by the practitioners' self-reflection.
Tellep TL, Chim M, Murphy S, Cureton VY. Great suffering, great compassion: A transcultural opportunity for school nurses caring for Cambodian refugee children. Journal of Transcultural Nursing Oct 2001;12(4):261-274.	United States	Qualitative	6 school nurses<br>2 Cambodian liaisons*	Schools	Refugees	Focus group	Not specified	To describe the nature and meaning of school nurses' and Cambodian liaisons' experiences of caring for Cambodian refugee children and families and to explore whether those meanings validated Dobson's (1989) conceptual framework of transcultural health visiting.	1. Transcultural health-visiting education<br>2. Intracultural reciprocity<br>3. Transcultural reciprocity<br>4. Goal of maximising health and wellbeing: Letting go of one's own views<br>5. Multifaceted roles of Cambodian liaisons: We want to help them in any way<br>6. School and home: "Caught in the middle"<br>7. Intergenerational conflict: "It's hard for the kids"<br>8. The Cambodian	>Awareness of transcultural reciprocity and the importance of establishing trust may help guide other nurses in the development of meaningful relationships with Cambodian refugee children and families.<br>> Transcultural nursing care should be incorporated into all stages of the nursing process when caring for Cambodians.<br>> In partnership with the Cambodian community, interventions that target Cambodian refugee children with direct services, as well as indirect services through support of their families, are needed.<br>>Collaboration with others outside the school setting is vital to creating a cross-cultural team approach of coordinated and comprehensive service to Cambodian refugee children and families.<br>>Individualize care based on family's background and refugee history.<br>>Keep reaching out; trust takes time.<br>>Take a slow, friendly, no direct spiralling approach.

Study Reference	Country of study	Study type	Total Participants in Study	Health professional practice setting	Service users	Data collection method	Analysis method	Study aims/purpose (Author's words)	Reported results (Themes/main headings)	Recommendations/applications (Author's words)
									refugee experience: "Left for dead"<br>9. Spiritual healing: "It lifts your spirits"<br>10. Cultural strengths: Carried across the ocean"	>Gently probe.<br>>Suspend assumptions and worldview.<br>>Look beyond the behavior to understand the underlying dynamic.<br>>Support cultural traditions and share your interest<br>>Elicit explanatory models for illness.<br>>Incorporate spiritual healing practices and the temple into delivery of health services.<br>>Encourage and mentor Cambodian role models.<br>>Provide health education: family planning, nutrition, safety, and routine check-ups.<br>>Assist with access to care.<br>>Provide support to parents and elders.<br>>Assess refugee risk factors as part of special education process.<br>>Monitor medications.
Tobin C.L., Murphy-Lawless J. Irish midwives' experiences of providing maternity care to non-Irish women seeking asylum. International Journal of Women's Health 2014 31 Jan 2014;6(1):159-169.	Ireland	Qualitative	10 midwives	Maternity hospitals	Female asylum seekers	In-depth unstructured interviews	Content analysis	To explore midwives' perceptions and experiences of providing care to women in the asylum process and to gain insight into how midwives can be equipped and supported to provide more effective care to this group in the future.	1. Barriers to communication<br>2. Understanding cultural difference<br>3. Challenges of caring for women who were unbooked<br>4. The emotional cost of caring,<br>5. Structural barriers to effective care.	>For women in the asylum process, having access to dedicated community-based services would begin to address the problems of access, late booking, and development of midwife/client relationships which in turn would help to decrease fear and anxiety for both the women themselves and the midwives who care for them.<br>>Cultural competency training: When considering how best to educate midwives to provide culturally competent care, the most important focus should be on using a framework of cultural humility.<br>> There is an urgent need for increased clinical support for midwives who care for traumatized women.<br>>Access to continuing education is also essential, along with debriefing and clinical supervision in order to maintain providers' own health and well-being.<br>> Trained interpreter service should be embedded within hospitals.<br>>dedicated community-based services that provide the possibility of continuity of care, make access to care easier for women, and provide the possibility of good midwife/client relationships and trust building.<br>> Revision of the government policy of forced dispersal for women in the asylum process who are pregnant or in the early postpartum period is urgently needed.
Twohig PL, Burge F, MacLachlan R. Pod people. Response of family physicians and family practice nurses to Kosovar refugees in Greenwood, NS. Canadian Family Physician 2000 Nov;46:2220-2225.	Canada	Qualitative	6 family practice nurses<br>10 family physicians	Clinic in refugee processing centre	Refugees	Semi-structured interviews	Textual analysis	To explore roles of family physicians and family practice nurses who provided care to Kosovar refugees at Greenwood, NS.	1. Clinical encounter<br>2. Expectation and experience<br>3. Roles and team functioning<br>4. Response	> Future responses to emergency situations might benefit from clearer descriptions of individual roles within the team.
Yelland J, Riggs E, Wahidi S, Fouladi F, Casey S, Szwarc J, et al. How do Australian	Australia	Mixed Methods. Interviews conducted	30 Afghan parents*<br>10 midwives<br>5 medical	Mixed Methods. Interviews conducted with Afghan parents	Refugee background	Interviews and focus groups	Thematic analysis	(1) investigate Afghan women and men's experience of the way that health professionals	1. Language services in the context of care<br>2. Women and men's experience of being	>Our findings support calls for standardised procedures to improve identification of people of refugee background in clinical settings.<br>>Building an understanding of the refugee experience,

Study Reference	Country of study	Study type	Total Participants in Study	Health professional practice setting	Service users	Data collection method	Analysis method	Study aims/purpose (Author's words)	Reported results (Themes/main headings)	Recommendations/applications (Author's words)
maternity and early childhood health services identify and respond to the settlement experience and social context of refugee background families?. BMC Pregnancy & Childbirth 2014;14:348.		with Afghan parents contained a quantitative element.<br>No reported quantitative element in interviews with health professionals.	practitioners*<br>19 Community based health professionals*	contained a quantitative element.<br>No reported quantitative element in interviews with health professionals.				approach inquiry about social factors affecting families having a baby in a new country, and (2) investigate how health professionals identify and respond to the settlement experience and social context of families of refugee background.	asked about social health issues<br>3. Identifying and responding to social health issues: the experience of health professionals	what health care providers need to be mindful of in providing care to families of refugee background, and knowledge of services for referral, is likely to go some way in building workforce capacity to assess and respond to the social circumstances of refugees.<br>>Interactive training opportunities incorporating knowledge of the refugee and asylum seeker experience and ways of working with these families is a strategy to enhance health professionals understanding and skills.<br>>Any attempts to improve the responsiveness of health services to the needs of families of refugee background need to consider innovative ways to work within system constraints.
Yelland J, Riggs E, Szwarc J, Casey S, Duell-Piening P, Chesters D, et al. Compromised communication: a qualitative study exploring Afghan families and health professionals' experience of interpreting support in Australian maternity care. BMJ Qual Saf 2016 Apr;25(4):e1-2014-003837. Epub 2015 Jun 18	Australia	Qualitative	30 Afghan parents*<br>10 midwives<br>5 medical practitioners*<br>19 Community based health professionals*	Various maternity care services	Refugee background	Interviews and focus groups	Thematic analysis	(1) describe Afghan women's and men's experiences of language support during pregnancy check-ups, labour and birth; (2) explore health professionals' experiences of communicating with Afghan and other refugee clients with low English proficiency; and (3) consider implications for health services and health policy.	1. The use of accredited interpreters in maternity care<br>2. Family members interpreting during pregnancy, labour and birth	> Improving identification of language needs at point of entry into healthcare, developing innovative ways to engage interpreters as integral members of multidisciplinary healthcare teams and building health professionals' capacity to respond to language needs, especially when clients' have experienced trauma that is likely to impact on their capacity to engage with healthcare, are critical to reducing social inequalities in maternal and child health outcomes for refugee and other migrant populations.<br>>Potential 'solutions' in the context of maternity care include community and language-specific group pregnancy care sessions combining antenatal check-ups with information and support provided by a multidisciplinary team of health professionals including an accredited interpreter.
* These participants are not within the study definition of primary health care professionals and therefore their data have not been included in the thematic synthesis.										
