# Supplementary material for: Challenges and facilitators for health professionals providing primary healthcare for refugees and asylum seekers in high-income countries: a systematic review and thematic synthesis of qualitative research
Source: BMJ Open. 2017 Aug 4;7(8):e015981. doi: 10.1136/bmjopen-2017-015981 (PMC5629684; doi:10.1136/bmjopen-2017-015981)
Supplement: Supplementary material 4 [file bmjopen-2017-015981supp004.pdf]

Supplement 4: Quality assessment of studies included in the thematic synthesis

Study reference*	Aims & methods	Research design	Sampling†	Data collection	Reflexivity	Ethical issues	Data analysis†	Discussion of findings	Value
Begg H, Gill PS. Views of general practitioners towards refugees and asylum seekers: an interview study. Diversity Health Soc Care 2005 12;2(4):299-305 7p.	Research aims not clearly articulated. Importance and relevance considered. Qualitative methodology is appropriate to capture General practitioner's views.	Use of qualitative design not explicitly justified.	17 general practitioners. "Purposeful sampling was used to recruit GP's, with more or less than 10% of the area population from the black and minority ethnic communities" and areas cross checked with the Refugee council. Age and ethnicity were not controlled for. One hundred GPs were randomly selected from the target locations using computer generated numbers, and approached via post and a follow-up phone call. Of these, 20 GPs volunteered to participate but 17 were actually interviewed as three opted out at the last minute due to work priorities.	Semi-structured Interviews conducted at GP practices by the author. No justification given for methods or setting of data collection. A previously piloted and refined topic guide was utilised with topics listed No detail on how data was recorded. Data collection terminated upon saturation of emergent themes.	Researcher considered the potential influence of her age (medical student), sex (female) ethnicity (as from ethnic minority) in the openness of participants. In addition, recruitment bias was considered (people with stronger opinions more likely to respond). Also discussed the reasons for volunteers opting out not being related to study aims.	No detail on how the study was explained to participants. Written consent was obtained prior to the commencement of each interview, and confidentiality maintained throughout. Lacking details on how researchers handled issues raised for participants by the study. Ethical approval was obtained from North West Multisite Research Ethics Committee.	A thematic framework analysis was conducted. "Data collection and analysis proceeded simultaneously, incorporating emergent themes into subsequent interviews. Emergent themes were compared by HB and PG independently before agreement and refinement of the themes." Did not contain a description of how data presented was selected. Sufficient data were presented to support the findings. Contradictory data were taken into account Researcher highlights the use of multiple coding to reduce bias in the analysis along with respondent validation.	The findings were explicit and clearly discussed. As mentioned in Q1, the research question is not clearly defined. The findings are discussed in the context of the wider literature. Credibility enhanced by respondent validation and multiple analysts.	Briefly considered the value of the study and contribution to research (highlighted some important issues surrounding the delivery of care to refugees and asylum seekers) identified areas for further research (lack of time, support, education, training and, financial resources) Acknowledges the limitations in generalisability as conducted in one metropolitan area.
Bennett S, Scammell J. Midwives caring for asylum-seeking women: research findings. Pract Midwife 2014 Jan;17(1):9-12.	Aims clearly stated with explanation of how the findings would be used to inform policy, education and practice. Qualitative methodology is appropriate for exploration of midwives experiences of caring for asylum seekers.	Use of qualitative design not explicitly justified.	10 midwives. The study was targeted at qualified midwives who had practised for a minimum of one year and had some experience of working with asylum-seeking women. Midwives were recruited via an email sent by the Head of midwifery; 10 volunteered to participate. All those who volunteered were included in the sample. Not clear whether there was a process to check eligibility of volunteers. Non-participation was not discussed.	Semi-structured interviews. Lacking details about the setting and who conducted the interviews. No justification given for methods or setting of data collection. No explicit reporting of how the interviews were conducted and the areas of enquiry. Interviews were audio recorded and transcribed. Data saturation not discussed.	The researcher's role and potential bias in the formulation of questions or data collection was not discussed. An 'audit trail' was kept, capturing influences, events, actions and decisions taken during the conduct of the study.	All participants were provided with information about the study and gave written consent. States that all the participants were volunteers and free to withdraw at any time. Confidentiality was not discussed. Lacking details on how researchers handled issues raised for participants by the study. Ethical approval was gained from the trust and NHS National Research Ethics Service.	"A thematic analysis was used to capture emerging patterns of data. These were reviewed and grouped into two overarching themes and four interconnected sub-themes. Rigour was maintained through a systematic process of enquiry, sampling and analysis." No indication of involvement of multiple researchers in the analysis. Sufficient data were presented to support the findings. Contradictory data not discussed. No examination of researcher's role, potential bias and influence during the analysis and in presentation of the data. Only some of the themes from the analysis are reported in this paper.	The findings were explicit and clearly discussed in relation to the original research question and within the context of the wider research literature. No discussion of the credibility of the research and did not report whether multiple researchers were involved in coding transcripts or interpretation of findings.	Considered the value of the study and the contribution of the research. Did not make suggestions for future research. Considered the generalisability of the findings. Provided a number of recommendations for practice, education and policy.
Burchill J. Safeguarding vulnerable families: work with refugees and asylum seekers.	No clear statement of research aims. Importance and relevance of	Use of qualitative design not explicitly	14 health visitors. Purposive sampling was used in which participants were selected for their	In-depth interviews were conducted at multiple health centres across the borough (number not	Author acknowledges that there may have been bias related to	Research aims were explained at a professional meeting of health	A thematic framework method was utilised that involved a constant comparative approach in which codes and transcripts	The findings were explicit and clearly discussed in relation to the original research	Considered the value of the study in raising awareness of commissioners to

Study reference*	Aims & methods	Research design	Sampling†	Data collection	Reflexivity	Ethical issues	Data analysis†	Discussion of findings	Value
Community Practitioner 2011 Feb;84(2):23-26.	research adequately stated.<br>Qualitative methodology is appropriate for understanding health visitor's experiences of working with refugees and asylum seekers.	justified.	ability to contribute to the data. Recruitment was conducted by approaching potential participants through a presentation at a professional meeting.<br>Sample was approximately 1/3 of all health visitors in the borough.<br>Participants required to have worked for 2yrs as would be highly likely to have worked with refugees and asylum seekers.<br>No discussion about the reasons why some health visitors chose not to participate.	specified), but unclear who conducted the interviews.<br>No justification given for methods or setting of data collection.<br>A topic guide used that had been developed from a literature review.<br>Participants were asked primarily to describe their experiences of working with refugees and asylum seekers and what problems/difficulties they faced.<br>Method of recording interview not described but states that interviews were transcribed.<br>Data saturation not discussed.	the fact that he worked in the same workplace (colleagues) as the participants.<br>Participants may not have been as open or willing to tell the truth in interviews.	visitors.<br>Lacking discussion about how consent was gained, confidentiality maintained and how issues raised by the study were handled by researchers.<br>Approval to proceed with the study was granted by the Primary Care Trust research and development team and the Local Research Ethics Committee.	were constantly reassessed and re-interpreted. Themes identified were compared across the data and interpretations discussed with external researchers.<br>No reported duplicate coding.<br>Quotations were chosen to illustrate the particular issues described<br>Sufficient data were presented to support the findings.<br>Contradictory findings were not presented.<br>No examination of researcher's role, potential bias and influence during the analysis and in presentation of the data.	question. Limited discussion of findings in the context of the wider literature.<br>No explicit discussion of the credibility of the research, but methods report that interpretation was discussed with external researchers.	provide appropriate services for refugees and asylum seekers.<br>No further research areas suggested.<br>No explicit discussion of transferability to other populations but suggests the findings will be useful for commissioners in other settings and that the study adds to literature that can inform policy and practice.
Burchill J, Pevalin D. Barriers to effective practice for health visitors working with asylum seekers and refugees. Community Practitioner 2012 Jul;85(7):20-23.	Research aims clearly stated.<br>Importance and relevance were articulated.<br>Qualitative methodology is appropriate for understanding barriers to effective practice for health visitors working with refugees and asylum seekers.	Use of qualitative design not explicitly justified.	14 health visitors.<br>Purposive sampling was used in which participants were selected for their ability to contribute to the data. Recruitment was conducted by approaching potential participants through a presentation at a professional meeting.<br>Participants required to have worked for 2yrs as would be highly likely to have worked with refugees and asylum seekers.<br>No discussion about the reasons why some health visitors chose not to participate.	In-depth interviews were conducted at multiple health centres across the borough in which the participants worked (Number of centres not specified). Unclear who conducted the interviews.<br>No justification given for methods or setting of data collection.<br>A topic guide used that had been developed from a literature review and consisted of a number of broad statements that would help guide the interview.<br>The interviews were taped and transcribed verbatim.<br>Data saturation not discussed.	No critical examination of the researcher's role, potential bias and influence in research question formulation or data collection.	Research aims were explained at a professional meeting of health visitors.<br>Lacking discussion about how consent was gained, confidentiality maintained and how issues raised by the study were handled by researchers.<br>The Primary Care Trust and the Local Research and Ethics Committee granted approval for this study.	A framework method was used that involved a constant comparative approach in which the codes were continually reassessed and interpreted.<br>The themes that were identified were compared across the data and discussed with external researchers.<br>Quotations were chosen to illustrate the particular issues described.<br>Sufficient data were presented to support the findings.<br>Contradictory data were not presented.<br>No examination of researcher's role, potential bias and influence during the analysis and in presentation of the data.	The findings were explicit and clearly discussed in relation to the original research question and within the context of the wider research literature.<br>No discussion of the credibility of the research.	Discusses the contribution of the study in increasing awareness in primary health care staff of health service entitlements of refugees and asylum seekers.<br>Also raises awareness for commissioners of barriers to effective services when deciding how to invest in appropriate services.
Burchill J, Pevalin DJ. Demonstrating cultural competence within health-visiting practice: working with refugee and asylum-seeking families. Diversity Equality Health Care 2014 06;11(2):151-159 9p.	The aims of the research clearly stated<br>The importance and relevance of the research were articulated<br>Qualitative methodology is appropriate to explore health visitor's experiences of working with	Use of qualitative design not explicitly justified.<br>Authors describe the purpose and key features of the Framework approach that they have	14 health visitors.<br>A presentation was given at the health visitors' main professional meeting with details of the study and an invitation to participate.<br>Participants had to be qualified health visitors and worked in the borough for over 2 years - ensuring that they had enough experience. Sample size was 14/42 health visitors	In-depth interviews were conducted at multiple health centres across the borough in which the participants worked (Number of centres not specified). Unclear who conducted the interviews.<br>A topic guide used that had been developed from a literature review and consisted of 10 broad open-ended	No critical examination of the researcher's role, potential bias and influence in research question formulation or data collection.	Potential participants approached at a professional meeting of health visitors. All confirmed participants were sent an information letter and consent form to be signed before	Framework analysis. "Each interview was first transcribed and then analysed using Framework. This involved a constant comparative approach throughout. The themes that were identified were compared across the data, and interpretations were discussed between the interviewer (JB) and external researchers consisting of an academic supervisor and a doctoral	The findings were explicit and discussed in relation to the research question.<br>Findings not discussed in the context of the wider literature.<br>No explicit discussion of the credibility of the research, but methods report that interpretation was discussed with	The author discusses the contribution of the study to existing knowledge. Concludes that aspects of cultural competence are lacking, but are being addressed at the local level.<br>Identifies the need for research into models of cultural competence in a variety of primary

Study reference*	Aims & methods	Research design	Sampling†	Data collection	Reflexivity	Ethical issues	Data analysis†	Discussion of findings	Value
	refugees and asylum seekers.	chosen for the analysis.	working in the borough. No discussion about the reasons why some health visitors chose not to participate.	statements. The topic guide was given to participants prior to the interview. No justification given for methods or setting of data collection. A tape recorder was used to record the interview, which was transcribed for the analysis. Data saturation not discussed.		participation in the study. Lacking discussion of how confidentiality was maintained. "Each participant was offered debriefing at the end of the interview session to discuss any issues that might have arisen, particularly if any difficult experiences were referred to." Ethical approval was granted by the local NHS Research Ethics Committee, and research governance permission was gained from the Primary Care Trust Research and Development Team.	student." Sufficient data are presented to support the findings. Contradictory data were taken into account. No examination of researchers own role, potential bias and influence during the analysis and in presentation of the data.	external researchers.	care settings. Discusses the generalisability of the results and highlights the limitations of the model used for this research study for other health care settings.
Carolan M, Cassar L. Pregnancy care for African refugee women in Australia: attendance at antenatal appointments. EVID BASED MIDWIFERY 2007 2007;5(2):54-58 5p.	The aims of the research clearly stated. The importance and relevance of the research were articulated. A qualitative methodology is appropriate for understanding factors that facilitate or impede uptake of antenatal care among refugee communities.	Researchers justified their choice of study methods. The use of observational methods before the semi structured interviews could help the researcher gain cultural understandin g and build trust with the participants.	10 African women, 2 midwives, 1 family reproductive rights education program worker, 1 interpreter. African women: Recruitment was facilitated by the midwife, who asked women attending the clinic if they were interested in the study. Those indicating an interest were approached by the researcher and the nature of the study, time requirements and study purpose were explained. Women who were still interested were invited to participate. No discussion about the reasons why some people chose not to participate Clinic staff: No description of how the clinic staff were selected for interview. No explanation as to why this clinic was an appropriate place to	Data were collected in two phases. Phase 1 was 40 hours of observation at the women's clinic by a researcher. Phase 2 employed semi-structured interviews with staff and refugee women. Setting of data collection was African Women's Clinic. Unclear who conducted the interviews. No justification given for methods or setting of data collection. Areas of enquiry in the interviews are described. Researchers modified the questions asked in the interviews with attending women when it became apparent they did not understand questions. Field notes were used to record observation element. Specific method of data recording during interviews not	No critical examination of the researcher's role, potential bias and influence in research question formulation or data collection.	Potential participants were approached by the researcher, who explained the nature and purpose of the research and the time commitment. Participant's names were changed in the reporting of the study, but not clear whether this was explained to the participants. No discussion of informed consent or how researchers handled issues raised by the study for participants. The project was approved by university and hospital ethics committees.	Exact method used for data analysis not specified. Brief description of analysis process. "data analysis then proceeded through the following stages: Organising the data; Immersion in the data; Generating categories and themes; Coding the data; Offering interpretations; Seeking alternative explanations. Notes of analytical understandings and decisions were made throughout the process. Trustworthiness of findings was enhanced by asking two academic colleagues to independently generate a theme list." No explanation of how the data presented were selected from original sample. Sufficient data are presented to support the findings. Contradictory data are taken into account in the findings. No examination of researcher's role, potential bias and influence during the analysis and in presentation of the data.	The findings were explicit, discussed with reference to the research question and set within the context of the wider literature. The authors state that the trustworthiness of the findings are enhanced by asking two academic colleagues to independently generate a theme list during the analysis.	Authors suggest that community midwifery clinics might offer a solution for providing acceptable and sensitive services to refugee African women. Findings considered in relation to relevant research-based literature. No further research areas are suggested. Transferability not discussed, but implied that similar healthcare services could be effective in other settings with refugee women.

Study reference*	Aims & methods	Research design	Sampling†	Data collection	Reflexivity	Ethical issues	Data analysis†	Discussion of findings	Value
			sample.<br>Non-participation not discussed	stated but transcription is mentioned.<br>Data saturation not discussed.					
Crowley P. The mental health needs of adult asylum seekers in Newcastle upon Tyne. Journal of Public Mental Health 2005;4(1):17-23.	Aims of the study were clearly stated and its importance and relevance articulated.<br>The qualitative element of this study was an appropriate methodology to explore perceptions of the causes of mental ill health among asylum seekers and investigate issues in delivering services to this group.	The use of qualitative methodology was not explicitly justified, but the purpose of interviews and focus groups was explained.	10 general practitioners and unspecified numbers of other participants (asylum seekers, managers, mental health service providers, housing support, agency staff, voluntary sector service providers, Interpreters)<br>Exact numbers of participants not reported.<br>No details given about how participants were selected for focus groups or interviews.<br>No justification given for the choice of these participants	Interviews, telephone interviews and focus groups were used to collect qualitative data, but no details about the interviewer(s). Lacking details of the setting of data collection, but some participants were interviewed by telephone.<br>Researcher justifies the use of some of the focus groups and interviews, but not the setting of data collection.<br>No details about how the interviews were conducted.<br>No details about how data were recorded during the interviews/focus groups.<br>No discussion of data saturation.	The researcher's role and potential bias in the formulation of questions or data collection was not discussed.	No details given about how the research was explained to participants.<br>No discussion of informed consent, confidentiality, or how issues raised in the course of study were handled by researchers.<br>Approval from an ethics committee is not reported.	No description given of the analysis process or whether multiple researchers were involved in the analysis.<br>Not clear how findings were derived from the data<br>Insufficient data are presented to support the findings.<br>Contradictory data were not taken into account.<br>No examination of researcher's role, potential bias and influence during the analysis and in presentation of the data.	The findings are explicit and discussed in relation to the research question<br>The findings are discussed in the context of the wider literature.<br>The credibility of the findings are not discussed	The author discusses the contribution of the study to existing knowledge, practice and policy.<br>No identification of new areas for research.<br>No discussion of whether the findings can be transferred to other populations.
Drennan VM, Joseph J. Health visiting and refugee families: issues in professional practice. J Adv Nurs 2005 01/15;49(2):155-163 9p.	The aims of the research clearly stated.<br>The importance and relevance of the research were articulated.<br>A qualitative methodology is appropriate to understand the perceptions of health visitors working with refugees and asylum seekers.	Authors had formulated a hypothesis that health visitors framed their work with refugee and asylum seeking women using Maslow's hierarchy of need. The study was undertaken to explore this hypothesis.<br>No justification of the specific qualitative methods employed.	13 health visitors.<br>The participants were recruited by purposive sampling. Health visitors who identified themselves as having a significant number of refugees and asylum seekers on their caseloads and had worked in inner London for more than 5 years and were currently working with refugees and asylum seekers.<br>No discussion about whether some people chose not to participate and their reasons.	Data were collected through semi-structured interviews, conducted at the health visitor's places of work. Unclear who conducted the interviews<br>No justification given for methods or setting of data collection.<br>"Broad, open ended questions were used in the interview, inviting informants to be discursive and reflective in recounting their experiences.". Areas of enquiry in the interviews are described.<br>Interviews lasted 45min-1hr.<br>Interviews were tape-recorded and subsequently transcribed.<br>Data saturation was not discussed.	No critical examination of the researcher's role, potential bias and influence in research question formulation or data collection.	"Participants…given a full information sheet about the purpose, methods and use of the study". "Formal written consent was obtained and participants were assured that their data would be anonymized and deleted after transcription. "Participants were sent draft copies of the report to demonstrate that anonymity had been preserved".<br>Lacking details on how researchers handled issues raised for participants by the study.<br>Ethical approval was obtained from	Framework method was used to analyse data. "The theoretical issues identified in the literature were used to devise the coding framework. The interviewer and second author independently coded the transcripts against the framework; using word processing and spreadsheet functions software. Additional codes were assigned as the data suggested new themes and issues. A small number of discrepancies in coding between the two analyses were resolved through subsequent discussion. The coded material was then analysed for: (a) Commonalities between informants, (b) conflicting perceptions between informants and (c) evidence to support or disprove the use of a hierarchy of needs in framing practice."<br>Sufficient data are presented to support the findings.<br>Contradictory data are taken into account.<br>No examination of researcher's	The findings were explicit and discussed with reference to the research question.<br>Minimal discussion of the findings in relation to the wider literature.<br>The credibility of the research is not explicitly discussed, but the two authors independently coded transcripts against the framework with discrepancies resolved through discussion. In addition, participants were sent draft copies of the report for comment.	Briefly considers the value of the study. The author acknowledges that the single geographical setting and small sample size limit the conclusions.<br>The contribution of the study to existing knowledge and understanding is discussed.<br>Identified one possible avenue for further research - whether prioritization of children's needs over mothers could be another issue related to Maslow's pyramid.<br>They suggest that although the study was UK based, the issues raised in the study will likely resonate for public health nurses working in other countries.

Study reference*	Aims & methods	Research design	Sampling†	Data collection	Reflexivity	Ethical issues	Data analysis†	Discussion of findings	Value
						the local Research Ethics Committee.	role, potential bias and influence during the analysis and in presentation of the data.		
Farley R, Askew D, Kay M. Caring for refugees in general practice: perspectives from the coalface. Australian Journal of Primary Health 2014;20(1):85-91.	The aims of the research clearly stated. The importance and relevance of the research were articulated. Qualitative methodology is appropriate for exploring the experiences of primary health care providers working with refugees.	Use of qualitative design not explicitly justified.	20 general practitioners, 5 practice nurses, 11 administrators. Researchers explain how the participants were selected. 6 general practices were purposively selected on the basis that they had received newly arrived refugees in the past 6 months. Purposive sampling ensured that participating practices had experience of caring for refugees. Practices were approached by a researcher to discuss involvement in the project, which was followed up by a phone call to clarify involvement. No discussion of the proportion of practice staff that agreed to participate in the research or any reasons for non-participation.	5 Focus groups and 4 semi-structured interviews were used. The exact setting for data collection is not clear, but occurred during staff lunch breaks. RF facilitated the focus groups and conducted the semi-structured interviews. Authors justified the use of some semi-structured interviews as a way of overcoming time constraints for some participants and for testing whether focus groups were effective in surfacing the key themes. The setting was not justified. A standard introduction and interview schedule informed by the literature was used to stimulate conversation and discussion, but unclear whether this was for the focus groups, interviews or both. Brief description of the types of questions used. Authors report modification of methods in the study. Semi-structured interviews were used when time constraints prevented a focus group occurring and when a participant missed a focus group. Focus groups and interviews were audio recorded and transcribed. Data saturation is discussed.	It was acknowledged that personal relationships and power differentials in the workplace may have impacted on individual's freedom to express opinions in the focus groups. The authors were aware of this potential and took steps to minimise this. (offering opportunity to provide confidential feedback). Both researchers were working in refugee health and were aware of potential for influencing data collection and interpretation. To minimise this, a clear statement of the role of the researcher was explained to participants in the preamble to data collection.	Practices were provided with information sheets, confidentiality agreement and consent forms. Informed consent was obtained from each participant before involvement. Lacking details on how researchers handled issues raised for participants by the study. Ethical approval was granted by the Mater Health Services Human Research Ethics Committee.	"Key themes were identified using inductive thematic analysis and NVivo software was used to assist with data management. Analysis was iterative and data collection ceased when no new issues emerged, suggesting data saturation. RF and MK read each transcript and independently coded data, identifying a preliminary list of themes. RF produced a refined list of major themes and subthemes; MK endorsed these themes. Because similar themes were identified during the focus groups and interviews, the data were considered comparable and therefore analysed together." Sufficient data were presented to support the findings. Some Contradictory data were presented in the findings. Authors were aware of the potential bias in data analysis and stated that they critically reflected on how their own views and differing perspectives were influencing interpretation. One of the authors worked outside the field and was able to bring more objectivity.	The findings were explicit and clearly discussed in relation to the research question. Adequate discussion of the findings in relation to the wider literature. The researchers discuss the use of more than one analyst enhancing the credibility of the study. In addition, anonymised transcripts were provided to participants to give an opportunity for any further feedback.	The researcher provides an extensive list of recommendations for practice in relation to each of the main themes identified in the study. The research builds on the body of literature that focusses on the refugee perspective. Further areas for research are identified It is implied that this research will be able to help inform refugee healthcare on a national level although it is acknowledged that this research was carried out in one healthcare model.
Feldmann CT, Bensing JM, de Ruijter A. Worries are the mother of many diseases: General practitioners and refugees in the Netherlands on stress, being ill and prejudice.	The aims of the research clearly stated. The importance and relevance of the research were articulated. Qualitative	Authors state that "we set up an open ended, explorative study to learn about their frames of	66 refugees, 24 general practitioners. Refugee participants were approached through refugee initiated community organisations, Dutch Council for Refugees and personal networks (at least	Refugees: In-depth interviews were conducted by the first author (female former GP) with the help of female Somali or Afghan researchers. Setting for collection of data not	Researcher's role, potential bias and influence in research question formulation or data collection.	Lacking details about how the research was explained to participants. Interviews were conducted with consent from the	Refugees: "The first author analysed and coded the transcripts of the refugee interviews, using the WinMAX software program to organise the data and facilitate retrieval... After initial coding and cross-sectional comparison, a	The findings are explicit and discussed in relation to the research question. Authors discuss the findings in relation to the wider literature. No discussion of the	The contribution of the study to inform healthcare practice is discussed. A number of practice implications are given. Potential new areas of research are

Study reference*	Aims & methods	Research design	Sampling†	Data collection	Reflexivity	Ethical issues	Data analysis†	Discussion of findings	Value
Patient Educ Couns Mar 2007;65(3):369-380	methodology is appropriate for investigating the views of refugee patients and general practitioners about medically unexplained physical symptoms.	reference, expectations and experiences concerning health and healthcare."	partially purposive). Most GPs were a convenience sample from a letter sent to 325 GPs. 3 GPs were selected through personal contacts. Not clear what criteria were applied at the recruitment stage, but the refugees sample was shown to be diverse and representative. GPs had significant experience of caring for this group (21 had > 5 years' experience caring for Somali and Afghan refugees).<br>No discussion around non-participation.	described.<br>No justification given for methods or setting of data collection.<br>Topic list used that was developed in consultation with refugee experts and used in a flexible way. It was adapted during data collection, adding issues that seemed important. Areas of enquiry are described.<br>Data recorded on tape and transcribed verbatim.<br>Data saturation not discussed.<br>GPs: Semi-structured interviews with open ended questions were conducted by a medical student (22) and the first author (3). Setting is not fully described, but reported that 12 were conducted on the telephone and 12 face-to-face at a place of participants' preference. States that the GP participants were likely to give a more positive response towards refugees as they were willing to make time for the interview.<br>No justification given for methods or setting of data collection.<br>Not clear whether a topic list was used for these interviews or the areas of enquiry covered.<br>3 interviews were tape recorded and transcribed verbatim. 21 were recorded through note-taking with the interviewer conscientiously elaborating on them immediately afterwards.<br>Data saturation not discussed.		participants. No discussion about how confidentiality was maintained or how issues raised through the study were handled by researchers.<br>No reference to ethics committee reported.	schematic presentation in short quotes was made of each refugee interview"<br>GPs: "The GP interviews were analysed and coded in the same way. A short profile was written for each doctor, linking interview results to doctor and practice variables. In an initial analysis, rough codes were assigned for the doctors' perceptions of the refugee groups, the problems the refugees presented to them, the way they dealt with these problems, and the constraints they met."<br>A secondary analysis was performed on both refugee and GP data with further content analysis, which formed the body of the article.<br>Sufficient data are presented to support the findings.<br>Contradictory data were taken into account.<br>No examination of researcher's role, potential bias and influence during the analysis and in presentation of the data.	credibility of the findings.	suggested.<br>No discussion of transferability to other populations.
Furler J, Kokanovic R, Dowrick C, Newton D, Gunn J, May C. Managing depression among ethnic	The aims of the research were stated.<br>Research question does not define	Use of qualitative design not Justified, however, in	8 family physicians. Participants were included as part of a larger study known as 'Re-order', but lacking details on how they	Semi-structured interviews conducted by one of the authors (RK) and a research assistant. Lacking details about the	No critical examination of the researcher's role, potential bias and influence in research	Insufficient details about how the research was explained to participants.	"Three authors read transcripts and analysed them independently to identify themes and categories. Results were compared and discrepancies	The findings were explicit and clearly discussed in relation to the research question. The evidence from the	Discusses the findings in relation to practice of physicians and their approach to working with depressed patients

Study reference*	Aims & methods	Research design	Sampling†	Data collection	Reflexivity	Ethical issues	Data analysis†	Discussion of findings	Value
communities: A qualitative study. Annals of Family Medicine May-Jun 2010;8(3):231-236.	participants as refugees, but throughout the study it is apparent that they are refugees. The importance and relevance of the research were articulated. Qualitative methodology is appropriate to understand experiences of family physicians that work with patients with depression.	the discussion section, Authors state that the findings would not be found through conventional studies of medical records, billing records or patient reports.	were recruited. Explained that the participants were chosen because they were known to work extensively with a range of refugee and migrant communities (Table 1 displays length of time they had worked with these communities). No discussion about reasons for non-participation.	exact location of data-collection No justification given for methods or setting of data collection. Brief explanation of the areas covered in the interviews, but the full interview schedule is provided in an on-line appendix. Interviews lasted 1-1.5 hours and were audio-recorded and transcribed. Data saturation not discussed.	question formulation or data collection.	Lacking discussion about how consent was gained, confidentiality maintained and how issues raised by the study were handled by researchers. Ethical approval for the study was granted by the University of Melbourne Human Research Ethics Committee.	discussed with the wider group, and concepts were further refined. Additional thematic categories were added as the analysis developed." Authors emphasise that transparency in analysis and reporting was achieved by providing extensive verbatim quotes and independent assessments of transcripts and themes. Sufficient data were presented to support the findings. Contradictory data were not presented in the findings No examination of researcher's role, potential bias and influence during the analysis and in presentation of the data.	wider literature is discussed in relation to the findings of the study. Authors acknowledge that the sample was small and that the physicians were working with specific cultural groups. They also mention that 3 authors were involved in the thematic analysis and themes were discussed with the wider group.	in ethnic communities. Suggest areas for future research Lacking discussion about the transferability of the findings of the study or other ways the research could be used.
Griffiths R, Emrys E, Lamb CF, Eagar S, Smith M. Operation Safe Haven: The needs of nurses caring for refugees. Int J Nurs Pract Jun 2003;9(3):183-190.	The aims of the research clearly stated. The importance and relevance of the research were articulated. Qualitative research is an appropriate methodology to ascertain the needs of nurses that worked with refugees arriving from conflict areas.	Use of qualitative design not explicitly justified.	13 nurses, 1 medical records clerk, 2 nurse managers. Researcher explains that all the nurses and midwives employed at the centre during its 14-month operation were invited to participate in focus group discussions (Convenience sampling). 14 positive responses were received, which included a medical records clerk. Unclear how the two nurse managers were chosen for semi-structured interviews. Unclear why some people did not participate in the study, but the authors hypothesise that it could have been due to the distance from residence to study location, nurses no longer working in the same workplace or unable/unwilling to participate.	Data was collected through 2 focus groups (13 nurses and 1 medical records clerk) and 2 semi-structured interviews (Nurse managers). No information is given about the settings of data collection or the researcher(s) that conducted interviews. No justification given for methods or setting of data collection. For focus groups, an interview schedule developed by the researchers was used to guide discussion. 5 areas of discussion were described that were triggered by interview questions. Semi-structured interviews lasted 60-9-0 min and followed another format developed by the researchers, but lacking detail on the areas of discussion. Data were audio-recorded and transcribed Data saturation not discussed.	No critical examination of the researcher's role, potential bias and influence in research question formulation or data collection.	Lacking details about how the research was explained to participants and how consent was gained. To protect confidentiality, all participants were assigned a pseudonym. Lacking details on how researchers handled issues raised for participants by the study. Ethics approval was obtained from the South Western Sydney Area Health Service Research Ethics Committee and the University of Western Sydney Ethics Review Committee.	"Thematic analysis of focus group and in-depth interview transcripts was undertaken by a multidisciplinary research team, who re-read them several times to become immersed in the data. The team, drawing upon informants' stories of their experiences, then generated broad themes common throughout the text. Themes and emerging subthemes identified by the research team were then coded from the transcripts using a qualitative data management program (QSR Nvivo, QSR International)." Sufficient data were presented to support the findings, however the authors did not include many quotations. Some contradictory data are presented in the findings No examination of researcher's role, potential bias and influence during the analysis and in presentation of the data.	The findings are explicit and discussed in relation to the original research question. The findings are discussed within the context of the wider evidence in the literature. No explicit discussion of the credibility of the results, but authors report that a team conducted the thematic analysis implying multiple researchers involved in generating themes from the data.	The contribution of the study to practice within similar settings is discussed. Several recommendations are given for health care providers to improve support for nurses caring for refugees. Authors discussed how the findings might be relevant in other contexts and further research areas are suggested.
Jensen NK, Norredam M, Priebe S, Krasnik A. How do general	The aims of the research clearly stated.	Use of qualitative design not	9 general practitioners. The participants were purposively selected based	Semi-structured interviews took place at the workplace of the	No critical examination of the researcher's role,	The research was explained to the participants in a	Qualitative content analysis was undertaken. "The interviews were read several times to	The findings are explicit and discussed in relation to the	The researchers briefly consider the findings in the context of national

Study reference*	Aims & methods	Research design	Sampling†	Data collection	Reflexivity	Ethical issues	Data analysis†	Discussion of findings	Value
practitioners experience providing care to refugees with mental health problems? A qualitative study from Denmark. BMC Family Practice 2013;14:17.	The importance and relevance of the research were articulated. Qualitative methodology is appropriate for exploring general practitioner's experiences of providing care for refugees with mental health problems.	explicitly justified.	on working in clinics with high proportions of immigrants and were expected to have a high experience of working with immigrant and refugee patients. The research was explained to participants in a letter which was followed up with a phone call with further details and to inquire about their interest in taking part in the study. No discussion of non-participation.	professionals and carried out by the first author. No justification given for methods or setting of data collection Methods for data collection are described. An interview guide was developed by a project coordinating group in London (study was part of a broader EU project) and translated into Danish for use in this study. The first part of the interview included questions around delivery of care to immigrants in general. The second part began with a vignette (scenario of a refugee patient consultation), with pre-prepared questions to begin discussion. Interviews were recorded on a Dictaphone and transcribed. Data saturation was not discussed.	potential bias and influence in research question formulation or data collection.	letter, with more details being given in a phone call. Informed consent was obtained orally from all participants and they were ensured anonymity. Lacking details on how researchers handled issues raised for participants by the study. Ethical permission for this study has been waived by the Ethical Committee of the Capital Region of Denmark as Danish legislation does not require ethical approval for this type of study.	obtain a sense of the whole. The text was then divided into meaning units, which were then condensed and assigned categories and themes in a process moving towards a higher level of abstraction. The creation of categories and themes took place as an iterative process with ongoing reflection and revision of categories and themes. The whole context of the interviews was considered concurrently throughout this process. The initial analysis was carried out by the first author, but presented to and discussed with co-authors and other researchers with a background in public health, medicine and anthropology as part of the analytic process." Sufficient data are presented to support the findings. Contradictory data are presented and discussed. No examination of researcher's role, potential bias and influence during the analysis and in presentation of the data.	original research question. Findings are discussed within the context of evidence in the wider literature. Credibility of findings not explicitly discussed, but the author mentions that the initial stages of the analysis were conducted by the lead author and as themes emerged, they were discussed with the wider group including members from different discipline backgrounds.	policy for health care management of refugees. Briefly suggests ways to improve practice. Suggest the development of conversational models for general practitioners with points to be aware of in consultations with refugees. There is some discussion of the transferability of the results. The authors acknowledge that the participants had high levels of knowledge about refugees and asylum seekers, which is not true of many general practitioners. In addition, the vignette used for the interview gave a theoretical, isolated situation, which they acknowledge may limit generalisability.
Johnson D.R., Ziersch A.M., Burgess T. I don't think general practice should be the front line: Experiences of general practitioners working with refugees in South Australia. Australia and New Zealand Health Policy 2008;5(pagination):Arte Number: 20. ate of Pubaton: 08 Aug 2008.	The aims of the research clearly stated. The importance and relevance of the research were articulated. Qualitative methodology is appropriate to explore the challenges for GPs working with refugees.	Researchers justify the use of qualitative methodology. A qualitative approach was taken in order to gain a deeper understandin g of the challenges faced by general practitioners in private practice when providing care to refugees.	12 general practitioners and 3 medical directors of divisions. Potential participants were identified through a database of GPs who could be identified as having accepted refugee referrals. One of the authors also used his personal knowledge from previous related work. Further GPs were identified after interviews with medical directors of divisions. An introductory letter/invitation was sent to 77 potential GP participants, providing 6 participants. the remaining six were recruited through follow up phone calls. Medical directors of divisions were contacted by email with 2 agreeing to participate with a further participant agreed after a follow up phone call. These	Data were collected through semi-structured interviews. Most were conducted individually, but 3 of the GPs were conducted together in a group setting. No description of the setting for data collection or who conducted interviews. Use of semi-structured interviews was justified. They were able to examine challenges already identified in the literature as well as allowing new themes to emerge. No justification of setting. Lacking detail on how the interviews were conducted, but does briefly outline the general focus of the questions for the GPs and the medical directors of divisions. The interviews were tape recorded and transcribed	No critical examination of the researcher's role, potential bias and influence in research question formulation or data collection.	Lacking details about how the research was explained to participants and how consent was gained. Confidentiality was protected by assigning participants random numbers in the coding process. Lacking details on how researchers handled issues raised for participants by the study The study was approved by the University of Adelaide Human Research Ethics Committee.	"A template analysis approach was adopted where a coding template was developed which included a priori themes in addition to new themes identified from initial reading and analysis of the transcripts. Final thematic templates for both the GP and Division transcripts were agreed upon by the Project Team and then all data was coded according to these themes, with DJ undertaking the bulk of the coding. Two transcripts were also independently coded by the other members of the Project Team. Following this, comparisons were made and a consensus reached on how the remaining data was to be coded." Sufficient data are presented to support the findings. Contradictory data not presented No examination of researcher's role, potential bias and influence	The findings are explicit and discussed in relation to the original research question. Findings are discussed within the context of evidence in the wider literature. Lacking discussion of the credibility of the findings 2 out of 15 transcripts were independently coded by multiple researchers.	Considered the findings of the study in relation to practice and policy. Suggested that to provide more generalisable results a quantitative study should be conducted, but does not give any information about the aims of such a study. The authors discuss the transferability of the study and state that the small numbers limit its generalisability.

Study reference*	Aims & methods	Research design	Sampling†	Data collection	Reflexivity	Ethical issues	Data analysis†	Discussion of findings	Value
			were contacted because their areas were thought to contain high levels of refugee settlement.<br>Discusses the low response rate and some of the potential bias around those who did participate (i.e. participants more likely to be dissatisfied with current system of provision), however the researchers believe that there were also limited numbers of GPs with experience working with refugees.	verbatim.<br>Researchers state that data saturation was reached.			during the analysis and in presentation of the data.		
Kokanovic R, May C, Dowrick C, Furler J, Newton D, Gunn J. Negotiations of distress between East Timorese and Vietnamese refugees and their family doctors in Melbourne. Sociol Health Illn May 2010;32(4):511-527.	The aims of the research clearly stated.<br>The importance and relevance of the research were articulated.<br>Qualitative methodology is an appropriate methodology to explore how migration experiences are manifested in the lives of the participants and how resulting distress is negotiated and contested in their interaction with family doctors.	Use of qualitative design not explicitly justified, however the choice of in-depth interviews was justified as it allowed enough time for respondents to talk about their lives in their own words and focus on issues that were important to them.	5 general practitioners, 24 refugees from Vietnam and East Timor.<br>The refugee participants were purposively selected to include patients who had experienced depression and had used health services for depression care. They were recruited if they had been diagnosed with depression or prescribed antidepressants in the past year.<br>Lacking details about how refugee participants were first contacted, but the initial approach involved use of interpreters to explain the study. Those agreeing to be contacted by the research team were telephoned by a bilingual researcher with more information and to arrange a time for the interview.<br>Unclear how the GPs were selected or recruited to the study.<br>Authors give characteristics of the participants that suggest that these were an appropriate sample (10-25 years' experience).<br>No discussion around non-participation.	In-depth interviews were conducted. Most interviews were conducted in the Community Health Centre, but some (number not reported) of the refugees were interviewed in their homes. Interviews conducted by experienced qualitative researchers (first author and research fellow)<br>Researchers justified use of in-depth interviews as it allowed enough time for respondents to talk about their lives in their own words and focus on issues that were important to them.<br>Setting not justified<br>Authors report the use of an interview guide for a section of the interviews, but unclear about the full methods used with the refugees and the GP's.<br>The differing areas of discussion with refugees and GP's were outlined.<br>Interpreters were utilised for the majority of the interviews with refugees.<br>Data were audio-recorded and research notes were kept by the interviewer and interpreter. These were translated and transcribed in duplicate	Researchers discuss the complexities of interviewing using translators and the impact on researcher-interviewee communication. It is uncertain whether interpreters may have summarised or modified questions, answers and meanings.	Research was explained to refugees in their own language (through interpreters) at the initial contact and then in more detail in a telephone call. Unclear how the research was explained to GPs.<br>Consent was gained from all participants using consent forms in English or translations into relevant languages. No discussion of confidentiality or how issues raised through the stud for participants were handled by researchers.<br>Ethical approval was given by the University of Melbourne Human Ethics Research Committee.	An inductive thematic approach was taken.<br>The themes from the preliminary coding were used to create a coding frame which was applied to the data across all transcripts. The transcripts were marked and annotated, and emerging themes were then discussed among authors.<br>Unclear who and how many people coded the transcripts.<br>Sufficient data are presented to support the findings.<br>Researchers refer to contradictory data within their dataset.<br>No examination of researcher's role, potential bias and influence during the analysis and in presentation of the data.	The findings of the study were explicit, discussed in relation to the research question and set in context of the wider literature.<br>No explicit discussion of the credibility of the findings, but it is reported that transcripts were translated in duplicate and multiple researchers were involved in developing emerging themes.	Limited discussion of the contribution of the findings to practice or policy. Authors do suggest a reinvestigation of the way of conducting research on depression in a cross-cultural context.<br>Researchers point out that the issues around negotiation of distress investigated in this paper are broadly relevant in (cross-cultural) clinical practice.

Study reference*	Aims & methods	Research design	Sampling†	Data collection	Reflexivity	Ethical issues	Data analysis†	Discussion of findings	Value
				(to maximise legitimacy). Data saturation was not discussed.					
Kurth E, Jaeger FN, Zemp E, Tschudin S, Bischoff A. Reproductive health care for asylum-seeking women - a challenge for health professionals. BMC Public Health 2010;10:659.	Aims of the study were clearly stated and its importance and relevance articulated. A qualitative study is appropriate to explore the perceptions of health care professionals providing health care to asylum seeking women.	Use of qualitative design not explicitly justified.	80 asylum seekers, 3 physicians, 3 nurse/ midwife, 1 psychologist, 3 interpreters Purposive sample. The people who were invited to participate were those who had been most involved with the asylum-seeking patients insured in the Health Maintenance Organisation (HMO) model - a service specifically set up for asylum seekers in Basil University Hospital. All the professionals invited agreed to participate.	Semi-structured interviews were conducted in a quiet hospital room of the participant's choice. Information about interviewers is not reported. No justification given for methods or setting of data collection. An interview guide was used. Areas of enquiry are described. Additional questions were asked to physicians about roles specific to them. The interviews were audio recorded and transcribed verbatim. Data saturation was not discussed.	The researcher's role and potential bias in the formulation of questions or data collection was not discussed.	Participants were informed about the aims of the study and they signed a consent form. Lacking details about how confidentiality was maintained or how researchers handled issues raised for participants by the study The study was approved by the joint ethical committee of the Cantons of Basel Stadt and Basel Land (Ethikkommission beider Basel).	Analysis followed steps of grounded theory methodology. "We started the process by open coding, which means that we categorized text segments into broad categories or themes … We continued with axial coding which included examining relationships between categories and connecting them accordingly…Finally, selective coding included the organisation of the diverse categories into a framework to explain the phenomenon under study. This framework is depicted and explained in details in the result section. To strengthen the rigour of the analysis, we discussed the results with experts in women's health, ethics and research." Sufficient data are presented to support the findings. Contradictory data not taken into account No critical examination of researcher's role and influence in the analysis	The findings of the study were explicit, discussed in relation to the research question and in context of the wider research literature Cross validation of quantitative and qualitative elements was thought to add to credibility.	Considered the value of the study and the findings in relation to practice and policy. Suggests areas for further research. Researchers discussed the limitations of the small sample size and the research being conducted in a hospital setting with highly developed services. Authors suggest that challenges could be greater in other settings.
Lawrence J, Kearns R. Exploring the 'fit' between people and providers: refugee health needs and health care services in Mt Roskill, Auckland, New Zealand. Health & Social Care in the Community 2005 Sep;13(5):451-461.	Aims of the study were clearly stated and its importance and relevance articulated. Qualitative methodology is appropriate for investigating barriers faced by refugees in accessing health services and challenges faced by providers to meet their needs.	Use of qualitative design not explicitly justified, however the authors explain why they chose in-depth interviews as a data collection method.	5 Community representatives, 9 Refugee group representatives, 5 Medical practitioners, 1 Manager, 1 Administrator. Participants were purposively selected in consultation with staff at the clinic. Community representatives selected based on length of involvement in the Mt Roskill community. Refugee representatives were representative of ethnic groups in the area and chosen based on involvement with the community. All seven members of staff at the clinic were sampled. No discussion around non-participation of community representatives.	In-depth interviews were conducted with participants with most taking place at the clinic and some in the workplace of representatives. All interviews were conducted by the first author. Authors justify their use of in-depth interviews: "Our rationale for this approach is that experience is constituted in participants' accounts as they talk about their surroundings and reactions to them in ways which others can accept and understand. In-depth interviews are a suitable way of gathering and accessing such talk". Setting justified on	The researcher's role and potential bias in the formulation of questions or data collection was not discussed.	Not clear how the study was explained to participants. Respondents gave permission in accordance with agreed ethics protocols, but no further details. No discussion of how confidentiality was maintained or how issues raised in the study were handled by researchers. No reference to ethics committee reported.	A thematic analysis process is described, but it isn't clear whether this applied to all participant groups. "we used a research framework that was built on a critical realist theoretical base, which assumes that realities are socially, culturally and historically situated, but are, nevertheless, experienced as material, objective and stable by participants ...After a period of familiarisation with the transcribed narratives, key themes were identified with reference to topics discussed in the interviews. Indicative narratives identified through this exercise are used to illustrate themes in this paper." No indication of involvement of multiple researchers in the analysis. Sufficient data are presented	The findings of the study were explicit, and discussed in relation to the research question. Limited discussion of the findings in the context of the wider literature. No discussion of the credibility of the findings.	Authors discuss the contribution the study makes to existing knowledge and understanding. The findings are discussed in relation to practice and policy. It is acknowledged that the study focussed on one clinic in one city. Suggestion of conducting further similar studies in other locations to increase generalisability.

Study reference*	Aims & methods	Research design	Sampling†	Data collection	Reflexivity	Ethical issues	Data analysis†	Discussion of findings	Value
				grounds of convenience<br>A list of topics covered by the interviews is included.<br>Data were audio taped and transcribed.<br>Data saturation is not discussed.			support the some of the findings, however the section reporting health practitioner's experiences did not provide supporting quotations for some of the key challenges presented.<br>Contradictory data are not presented<br>No examination of researcher's role, potential bias and influence during the analysis and in presentation of the data.		
Riggs E, Davis E, Gibbs L, Block K, Szwarc J, Casey S, et al. Accessing maternal and child health services in Melbourne, Australia: Reflections from refugee families and service providers. BMC Health Serv Res 2012 01;12(1):117-117 1p.	Aims of the study were clearly stated and its importance and relevance articulated.<br>Qualitative methodology is appropriate to explore perspectives of parents from refugee backgrounds and service providers.	Qualitative research design is justified and the use of focus groups was justified on the grounds that some of the participants favoured this approach.	87 refugee background mothers, 12 nurses, 1 community worker, 1 community liaison, 5 bilingual workers,<br>3 community representatives, 2 managers of bilingual workers<br>The refugees were recruited by convenience sampling at locations where they were known to attend - playgroups, kindergarten, peer education programme, English language organisation. They were invited to participate through a bilingual worker/health worker who was known to them.<br>Researchers took measures to recruit a more representative sample when it became apparent that initial focus groups were not representative.<br>Researchers sought to understand the depth of experiences of refugee background parents had when engaging with MCH services.<br>Healthcare service providers were recruited through purposive sampling. Lacking information about how they were recruited or why they were an appropriate sample.<br>No discussion of reasons of non-participation.	7 Focus groups were used to collect data from refugees and 4 focus groups were used to collect data from service providers. Interviews used with community representatives/manager s of bilingual workers. All focus groups were conducted by ER with assistance from KB or ED The setting of data collection is not described.<br>Use of focus groups and interviews was justified, but setting not described or justified.<br>Focus group guides were used and the areas of questioning were described. Modifications were made to the questions to maximise relevance for different groups.<br>Unclear what methods were used for the interviews<br>Focus groups and interviews were digitally recorded and transcribed, including interpreter translations.<br>Data saturation was not discussed.	The researcher's role and potential bias in the formulation of questions or data collection was not discussed.	Lacking details about how the research was explained to participants. A plain language statement and consent form were provided. No discussion around confidentiality or how issues raised throughout the study were handled by the researchers.<br>Ethical approval was given by the University of Melbourne and the Department of Education and Early Child Development.	Thematic analysis was used.<br>"ER listened to all voice recordings, read and coded all transcripts, and developed categories to organise the data. ED and LG also read a sub-sample of transcripts and coded them. The coding was found to be very similar with any differences discussed by the researchers to arrive at a consensus about final codes. The researchers also discussed patterns, consistencies and contradictions within the data to develop the main themes. ER then refined the themes in consideration of their alignment with the existing literature. All research investigators and the study advisory group came together to discuss the themes, further interpret and explain the results and the implications and applications of the findings."<br>Sufficient data are presented to support the findings, however not all subthemes are supported with direct quotations from participants.<br>contradictory data were taken into account in the analysis.<br>No examination of researcher's role, potential bias and influence during the analysis and in presentation of the data.	The findings of the study were explicit, discussed in relation to the research question and extensively discussed within the context of wider literature.<br>Researchers discussed the credibility of the findings. They discuss triangulation when combining data from the focus groups and interviews, which they suggest can lead to an enhanced description of the phenomenon being explored.<br>Although not explicitly discussed in terms of credibility, the involvement of multiple researchers in coding a sample of transcripts and development of themes enhances the credibility of the findings.	Considered the value of the study and the findings in relation to practice and policy.<br>Suggested areas of further research. In particular, to assess the 'refugee mentor' model described as a potential way to promote access to MCH services.<br>The authors discuss the generalisability of the findings. They comment that as the study was conducted in outer urban areas of Melbourne, the findings may not be applicable to other locations in Victoria (e.g. rural and regional areas).
Samarasinghe K, Fridlund B, Arvidsson B.	Aims of the study were clearly stated	The authors discussed	34 PHCNs. Purposive sampling was used to	Interviews were conducted at the	Authors critically examine the	Research was explained to the	Contextual analysis with reference to phenomenography	The findings are explicit and discussed	The authors discuss the contribution the study

Study reference*	Aims & methods	Research design	Sampling†	Data collection	Reflexivity	Ethical issues	Data analysis†	Discussion of findings	Value
Primary health care nurses' promotion of involuntary migrant families' health. Int Nurs Rev 2010;57(2):224-231.	and its importance and relevance articulated. Qualitative methodology is appropriate for exploring the experiences of primary health care nurses (PHCN) in health promotion with involuntary migrants.	their reasons for using a phenomenographic approach.	select participants for the study, which sought a wide spectrum of participants (sex, age, ethnicity, specialist education, length of primary health care nursing practice).<br>It is stated that each PHCN nurse had worked with approximately 200 involuntary migrant families, indicating that they would have the knowledge required for the studies aims.<br>No discussion about non-participation.	participant's workplace and were all conducted by the first author.<br>Methods not explicitly justified, however researchers explain that they piloted the interview questions beforehand to test the relevance of the questions (these pilots were included in the analysis). No justification given for choice of setting.<br>Areas of enquiry in the interview are described and the interviews lasted approximately 60 min each.<br>No modifications in the methods were necessary.<br>Interviews were tape recorded and transcribed verbatim.<br>Data saturation was not discussed.	influence of the researcher during the interview. "The first author, being a former PHCN herself, may have contributed to a common bond between the participants and the author, making the PHCNs able to freely express their thoughts throughout the interviews, which is crucial in a qualitative study"	participants through verbal and written information, including their right to withdraw from the study at any time.<br>Participants gave written consent and were assured of confidentiality (data being unidentifiable)<br>Lacking details on how researchers handled issues raised for participants by the study.<br>The study was approved by the university ethics committee of Sweden.	was used.<br>“The first author with experience of working within PHC carried out the analysis, while the two co-authors with specialized knowledge of the methodology served as additional evaluators in the categorization procedure.…The analysis was carried out in six steps: (1) the transcribed interviews were read several times to obtain a sense of the whole; (2) the interviews were processed, and descriptive statements relating to the aim of the study were identified, delimited, analysed and structured into an overview of concepts and keywords; (3) a comparative reduction of the data was commenced by giving a summarized description of each interview from this overview; (4) the summarized descriptions were differentiated by comparisons in relation to similarities and differences of the summarized descriptions, and were grouped together in three qualitatively distinct groups; (5) the underlying structure of the grouped descriptions was identified and described by going back and forth between the grouped descriptions and the original interviews;<br>(6) and the transcribed interviews of the 34 participant PHCNs were finally allocated to the three qualitatively distinct groups of these descriptions.”<br>Sufficient data are presented to support the findings.<br>No examination of researcher’s role, potential bias and influence during the analysis and in presentation of the data.	in relation to the original research question.<br>Findings are discussed in relation to the wider literature.<br>Authors discuss the process of re-evaluating data in validating the descriptive categories, including the choice of quotations.<br>The analysis was conducted by one person (lead author), but was evaluated by two other co-authors.	makes to informing clinical practice and policy.<br>Recommendations are given to improve the training of nurses, to equip them to work with involuntary migrant families.<br>Further research is suggested to determine how to facilitate cultural transition for involuntary migrants.<br>Transferability of the findings is discussed.
Suurmond J, Rupp I, Seeleman C, Goosen S, Stronks K. The first contacts between healthcare providers and newly-arrived asylum seekers: A qualitative study about which issues need to be addressed. Public Health Jul	Aims of the study were clearly stated and its importance and relevance articulated. Qualitative methodology is appropriate for investigating the issues that healthcare providers	Use of qualitative design not explicitly justified.	36 nurse practitioners and 10 public health physicians. Participants were a purposive sample of nurse practitioners and public health physicians from different asylum seeker centres (from across the Netherlands). They were approached by the coordinator to ascertain if	7 group interviews were used to collect data and were conducted by two specified researchers (IR and CS). The setting was not described.<br>No justification given for methods or setting of data collection.<br>A topic list was used, which had been	The researcher's role and potential bias in the formulation of questions or data collection was not discussed.	Written information about the study was given to all participants who were assured of confidentiality and anonymity (Anonymity was assured by the use of codes).	Data was analysed by Framework approach.<br>Interviews were analysed, starting with the familiarization stage. “Short notes were made to identify themes. This resulted in a thematic framework. The framework was systematically applied to the material, and all interviews were reread and annotated accordingly. Charts	The findings are explicit and discussed in relation to the original research question.<br>There is adequate discussion of the findings in context of the wider literature.<br>No discussion of the credibility of the	The authors discuss the findings in relation to practice and policy, providing perspectives and models that can inform service provision for this group.<br>Authors sought to provide a generic model (beyond first contact) for healthcare provision

Study reference*	Aims & methods	Research design	Sampling†	Data collection	Reflexivity	Ethical issues	Data analysis†	Discussion of findings	Value
2013;127(7):668-673.	need to address in their first contact with asylum seekers.		they were willing to participate in a group interview.<br>The role of these health professionals in caring for asylum seekers was described, giving justification for their selection.<br>No discussion around non-participation.	developed from the results a survey that had previously been sent to a sample of nurse practitioners and public health physicians. Areas of enquiry are described.<br>All interviews were recorded on tape and transcribed.<br>Data saturation was not discussed.		Informed consent was tape-recorded a priori the interviews.<br>Lacking details on how researchers handled issues raised for participants by the study.<br>The employer of the nurse practitioners and public health physicians (Community Health Services for Asylum Seekers) approved the study. Medical-ethical approval of this study was not required, according to the Dutch Medical Research Involving Human Subjects Act as it only involved care providers and it was not an intervention study.	were devised with headings (and sometimes subheadings) for each key theme. Each chart contained entries for several respondents. Finally, these charts were used to describe patterns through an iterative, comparative process of searching, reviewing, and comparing the data."<br>Sufficient data are presented to support the findings." No indication of involvement of multiple researchers in the analysis.<br>Contradictory data were not presented.<br>No examination of researcher's role, potential bias and influence during the analysis and in presentation of the data.	findings.	for asylum seekers, to increase generalisability to other settings.
Suurmond J, Seeleman C, Rupp I, Goosen S, Stronks K. Cultural competence among nurse practitioners working with asylum seekers. Nurse Educ Today 2010 11;30(8):821-826 6p.	Aims of the study were clearly stated and its importance and relevance articulated.<br>Qualitative methodology is appropriate to explore the views of nurse practitioners about cultural competencies that are important for working with asylum seekers.	Qualitative methodology not explicitly justified.	89 nurse practitioners completed questionnaires. 36 nurse practitioners participated in group interviews. Not reported whether there was overlap in these two data sources.<br>Participants in the questionnaire were a convenience sample. Those who returned the questionnaire were included. It is not known how many questionnaires were distributed, so a response rate cannot be given.<br>Participants for the group interviews were a purposive sample, selected by local coordinators in order to increase representation from different asylum seeker centres and maximise variation in experiences.	89 questionnaires and 7 group interviews were used to collect data., which were conducted by 2 named researchers.<br>The setting for data collection is not clearly described<br>The combination of questionnaires and group interviews (triangulation) was put forward as a way of increasing credibility.<br>No justification of setting<br>No discussion of how the questionnaires were developed. A topic guide was used for group interviews; however, the questions were not focussed on this particular research question. Data about cultural competence emerged in the course of the discussions.	The researcher's role and potential bias in the formulation of questions or data collection was not discussed.	Information about the study was given in the form of a flyer as well as in a letter accompanying the questionnaire.<br>Lacking details about how the research was explained to participants in group interviews.<br>Consent was gained from all participants and they were assured of confidentiality.<br>Lacking details on how researchers handled issues raised for participants by the study.<br>According to the Medical Research	Framework approach was used to analyse the data. "After familiarisation with the data, a coding framework was identified. The questionnaires were then systematically coded using this framework. Data were subsequently charted and three major charts were constructed: educational background, important cultural competences in connection with asylum seekers, and ideas about how cultural competences may be improved. The transcription of each group interview was read carefully to gain an overall impression before being coded and analysed. One chart was designed on the basis of different cultural competences that were mentioned in the interviews. Using this chart, patterns and connections could be described." Not clear how the two sources of data were	The findings are explicit and discussed in relation to the original research question.<br>There is adequate discussion of the findings in context of the wider literature.<br>Authors suggest that credibility is enhanced by having two data sources (questionnaires and group interviews), which allows triangulation.	The contribution to existing knowledge and understanding is discussed. The authors state that the results of the study can be used for training and education of health care professionals. They believe that the results are relevant to other care providers who work with asylum seekers (generalisable)<br>Further areas of research are not discussed.

Study reference*	Aims & methods	Research design	Sampling†	Data collection	Reflexivity	Ethical issues	Data analysis†	Discussion of findings	Value
			The role of these health professionals in caring for asylum seekers was described, giving justification for their selection.<br>No details given about reasons for non-participation.	Group interviews were recorded on tape and transcribed.<br>Data saturation was not discussed.		Involving Human Subjects Act, medical–ethical approval of this study was not required in the Netherlands (only care providers involved and not an intervention study). approval was obtained from the Community Health Services for Asylum Seekers, the employer of the nurse practitioners.	synthesised. No indication of involvement of multiple researchers in the analysis.<br>Sufficient data are presented to support the findings, however only 2 direct quotations are used in the entire findings section, which had 9 headings.<br>Contradictory data were not presented.<br>No examination of researcher's role, potential bias and influence during the analysis and in presentation of the data.		
Tellep TL, Chim M, Murphy S, Cureton VY. Great suffering, great compassion: A transcultural opportunity for school nurses caring for Cambodian refugee children. Journal of Transcultural Nursing Oct 2001;12(4):261-274.	Aims of the study were clearly stated and its importance and relevance articulated.<br>Qualitative methodology is appropriate to explore experiences of school nurses and Cambodian liaisons that provide care for refugee families.	Qualitative methodology not explicitly justified, although it is mentioned that the focus groups were conducted to gain insight into the concepts of transcultural and intracultural reciprocity as experienced by school nurses in their relationships with Cambodian refugees.	6 school nurses, 2 Cambodian liaisons.<br>"A purposive sample of school nurses and Cambodian liaisons was recruited from a school district serving a large population of Cambodian children in California. Six of the district's eight nurses volunteered as well as two of the three Cambodian liaisons."<br>Invitation was through a phone call or letter.<br>No reasons given for why these participants were chosen, although it is clear that the nurses had a high level of experience working with Cambodian refugees (6-15 years' experience).<br>No discussion of the reasons for non-participation of those approached, that did not volunteer.	Focus group with Cambodian liaisons was held in the home of a non-Cambodian school nurse. Focus group with school nurses was held in their school district conference room. Focus groups were moderated by two of the authors.<br>No justification for the methods Setting of the groups with Cambodians was justified based on wanting to provide a friendly atmosphere and authors explain the cultural reasons for tea/coffee and relational time before the interviews. No justification of the setting of nurse interviews.<br>A semi-structured interview guide was used in the focus group. Broad areas of enquiry are described, but specific questions not stated.<br>Data were tape recorded and transcribed verbatim. field notes were reviewed.<br>Data saturation was not discussed.	The researchers critically examined their roles and potential bias in the data collection.<br>"Through directing the research to look for insights into the concepts of transcultural and intracultural reciprocity, the authors may not have been as open to other concepts arising from the data regarding the nature of the participants' interactions with Cambodian refugee families. In retrospect, serving Cambodian refreshments at the school nurse focus group relayed the school nurse moderator's bias of transcultural interest and empathy toward Cambodians. This bias may have limited the types of information and viewpoints shared by the nurses. In addition, they may have been hesitant to share issues in the presence of the	Lacking details about how the research was explained to participants.<br>Consent was gained before conducting the focus groups and issues of confidentiality were considered.<br>Lacking details on how researchers handled issues raised for participants by the study.<br>"San Jose State University's Human Subjects Institutional Review Board approved the study's research protocol."	Limited details about the analysis methodology or process. "Tapes were listened to and transcripts were reviewed several times by the moderators individually and together. The data were grouped and categorized into emergent issues and themes and also reviewed in light of Dobson's (1989) conceptual framework of transcultural health visiting." No indication of involvement of multiple researchers in the analysis.<br>Sufficient data were presented to support the findings.<br>Contradictory data were not presented.<br>No examination of researcher's role, potential bias and influence during the analysis and in presentation of the data.	The findings are explicit and discussed in relation to the original research question.<br>Little discussion of the findings in the context of the wider literature.<br>As already noted in the reflexivity section, authors acknowledge potential bias in the interview process, which could impact the credibility of the findings.	Authors provide a number of recommendations for nursing practice<br>Areas for further research are suggested.<br>Authors discuss generalisation of findings, pointing out that focus group research results are not meant to be generalised. They refer readers to Kruger's concept of transferability when reflecting on using these findings in other settings.

Study reference*	Aims & methods	Research design	Sampling†	Data collection	Reflexivity	Ethical issues	Data analysis†	Discussion of findings	Value
					Cambodian nurse assistant moderator. A similar inhibitor may have existed in the presence of the non-Cambodian school nurse assistant moderator with the Cambodian liaison focus group.				
Tobin C.L., Murphy-Lawless J. Irish midwives' experiences of providing maternity care to non-Irish women seeking asylum. International Journal of Women's Health 2014 31 Jan 2014;6(1):159-169.	Aims of the study were clearly stated and its importance and relevance articulated. Qualitative methods are appropriate to explore midwives' perceptions of caring for women in the asylum process and gain insights into how they can be equipped to provide effective care to this group.	Use of qualitative methodology not explicitly justified.	10 midwives. The participants were purposively selected to ensure that they had experience providing care to asylum seekers. They were chosen from two different sites (an urban hospital and a rural hospital) to gain a wider variety of experiences. Information packs describing the study were distributed by researchers to the two sites; the researchers then followed up with visits to the sites to hold information sessions about the study and to answer questions. Demographic information about participants is included, demonstrating the appropriateness of the sample. No discussion around non-participation.	Data were collected by in-depth, unstructured interviews at a place convenient for participants (usually at home of at a private office space). Interviewer not reported. No justification given for methods or setting of data collection Interviews were launched with one open-ended question and ranged from 26-70 minutes. Data were audio recorded and later transcribed verbatim. Data saturation was not discussed.	Extensive field notes and reflective journals were kept, that provide an audit trail of decision-making and an aid for the qualitative researcher to deepen awareness of their own bias, reactions, and emotions to the data as they emerge. Clinical and peer supervision was used throughout the data collection process.	Information packs describing the study were distributed by researchers to the two sites; the researchers then followed up with visits to the sites to hold information sessions about the study and to answer questions. Informed consent, voluntary participation, and assurance of confidentiality were made explicit. Lacking details on how researchers handled issues raised for participants by the study. Article states that ethical approval was gained from relevant institutions, but details not provided.	Data were analysed using content analysis. "The analysis was undertaken by hand, and involved several readings of transcripts, followed by coding of data and grouping coded material based on shared content or concepts to identify common themes. Transcripts were also read in their entirety by a second researcher to confirm the themes that were identified and add to the validity of the findings." Sufficient data is presented to support the findings. Contradictory data are considered No examination of researcher's role, potential bias and influence during the analysis and in presentation of the data.	The findings are explicit and discussed in relation to the original research question. There is adequate discussion of the findings in context of the wider literature. No explicit discussion of the credibility of the findings.	The contribution of the study to existing knowledge and understanding was discussed. highlights the continued difficulties midwives experience in achieving effective communication, understanding difference, and coping with the emotional cost of caring within a hospital-based technological model of maternity care. Recommends ways that service delivery to asylum seekers could be improved. New areas for research are identified. Authors acknowledge that the study is small scale, and cannot be generalised to the whole population.
Twohig PL, Burge F, MacLachlan R. Pod people. Response of family physicians and family practice nurses to Kosovar refugees in Greenwood, NS. Canadian Family Physician 2000 Nov;46:2220-2225.	Aims of the study were clearly stated and its importance and relevance articulated. Qualitative methodology is appropriate for exploring the experiences of family practice nurses and family physicians that cared for refugees in a refugee	Use of qualitative methodology not explicitly justified.	6 family practice nurses, 10 family physicians. Participants were purposively sampled from the service roster to enrol different kinds of family practice nurses and family physicians. Lacking details about how participants were invited to participate. All the participants had worked at the centre that was the focus of the study. No discussion about non-	Data were collected through semi-structured interviews at a 'private setting' (no further details) and were conducted by one team member (PT). No justification given for methods, but does justify setting as a private place to allow the participants to freely and openly share experiences. Lacking explicit details about the methods (no	The researcher's role and potential bias in the formulation of questions or data collection was not discussed.	Lacking details about how the research was explained to participants. Written consents were obtained, but lacked details about confidentiality and how issues raise for participants in the study were handled by researchers.	A form of textual analysis was applied. "For each interview, key words or phrases were identified and compared with subsequent inter views until no significant new ideas emerged. Once researchers were satisfied that saturation had been achieved, words and phrases were grouped into larger conceptual categories. A second researcher reviewed a subset of transcripts and critiqued and confirmed the preliminary categories. This	The findings are explicit and discussed in relation to the original research question. Lacking discussion of the findings in the context of the wider literature. No explicit discussion of the credibility of the findings although researchers report that a second researcher critiqued categories	The contribution made by the study to existing knowledge and understanding is discussed. Teamwork in emergency response is suggested as a possible avenue for further research. Authors acknowledge that the findings of the study cannot be generalised to other relief settings, but

Study reference*	Aims & methods	Research design	Sampling†	Data collection	Reflexivity	Ethical issues	Data analysis†	Discussion of findings	Value
	processing centre.		participation.	description of interview guide, format, areas of enquiry) Data were audiotaped and transcribed verbatim. Data saturation was discussed in the analysis process. Comparisons of key words and phrases were made across interviews until no new themes emerged.		Ethics approval was obtained from Dalhousie's Faculty of Medicine.	process was repeated until the categories were clear. These categories became the basis for a coding structure within QSR NUD*IST, software designed for textual analysis." Sufficient data were presented to support the findings. Contradictory data were taken into account. No examination of researcher's role, potential bias and influence during the analysis and in presentation of the data.	that emerged in the analysis	suggest that they could offer insights to generate other research questions.
Yelland J, Riggs E, Wahidi S, Fouladi F, Casey S, Szwarc J, et al. How do Australian maternity and early childhood health services identify and respond to the settlement experience and social context of refugee background families?. BMC Pregnancy & Childbirth 2014;14:348.	The aims of the study were clearly stated and its importance and relevance articulated. Qualitative methodology is appropriate to explore experiences of Afghan parents accessing maternity services and health professional's views on/experiences of identification of refugee background.	Authors state that the methods were informed by community and service provider consultations.	30 Afghan parents, 10 midwives, 5 medical practitioners, 19 Community based health professionals. Afghan men/women: "Purposive recruitment methods and multiple initial contacts were used to optimise recruitment and ensure diversity of potential participants." Inclusion criteria was women and men born in Afghanistan ≥ 18 years old and had a baby that was around 4-12 months old. Also an element of convenience sample. "A postcard with information about the study and details about how to take part, in Dari and English, was distributed to local groups and services, and the postcard was printed in the Afghan community newspaper. Potential participants were provided with a telephone number to contact the community researchers to register their interest in participating in an interview." No discussion around non-participation. Health professionals: Purposive sample. Key informants invited to participate after identification by researchers, with further participants identified through initial participants. All provided care for	Afghan parents: Semi-structured interviews were conducted by community researchers. Setting of data collection was described. Participants were given a choice of location and language preference for interview No justification given for methods or setting of data collection. Interview schedule was designed based in information from a previous community consultation. Areas on enquiry are described. Authors report that the interview schedule was modified after piloting with 6 participants. Interviews were recorded on audio tape. Those conducted in Afghan language (80%) were translated into English and transcribed by community researchers. Data saturation not discussed. Health professionals: A mixture of focus groups and interviews were used with the majority being conducted by one author (ER); one was conducted by another author (JY). The setting of interviews/focus groups not reported. No justification given for methods or setting of	The researcher's role and potential bias in the formulation of questions or data collection was not discussed.	Afghan parents: Potential participants were provided with verbal information and given a copy of the study information in Dari or English and were asked to consent in writing or verbally. Confidentiality, or how issues raised in the study for participants were handled by researchers, are not discussed. Health professionals: Lacking detail about how the research was explained to these participants. No discussion around consent, confidentiality or how issues raised through the study were handled by researchers. "The project was approved by the research ethics committees of the Victorian Foundation for Survivors of Torture and the Royal Children's Hospital."	A thematic analysis approach was taken and the analysis process is described for analysing qualitative data from Afghan parents and health professionals. Afghans: "Analysis began after the first three interviews with women which were coded, informing the coding manual. A coding manual was developed using some a priori codes from the interview schedule; an iterative process was used to add additional codes to the manual (undertaken by ER, JY, FF,SW). This coding manual was used to code all women and men's interviews. JY and ER cross-checked the coding of all interview transcripts, providing an opportunity to discuss differences in the interpretation of the data. Codes were then grouped into logical categories which then provided the overarching themes." Health professionals: "All transcripts were read (by ER, JY) and imported and stored in NVivo10 [26]. Coding and categorising of data was undertaken (by ER), and key themes identified." Authors state that the paper does not report all the themes. Quotations were selected to illustrate the themes identified in the analysis. Sufficient data were presented to support the findings. Contradictory data were taken into account. No examination of researcher's role, potential bias and influence	The findings are explicit and discussed in relation to the original research question. Authors discuss the findings in relation to the wider research literature. Authors discuss the strength of having two components of the study - afghan community and health professionals. Thematic analysis of afghan participant data involved multiple analysts. Analysis of health professional data was primarily completed by one author.	Authors discuss the contribution of the study to existing knowledge and understanding. Further areas for research are not discussed. The transferability of the results is discussed. The authors acknowledge that this study included one community group in one region of Melbourne, so may not be generalisable to other groups. However, authors suggest that the stories told here may resonate with other groups in other settings.

Study reference*	Aims & methods	Research design	Sampling†	Data collection	Reflexivity	Ethical issues	Data analysis†	Discussion of findings	Value
			families of refugee background.<br>No discussion around non-participation.	data collection.<br>An interview schedule was used with areas of enquiry described.<br>Interviews were digitally recorded and transcribed by an outside agency.<br>Data saturation was not discussed.			during the analysis and in presentation of the data.		
Yelland J, Riggs E, Szwarc J, Casey S, Duell-Piening P, Chesters D, et al. Compromised communication: a qualitative study exploring Afghan families and health professionals' experience of interpreting support in Australian maternity care. BMJ Qual Saf 2016 Apr;25(4):e1-2014-003837. Epub 2015 Jun 18	Aims of the study were clearly stated and its importance and relevance articulated.<br>Qualitative methodology is appropriate to explore experiences of Afghan parents and health professionals.	Research methodology not explicitly justified, but authors state that the data collection methods were informed by consultation with Afghan community members and health professionals working in the area.	Afghan women and men:<br>Potential participants were identified through consultation with community groups, community leaders and the project's advisory group.<br>Not clear how individuals were approached.<br>Inclusion criteria was women and men born in Afghanistan ≥ 18 years old and had a baby that was around 4-12 months old.<br>No discussion around non-participation<br>Heath professionals:<br>Mixed purposive/convenience sample. The research was promoted within organisations that health professionals worked. Those interested in participating responding. Others personally recommended by key stakeholders.<br>Participants were eligible if they had provided services to Afghan families.<br>No discussion around non-participation.	Afghan participants:<br>Interviews were used to collect data and were conducted by Afghan background researchers (one woman, one man).<br>The setting of data collection was not described.<br>No justification given for methods or setting of data collection.<br>An interview schedule that had been developed with input from a previous population-based survey and was translated into Dari and piloted with 6 community members. Areas of enquiry are described.<br>Interviews were audio-taped and transcribed into English.<br>Data saturation was not discussed.<br>Health professionals:<br>Focus groups and interviews were used, but the setting or the interviewer(s) for data collection are not described.<br>An interview schedule was used and areas of enquiry are described.<br>No justification given for methods or setting of data collection.<br>Interviews were digitally recorded and transcribed by an external agency.<br>Data saturation was not discussed.	No explicit critical examination of the researcher's role and potential bias in formulating the research question or data collection. The Authors employed a participatory approach, which enhanced their capability to engage with the community (involved community members in recruitment and conducting interviews)	Afghan participants:<br>Lacking details on how the research was explained to potential participants.<br>Permission was given for audio-recording, but unclear whether consent was given for participation in the study.<br>No discussion of how confidentiality was maintained or how issues raised through the study were handled by researchers.<br>Health professionals:<br>Lacking details about how the research was explained to potential participants.<br>No details given about how participants consented, how confidentiality was maintained or how issues raised through the study were handled by researchers.<br>"The project was approved by the research ethics committees of the Victorian Foundation for the Survivors of Torture and The Royal Children's Hospital."	Afghan participants:<br>A thematic approach was taken. "All transcripts were coded manually by the community researchers and cross-checked (by FF, SW, ER, JY) and entered into NVivo10. Based on the completed coding of the first four transcripts (two women and two men) a coding manual was developed and used to code remaining transcripts. Discussion among the research team was done to place all codes into logical categories. From this seven, major themes were identified and the theme of 'language services and communication' is reported in this paper"<br>Health professionals:<br>Thematic approach was taken. "JY read all of the transcripts. The data were analysed thematically. All transcripts were coded using NVivo software (by ER) into practical categories and overarching themes."<br>Authors discuss how data were selected for this publication and that it does not represent all the themes, which were published elsewhere.<br>Sufficient data are presented to support the findings.<br>Contradictory data are taken into account<br>No examination of researcher's role, potential bias and influence during the analysis and in presentation of the data.	The findings are explicit and discussed in relation to the research question<br>The findings are discussed in the context of the wider literature.<br>Lacking explicit discussion of the credibility of the findings, however authors discuss the merits of using a participatory approach to engage refugees and it is apparent that more than one analyst was involved in defining themes from data from Afghan participants.	Authors discuss the contribution of the study to existing knowledge and understanding.<br>Further areas for research are not discussed.<br>The transferability of the results is discussed. The authors acknowledge that this study included one community group in one region of Melbourne, so may not be generalisable to other groups. However, authors suggest that the stories told here may resonate with other groups in other settings.

Study reference*	Aims & methods	Research design	Sampling†	Data collection	Reflexivity	Ethical issues	Data analysis†	Discussion of findings	Value
* Direct quotations from articles in this table are presented within quotation marks.<br>† Sampling and analysis methods are as reported by the authors.									
