# Supplementary material for: Challenges and facilitators for health professionals providing primary healthcare for refugees and asylum seekers in high-income countries: a systematic review and thematic synthesis of qualitative research
Source: BMJ Open. 2017 Aug 4;7(8):e015981. doi: 10.1136/bmjopen-2017-015981 (PMC5629684; doi:10.1136/bmjopen-2017-015981)
Supplement: Author's manuscript [file bmjopen-2017-015981.draft_revisions.pdf]

BMJ Open

Challenges and Facilitators for Health Professionals Providing Primary Healthcare for Refugees and Asylum Seekers in High-Income Countries: A Systematic Review and Thematic Synthesis of Qualitative Research

Journal:	*BMJ Open*
Manuscript ID	bmjopen-2017-015981
Article Type:	Research
Date Submitted by the Author:	19-Jan-2017
Complete List of Authors:	Robertshaw, Luke; University of Birmingham, Public Health, Epidemiology & Biostatistics<br>Dhesi, Surindar; University of Birmingham, School of Geography, Earth and Environmental Sciences<br>Jones, Laura; University of Birmingham, Public Health, Epidemiology & Biostatistics
**Primary Subject Heading**:	Public health
Secondary Subject Heading:	Qualitative research, Health services research, Health policy, Global health, General practice / Family practice
Keywords:	Asylum seeker, Refugee, Primary healthcare, PRIMARY CARE

SCHOLARONE™ Manuscripts

For peer review only

Challenges and Facilitators for Health Professionals Providing Primary Healthcare for Refugees and Asylum Seekers in High-Income Countries: A Systematic Review and Thematic Synthesis of Qualitative Research

Corresponding Author:

Dr Laura L Jones, Institute for Applied Health Research, University of Birmingham, Birmingham, UK

l.l.jones@bham.ac.uk

+44 (0)121 414 3024

First Author:

Luke Robertshaw, Institute for Applied Health Research, University of Birmingham, Birmingham, UK

Second Author:

Surindar Dhesi, School of Geography, Earth and Environmental Sciences, University of Birmingham, Birmingham, UK

Third Author:

Dr Laura L Jones, Institute for Applied Health Research, University of Birmingham, Birmingham, UK

Word count: Abstract 234, Main article: 6246

For peer review only

For peer review only - http://bmjopen.bmj.com/site/about/guidelines.xhtml

Abstract

Objectives: To thematically synthesise primary qualitative studies that explore challenges and facilitators for health professionals providing primary healthcare for refugees and asylum seekers in high-income countries.

Design: Systematic review and qualitative thematic synthesis.

Methods: Searches of MEDLINE, EMBASE, PsycINFO, CINAHL and Web of Science. Search terms were combined for qualitative research, primary healthcare professionals, refugees and asylum seekers, and were supplemented by searches of reference lists and citations. Study selection was conducted by two researchers using pre-specified selection criteria. Data extraction and quality assessment using the CASP tool was conducted by the first author. A thematic synthesis was undertaken to develop descriptive themes and analytical constructs.

Results: Twenty-six articles reporting on 21 studies and involving 357 participants were included. Eleven descriptive themes were interpreted, embedded within three analytical constructs: Healthcare encounter (trusting relationship, communication, cultural understanding, health and social conditions, time); Healthcare system (training and guidance, professional support, connecting with other services, organisation, resources and capacity); Asylum and resettlement. Challenges and facilitators were described within these themes.

Conclusions: A range of challenges and facilitators have been identified for health professionals providing primary healthcare for refugees and asylum seekers that are experienced in the dimensions of the healthcare encounter, the healthcare system and wider asylum and resettlement situation. Comprehensive understanding of these challenges and facilitators is important to shape policy, improve the quality of services and provide more equitable health services for this vulnerable group.

For peer review only - http://bmjopen.bmj.com/site/about/guidelines.xhtml

Strengths and limitations of this study

- This is the first review to systematically identify and synthesise qualitative research exploring challenges and facilitators for health professionals providing primary healthcare for refugees and asylum seekers.
- Thematic synthesis of studies from a range of countries and primary healthcare settings allows identification of common, generalisable themes with potential to influence policy and practice.
- The review was limited to English language studies, which may have led to over-representation of studies conducted in English-speaking high-income countries.
- The review was limited to core, clinical health professionals: doctors nurses and midwives.

Background and introduction

Throughout human history, countless people have been forced to flee from their homes and countries due to violence or threats of violence. Other nations may provide refuge for those seeking a safe haven, and In 1950, the Office of the United Nations High Commissioner for Refugees (UNHCR) was established to provide international leadership and coordination for the protection of refugees and promotion of their wellbeing.[1] The UNHCR convention defines refugees as persons who have a “well-founded fear of being persecuted for reasons of race, religion, nationality, membership of a particular social group or political opinion, is outside the country of his nationality and is unable or, owing to such fear, is unwilling to avail himself of the protection of that country; or who, not having a nationality and being outside the country of his former habitual residence as a result of such events, is unable or, owing to such fear, is unwilling to return to it.”[2] Those in the application process to be granted refugee status are referred to as ‘asylum seekers’. By the end of 2015 there were an estimated 65.3 million forcibly displaced people worldwide, including

For peer review only - http://bmjopen.bmj.com/site/about/guidelines.xhtml

40.8 million internally displaced people, 21.3 million refugees and 3.2 million asylum seekers.[3]

Refugees and asylum seekers are a vulnerable group with significant and complex health needs.[4] A survey by the UK Border Agency in 2010 showed refugees to be in poorer health than the general population.[5] As most refugees and asylum seekers originate from low-mid income countries, there are, accordingly, higher prevalence's of pre-existing infectious diseases such as Hepatitis B, TB and HIV compared to host populations.[6] The risk of contracting infectious diseases may be further exacerbated by poor hygiene conditions during flight from conflict, coupled with insufficient vaccine coverage.[7] Studies have also highlighted the sexual and reproductive health needs of this group,[8] with high levels of sexual gender based violence (SGBV) being reported along with limited access to contraception.[8, 9]

A further concern for refugee and asylum seeker populations is their mental health. Violence experienced in countries of origin, including war, sexual abuse and torture are reported, that may lead to psychological and physical trauma.[10] These pre-migration traumas are compounded by post-migration stressors such as loss of social networks, shifting societal roles and cross-cultural stress while integrating into countries of settlement.[11] Fazel et al [12] estimated that 9% of adult refugees may suffer with post-traumatic stress disorder (PTSD), which is approximately ten times estimates in an age-matched American population.[12]

Considering the complex health and social needs presented by refugees and asylum seekers, significant challenges are faced by healthcare providers [13-15] that may contribute to recognised healthcare inequalities, where refugees and asylum seekers experience lower quality of care compared to other service users.[16] Primary healthcare teams are at the front-line of such healthcare provision in high-income countries.[17] These teams may include members from a variety of professional backgrounds, clinical and non-clinical, but typically include a core of general practitioners, community based nurses and midwives.[18, 19] Experiences of health professionals caring for refugees and

For peer review only - http://bmjopen.bmj.com/site/about/guidelines.xhtml

asylum seekers in high-income countries have been investigated through a range of qualitative research studies conducted across several countries and primary healthcare settings. A recent systematic review by Suphanchaimat et al [20] synthesised challenges providing healthcare services to migrants from a provider perspective. The review included a minority of studies that had refugees and asylum seekers as service users, focussed purely on challenges of healthcare provision, and adopted a limited, purposive search strategy. To our knowledge, this present review is the first to synthesise experiences of health provision for migrants defined specifically as refugees and asylum seekers; synthesise both challenges and facilitators for health professionals; and adopt a systematic approach to identification of qualitative research. Therefore, this review aims to systematically identify and thematically synthesise challenges and facilitators experienced by health professionals that provide primary healthcare for refugees and asylum seekers in high-income countries.

For peer review only

For peer review only - http://bmjopen.bmj.com/site/about/guidelines.xhtml

Methods

This systematic review sought qualitative research studies as they are the appropriate design for understanding perceptions and experiences of healthcare provision. [21, 22] Systematic identification and synthesis of these studies may consolidate the current evidence-base, increase the breadth and depth of understanding and provide more generalisable conclusions than individual primary studies.[23, 24]

This review was guided by established methodology for systematic review and thematic synthesis of qualitative research, outlined by Thomas and Harden.[25] Thematic synthesis of data, applied in this methodology, is suited to development of recommendations for practice and policy and provides a transparent link between conclusions and the primary studies synthesised.[25, 26] Reporting of this review has been guided by Enhancing Transparency of Reporting the Synthesis of Qualitative Research (ENTREQ) framework.[27]

Search strategy

The following databases were searched from inception until week 3 of March 2016: MEDLINE, EMBASE, PsycINFO, CINAHL and Web of Science. The search strategy was based on the SPIDER (Sample, Phenomenon of interest, Design, Evaluation, Research type) tool.[28] Search terms were combined for primary health professionals/heathcare, refugees and asylum seekers, and qualitative research. No language or date limits were applied. The full detailed search strategy is documented in online supplement 1. Further hand-searches were conducted based on included studies' reference lists and citations (in Google Scholar).

After removal of duplicates, titles and abstracts were screened by one researcher, excluding articles that clearly did not meet the inclusion criteria. Full-texts of remaining articles were obtained and assessed by two independent researchers, according to pre-specified study selection criteria (detailed below). Disagreements were resolved via discussion.

For peer review only - http://bmjopen.bmj.com/site/about/guidelines.xhtml

Selection criteria

This review included peer reviewed, qualitative primary research studies that met the following criteria: English language; conducted in a high-income country as defined by the World Bank country classification 2015;[29] explored challenges or facilitators (defined in Box 1) for health professionals providing primary healthcare to refugees and asylum seekers (including forced migrants, involuntary migrants or refugee claimants).

> **Box 1:** Definitions of challenge and facilitator
>
> Challenge: A factor that inhibits, obstructs or creates difficulties for health professionals when providing primary healthcare.
>
> Facilitator: A factor that promotes, enables or assists health professionals when providing primary healthcare

Mixed-methods studies were included if the qualitative element's methods and results could be isolated for synthesis. As definitions of health professionals in primary healthcare teams are diverse, [19] this review was limited to articles that interviewed core clinical healthcare professionals including: general practitioners, nurses, pharmacists and midwives working in primary healthcare settings. Articles were excluded if: they were not based on peer reviewed primary qualitative studies (i.e. reviews, case studies, reports, opinion pieces); were conducted in a secondary care setting; or if the service users were described as illegal immigrants, undocumented migrants, migrants or immigrants. Articles interviewing mental health professionals were excluded as this clinical area has specific characteristics. Where studies contained a mixture of eligible and ineligible participants, they were only included if data for eligible participants could be isolated for synthesis. Studies were also excluded if the full text articles could not be obtained through institutional access or requests sent to authors through Research Gate. The full inclusion and exclusion criteria applied in this review are documented in online supplement 2.

For peer review only - http://bmjopen.bmj.com/site/about/guidelines.xhtml

Data extraction

Study characteristics were extracted by one author using a data extraction proforma. Characteristics included aims, setting, participants, methodology, results and recommendations/applications. Findings (results) and discussion sections from included studies were imported into NVivo 11 software (NVivo qualitative data analysis Software; QSR International Pty Ltd. Version 11, 2016) for analysis.

Assessment of quality

Included studies were assessed by one author using the Critical Appraisal Skills Programme (CASP) tool for appraisal of qualitative research.[30] Studies were not excluded from the synthesis or given weighting based on this assessment as there is currently no accepted method for this in syntheses of qualitative research.[31] All studies were included irrespective of their reporting quality given that they contributed to the conceptual richness of the synthesis. Where studies used mixed-methods, only the qualitative element was appraised.

Data synthesis

A thematic synthesis was conducted broadly following the methodology outlined by Thomas and Harden.[25] An article, considered data-rich (containing numerous challenges and facilitators), was selected as an index-article and uploaded into NVivo 11 software. The findings (results) and discussion sections were coded inductively within an a priori framework of challenges and facilitators. Primary quotations, author's commentary and author's interpretations were coded. Sections were only coded if they contained challenges or facilitators (Box 1), and referred to the health professionals defined for this review. Following the index-article, subsequent articles were coded using the same method in approximate order of descending data-richness. Concepts in each article were coded into existing concepts, with new codes being added as deemed appropriate to develop a codebook. The final codebook was analysed to inform descriptive themes closely resembling the prevailing concepts across primary studies. These themes were discussed and agreed within the research team. An analytical model was then developed to create higher-order constructs within which descriptive themes were located.

For peer review only - http://bmjopen.bmj.com/site/about/guidelines.xhtml

Results

Systematic search and selection

Systematic database searches identified 5970 articles. A further 16 articles were identified through hand-searching of reference lists and citations. After removal of duplicates, 3571 articles remained. 3493 articles were excluded based on the title and abstract. Full-texts of the remaining 78 articles were sought for detailed assessment against the inclusion criteria. Nine of these articles could not be obtained. In addition, due to resource limitations, four non-English language studies were unable to be translated and assessed against the selection criteria. After reviewing the 65 available full-text papers and applying the full selection criteria, 26 articles were included in the thematic synthesis (Figure 1).

For peer review only

For peer review only - http://bmjopen.bmj.com/site/about/guidelines.xhtml

Figure 1 Flow diagram of systematic search and study selection

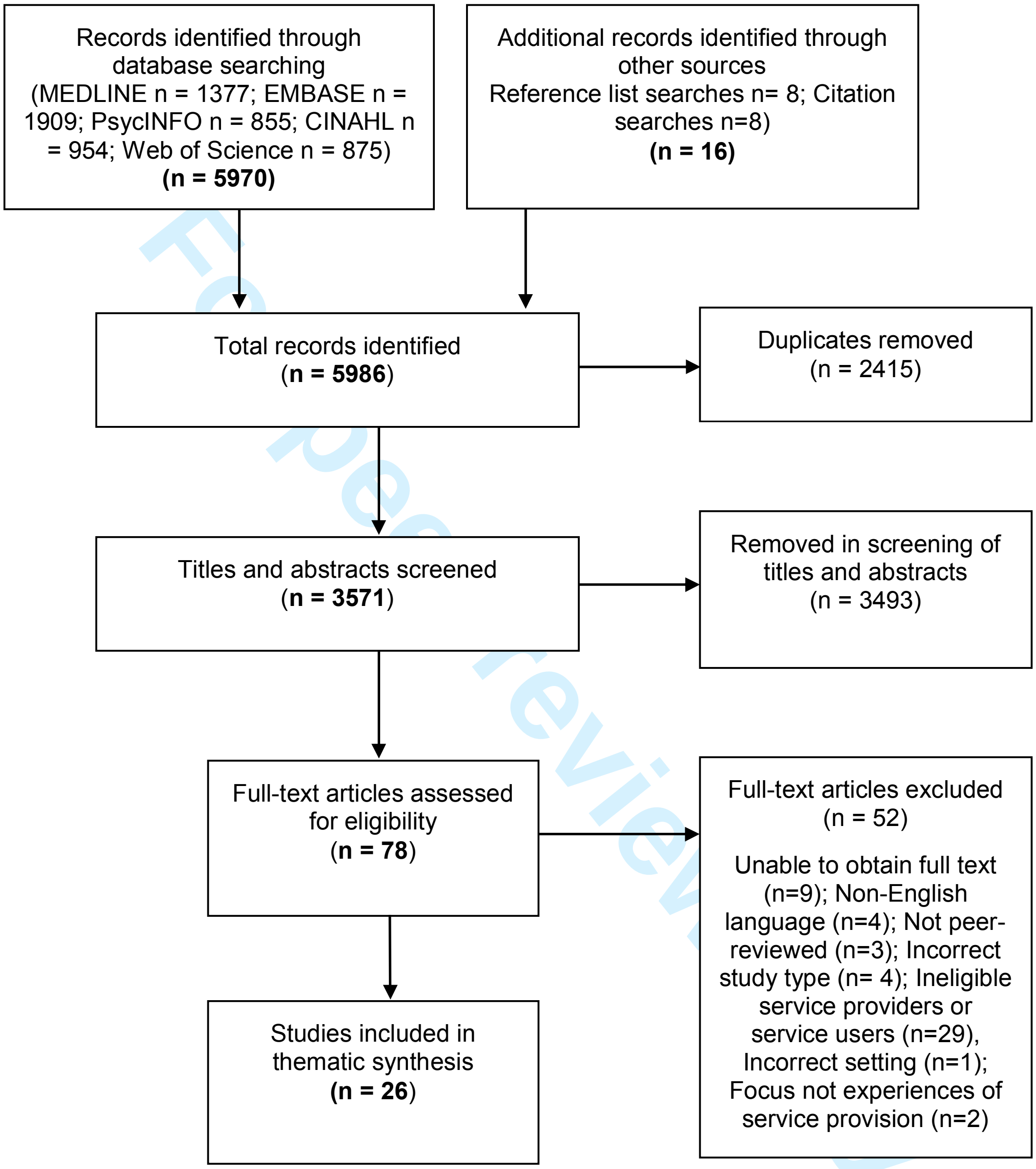

For peer review only - http://bmjopen.bmj.com/site/about/guidelines.xhtml

Characteristics of included studies

The 26 articles included were based on 21 primary studies of which 19 were qualitative studies [13, 14, 32-53] and two were mixed-methods.[15, 54] Nine articles were from Australia,[36, 38, 40-43, 45, 52, 53] seven from the United Kingdom,[14, 32-35, 37, 54] three from the Netherlands [39, 47, 48] and one from each of Denmark,[13] Switzerland,[15] New Zealand,[44] Sweden,[46] the United States,[49] Ireland [50] and Canada.[51] All articles were published between 1999 and 2016. Service users were described as ‘refugees’ in 11 articles,[13, 36, 38-44, 49, 51] ‘asylum seekers’ in six articles,[15, 33, 47, 48, 50, 54] ‘refugees and asylum seekers’ in five articles,[14, 32, 34, 35, 37] ‘of refugee background’ in three articles,[45, 52, 53] and ‘involuntary migrants’ in one article.[46]

Qualitative data extracted for this synthesis were derived from 357 participants with a combined sample of 194 nurses, 35 midwives and 128 doctors. None included pharmacists. Data collection methods varied across the 21 primary studies represented, with 14 solely using individual interviews (including in-depth, semi-structured, unstructured),[13-15, 32-35, 37, 39, 40, 42-44, 46, 50, 51, 54] One employed group interviews only,[49] and four combined individual and group interviews.[38, 41, 45, 52, 53] One study used observational methods and individual interviews,[36] and one combined group interviews and qualitative questionnaires.[47, 48] Table 1 summarises characteristics of included articles and online supplement 3 contains the complete data extraction.

For peer review only - http://bmjopen.bmj.com/site/about/guidelines.xhtml

Table 1 Characteristics of articles included in the thematic synthesis

First author	Publication year	Country	Eligible participants[a] and practice setting	Service users[b]	Data collection method	Analysis methodology	Study aims/objectives[c]
Begg, H.[32]	2005	United Kingdom	17 general practitioners<br><br>General practice	Refugees & asylum seekers	Semi-structured interviews	Thematic framework	To identify some of the concerns of 17 general Practitioners (GPs) working in an urban environment.
Bennett, S.[33]	2014	United Kingdom	10 midwives<br><br>Community, rotational, specialist and delivery suite midwives	Female asylum seekers	Semi-structured interviews	Thematic analysis	To gain an in depth analysis of the experiences of midwives and their understanding of the specific needs of asylum-seeking women. The findings would be used to inform education, practice and policy to enable more effective delivery of woman-centred care for this group locally.
Burchill, J.[34][d]	2011	United Kingdom	14 health visitors<br><br>London borough	Refugees and asylum seekers	In-depth interviews	Framework	Not clearly stated.
Burchill, J.[14][d]	2012	United Kingdom	14 health visitors<br><br>London borough	Refugees and asylum seekers	In-depth interviews	Framework	To determine the barriers to effective practice that health visitors when working with refugees and asylum seekers.
Burchill, J.[35][d]	2014	United Kingdom	14 health visitors<br><br>London borough	Refugees and asylum seekers	In-depth interviews	Framework	Explored the experiences of health visitors working with refugee and asylum-seeking families in central London, and assessed the dimensions of their cultural competency using Quickfall's model.
Carolan, M.[36]	2008	Australia	2 midwives<br><br>African women's clinic (community health centre)	Female African refugees	Observational methods and semi-structured interviews	Thematic analysis	To explore factors that facilitate or impede the uptake of antenatal care among African refugee women.
Crowley, P.[54][e]	2005	United Kingdom	10 general practitioners<br><br>General practice	Asylum seekers	Telephone interviews	Not specified	To assess the mental health care needs of adult asylum seekers in Newcastle upon Tyne.

For peer review only - http://bmjopen.bmj.com/site/about/guidelines.xhtml

First author	Publication year	Country	Eligible participants[a] and practice setting	Service users[b]	Data collection method	Analysis methodology	Study aims/objectives[c]
Drennan, V.[37]	2005	United Kingdom	13 health visitors<br><br>2 London borough's	Refugees and asylum seekers	Semi-structured interviews	Framework	Describe health visitors' experiences working in Inner London and identifying and addressing the health needs of refugee woman in the first 3 months after the birth of a baby.<br>Investigate health visitors' perceptions of effective and ineffective strategies in identifying and addressing health needs of these women.<br>Investigate whether health visitors used a framework corresponding to Maslow's theory of a hierarchy of needs to prioritize their public health work.
Farley, R.[38]	2014	Australia	20 general practitioners<br>5 practice nurses<br><br>General practice	Newly arrived refugees	Focus groups and Semi-structured interviews	Thematic analysis	Explored the experiences of primary health care providers working with newly arrived refugees in Brisbane...focusing on the barriers and enablers they continue to experience in providing care to refugees.
Feldmann, C.[39]	2007	Netherlands	24 general practitioners<br><br>General practice	Refugees (Afghan/Somali)	In-depth interviews	Thematic analysis	To confront the views of refugee patients and general practitioners in the Netherlands, focusing on medically unexplained physical symptoms (MUPS).
Furler, J.[40][†]	2010	Australia	8 family physicians<br><br>Community health centre	Refugees with depression	Semi-structured interviews	Thematic analysis	This study explores the complexities of this work [clinical care for depression] through a study of how family physicians experience working with different ethnic minority communities in recognizing, understanding, and caring for patients with depression.
Griffiths, R.[41]	2003	Australia	13 nurses<br>2 nurse managers<br><br>Refugee reception centre	Refugees	Focus groups and semi-structured interviews	Thematic analysis	To identify the skills, knowledge and support nurses require to provide holistic and competent care to refugee children and their families and the nature of support that is required to assist their transition back to mainstream health services.

For peer review only - http://bmjopen.bmj.com/site/about/guidelines.xhtml

First author	Publication year	Country	Eligible participants[a] and practice setting	Service users[b]	Data collection method	Analysis methodology	Study aims/objectives[c]
Jensen, N.[13]	2013	Denmark	9 general practitioners<br><br>Medical clinics	Refugees	Semi-structured interviews	Content analysis	To qualitatively explore issues identified by general practitioners as important in their experiences of providing care for refugees with mental health problems.
Johnson, D.[42]	2008	Australia	12 general practitioners<br><br>General practice	Refugees	Semi-structured interviews	Template analysis	To document the existence and nature of challenges for GPs who do this work in SA.<br>To explore the ways in which these challenges could be reduced.<br>To discuss the policy implications of this in relation to optimising the initial health care for refugees.
Kokanovic, R.[43][†]	2010	Australia	5 general practitioners<br><br>Community health centre	Refugees with depression	In-depth interviews	Thematic analysis	We explore a set of cultural boundaries across which depression is contested: between recent migrants to Australia from East Timor and Vietnam, and their white 'Anglo' family doctors.
Kurth, E.[15][e]	2010	Switzerland	3 physicians<br>3 nurses/midwives<br><br>Women's clinic	Female asylum seekers	Semi-structured interviews	Grounded theory	To investigate the reproductive health care provided for women asylum-seekers attending the Women's Clinic of the University Hospital in the city of Basel, Switzerland. To identify the health needs of asylum seekers attending the Women's Clinic and to investigate the health care they received in a Health maintenance organisation (HMO) specifically established for asylum seekers…Explored the perceptions of the health care professionals involved, about providing health care for this group in this setting.
Lawrence, J.[44]	2005	New Zealand	5 medical practitioners<br><br>Community health centre	Refugees	In-depth interviews	Thematic analysis	This paper reports on research that sought to reveal the barriers faced by refugees in accessing health services, and the challenges faced by providers in endeavouring to meet needs in an effective and culturally appropriate manner.

For peer review only - http://bmjopen.bmj.com/site/about/guidelines.xhtml

First author	Publication year	Country	Eligible participants[a] and practice setting	Service users[b]	Data collection method	Analysis methodology	Study aims/objectives[c]
Riggs, E.[45]	2012	Australia	12 nurses<br><br>Maternal and child health services	Refugee background mothers	Focus groups and Interviews	Thematic analysis	To explore the utilisation and experience of MCH services in Melbourne, Victoria for parents of refugee background from the perspective of users and providers.
Samarasinghe, K.[46]	2010	Sweden	34 primary health care nurses<br><br>Various: Maternity, child, school, community health care, nurse-led clinics.	Involuntary migrant families	Interviews	Contextual analysis	The aim of this study was to describe the promotion of health in involuntary migrant families in cultural transition as conceptualized by Swedish PHCNs.
Suurmond, J.[48][g]	2013	Netherlands	36 nurse practitioners<br>10 public health physicians<br><br>Asylum seeker centres	Newly arrived asylum seekers	Group interviews	Framework	To describe the tacit knowledge of Dutch healthcare providers about the care to newly arrived asylum seekers and to give insight into the specific issues that healthcare providers need to address in the first contacts with newly arrived asylum seekers.
Suurmond, J.[47][g]	2010	Netherlands	89 nurse practitioners (questionnaires)<br>36 nurse practitioners (group interviews)<br><br>Asylum seeker centres	Asylum seekers	Questionnaires and group interviews	Framework	We explored the cultural competences that nurse practitioners working with asylum seekers thought were important.
Tellep, T.[49]	2001	United States	6 school nurses<br><br>Schools	Refugees	Focus group	Unspecified	To describe the nature and meaning of school nurses' and Cambodian liaisons' experiences of caring for Cambodian refugee children and families and to explore whether those meanings validated Dobson's conceptual framework of transcultural health visiting.

For peer review only - http://bmjopen.bmj.com/site/about/guidelines.xhtml

First author	Publication year	Country	Eligible participants[a] and practice setting	Service users[b]	Data collection method	Analysis methodology	Study aims/objectives[c]
Tobin, C.[50]	2014	Ireland	10 midwives<br><br>Maternity hospitals	Female asylum seekers	In depth unstructured interviews	Content analysis	To explore midwives' perceptions and experiences of providing care to women in the asylum process and to gain insight into how midwives can be equipped and supported to provide more effective care to this group in the future.
Twohig, P.[51]	1999	Canada	6 family practice nurses<br>10 family physicians<br><br>Clinic at refugee processing centre	Refugees	Semi-structured interviews	Textual analysis	To explore roles of family physicians and family practice nurses who provided care to Kosovar refugees at Greenwood, NS.
Yelland, J.[52][h]	2014	Australia	10 Midwives<br><br>Maternity services	Refugee background families	Interviews and focus groups	Thematic analysis	(1) investigate Afghan women and men's experience of the way that health professionals approach inquiry about social factors affecting families having a baby in a new country, and (2) investigate how health professionals identify and respond to the settlement experience and social context of families of refugee background.
Yelland, J.[53][h]	2016	Australia	10 Midwives<br><br>Maternity services	Refugee background families	Interviews and focus groups	Thematic analysis	(1) describe Afghan women's and men's experiences of language support during pregnancy check-ups, labour and birth; (2) explore health professionals' experiences of communicating with Afghan and other refugee clients with low English proficiency; and (3) consider implications for health services and health policy.

[a]Some studies included some participants not eligible for this review. These participants have not been included on this table.
[b]Service users as described by the authors.
[c]The aims and objectives are from the author (i.e. extracted directly from papers.)
[d,h]These articles were based on data from the same sample, but reported different aspects.
[e]Mixed-methods were utilised in these studies. This table only includes characteristics of the qualitative element relevant to this review.
[f]The 5 GP's in Kokanovic 2010 are included within the 8 physicians in Furler 2010 but report different aspects.
[g]The 36 nurse practitioners are common between articles, but report different aspects.

For peer review only - http://bmjopen.bmj.com/site/about/guidelines.xhtml

Quality assessment

Application of the CASP critical appraisal tool revealed variable results across the 26 articles assessed. All except one article [34] gave a clear statement of the research aims. The majority (21 articles) [13-15, 32-35, 37-39, 41, 43, 44, 46-53] sufficiently described the sampling strategy and provided some rationale for participants' selection. Possible reasons for non-participation were discussed in only four articles.[15, 32, 41, 42] The data collection method was stated in all articles, however the extent of information provided about interview schedule's content was variable. A significant number did not describe the setting of data collection (13 articles) [33, 38-42, 45, 47, 48, 51-54] or the identities of interviewers (12 articles).[14, 15, 33-37, 41, 42, 50, 53, 54] Only eight articles [38, 42-45, 47, 51, 54] gave justification for chosen data collection methods or interview settings. Data saturation was rarely discussed, featuring in five articles.[32, 38, 42, 43, 51]

Reflexivity was particularly poorly discussed across articles. Only seven [32, 34, 38, 43, 46, 49, 50] reflected on potential bias and influence of researchers at any stage in the study (formulation of review question, sampling, data collection or analysis).

Ethical Approval was described in the majority of articles (23 articles),[13-15, 32-38, 40-43, 45-53] but they often lacked sufficient information to judge whether ethical standards had been followed. Thirteen articles [13-15, 33-38, 43, 46, 48, 50] described how participants were informed about the nature and purpose of the study, 17 articles [13, 15, 32, 33, 35, 37, 38, 43-51, 55] described obtaining consent and 12 articles [13, 32, 36-38, 41, 42, 46-50] discussed how confidentiality was assured or maintained.

The approach to data analysis was described to some extent in all but one article,[54] however there was variation in the level of detail given. Involvement of multiple researchers in the analysis process was reported in 19 articles.[13-15, 32, 34-38, 40-43, 45, 46, 50-53] The majority (25 articles) [13-15, 32-53] gave support for findings with references to primary data (e.g. quotations from participants). Findings were generally clearly presented and discussed in

For peer review only - http://bmjopen.bmj.com/site/about/guidelines.xhtml

For Peer Review Only

context of wider research literature, policy and practice, although a few (six articles) [34, 35, 37, 44, 49, 51] were limited in this area. Ten articles [15, 32, 36, 38, 40, 45-48, 52] explicitly reflected on the credibility of their findings.

Full details of the CASP assessment are provided in online supplement 4.

For peer review only

For peer review only - http://bmjopen.bmj.com/site/about/guidelines.xhtml

Thematic synthesis findings

Challenges and facilitators for health professionals providing primary healthcare to refugees and asylum seekers were interpreted within 11 descriptive themes, embedded in 3 analytical constructs: healthcare encounter (trusting relationship, communication, cultural understanding, health and social conditions, time), healthcare system (training and guidance, professional support, connecting with other services, organisation, resourcing and capacity), and asylum and resettlement. Figure 2 illustrates the relationships between analytical constructs and descriptive themes. Healthcare encounters occur within the environment of healthcare systems, both of which operate within wider asylum and resettlement policies and processes. Table 2 provides a taxonomy of challenges and facilitators and Table 3 contains illustrative quotations from primary studies for each descriptive theme.

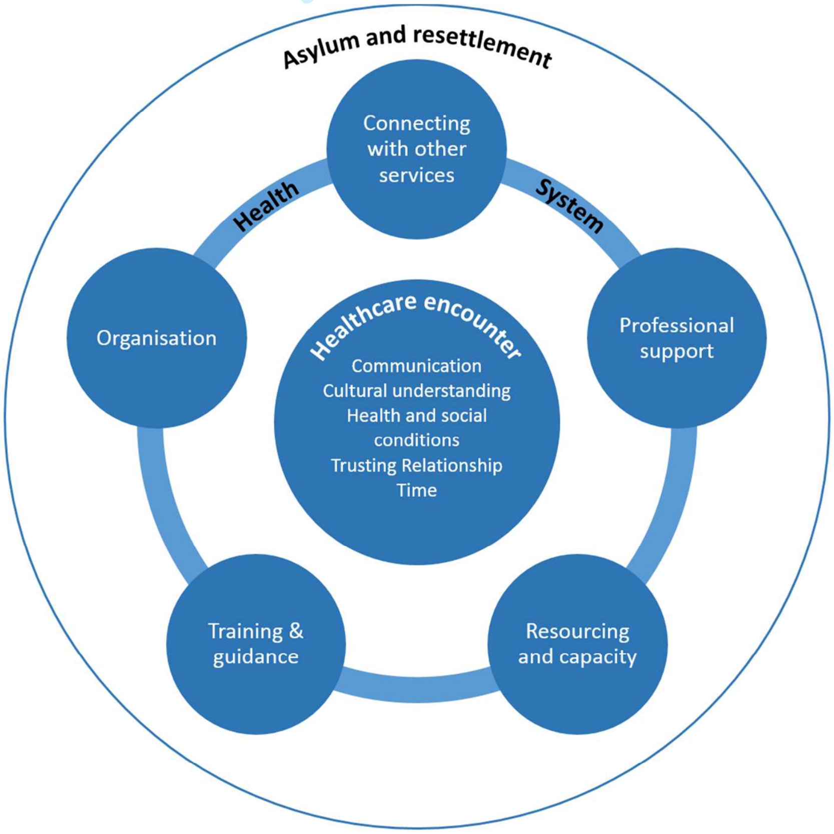

Figure 2: Model illustrating analytical constructs and descriptive themes

For peer review only - http://bmjopen.bmj.com/site/about/guidelines.xhtml

Table 2 Taxonomy of challenges and facilitators

Analytical construct	Descriptive theme	Challenges	Facilitators
Healthcare encounter	Trusting relationship	-Transience of refugees/ asylum seekers<br>-Suspicion of authorities	-Continuity of care<br>-Assisting with wider needs<br>-Taking an interest<br>-Compassion/empathy<br>-Explaining role
	Communication	-Language: assessing case history/gaining consent/ ensuring patient understanding<br>-Interpreters: additional time/ expense, unavailability, inaccuracy/imposition of own views<br>-Telephone interpreters: impersonal, technological failures<br>-Illiteracy<br>-Lack of language specific resources	-Interpreters: professionally trained, continuity<br>-Telephone interpreters: increased availability<br>-Visual aides
	Cultural understanding	-Different understandings of health concepts/terminology/ healthcare systems<br>-Understanding patient's symptoms<br>-High expectations of patients<br>-Different cultural values	-Knowledge of other cultures: values, health practices, body language<br>-Personal qualities - sensitivity, empathy, cultural humility
	Health and social conditions	- Physical: communicable diseases, FGM, Injuries<br>-Unusual diseases<br>-Psychological: torture, abuse, social difficulties, somatisation<br>-Lacking skills, knowledge, support	-Training<br>-Guidance<br>-Professional support<br>-History taking
	Time	-Increased time requirement<br>-Increased duration/occurrences of appointments<br>-Insufficient time – rushed appointments<br>-Time taken away from other patient groups	

For peer review only

For peer review only - http://bmjopen.bmj.com/site/about/guidelines.xhtml

Table 2 (continued)

Analytical construct	Descriptive theme	Challenge	Facilitator
The healthcare system	Training and guidance	-Lack of training/guidance<br>-Lack of awareness of available resources<br>-Time constraints	-Cultural competency training<br>-Orientation to services/resources/asylum process<br>-Culture specific information
	Professional support	-Deficiency of professional support<br>-Supporting traumatised patients without support<br>-Isolation	
	Connecting with other services	-Referral difficulties; services not present/not suitable<br>-Difficulty understanding/ navigating healthcare system	-Establishing referral pathways – health system/civil society<br>-Accompanying refugees and asylum seekers<br>-Communication/coordination /collaboration with other services<br>-Co-delivery of services<br>-Multi-agency teams
	Organisation		-Flexibility of primary healthcare system: innovation/adaptation<br>-Specialised services
	Resourcing and capacity	-Increased costs<br>-Funding shortages<br>-Workforce shortages<br>-Inflexibility/unsuitability of interpreter services	
Asylum and resettlement	Asylum and resettlement	-Policy restrictions<br>-Conflicts of interest<br>-Understanding changing policy environment and healthcare provisions<br>-Perceived abuses of system	-Training in asylum and resettlement policy/process

For peer review only

Table 3 Illustrative quotations

Theme		Quotation and reference[a]
**Healthcare encounter**		
Trusting relationship	Challenge:	*'… you put your mind around trying to sort things out, the dreadful things that have happened to them, and then the next week it will be a different family there and you start the whole process all over again, trying to build up some sort of trust…'*[37]
	Facilitator:	*'Creating trust is an important aspect, to show that you are interested in the person, not only in the disease; to show that you want to know something about the context. Sometimes it is difficult to find time for it in a busy practice, but I see it is a worthwhile investment'.[39]*
Communication	Challenge:	*'I've had some pretty bad examples recently of interpreters where they have actually started giving their opinion, which has been a nightmare, …they start adding their points of view.'[35]*<br>*'The phone interpreter is too impersonal. And I found that a lot of them use mobile phones so you're constantly cutting out...'*[45]
	Facilitator:	*'Everything comes down to communication. To know what's going on, what they need, what you need, because it's a partnership, isn't it?' [33]*<br>*'... this [telephone interpreting] is available 24 hours and is instantaneous ... it's revolutionised, all the doctors use it, the receptionists, the nurses….'* [32]
Cultural understanding	Challenge:	*'…they have a different culture, so their cultural perception of symptoms and what they mean . . . trying to interpret the difference between a bloated abdomen and a painful abdomen, just becomes an impossible task.'[38]*<br>*'I sometimes say, 'I am only a doctor'. Sometimes there are far greater expectations than what you can honour'[13]*<br>*'I think most people understand sadness…but in some cultures, they don't understand [depression] as a condition that requires treatment'*[40]
	Facilitator:	*'…there were specialized nurses who had worked overseas, who gave workshops for us, and explained much of the history, and explained some of the conflicts which they bring over here.'[49]*
Health and social conditions	Challenge:	*'I am quite overwhelmed at times as to how complex these ladies' lives are….'[33]*<br>*'I guess it is out of our comfort zone, because our medical experience doesn't include the exotic illnesses that they front up with...'*[42]<br>'Midwives spoke of the emotional impact of working with women with trauma histories: *"How does it affect me, you just feel sad you know, but you just do the best that you can and that's all you can do'[50]*
	Facilitator:	*'[Specialist team teaching sessions] is the sort of thing that people need to help give them a baseline of knowledge, and I suppose, the support to realise that there are other people they can talk to, to help them and signpost, or help them to signpost their clients in the right direction.'[35]*<br>*'We don't need to know the whole lot; we don't need the whole case history […] to have a bit more understanding.'[33]*
Time	Challenge	*'... generally speaking a consultation with a refugee will take twice as long [as with] a local patient.'[32]*<br>'…providing care with interpreters was more time consuming than without, meaning that midwives had to *'juggle their time'* to facilitate good care.'[33]

For peer review only

For peer review only - http://bmjopen.bmj.com/site/about/guidelines.xhtml

Table 3 (continued)

Theme			Quotation and reference[a]
**Healthcare system**			
	Organisation	Facilitator	'The flexibility of the general practice setting enabled providers to act on their commitment to provide refugee health care, allowing them to be responsive and innovative in their approach to caring for refugees and also providing flexibility in the hours they work.'[38] Participants felt that significant gains had been made to the refugee health care system, with the establishment of a specialised service. One provider working in the field for some time described thinking, *'. . . fantastic, finally'[38]*
	Resourcing and capacity	Challenge	*'... [asylum seekers] should be budgeted for ... they're actually slightly harder work than somebody else [this] needs to be acknowledged.'[32]* *'But I was more angry that I just needed more hands to help. So, for me it was about practical support.'[41]*
	Training and guidance	Challenge	*'Even when we called . . . the* [Division of General Practice] *. . . they didn't know how to guide us . . . I think we didn't have a guideline ...*'[38]
		Facilitator	'The specialist team facilitated a rolling programme of training for frontline staff working with refugees and asylum seekers, and this was regarded as an effective way of sharing knowledge.'[34]
	Professional support	Challenge	'…lack of institutional support all contributed to varying feelings of powerlessness on the part of the midwives themselves.'[50]
		Facilitator	'They described the value of currently available external supports, including language classes, translation and interpreting services, and specialised refugee health services, particularly in the area of mental health.'[38]
	Connecting with other services	Challenges	*'She explained she had seen a lot of problems…I put her touch with a voluntary [nationality specific] counselling organization to then discover she had to pay and she can't afford it.'[37]*
		Facilitator	*'so I referred her to ... and we went together for a joint meeting ... FORWARD [a women's campaign and support charity] specialises in FGM and I set her up for an appointment there and she was referred to a specialist nurse ... who was able to look at potentially reversing part of the FGM and the client was happy for this to happen and actually did attend.*'[35]
**Asylum and resettlement**			
		Challenge	*'These requirements differed: on the one hand to be the care giver, to be the patient's advocate in fact, and on the other to act as advocate of the Federal Office for Refugees, and thirdly to be responsible for the organisation, to save costs for the health insurance. But that is simply not possible.'[15]* *'I don't know if there is some sort of system that they go through, or some sort of protocol that they, medically, have to go through before they are granted visas...'[42]*

[a] Participant's quotations are in italics, study authors text is normal typeface.

For peer review only

For peer review only - http://bmjopen.bmj.com/site/about/guidelines.xhtml

The healthcare encounter

Challenges and facilitators for healthcare provision to refugees and asylum seekers were experienced within the healthcare encounter. This is the milieu of personal engagement between health professionals and service users. Five inter-related factors influenced health professionals' practice: Trusting relationship, communication, cultural understanding, health and social conditions, and time.

Trusting relationship

Building trusting relationships with refugees or asylum seekers featured in 15 of the articles.[14, 35-37, 39-41, 43, 45-50, 52] Facilitators included; continuity of the attending care provider;[37, 45, 47, 49, 52] taking an active interest in their background, language and culture;[35, 39, 49, 52] and assisting them with their wider needs.[14, 35, 45] Having a compassionate and empathetic disposition was also seen as important in relationship building.[36, 46, 47, 50, 52] The transient nature of some service users made building relationships challenging[37] and trust was threatened when refugees or asylum seekers thought that healthcare professionals were associated with immigration authorities.[33, 37, 47] Health professionals found that clearly explaining their role and confidentiality brought reassurance and allayed suspicions.[37, 47] Some benefits of establishing trusting relationships were said to be increased engagement with the healthcare service by refugees and asylum seekers [14, 35, 36, 45] and greater levels of disclosure about their health and social concerns.[37, 43, 45, 47, 52]

Communication

Communication was a theme found in 22 included articles.[13-15, 32, 33, 35, 37-40, 42-53] The language barrier was widely cited as challenging while caring for refugees and asylum seekers.[13-15, 32, 33, 35, 38, 39, 42-46, 48-53] Individual articles elaborated that language barriers presented difficulties in assessing case histories,[15] gaining consent [50] and ensuring patients understood treatment.[37]

For peer review only - http://bmjopen.bmj.com/site/about/guidelines.xhtml

Utilising interpreters was considered a major facilitator in communication [13, 32, 33, 35, 38, 40, 45, 46, 52] and was maximised when interpreters were well-trained and familiar with medical terminology.[13, 40] Continuity of the interpreter was deemed important in fostering good communication and increased confidence in the integrity of translation.[33, 35, 40, 45] There were, however, challenges associated with interpreter use.[13-15, 32, 33, 35, 37, 38, 40, 42, 44, 45, 47, 50, 51, 53] Communicating through interpreters required additional time [33, 42] and financial expense.[50] Suitable interpreters were not always available at the appropriate time,[13, 33, 37, 38, 42, 50] which could lead to delayed, extended or rearranged appointments.[13, 33, 42] This led, in some cases, to family or other community members being asked to translate instead of professional interpreters.[37, 50] Participants were also concerned that interpreters did not always accurately communicate [32, 35, 38, 40, 50, 51] and may impose their own views.[35, 38] The use of telephone interpreters received mixed opinions. Advocates hailed the increased availability of interpreters at any time of the day,[32] but others felt they were more impersonal [45, 53] and pointed to technological failures that hindered communication.[45, 53]

Further communication challenges included unavailability of written health information in service users' languages [48, 52] and in some cases patients were unable to read or write.[38] To improve communication with those with limited language skills, some participants used objects or other visual aids.[46]

Cultural understanding

Cultural understanding was a theme described across 21 articles.[13, 14, 32, 33, 35-44, 46-52] Healthcare provision could be challenging, when there were different understandings of health, illness or healthcare.[13, 14, 35, 39-44, 46, 48, 50] Health literacy could be limited [38, 42, 48] and different terms could be used to refer to health conditions.[14, 40, 43, 52] Healthcare concepts such as preventative care (e.g. screening),[42, 44] mental healthcare [43, 52] and self-management [46] were sometimes unfamiliar. Service users also lacked understanding host country's healthcare systems,[32, 35, 37, 38, 40, 44]

For peer review only

making them prone to miss appointments,[38] and attempt to inappropriately access services.[32]

Differences in health culture presented difficulties for health professionals' understanding of patient's symptoms [40] and required additional time and effort explaining health conditions, healthcare concepts or health systems.[37, 42, 46] It was also reported that some refugees or asylum seekers had very high, and sometimes unrealistic, expectations of health services or health professionals,[13, 32, 35, 47, 48] which needed to be counteracted by participants. [13, 48] Disparities in cultural values such as gender roles, decision-making, social taboos and time-orientation were also mentioned as challenges,[36, 42, 43, 48] with some health professionals expressing uncertainty about approaching some clinical tasks such as physical examinations.[42]

Gaining knowledge and understanding about cultures of refugees and asylum seekers was viewed as an important facilitator in cross-cultural care.[33, 35, 37, 42, 47, 49, 50, 52] This included understanding differences in values,[37] body language,[47] health practices [37] and health presentations[47]. Cultural understanding allowed health professionals to adjust their healthcare practice accordingly.[35, 40, 43, 44, 46, 50, 51] Personal qualities in health professionals that were deemed to enhance cross cultural interactions were sensitivity,[44, 47, 49] empathy [35, 36, 49] and cultural humility.[49, 50]

Health and social conditions

Health professionals spoke of challenges in dealing with physical, psychological and social problems that were typically presented by refugees and asylum seekers.[13, 32, 35, 38, 39, 41, 42, 47, 48, 50-52]

Physical conditions presented challenges[32, 35, 38, 39, 42] and included: tropical diseases such as malaria and schistosomiasis;[38] other communicable diseases such as TB and HIV;[32, 35, 39] and nutritional deficiencies.[32, 35, 39] Physical injuries were also encountered, such as female genital mutilation (FGM) [35, 50] and injuries inflicted from conflict or torture.[35] Health

For peer review only - http://bmjopen.bmj.com/site/about/guidelines.xhtml

professionals did not always feel prepared or equipped to deal with these conditions [38, 42] and there were concerns from general practitioners that some conditions could remain undiagnosed.[38, 39, 42]

Psychological conditions were considered challenging to deal with,[13, 32, 35, 38, 41, 47, 48, 50-52] and were frequently seen among refugees and asylum seekers.[32, 38] These included psychological trauma related to war,[13] torture [35, 38, 41] and other abuses.[13, 33, 35] Post-migration stresses were also perceived to impact negatively on their mental health such as the asylum and resettlement process, [13, 35, 42] social isolation,[13, 40, 50] and other social vulnerabilities.[35, 45, 52] Health professionals found engaging with these service users emotionally difficult,[32, 50] and distressing when hearing their disturbing stories.[35, 37, 41, 50] They also expressed feelings of powerlessness [13, 41, 50] believing they lacked required skills, knowledge and support to respond to their complex psychological needs.[38, 52]

A further challenge noted by health professionals across four articles was the manifestation of medically unexplained symptoms (somatisation) among some refugees and asylum seekers,[14, 38, 39, 43] which could be frustrating [38] and time consuming to address.[38, 43]

Several facilitators were identified that could help deal with complex physical and psychological conditions. Careful history-taking of medical, social and migration background was helpful [33, 39, 45, 48, 52] and could identify possible risk-factors.[48, 52] Training in conditions common among refugees and asylum seekers was deemed valuable,[32, 33, 35, 41, 47, 48, 50] increasing confidence in care delivery [35] and resulting in 'more effective, evidence based care'.[33] Clinical guidelines for refugee healthcare were considered beneficial [32, 42] although these were often unavailable.[32, 42] Professional support was regarded as a facilitator,[32, 33, 37, 38, 41, 46, 50] provided within services [37] or from external organisations specialising in refugee healthcare.[38, 41] The importance of psychological support for those working with traumatised patients was highlighted,[41, 46, 50] such as

For peer review only - http://bmjopen.bmj.com/site/about/guidelines.xhtml

counselling or debriefing.[41, 46] Challenges around training, guidance and professional support are described in 'The healthcare system' section.

Time

A significant challenge faced by health professionals was the time required to provide healthcare for refugees and asylum seekers.[14, 32, 33, 35, 38, 42, 44-46, 50, 51, 54] More time was necessary due to the aforementioned challenges around building relationships,[14, 33, 35] communication,[33, 45, 50, 54] achieving cultural understanding,[42] and dealing with complex health conditions.[14, 33, 42, 45, 46] This additional time demand meant that appointments needed to be extended in duration [32, 42] or occur more frequently.[14, 44] Health professionals were concerned that time limitations could lead to 'rushed consultations' [54] and the potential to miss some conditions.[54] Some also commented that the extra time spent caring for refugees and asylum seekers drew them away from other patient groups.[35, 38]

The healthcare system

Health systems have been defined as "the combination of resources, organization, financing and management that culminate in the delivery of health services to the population".[56] They are the environment in which healthcare encounters take place. Healthcare professionals described health system related challenges and facilitators within 5 areas: training and guidance, professional support, connecting with other services, organisation, and resourcing and capacity.

Training and guidance

As already described in 'health and social conditions', health professionals thought that specific training and guidance would facilitate their clinical practice, improving their competence and confidence. Positive examples of training delivered were: orientation to services and resources available for refugees and asylum seekers;[35] culture specific information;[37, 49] engaging with women about FGM;[35] and trauma-sensitive care.[41] Despite this, a broad base of

For peer review only - http://bmjopen.bmj.com/site/about/guidelines.xhtml

participants identified lack of training, education or guidance as detrimental for practice.[13, 32, 33, 37, 38, 41, 44, 45, 50] Even when available, training may be inaccessible due to lack of awareness or time constraints.[38] Participants called for more training, guidance or information regarding integration with other health and social care services,[32, 37, 45] health profiles of specific groups,[41] cultural awareness/competence,[37, 41, 42, 44, 45] and the wider process of asylum.[32, 37]

Professional support

As reported in the earlier section ‘health and social conditions’, professional support was needed by health professionals working with refugees and asylum seekers. However professional support was identified as deficient in healthcare systems.[32, 38, 41, 50] Participants in one study described ‘isolation’ [38] that they felt within the healthcare system and another study described support networks as ‘non-existent’.[32] Concerns were raised that health professionals exposed to distressing stories were not provided with sufficient psychological support.[41, 50]

Connecting with other services

Connecting with other health and social care services was another important facilitator for health professionals.[13, 14, 33, 35, 37, 42, 44-47, 49] Establishing referral pathways to different services in the healthcare system [35, 37, 42, 46, 47] and services within civil society [35, 37, 42] could direct refugees and asylum seekers to appropriate care. Some health visitors described accompanying refugees and asylum seekers to support groups to help with introductions.[35, 37] Good communication and cooperation between services was helpful [33] and fruitful collaborations with other services were recognised, such as delivering services together [45, 46] and working in multiagency teams to deliver holistic healthcare.[33, 46, 49]

Health professionals spoke of some difficulties referring refugees and asylum seekers to other health or social services.[13, 14, 34, 35, 45, 50] Some, services were not set up to meet their needs,[13, 35] others would not receive

For peer review only - http://bmjopen.bmj.com/site/about/guidelines.xhtml

referrals because they were operating at full capacity [14, 34] and sometimes services were simply not present.[14, 50] These challenges could be accentuated when health professionals themselves found it difficult to navigate complex healthcare systems themselves.[38]

Organisation

Some articles highlighted flexibility in primary healthcare systems as beneficial in practice among refugees and asylum seekers.[35, 36, 38, 44, 45] This allowed for innovative approaches to optimise service delivery [35, 38] such as relocating services to more accessible places [14, 35, 37, 45] and adaptation of working patterns to better suit service users' needs.[38, 45]

Provision of specialised services for refugees and asylum seekers was supported across some studies,[32, 35, 38, 42] including initial health assessment services,[42] specialist teams [35, 42] and specialist centres.[32, 42] However, it was emphasised that these should integrate well into mainstream healthcare services.[32, 35]

Resourcing and capacity

Longer, more frequent appointments and utilisation of interpreters led to additional costs being incurred,[14, 15, 32, 38, 42, 44, 46] which some felt was not taken into account in health system financing models.[38, 42, 44] Some participants did not think that they could deliver adequate care as a result of funding shortages,[32, 50] with one study citing an example where interpreters were not able to be utilised because of lack of finance.[50]

Shortages in workforces were reported in some articles,[41, 42, 44] putting additional workload and stress onto health professionals.[41, 44] Reported consequences of this were closures of services to new patients [42, 44] and health professionals leaving their posts, further exacerbating the problem.[44] Interpreter shortages were also mentioned as a difficulty [41, 44, 51] along with inflexibility of their service operations.[32, 37, 50]

Asylum and resettlement

For peer review only - http://bmjopen.bmj.com/site/about/guidelines.xhtml

Further challenges were associated with the immigration status of, and legislative policy towards, refugees and asylum seekers.[14, 15, 32, 34, 35, 41, 42, 54] In some instances, health professionals were hindered in meeting health needs due to policy restrictions.[35] Difficulties understanding the frequently-changing policies towards, and entitlements for, refugees and asylum seekers were reported [34, 35] and uncertainty was expressed about healthcare pathways for this group upon arrival in the host country.[42] Some health professionals described conflicts in their professional duty to act as an advocate for their patients whilst requirements were placed on them to conduct assessments used to inform the asylum process.[15, 41] Another concern raised was a perception that service users were abusing the health and welfare systems,[14, 32, 35, 54] such as feigning symptoms of post-traumatic stress disorder to further their asylum claims [32] or illegal benefit claims.[14]

For peer review only

For peer review only - http://bmjopen.bmj.com/site/about/guidelines.xhtml

Discussion

Three analytical constructs containing 11 descriptive themes were interpreted in the thematic synthesis. Challenges and facilitators were located within the healthcare encounter (trusting relationships; communication; cultural understanding; health and social conditions; time), working within the healthcare system (training and guidance; professional support; connecting with other services; organisation; resourcing and capacity) and asylum and resettlement.

The growing research field of ‘cultural competence’, identifies components that can be incorporated into practice to enhance quality of care towards ethnic minority groups and reduce healthcare inequalities.[57, 58] Betancourt et al [57] defined cultural competence in healthcare as “the ability of systems to provide care to patients with diverse values, beliefs and behaviors, including tailoring delivery to meet patients’ social, cultural, and linguistic needs”.[57] This literature mirrors themes interpreted in the current review, including trusting relationships, communication and cultural understanding, as key components that may be optimised to improve healthcare and reduce inequalities.[57, 58]

Trusting relationships are essential for effective healthcare delivery [59-61] Murray et al [62] identified continuity of relationship, time, interpersonal skills and ‘getting to know patients’ as enhancers of trust between health professionals and patients. The current review likewise recognised these elements, and it can be argued that even greater attention to trust-building is needed for refugees and asylum seekers, a vulnerable and ethnically diverse group who may be apprehensive about engagement with healthcare systems.[63, 64]

Communication between health professionals and patients is also regarded as essential.[65] Language discordance may compromise the quality of healthcare, lessening detection of ill health and referral to further healthcare.[66, 67] Health professionals in the current review consistently thought language barriers hindered their work with refugees and asylum seekers. The main

For peer review only - http://bmjopen.bmj.com/site/about/guidelines.xhtml

strategy used to overcome language barriers was communication through interpreters, as is recommended in the wider literature.[68-70] However, concerns were raised about the quality and availability of interpreters. Generally, it is recommended that professional interpreters are used, as they have been trained in professional standards, medical terminology and ethical issues.[70] Ad-hoc interpreters such as family or community members may be used pragmatically, although this may diminish the quality of interpretation and threaten patient confidentiality.[69, 70] Remote interpretation, such as telephone or video services have been developed to provide more efficient and timely services.[71, 72] The merits of such services have been debated [71, 72] and conflicting opinions were likewise given in this review. A systematic review [72] reported no significant difference in patient and provider satisfaction between remote and face-to-face interpreters, although subsequent primary studies have suggested a significant preference for in-person interpreters.[71]

Consistent with other research, [6-8, 10-12] health professionals encountered challenges dealing with complex physical, psychological and social problems of refugees and asylum seekers and did not always feel prepared to meet their needs. They also reported challenges in cross-cultural care such as different understandings of health, healthcare and healthcare systems, which introduced complications.

Participants in this review saw opportunities for improving care by working together with other health services and civil society. Identifying these organisations and possible areas of collaboration such as information sharing, referral pathways and joint service delivery may benefit health providers, health professionals and service users.

The organisation and delivery of primary healthcare services to refugees and asylum seekers is a growing research area, with service models being developed that integrate specialised components with existing structures.[73, 74] A model innovated in Australia established ‘Beacon practices’, which have expanded capacity for refugee care and may flexibly resource local services.[74] Such integrated services provide specialised resources without

For peer review only - http://bmjopen.bmj.com/site/about/guidelines.xhtml

isolating refugees and asylum seekers from general practice, which was a concern raised by some participants in this review.

Health professionals and health services operate within, and are influenced by, the wider healthcare policy environment. Decisions made at a political and health system levels invariably impact on front-line clinical practice in areas such as resourcing priorities, health professional roles and healthcare access.[75] Health professionals in this review recognised associated challenges, particularly when healthcare pathways were unclear and changeable. This emphasises the need for policy-makers to provide consistant, clear and up-to-date guidance on asylum and resettlement health policy for health professionals.

Public health implications

A central concern in public health is reduction of inequalities in health and healthcare.[76, 77] The WHO has established a commission on the social determinants of health that recommends actions addressing inequalities in health.[77] Healthcare inequalities exist when certain groups systematically receive lower quality care than the general population, resulting in poorer health outcomes.[75, 78] These inequalities have been widely observed in healthcare provision to ethnic minority groups across a broad range of health services [75] and has been highlighted as an issue for refugees and asylum seekers in the UK.[16] However, through knowledge translation, where evidence is moved into practice, challenges and facilitators identified in this review may be mapped onto components of healthcare interventions that may minimise such healthcare inequalities.[79]

Reduction in healthcare inequalities will likely require targeting healthcare resources towards disadvantaged groups.[74] For example, health professionals in this review highlighted the need for additional resources such as interpreter services, training and professional support to improve quality of care for refugees and asylum seekers.

For peer review only - http://bmjopen.bmj.com/site/about/guidelines.xhtml

Recommendations

Practice

Health professionals should be sufficiently resourced to meet the complex needs of refugees and asylum seekers. This should include provision of appropriate training on areas of cultural competence, asylum policies and process and health conditions. It is recommended that specific clinical guidelines are developed for provision of care to refugees and asylum seekers, drawing on the best available evidence. Further professional support should be given to those working with patients who present with complex psychological and social difficulties. Relevant, up to date information should be made available to inform health professionals about the needs of current waves of refugees and asylum seekers and other available services for referral and collaboration. Health providers should ensure adequate time is allocated for appointments with refugees and asylum seekers allowing space for trust building, communication and cultural understanding and develop infrastructure to ensure that trained interpreters are provided in a timely manner for appointments. Where resources permit, face to face interpreters should be utilised as a gold standard, with telephone interpreters used when these are unavailable.

Policy

Healthcare policy makers and commissioners should recognise the complex needs of refugees and asylum seekers, providing enhanced resources for quality and equitable service provision. Integration of specialised components with existing general practice may facilitate care. Asylum and resettlement policy makers should seek to promote continuity of relationship with healthcare providers, limiting relocations.

Research

Further systematic reviews could be conducted to investigate experiences of health professionals working with refugees and asylum seekers in other areas of the healthcare system. A systematic review of challenges and facilitators for

For peer review only - http://bmjopen.bmj.com/site/about/guidelines.xhtml

mental health professionals providing services to refugees and asylum seekers could inform service delivery for this group and searches in for this current review identified primary studies that could be included.

The outputs from this review may be used to inform service models for refugees and asylum seekers. Healthcare evaluations may be conducted to evaluate these models and identify areas that are able to improve quality of care

Strengths and limitations

An extensive and systematic search that was carried out across four databases complemented by reference and citation searches and it is therefore unlikely that published studies would have been overlooked. The inclusion of only English language studies may have led to under-representation of health professionals working in non-English speaking countries leading to a greater applicability to healthcare policy and practice in English speaking high-income countries.

A limitation was the involvement of a second reviewer in the detailed study selection stage only. Ideally, if more resources were available, the screening stage would also have been conducted in duplicate, minimising the potential for selection bias. A second reviewer in data extraction could have reduced possibility of transcription errors, and in the quality appraisal stage could have minimised potential for biased assessment. Ideally, the analysis process would also have involved multiple reviewers in coding and formation of descriptive and analytical themes, bringing a wider perspective to interpretation.

Participants in this review were limited to the core clinical professions of nurses, primary care doctors and midwives. Other professionals, that may be part of primary healthcare teams, such as mental health workers, counsellors, physiotherapists and other community workers, were not included, raising a question about the transferability to more diverse primary healthcare teams. Studies including other professional groups report similar themes to the present review, however those including mental health professionals may have a greater emphasis on secondary stress experienced when working with

For peer review only

For peer review only - http://bmjopen.bmj.com/site/about/guidelines.xhtml

traumatised patients.[80, 81] A further consideration for transferability of these findings is the combining of data from the three clinical professions as they have different care practices, interaction with patients and support networks, giving the potential to introduce imprecision to the findings.

A strength of syntheses of qualitative research is that concepts are translated across studies, with common themes described that may be more transferable to other contexts and a greater ability to inform policy and practice.[24, 82] This contrasts with primary qualitative studies that are tied to their context and transference of findings is treated with caution.[24, 82] On the other hand, a perceived limitation of thematic syntheses is that they introduce a greater degree of abstraction from original experiences, sacrificing thickness of data and details found within the primary studies.[83] In this case, given that refugees are not a homogeneous group, it is perhaps acceptable to emphasise only the more generalised themes that transcend the contexts of individual studies.

Conclusions

Many people continue to be displaced due to conflict and persecution, seeking sanctuary in high-income countries. Health professionals experience a range of challenges and facilitators providing primary healthcare for this vulnerable group within the healthcare encounter, the environment of the healthcare system and in the broader context of asylum and resettlement policy and process. These challenges and facilitators provide valuable insight to inform practice and policy, supporting quality healthcare and minimising healthcare inequalities for refugees and asylum seekers.

For peer review only

For peer review only - http://bmjopen.bmj.com/site/about/guidelines.xhtml

Footnotes

Contributors

LR designed the study, undertook the searches, data extraction, appraisal, synthesis and wrote the first draft of the manuscript. LLJ and SD supported study design, analysis, synthesis and interpretation, and provided critical revisions to the manuscript. LLJ had senior oversight and is the study guarantor. All authors revised the manuscript critically for important intellectual content and approved the final version for publication. All authors agree to be accountable for all aspects of the work.

Acknowledgements

The authors acknowledge the contribution of Madeline Flawn who was a second reviewer in study selection stage of the review.

Funding

This research received no specific grant from any funding agency in the public, commercial or not-for-profit sectors.

Competing interests

None

Data sharing

No additional data are available.

For peer review only

References

1 UNHCR. History of UNHCR. 2016;2016.

2 Anonymous . Convention and Protocol Relating to the Status of Refugees. 2010.

3 UNHCR. Global Trends; Forced Displacement in 2015. 2016.

4 Aspinall P. Hidden Needs. Identifying Key Vulnerable Groups in Data Collections: Vulnerable Migrants, Gypsies and Travellers, Homeless People, and Sex Workers . 2014.

5 Daniel M, Devine C, Gillespie R, et al. Helping new refugees integrate into the UK: baseline data analysis from the Survey of New Refugees. 2010.

6 Clark RC, Mytton J. Estimating infectious disease in UK asylum seekers and refugees: a systematic review of prevalence studies, *Journal of Public Health* 2007;29:420-8.

7 Allotey P. The health of refugees: public health perspectives from crisis to settlement.: Oxford University Press 2003.

8 Keygnaert I, Vettenburg N, Temmerman M. Hidden violence is silent rape: sexual and gender-based violence in refugees, asylum seekers and undocumented migrants in Belgium and the Netherlands. *Culture, Health & Sexuality* 2012;14:505-20.

9 Aptekman M, Rashid M, Wright V, et al. Unmet contraceptive needs among refugees. *Canadian Family Physician* 2014;60:e613-9.

10 Kalt A, Hossain M, Kiss L, et al. Asylum seekers, violence and health: a systematic review of research in high-income host countries, *Am J Public Health* 2013;103:e30-42.

11 Miller KE, Worthington GJ, Muzurovic J, et al. Bosnian refugees and the stressors of exile: a narrative study. *Am J Orthopsychiatry* 2002;72:341-54.

12 Fazel M, Wheeler J, Danesh J. Prevalence of serious mental disorder in 7000 refugees resettled in western countries: a systematic review, *Lancet* 2005;365:1309-14.

13 Jensen NK, Norredam M, Priebe S, et al. How do general practitioners experience providing care to refugees with mental health problems? A qualitative study from Denmark. *BMC Family Practice* 2013;14:17.

For peer review only

For peer review only - http://bmjopen.bmj.com/site/about/guidelines.xhtml

14 Burchill J, Pevalin D. Barriers to effective practice for health visitors working with asylum seekers and refugees. *Community Practitioner* 2012;85:20-3.

15 Kurth E, Jaeger FN, Zemp E, et al. Reproductive health care for asylum-seeking women - a challenge for health professionals. *BMC Public Health* 2010;10:659.

16 Jones D, Gill PS. Refugees and primary care: tackling the inequalities, *BMJ* 1998;317:1444-6.

17 Starfield B. Is primary care essential? *The Lancet* 1994;344:1129-33.

18 World Health Organization. WHO Nursing and Midwifery Progress Report 2008-2012. 2013.

19 Burke M. The perceived experiences of primary healthcare professionals in Ireland: interprofessional teamwork in practice. 2016.

20 Suphanchaimat R, Kantamaturapoj K, Putthasri W, et al. Challenges in the provision of healthcare services for migrants: a systematic review through providers' lens. *BMC Health Services Research* 2015;15:390.

21 Holloway I. Qualitative Research in Nursing and Healthcare. Somerset: Wiley 2013.

22 Pope C, van Royen P, Baker R. Qualitative methods in research on healthcare quality, *Qual Saf Health Care* 2002;11:148-52.

23 Harden A, Garcia J, Oliver S, et al. Applying systematic review methods to studies of people's views: an example from public health research, *J Epidemiol Community Health* 2004;58:794-800.

24 Finfgeld□Connett D. Generalizability and transferability of meta□synthesis research findings, *J Adv Nurs* 2010;66:246-54.

25 Thomas J, Harden A. Methods for the thematic synthesis of qualitative research in systematic reviews. *BMC Medical Research Methodology* 2008;8:45.

26 Barnett-Page E, Thomas J. Methods for the synthesis of qualitative research: a critical review, *BMC medical research methodology* 2009;9:1.

27 Tong A, Flemming K, McInnes E, et al. Enhancing transparency in reporting the synthesis of qualitative research: ENTREQ, *BMC medical research methodology* 2012;12:1.

28 Cooke A, Smith D, Booth A. Beyond PICO: the SPIDER tool for qualitative evidence synthesis, *Qual Health Res* 2012;22:1435-43.

For peer review only - http://bmjopen.bmj.com/site/about/guidelines.xhtml

29 The World Bank. World Bank list of economies (July 2015) [Data File]. 2015;2016.

30 Critical Appraisal Skills Programme (CASP). *CASP Checklists* . 2014;2016.

31 Dixon-Woods M, Bonas S, Booth A, et al. How can systematic reviews incorporate qualitative research? A critical perspective, *Qualitative research* 2006;6:27-44.

32 Begg H, Gill PS. Views of general practitioners towards refugees and asylum seekers: an interview study, *Diversity Health Soc Care* 2005;2:299,305 7p.

33 Bennett S, Scammell J. Midwives caring for asylum-seeking women: research findings. *Pract Midwife* 2014;17:9-12.

34 Burchill J. Safeguarding vulnerable families: work with refugees and asylum seekers. *Community Practitioner* 2011;84:23-6.

35 Burchill J, Pevalin DJ. Demonstrating cultural competence within health-visiting practice: working with refugee and asylum-seeking families, *Diversity Equality Health Care* 2014;11:151,159 9p.

36 Carolan M, Cassar L. Pregnancy care for African refugee women in Australia: attendance at antenatal appointments, *Evid Based Midwifery* 2007;5:54,58 5p.

37 Drennan VM, Joseph J. Health visiting and refugee families: issues in professional practice, *J Adv Nurs* 2005;49:155,163 9p.

38 Farley R, Askew D, Kay M. Caring for refugees in general practice: perspectives from the coalface. *Australian Journal of Primary Health* 2014;20:85-91.

39 Feldmann CT, Bensing JM, de Ruijter A. Worries are the mother of many diseases: General practitioners and refugees in the Netherlands on stress, being ill and prejudice. *Patient Educ Couns* Mar 2007;65:369-80.

40 Furler J, Kokanovic R, Dowrick C, et al. Managing depression among ethnic communities: A qualitative study. *Annals of Family Medicine* May-Jun 2010;8:231-6.

41 Griffiths R, Emrys E, Lamb CF, et al. Operation Safe Haven: The needs of nurses caring for refugees. *Int J Nurs Pract* Jun 2003;9:183-90.

42 Johnson D.R., Ziersch A.M., Burgess T. I don't think general practice should be the front line: Experiences of general practitioners working with refugees in South Australia. *Australia and New Zealand Health Policy* 2008;5:Arte Number: 20. ate of Pubaton: 08 Aug 2008.

For peer review only - http://bmjopen.bmj.com/site/about/guidelines.xhtml

43 Kokanovic R, May C, Dowrick C, et al. Negotiations of distress between East Timorese and Vietnamese refugees and their family doctors in Melbourne. *Sociol Health Illn* May 2010;32:511-27.

44 Lawrence J, Kearns R. Exploring the 'fit' between people and providers: refugee health needs and health care services in Mt Roskill, Auckland, New Zealand. *Health & Social Care in the Community* 2005;13:451-61.

45 Riggs E, Davis E, Gibbs L, et al. Accessing maternal and child health services in Melbourne, Australia: Reflections from refugee families and service providers, *BMC Health Serv Res* 2012;12:117,117 1p.

46 Samarasinghe K, Fridlund B, Arvidsson B. Primary health care nurses' promotion of involuntary migrant families' health, *Int Nurs Rev* 2010;57:224-31.

47 Suurmond J, Seeleman C, Rupp I, et al. Cultural competence among nurse practitioners working with asylum seekers, *Nurse Educ Today* 2010;30:821,826 6p.

48 Suurmond J, Rupp I, Seeleman C, et al. The first contacts between healthcare providers and newly-arrived asylum seekers: A qualitative study about which issues need to be addressed. *Public Health* Jul 2013;127:668-73.

49 Tellep TL, Chim M, Murphy S, et al. Great suffering, great compassion: A transcultural opportunity for school nurses caring for Cambodian refugee children. *Journal of Transcultural Nursing* Oct 2001;12:261-74.

50 Tobin C.L., Murphy-Lawless J. Irish midwives' experiences of providing maternity care to non-Irish women seeking asylum. *International Journal of Women's Health* 2014;6:159-69.

51 Twohig PL, Burge F, MacLachlan R. Pod people. Response of family physicians and family practice nurses to Kosovar refugees in Greenwood, NS. *Canadian Family Physician* 2000;46:2220-5.

52 Yelland J, Riggs E, Wahidi S, et al. How do Australian maternity and early childhood health services identify and respond to the settlement experience and social context of refugee background families?. *BMC Pregnancy & Childbirth* 2014;14:348.

53 Yelland J, Riggs E, Szwarc J, et al. Compromised communication: a qualitative study exploring Afghan families and health professionals' experience of interpreting support in Australian maternity care, *BMJ Qual Saf* 2016;25:e1,2014-003837. Epub 2015 Jun 18.

54 Crowley P. The mental health needs of adult asylum seekers in Newcastle upon Tyne, *Journal of Public Mental Health* 2005;4:17-23.

For peer review only - http://bmjopen.bmj.com/site/about/guidelines.xhtml

For peer review only

55 Feldmann T. What do refugees and general practitioners say about medically unexplained physical symptoms? Medical errors undermine trust in the GP. *Huisarts en Wetenschap* 2007;50:381-4.

56 Roemer MI. National health systems of the world: Oxford University Press 1993.

57 Betancourt J, Green A, Carillo J. Cultural competence in health care: Emerging frameworks and practical approaches. 2002.

58 Saha S, Beach MC, Cooper LA. Patient centeredness, cultural competence and healthcare quality, *J Natl Med Assoc* 2008;100:1275-85.

59 Hall MA, Dugan E, Zheng B, et al. Trust in physicians and medical institutions: what is it, can it be measured, and does it matter? *Milbank Q* 2001;79:613-39.

60 Mainous AG, Baker R, Love MM, et al. Continuity of care and trust in one's physician: evidence from primary care in the United States and the United Kingdom, *Fam Med* 2001;33:22-7.

61 Mechanic D. The functions and limitations of trust in the provision of medical care, *J Health Polit Policy Law* 1998;23:661-86.

62 Murray B, McCrone S. An integrative review of promoting trust in the patient–primary care provider relationship, *J Adv Nurs* 2015;71:3-23.

63 Duncan GF. Refugee Healthcare: Towards Healing Relationships, *Canadian Social Science* 2015;11:158-68.

64 Peterson P, Sackey D, Correa-Velez I, et al. Building trust: delivering health care to newly arrived refugees. 2011;2016:1-16.

65 Ong LM, De Haes JC, Hoos AM, et al. Doctor-patient communication: a review of the literature, *Soc Sci Med* 1995;40:903-18.

66 Bischoff A, Bovier PA, Isah R, et al. Language barriers between nurses and asylum seekers: their impact on symptom reporting and referral, *Soc Sci Med* 2003;57:503-12.

67 Timmins CL. The impact of language barriers on the health care of Latinos in the United States: a review of the literature and guidelines for practice, *Journal of Midwifery & Women's Health* 2002;47:80-96.

68 Flores G. The impact of medical interpreter services on the quality of health care: a systematic review, *Med Care Res Rev* 2005;62:255-99.

For peer review only

For peer review only - http://bmjopen.bmj.com/site/about/guidelines.xhtml

69 Karliner LS, Jacobs EA, Chen AH, et al. Do professional interpreters improve clinical care for patients with limited English proficiency? A systematic review of the literature, *Health Serv Res* 2007;42:727-54.

70 Mayo R, Parker VG, Sherrill WW, et al. Cutting Corners: Provider Perceptions of Interpretation Services and Factors Related to Use of an Ad Hoc Interpreter, *Hisp Health Care Int* 2016;14:73-80.

71 Locatis C, Williamson D, Gould-Kabler C, et al. Comparing in-person, video, and telephonic medical interpretation, *Journal of general internal medicine* 2010;25:345-50.

72 Azarmina P, Wallace P. Remote interpretation in medical encounters: a systematic review, *J Telemed Telecare* 2005;11:140-5.

73 Feldman R. Primary health care for refugees and asylum seekers: A review of the literature and a framework for services, *Public Health* 2006;120:809-16.

74 Kay M, Jackson C, Nicholson C. Refugee health: a new model for delivering primary health care, *Australian Journal of Primary Health* 2010;16:98-103.

75 Smedley BD, Stith AY, Nelson AR. Unequal treatment: Confronting racial and ethnic disparities in health care (full printed version): National Academies Press 2002.

76 Arcaya MC, Arcaya AL, Subramanian S. Inequalities in health: definitions, concepts, and theories, *Revista Panamericana de Salud Pública* 2015;38:261-71.

77 World Health Organization. Closing the gap in a generation: health equity through action on the social determinants of health. Final Report of the Commission on Social Determinants of Health. 2008.

78 Betancourt JR, Green AR, Carrillo JE, et al. Defining cultural competence: a practical framework for addressing racial/ethnic disparities in health and health care, *Public Health Rep* 2003;118:293-302.

79 Colquhoun H, Grimshaw J, Wensing M. Chapter 3.3b Mapping KT interventions to barriers and facilitators. In: Straus S, Tetroe J, Graham I, eds. Knowledge translation in health care: moving from evidence to practice: John Wiley & Sons 2013:137-49.

80 Puvimanasinghe T, Denson L, Augoustinos M, et al. Vicarious resilience and vicarious traumatisation: Experiences of working with refugees and asylum seekers in South Australia. *Transcultural Psychiatry* 2015;52:743-65.

For peer review only

For peer review only - http://bmjopen.bmj.com/site/about/guidelines.xhtml

81 Priebe S, Sandhu S, Dias S, et al. Good practice in health care for migrants: views and experiences of care professionals in 16 European countries, *BMC Public Health* 2011;11:1.

82 Levack WM. The role of qualitative metasynthesis in evidence-based physical therapy, *Physical Therapy Reviews* 2012;17:390-7.

83 Sandelowski M, Docherty S, Emden C. Focus on qualitative methods Qualitative metasynthesis: issues and techniques, *Research in nursing and health* 1997;20:365-72.

For peer review only

For peer review only - http://bmjopen.bmj.com/site/about/guidelines.xhtml

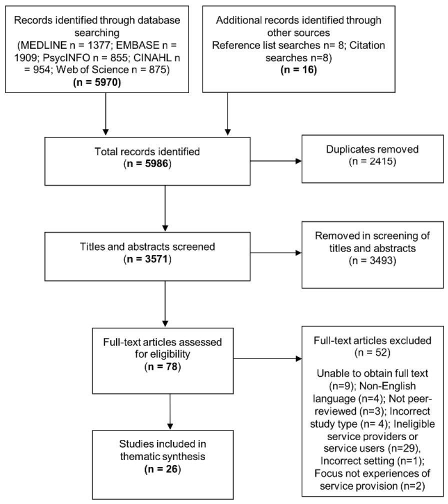

Figure 1 Flow diagram of systematic search and study selection

338x190mm (300 x 300 DPI)

review only

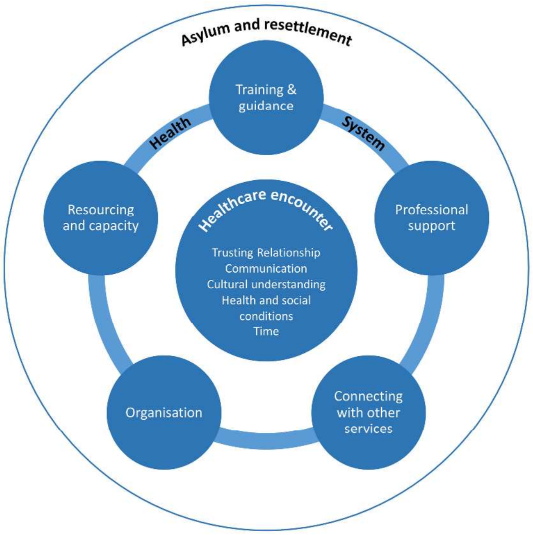

Figure 2: Model illustrating analytical constructs and descriptive themes

355x266mm (300 x 300 DPI)

For peer review only - http://bmjopen.bmj.com/site/about/guidelines.xhtml

Supplement 1: Database search strategy

MEDLINE	EMBASE	CINAHL	WEB OF SCIENCE	PSYCINFO
1. refugee/<br>2. asylum Seek$.mp.<br>3. refugee$.mp.	1. refugee/<br>2. asylum seeker/<br>3. asylum seek*.mp.<br>4. refugee*.mp.	1. MH “Refugees”<br>2. “refugee*”<br>3. ”asylum seek*”	1. refugee*<br>2. asylum seek*	1. exp refugees/<br>2. asylum seek*.mp.<br>3. refugee*.mp.
4. 1 or 2 or 3	5. 1 or 2 or 3 or 4	4. 1 or 2 or 3	3. 1 or 2	4. 1 or 2 or 3
5. exp Primary Healthcare/<br>6. exp health services/<br>7. exp health personnel/<br>8. nurs$.mp.<br>9. pharmacist$.mp.<br>10. health care.mp.<br>11. midwi$.mp.<br>12. general practi$.mp.<br>13. service provi$.mp.<br>14. care prov$.mp.<br>15. healthcare.mp.	6. exp primary health care/<br>7. exp health service/<br>8. exp health care personnel/<br>9. healthcare.mp.<br>10. health care.mp.<br>11. nurs*.mp.<br>12. pharmacist*.mp.<br>13. midwi*.mp.<br>14. general practi*.mp.<br>15. service prov*.mp.<br>16. care prov*.mp.	5. MH “Facilities Manpower and Services+”<br>6. MH “Health Personnel+”<br>7. “healthcare”<br>8. “health care”<br>9. “service prov*”<br>10. “care prov*”<br>11. “nurs*”<br>12. “pharmacist*”<br>13. “midwi*”<br>14. “general practi*”	4. healthcare<br>5. health care<br>6. service prov*<br>7. care prov*<br>8. nurs*<br>9. pharmacist*<br>10. midwi*<br>11. general practi*	5. exp Health Care Services/<br>6. exp primary health care/<br>7. exp Health Personnel/<br>8. health care.mp.<br>9. healthcare.mp.<br>10. care prov*.mp.<br>11. service prov*.mp.<br>12. nurs*.mp.<br>13. pharmacist*.mp.<br>14. midwi*.mp.<br>15. general practi*.mp.
16. 5 or 6 or 7 or 8 or 9 or 10 or 11 or 12 or 13 or 14 or 15	17. 6 or 7 or 8 or 9 or 10 or 11 or 12 or 13 or 14 or 15 or 16	15. 5 or 6 or 7 or 8 or 9 or 10 or 11 or 12 or 13 or 14	12. 4 or 5 or 6 or 7 or 8 or 9 or 10 or 11	16. 5 or 6 or 7 or 8 or 9 or 10 or 11 or 12 or 13 or 14 or 15
17. qualitative.mp.<br>18. qualitative research/<br>19. mixed method$.mp.<br>20. experienc$.mp.<br>21. perception$.mp.<br>22. attitude$.mp.<br>23. Perspective$.mp.<br>24. challenge$.mp.<br>25. barrier$.mp.<br>26. facilitator$.mp.	18. qualitative research/<br>19. qualitative.mp.<br>20. mixed method*.mp.<br>21. experienc*.mp.<br>22. perception*.mp.<br>23. attitude*.mp.<br>24. perspective*.mp.<br>25. challeng*.mp.<br>26. facilitator*.mp.<br>27. barrier*.mp.	16. MH “Qualitative Studies+”<br>17. “qualitative*”<br>18. “mixed method*”<br>19. “experienc*”<br>20. “perception*”<br>21. “attitude*”<br>22. “perspective*”<br>23. “challeng*”<br>24. “facilitator*”<br>25. “barrier*”	13. qualitative<br>14. mixed method*<br>15. experienc*<br>16. perception*<br>17. attitude*<br>18. perspective*<br>19. challeng*<br>20. facilitator*<br>21. barrier*	17. exp Qualitative Research/<br>18. qualitative.mp.<br>19. mixed method*.mp.<br>20. experienc*.mp.<br>21. perception*.mp.<br>22. attitude*.mp.<br>23. perspective*.mp.<br>24. challeng*.mp.<br>25. facilitator*.mp.<br>26. barrier*.mp.
27. 17 or 18 or 19 or 20 or 21 or 22 or 23 or 24 or 25 or 26	28. 18 or 19 or 20 or 21 or 22 or 23 or 24 or 25 or 26 or 27	26. 16 or 17 or 18 or 19 or 20 or 21 or 22 or 23 or 24 or 25	22. 13 or 14 or 15 or 16 or 17 or 18 or 19 or 20 or 21	27. 17 or 18 or 19 or 20 or 21 or 22 or 23 or 24 or 25 or 26
28. 4 and 16 and 27	29. 5 and 17 and 28	27. 4 and 15 and 26	23. 3 and 12 and 22	28. 4 and 16 and 27
**1377**	**1909**	**954**	**875**	**855**

**Total from database searches**	**5,970**

Robertshaw et al 2017

For peer review only

Supplement 2: Selection criteria

Study type:

Include	Exclude
Primary qualitative research studies	Theses/Dissertations
Interviews/focus groups	Opinion articles
Peer reviewed	Case studies
	Surveys (quantitative)
	Organisation reports
	Reviews

Primary health care professionals:

Include	Exclude
Nurses	Obstetricians
General practitioners	Psychologists
Midwives	Psychotherapists
Health visitors (nurse/midwives)	Physiotherapists
Pharmacists	Counsellors
	Social workers
	Managers
	Interpreters
	Volunteers
	Unqualified health professional (e.g student nurse)
	Unspecified staff within service providers

Health care service users:

Include	Exclude
Refugees	Migrants
Asylum seekers	Immigrants
Forced/Involuntary migrants	Undocumented migrant
Refugee claimant	Illegal immigrant

Setting of practice of health professionals:

Include	Exclude
Community	Asylum seeker detention centre
Community health centres	Hospitals- acute care
General practices	Specialist centres: referral from primary care
Community clinics	
Refugee/asylum centres	

For peer review only

Robertshaw et al 2017

High-income countries (World Bank classification 2015[1]):

Include:

Andorra
Antigua and Barbuda
Argentina
Aruba
Australia
Austria
Bahamas, The
Bahrain
Barbados
Belgium
Bermuda
Brunei Darussalam
Canada
Cayman Islands
Channel Islands
Chile
Croatia
Curaçao
Cyprus
Czech Republic
Denmark
Equatorial Guinea
Estonia
Faeroe Islands
Finland
France
French Polynesia
Germany
Greece
Greenland
Guam
Hong Kong SAR, China
Hungary
Iceland
Ireland
Isle of Man
Israel
Italy
Japan
Korea, Rep.
Kuwait
Latvia
Liechtenstein
Lithuania
Luxembourg
Macao SAR, China
Malta
Monaco
Netherlands
New Caledonia
New Zealand
Northern Mariana Islands
Norway
Oman
Poland
Portugal
Puerto Rico
Qatar
Russian Federation
San Marino
Saudi Arabia
Seychelles
Singapore
Sint Maarten (Dutch part)
Slovak Republic
Slovenia
Spain
St. Kitts and Nevis
St. Martin (French part)
Sweden
Switzerland
Taiwan, China
Trinidad and Tobago
Turks and Caicos Islands
United Arab Emirates
United Kingdom
United States
Uruguay
Venezuela, RB
Virgin Islands (U.S.)

Focus of study:

Include	Exclude
Experiences providing primary healthcare for refugees and asylum seekers	Experiences treating a specific condition common in refugees and asylum seekers, but no focus on healthcare interactions.<br>Experiences of a particular service or organisation for refugees and asylum seekers<br>HCP's perspectives on refugees and asylum seekers' experiences

[1] The World Bank, World Bank list of economies (July 2015) [Data file]. Retrieved from http://data.worldbank.org/about/country-and-lending-groups

For peer review only - http://bmjopen.bmj.com/site/about/guidelines.xhtml

Supplement 3: Data extraction of studies included in the thematic synthesis

Study Reference	Country of study	Study type	Total Participants in Study	Health professional practice setting	Service users	Data collection method	Analysis method	Study aims/purpose (Author's words)	Reported results (Themes/main headings)	Recommendations/applications (Author's words)
Begg H, Gill PS. Views of general practitioners towards refugees and asylum seekers: an interview study. Diversity Health Soc Care 2005 12;2(4):299-305 7p.	United Kingdom	Qualitative	17 general practitioners	General practice	Refugees and asylum seekers	Semi-structured interviews	Thematic framework (Ritchie and Spencer, 1993)	To identify some of the concerns of 17 general Practitioners working in an urban environment.	1.Political logistics and the asylum process<br>2 Community issues<br>3 Impact upon primary care<br>4 Resources and resource management<br>5 Training needs within primary care	> Guidelines and protocols for practice ...GPs would welcome those that might help them to deliver healthcare to refugees and asylum seekers.<br>>Primary care trusts need to liaise with local authorities and the Home Office to identify areas to which large numbers of asylum seekers are dispersed.
Bennett S, Scammell J. Midwives caring for asylum-seeking women: research findings. Pract Midwife 2014 Jan;17(1):9-12.	United Kingdom	Qualitative	10 midwives	Setting unclear, but includes community, rotational, specialist and delivery suite midwives.	Asylum seeking women	Semi-structured interviews	Thematic analysis (Bryman 2008)	The aim of this research was to gain an in depth analysis of the experiences of midwives and their understanding of the specific needs of asylum-seeking women. The findings would be used to inform education, practice and policy to enable more effective delivery of woman-centred care for this group locally.	1. Time<br>2. Communication	>Midwives deserve support in practice and enhanced education, and policy around asylum-seeking women would facilitate more effective, evidence-based care.<br>>It is essential that midwives (and other members of the multi-disciplinary team) have access to and training in the use of interpreting services.<br>>The additional time required to provide care to women seeking asylum should be factored into midwives' workloads.<br>>Education programmes to prepare/enhance knowledge and skills in caring for asylum seekers<br>>Web based resource with information about asylum seekers.
Burchill J. Safeguarding vulnerable families: work with refugees and asylum seekers. Community Practitioner 2011 Feb;84(2):23-26.	United Kingdom	Qualitative	14 health visitors	London borough	Refugees and asylum seekers	In-depth interviews	Thematic framework (Ritchie & Spencer, 1994)	Not clearly stated	1. Complexity of safeguarding-related needs<br>2. Sole support agent<br>3. Cultural challenges<br>4. Cycle of abuse<br>5. Disappearing from the system	> Increase awareness for effective commissioning of appropriate services for this group.<br>> Joint working may prevent the difficulties that health visitors face when working with vulnerable populations such as asylum seekers and refugees.<br>> Health visitors working with vulnerable populations need to explore opportunities to highlight concerns with their managers and commissioners.
Burchill J, Pevalin D. Barriers to effective practice for health visitors working with asylum seekers and refugees. Community Practitioner 2012 Jul;85(7):20-23.	United Kingdom	Qualitative	14 health visitors	London borough	Refugees and asylum seekers	In-depth interviews	Thematic framework (Ritchie & Spencer, 1994)	To determine the barriers to effective practice that health visitors when working with refugees and asylum seekers.	1.Ineffective engagement<br>2.Stretched resources	> Health professionals share innovative ways of working to in order to reduce the barriers experienced by refugees and asylum seekers.<br>> Increase awareness among primary health care staff of entitlement to health services for this particular client group.<br>> Commissioners should have an awareness of barriers to effective practice when deciding how to invest in services for vulnerable populations.
Burchill J, Pevalin DJ. Demonstrating cultural competence within health-visiting practice: working with refugee and asylum-seeking families. Diversity Equality Health Care 2014 06;11(2):151-159 9p.	United Kingdom	Qualitative	14 health visitors	London borough	Refugees and asylum seekers	In-depth interviews	Thematic framework (Ritchie & Spencer, 1994)	Explored the experiences of health visitors working with refugee and asylum-seeking families in central London, and assessed the dimensions of their cultural competency using Quickfall's model (Quickfall, 2004, 2010)	1.Institutional regard<br>2.Cultural awareness<br>3.Cultural sensitivity<br>4.Cultural knowledge<br>5.Cultural competence	> Health visitors need to be able to demonstrate cultural competence in their practice with refugee and asylum-seeking families.

For peer review only - http://bmjopen.bmj.com/site/about/guidelines.xhtml

Study Reference	Country of study	Study type	Total Participants in Study	Health professional practice setting	Service users	Data collection method	Analysis method	Study aims/purpose (Author's words)	Reported results (Themes/main headings)	Recommendations/applications (Author's words)
Carolan M, Cassar L. Pregnancy care for African refugee women in Australia: attendance at antenatal appointments. Evid Based Midwifery 2007 2007;5(2):54-58 5p.	Australia	Qualitative	2 midwives<br>10 African women*<br>1 community worker*<br>1 interpreter*<br>1 family and reproductive rights education program worker*	African women's clinic within a community health centre	African refugee women	Observational methods and Semi-structured interviews	Not explicitly stated, but think perhaps Thematic Analysis	To explore factors that facilitate or impede the uptake of antenatal care among African refugee women.	1.Staff attitudes<br>2.Availability of interpreters<br>3.Knowledge about the clinic at community level<br>4.Convenient location of the clinic	>Community midwifery clinics might offer a solution in terms of providing an acceptable and sensitive service to refugee African women. This familiar service would allow the women to meet the same carers on each visit, which would facilitate the development of trust.<br>>Opportunity for the clinic staff to tailor services to identified needs, such as the provision of interpreters in specific languages, liaison with medical and midwifery specialists with a knowledge of African disease and access to social and community workers.
Crowley P. The mental health needs of adult asylum seekers in Newcastle upon Tyne. Journal of Public Mental Health 2005;4(1):17-23.	United Kingdom	Mixed methods	10 general practitioners<br>67 asylum seekers (quantitative)*<br>? asylum seekers (qualitative)*<br>? managers*<br>? mental health service providers*<br>? housing support*<br>? agency staff*<br>? voluntary sector service providers*<br>? interpreters*	General practice and community	Asylum seekers	Interviews<br>Telephone interviews<br>Focus groups	Unspecified	To assess the mental health care needs of adult asylum seekers in Newcastle upon Tyne.	A. Quantitative<br>1. Demographic information<br>2. Mental illness prevalence in primary care<br>3. Mental illness prevalence in the general population<br>4. Mental health service use<br>B. Qualitative<br>1. Asylum seekers<br>2. Housing support workers and interpreters<br>3. Voluntary sector service providers<br>4. GP practices<br>5. Mental health service providers and managers<br>6. Regional and national agencies	> Increase opportunities for self-sufficiency; developing social support; developing peer groups; strengthening links with the host community; tackling racial harassment; improving economic well-being, and facilitating communication with families.<br>> Primary care practices need more education, training, support and resource to meet the needs of asylum seekers effectively, and to address the issue of hostility from other patients.<br>> There is a need both to improve mental health services and to strengthen social and other forms of support both within the communities to which asylum seekers belong and within host communities.<br>>In Newcastle, weaknesses in policy and practice in the mental health trust require attention in the light of the overall need to develop mental health services that best meet the need of the whole population.<br>> A greater level of sensitivity to the mental health needs of asylum seekers is required across the public sector, together with recognition of the major impact that experience in the host country has on their mental health and well-being.
Drennan VM, Joseph J. Health visiting and refugee families: issues in professional practice. J Adv Nurs 2005 01/15;49(2):155-163 9p.	United Kingdom	Qualitative	13 health visitors	2 inner London borough's	Refugees and asylum seekers	Semi-structured interviews	Framework method (Ritchie and Spencer 1994)	Describe health visitors' experiences working in Inner London and identifying and addressing the health needs of refugee woman in the first 3 months after the birth of a baby. Investigate health visitors' perceptions of effective and ineffective strategies in identifying and addressing health needs of these women. Investigate whether health visitors used a framework corresponding to Maslow's theory of a hierarchy of needs to prioritize their public health work.	1. Complexity of the relationship between health visitors and clients who are refugees.<br>2. Identification and prioritization of the health needs of the asylum seeking and refugee families.<br>3. Health visitors' perceptions of successful outcomes of their work.<br>4. Impact on health visitors of working with asylum seekers and refugees.	> There is a service and professional responsibility to ensure that health visiting and public health nursing practice is developed from the best evidence available and that collective knowledge and expertise are shared, rather than left for each practitioner to discover through trial and error.<br>> Both professional education providers and service providers need to pay attention to the specific health and social needs of asylum seeking women, who will unfortunately continue to arrive in the UK and other parts of the world.
Farley R, Askew D, Kay M. Caring for refugees in	Australia	Qualitative	20 general practitioners	General practice	Newly arrived refugees	Focus groups and	Thematic analysis	Explored the experiences of primary health care	1. Communication<br>2. Knowledge	> Increase range of resources available in languages other than English. Support English education for

For peer review only - http://bmjopen.bmj.com/site/about/guidelines.xhtml

Study Reference	Country of study	Study type	Total Participants in Study	Health professional practice setting	Service users	Data collection method	Analysis method	Study aims/purpose (Author's words)	Reported results (Themes/main headings)	Recommendations/applications (Author's words)
general practice: perspectives from the coalface. Australian Journal of Primary Health 2014;20(1):85-91.			5 practice Nurses<br>11 administrators*			semi-structured interviews		providers working with newly arrived refugees in Brisbane...focusing on the barriers and enablers they continue to experience in providing care to refugees.	3. Practice and health care systems	refugees. Support providers in understanding the linguistic backgrounds of their patients. Consider the importance of literacy in English education for refugees Improve availability and quality of visual resources. Raise awareness of refugees' limited literacy among providers. Increase interpreter service availability across all health care sectors (including allied health). Improve medical interpreter training. Provide information for providers regarding cultural differences in communication and the impact this can have on a consultation.<br>> Provide focussed education and training around important refugee health issues.<br>> Provide mental health training for providers, particularly in relation to caring for victims of past torture and trauma Improve supports available to providers working in this area, through access to trained psychologist and bicultural workers. Enhance psychologists' access to interpreters.<br>> Provide initial refugee health care in a specialised refugee health setting and ensure effective communication and support at the time of referral and beyond. Provide a forum for the exchange and transfer of experiences, information and resources between providers working in this area. Provide focussed education and training for providers, regarding the health care system as it pertains to refugee health care. Identify and adequately resource relevant support organisations. Consider methods to adequately remunerate providers (e.g. Medicare payments when interpreters are used). Provide case workers with appropriate training to assist in coordinating care.<br>> Provide education for refugees around health care within the Australian health care system.
Feldmann CT, Bensing JM, de Ruijter A. Worries are the mother of many diseases: General practitioners and refugees in the Netherlands on stress, being ill and prejudice. Patient Educ Couns Mar 2007;65(3):369-380	Netherlands	Qualitative	66 refugees*<br>24 general practitioners	General practice	Refugees (Afghan & Somali)	In-depth interviews	Not specified	To confront the views of refugee patients and general practitioners in the Netherlands, focusing on medically unexplained physical symptoms (MUPS).	1. Perspectives of refugees<br>-General narrative versus personal narratives<br>-Refugees' concepts of health and illness<br>-Causes of illness—mental worries<br>-Personal responsibility—strategies to stay healthy<br>-Expectations from doctors<br>-Refugees' problems with doctors<br>2. The general practitioners' perspective<br>-General practitioners on refugee problems<br>-How doctors deal with refugee problems<br>-Human interest strategy	>For a fruitful cooperation to develop, based on trust, GPs need to invest in the relationship with individual refugees, and avoid statements or actions based on stereotypes and prejudice. There is a heartening parallel between refugees' expectations and GPs' best practices.<br>> Direct observation, visual registration and later (qualitative) analysis of consultations between general practitioners and refugee patients, combined with eliciting refugees' expectations and level of trust before the consultation, and both the GPs' and the refugees' assessments afterwards, can help to raise awareness of possibilities for improvement in specific practices.<br>>Early investment in the relationship with new refugee patients may be crucial to establishing a basis of trust and dealing with unexplained physical symptoms effectively.<br>>Asking (refugee) patients about their situation and the way they are dealing with it, separate from the complaint that is being presented, helps to create an atmosphere of joint responsibility.

For peer review only - http://bmjopen.bmj.com/site/about/guidelines.xhtml

Study Reference	Country of study	Study type	Total Participants in Study	Health professional practice setting	Service users	Data collection method	Analysis method	Study aims/purpose (Author's words)	Reported results (Themes/main headings)	Recommendations/applications (Author's words)
									-Technical strategy<br>-Elements that occur in both 'human interest' and 'technical' strategies	>A physical complaint always deserves a thorough physical examination.<br>>The tendency to stereotype refugee patients may be a serious pitfall for practitioners.<br>>Critical reflection by practitioners is needed on strategies they employ for dealing with unexplained physical symptoms.<br>>Professional errors by medical practitioners have a long life circulating as part of the 'general narrative' in refugee communities, undermining trust. A more open climate when dealing with professional mistakes, especially towards the patients involved and their relatives, may help to address this phenomenon.
Furler J, Kokanovic R, Dowrick C, Newton D, Gunn J, May C. Managing depression among ethnic communities: A qualitative study. Annals of Family Medicine May-Jun 2010;8(3):231-236.	Australia	Qualitative	8 family physicians	Community health centre	Refugees with depression	Semi-structured interviews	Thematic analysis (Mays & Pope 1995)	Explores the complexities of this work [clinical care for depression] through a study of how family physicians experience working with different ethnic minority communities in recognizing, understanding, and caring for patients with depression.	1. Understanding and negotiating the problem of depression<br>2. Managing the depression<br>3.Working with the interpreter	>Highlight the need for more detailed observational research of clinical care for depression across a range of primary care settings and contexts.
Griffiths R, Emrys E, Lamb CF, Eagar S, Smith M. Operation Safe Haven: The needs of nurses caring for refugees. Int J Nurs Pract Jun 2003;9(3):183-190.	Australia	Qualitative	13 nurses<br>1 medical records clerk*<br>2 nursing managers	Refugee reception centre	Refugees	2 focus groups (13 nurses + 1 clerk), Semi-structured interviews (2 nurse managers)	Thematic analysis	To identify the skills, knowledge and support nurses require to provide holistic and competent care to refugee children and their families and the nature of support that is required to assist their transition back to mainstream health services.	1. Clinical skills and knowledge required by Safe Haven nursing staff.<br>2. Cultural competency skills<br>3. Trauma-sensitive care<br>4. Stressors impacting on Safe Haven nurses<br>5. Sources of support for Safe Haven nurses<br>6. Rewards<br>7. Return to work	>Counselling (for Nurses) should be provided by qualified, on-site counsellors with good understanding of trauma-related issues.<br>>Nursing workforce planners need to be able to employ appropriate numbers of permanent staff for extended disaster operations, avoiding the need for excessive work hours or the unsustainable practice of 'partial secondment', where nurses are expected to carry out disaster-type work and maintain their existing work responsibilities.<br>>Nursing workforce planners should undertake strategic recruitment during extended disaster operations, identifying appropriately skilled workers to form a stable workforce offering continuity of care.<br>>Disaster planners at the Area Health Service level should identify appropriate external agencies and designated health providers to assist with clinical management during extended operations, where nurses work with increased autonomy.
Jensen NK, Norredam M, Priebe S, Krasnik A. How do general practitioners experience providing care to refugees with mental health problems? A qualitative study from Denmark. BMC Family Practice 2013;14:17.	Denmark	Qualitative	9 general practitioners	Medical clinics with high proportion of immigrants	Refugees	Semi-structured interviews	Content analysis (Graneheim and Lundman 2004)	To qualitatively explore issues identified by general practitioners as important in their experiences of providing care for refugees with mental health problems.	1. Communication<br>2. Quality of care<br>3. Referral pathways<br>4. Understandings of disease and expectations of treatment.	>The findings from this study suggest that there is an increased need for general practitioners to be aware of potential traumas experienced by refugee patients, but also leave room for taking individual differences into account in the consultation. This could be attained by the development of conversational models for general practitioners including points to be aware of in the treatment of refugee patients. This may serve as a support in the health care management of refugee patients, but at the same time does not disregard the resources of individual refugee patients.

For peer review only - http://bmjopen.bmj.com/site/about/guidelines.xhtml

Study Reference	Country of study	Study type	Total Participants in Study	Health professional practice setting	Service users	Data collection method	Analysis method	Study aims/purpose (Author's words)	Reported results (Themes/main headings)	Recommendations/applications (Author's words)
Johnson D.R., Ziersch A.M., Burgess T. I don't think general practice should be the front line: Experiences of general practitioners working with refugees in South Australia. Australia and New Zealand Health Policy 2008;5(pagination):Arte Number: 20. ate of Pubaton: 08 Aug 2008.	Australia	Qualitative	12 general practitioners<br>3 medical directors of divisions of general practice*	General practice	Refugees	Semi-structured interviews	Template analysis	To document the existence and nature of challenges for GPs who do this work in SA.<br>To explore the ways in which these challenges could be reduced.<br>To discuss the policy implications of this in relation to optimising the initial health care for refugees.	1. Challenges for GPs<br>a)Refugee health issues<br>-GP knowledge of previous health assessments<br>- GP awareness of and experience managing health conditions unique to refugees<br>- The multiple and complex nature of refugee health conditions<br>b) GP-refugee interaction<br>- Issues related to culture<br>- Issues related to language<br>- Refugee knowledge of the Australian healthcare system<br>c) Structure of general practice<br>- GP workforce shortages<br>- Referral systems<br>- Remuneration<br>- Infrastructure supports to perform initial assessments<br>2. Challenges for Divisions assisting GPs<br>3. Ways GPs could be better supported<br>a) Providing GPs with more resources<br>b) Providing initial refugee health care via a specialist service	>Utilise a specialist service for refugees in refugees' resettlement period, which could provide initial health assessments and expertise in working with this population.<br>> If initial health assessments are provided by a specialist service, it is important that a clear, transparent and effective referral system to a nominated general practice is part of this process when initial health care needs have been met.
Kokanovic R, May C, Dowrick C, Furler J, Newton D, Gunn J. Negotiations of distress between East Timorese and Vietnamese refugees and their family doctors in Melbourne. Sociol Health Illn May 2010;32(4):511-527.	Australia	Qualitative	5 general practitioners<br>24 refugees from Vietnam and East Timor*	Community health centre	Refugees	In depth interviews	Thematic analysis	We explore a set of cultural boundaries across which depression is contested: between recent migrants to Australia from East Timor and Vietnam, and their white 'Anglo' family doctors. We are concerned with the ways that the experiences of migration and its aftermath are manifest in the lives of people from these ethnic groups; how their consequent distress is negotiated and contested in their interactions with family doctors; and how the	1. The journey and the arrival are important<br>2. Home and family: here and there<br>3.The naming of parts: manifestations of and bringing distress into the medical encounter<br>4. Illness Labels: naming distress	> Reinvestigate the way of conducting research on depression in a cross-cultural context.

For peer review only

For peer review only - http://bmjopen.bmj.com/site/about/guidelines.xhtml

Study Reference	Country of study	Study type	Total Participants in Study	Health professional practice setting	Service users	Data collection method	Analysis method	Study aims/purpose (Author's words)	Reported results (Themes/main headings)	Recommendations/applications (Author's words)
								resulting collisions affect the meaningfulness of the concept of depression.		
Kurth E, Jaeger FN, Zemp E, Tschudin S, Bischoff A. Reproductive health care for asylum-seeking women - a challenge for health professionals. BMC Public Health 2010;10:659.	Switzerland	Mixed-Methods. Quantitative element, based on patient files, explored frequencies of diagnoses and medical interventions. Qualitative element analysed data from asylum seekers patient notes and interviews with health professionals.	80 asylum seekers*<br>3 physicians<br>3 nurse/ midwife<br>1 psychologist*<br>3 interpreter*	Women's clinic	Female asylum seekers	Semi-structured Interviews with the 10 health professionals. Textual data was extracted from the 80 asylum seeker's patient files. The quantitative element extracted data from hospital electronic database and patient files.	Grounded theory methodology	The aim of the present study was to investigate the reproductive health care provided for women asylum seekers attending the Women's Clinic of the University Hospital in the city of Basel, Switzerland. To identify the health needs of asylum seekers attending the Women's Clinic and to investigate the health care they received in a Health maintenance organisation (HMO) specifically established for asylum seekers. Explored the perceptions of the health care professionals Involved about providing health care for this group in this setting.	1. Language and cultural barriers<br>2. Conflicting roles of physicians<br>Unclear how these themes were chosen from all of the data	> Specific training and support for health care providers.<br>> Training and support are needed not only because of the emotional challenges resulting from the situation, but because the patients do not only need medical care, but very often suffer from severe psychosocial problems arising from the stressful situation they are in.<br>>Attention should also be paid to stressors that could potentially affect health professionals and their work: the need for support and training of health care providers caring for vulnerable populations should be investigated further.<br>> The effect on health care providers of working in a restrictive HMO setting, where they do not only have to carry out their traditional clinical tasks but must also cope with increasing managerial responsibilities and financial restrictions, may also warrant further study.<br>> Language barriers can be overcome with the use of well-trained professional interpreters - both for the patients' sake and to avoid frustration in health care providers.
Lawrence J, Kearns R. Exploring the 'fit' between people and providers: refugee health needs and health care services in Mt Roskill, Auckland, New Zealand. Health & Social Care in the Community 2005 Sep;13(5):451-461.	New Zealand	Qualitative	5 community representatives*<br>9 refugee group representatives*<br>5 medical practitioners<br>1 manager*<br>1 administrator*	Community health centre	Refugees	Semi-structured Interviews	Thematic analysis	This paper reports on research that sought to reveal the barriers faced by refugees in accessing health services, and the challenges faced by providers in endeavouring to meet needs in an effective and culturally appropriate manner.	1. Population change within the Roskill area<br>2. Refugee perspectives on barriers to accessing health services<br>- Resettlement issues<br>-Differing cultural understanding of illnesses and health care systems<br>-Distrust of others<br>- Difficulties in communication<br>-Cost<br>-Physical access difficulties<br>3. Experiences of health practitioners in delivering health services to refugees	>The changing social landscape of larger Western cities...demands a greater attentiveness to the health needs of a population and the health services in place at a neighbourhood level<br>>In Mt Roskill...further adjustments in terms of funding, staffing, training and the style of patient/professional contact seem a necessary prerequisite for advancing health and social care in the community.<br>>There is clear need for funded health educators to provide a comprehensive orientation on such matters at the time of their registration at a service like HoP.<br>>Many of the delays and frustrations experienced by both the users and providers of services would be addressed by the funding of appropriate translation services.<br>>We advocate an enhanced commitment to developing cultural awareness through incorporating social-scientific perspectives to complement biomedical knowledge in medical education.<br>>To achieve this responsiveness [to community demographics], maintaining an elected board comprising both community and clinic representatives, as well as developing relationships with sympathetic researchers, can assist in bridging what otherwise might be a gulf between clinic and community.
Riggs E, Davis E, Gibbs L, Block K, Szwarc J,	Australia	Qualitative	87 refugee background	Maternal and child health (MCH)	Refugee background	Focus groups	Thematic analysis	This study aims to explore the utilisation	1. Facilitating access to MCH services.	> Provision of refugee focussed training for service providers and a strategically coordinated approach is

For peer review only - http://bmjopen.bmj.com/site/about/guidelines.xhtml

Study Reference	Country of study	Study type	Total Participants in Study	Health professional practice setting	Service users	Data collection method	Analysis method	Study aims/purpose (Author's words)	Reported results (Themes/main headings)	Recommendations/applications (Author's words)
Casey S, et al. Accessing maternal and child health services in Melbourne, Australia: Reflections from refugee families and service providers. BMC Health Serv Res 2012 01;12(1):117-117 1p.			mothers*<br>12 nurses<br>1 community worker*<br>1 community liaison*<br>5 bilingual workers*<br>3 community representatives*<br>2 managers of bilingual workers*	service	mothers	(refugees, nurses, bilingual workers, community worker, community liaison) and individual interviews (community representatives, managers of bilingual workers)		and experience of MCH services in Melbourne, Victoria for parents of refugee background from the perspective of users and providers.	2. Promoting continued engagement with the MCH service.<br>3. Language challenges.<br>4. What is working well and what could be done better?	likely to facilitate access, build rapport and ongoing engagement and retention to the service for families of refugee background.<br>> Innovative culturally competent strategies to organise individual MCH service appointments should be trialled and evaluated to develop a MCH system that promotes refugee maternal and child health.<br>> Trial a model where MCH nurses attend venues where refugees already gather to promote MCH services, provide information and build trust.<br>> The role played by bicultural workers should be recognised and utilised in a way that benefits clients and service providers.<br>> MCH services could proactively work in partnership with bilingual community workers to call clients directly to make appointments. Where these workers are not available, interpreters could also be utilised for this purpose.
Samarasinghe K, Fridlund B, Arvidsson B. Primary health care nurses' promotion of involuntary migrant families' health. Int Nurs Rev 2010;57(2):224-231.	Sweden	Qualitative	34 primary health care nurses	Various primary health care settings: maternity, child, school and community health care, and nurse-led clinics covering asthma, allergy, diabetes and hypertension	Involuntary migrants	Interviews	Contextual analysis (Phenomenography)	The aim of this study was to describe the promotion of health in involuntary migrant families in cultural transition as conceptualized by Swedish PHCNs.	1. Category I. An ethnocentric approach focusing on the physical health of the individual<br>2. Category II. An empathic approach focusing on the mental health of the individual in a family context<br>3. Category III. A holistic approach empowering the family to function well in everyday life	> In orientating families to cultural values of host country, teaching new cultural behaviours must be carried out in a respectful way so that the families do not feel subjected to forced assimilation.<br>> having family conversations with the entire family about the impact of acculturation on interpersonal relationships may be helpful in strengthening family relations.<br>> To enhance family health and family cohesion, nurses need to facilitate involuntary migrant families' cultural transition by empowering the family to be in control of acculturation.<br>>For nurses to enhance family health during cultural transition, adequate education encompassing the development of intercultural communication skills and cultural self-awareness must be available at both undergraduate as well as post-graduate level on a national basis.<br>>In clinical practice, the implementation of family-focused nursing incorporating supportive conversations about acculturation and adaptation will be useful.
Suurmond J, Rupp I, Seeleman C, Goosen S, Stronks K. The first contacts between healthcare providers and newly-arrived asylum seekers: A qualitative study about which issues need to be addressed. Public Health Jul 2013;127(7):668-673.	Netherlands	Qualitative	36 nurse practitioners<br>10 public health physicians	Asylum seeker centres	Newly arrived asylum seekers	Group interviews	Framework	To describe the tacit knowledge of Dutch healthcare providers about the care to newly arrived asylum seekers and to give insight into the specific issues that healthcare providers need to address in the first contacts with newly arrived asylum seekers.	1. Investigation of the current health condition of asylum seekers<br>2. Assessment of health risks<br>3. Providing information about the health care system<br>4.Health education	> In education and training this rough framework thus can be used as a means to reflect upon priorities in health care to asylum seekers as well as being aware of possible pitfalls, dilemmas and difficulties.<br>> Potential aspects of training: the need for good communication skills (including the skill to work with a professional interpreter) to deal with cultural differences and to deal with possible high expectations of asylum seekers.<br>>Training may help care providers reflect upon their own boundaries of their medical profession: for example, should they be the ones to assess mental health problems of asylum seekers or is it better to refer to another institution with more relevant competencies?<br>>Sufficient time is needed for a consultation when all four elements are included.

For peer review only

For peer review only - http://bmjopen.bmj.com/site/about/guidelines.xhtml

Study Reference	Country of study	Study type	Total Participants in Study	Health professional practice setting	Service users	Data collection method	Analysis method	Study aims/purpose (Author's words)	Reported results (Themes/main headings)	Recommendations/applications (Author's words)
										>Reference to other types of care, such as mental health care, need to be ascertained, before care providers assess asylum seekers' needs such as mental health needs.<br>>Different issues may be addressed by different professionals (for example, assessing mental health problems may be done by a psychologist, health education may be done by a health educator).
Suurmond J, Seeleman C, Rupp I, Goosen S, Stronks K. Cultural competence among nurse practitioners working with asylum seekers. Nurse Educ Today 2010 11;30(8):821-826 6p.	Netherlands	Qualitative	89 nurse practitioners for survey element. 36 nurse practitioners in group interviews.	Asylum seeker centres	Asylum seekers	Questionnaires and group interviews	Framework	We explored the cultural competences that nurse practitioners working with asylum seekers thought were important.	1. Training and education in cultural competence<br>2. Knowledge of the political and humanitarian situation in the country of origin<br>3. Knowledge of epidemiology and the manifestation of diseases in asylum seekers' countries of origin<br>4. Knowledge of the effects of refugeehood on health<br>5. Awareness of the juridical context in which asylum seekers live<br>6. Skills to develop a trustful relationship with an asylum seeker<br>7. Ability to ask delicate questions about traumatic events and personal problems.<br>8. Ability to explain what can be expected from health care<br>9. Improving cultural competence	> These results add more specific competences to the cultural competences that have been described in other studies.<br>> It is not merely education or training that helps nurse practitioners feel culturally competent. Equally significant is the concrete experience of working with asylum seekers. This suggests that 'learning in action' by way of adequate supervision, mutual peer supervision, and systematic feedback on the work floor may also be a key teaching instrument. Thus, experiential and didactic learning may be integrated in order to develop relevant cultural competences.<br>> Cultural competences should not be seen as a list of skills that are acquired and ticked off one at a time, resulting in a person who is culturally competent. Acquiring cultural competence is an ongoing process, driven by the practitioners' self-reflection.
Tellep TL, Chim M, Murphy S, Cureton VY. Great suffering, great compassion: A transcultural opportunity for school nurses caring for Cambodian refugee children. Journal of Transcultural Nursing Oct 2001;12(4):261-274.	United States	Qualitative	6 school nurses 2 Cambodian liaisons*	Schools	Refugees	Focus group	Not specified	To describe the nature and meaning of school nurses' and Cambodian liaisons' experiences of caring for Cambodian refugee children and families and to explore whether those meanings validated Dobson's (1989) conceptual framework of transcultural health visiting.	1. Transcultural health-visiting education<br>2. Intracultural reciprocity<br>3. Transcultural reciprocity<br>4. Goal of maximising health and wellbeing: Letting go of one's own views<br>5. Multifaceted roles of Cambodian liaisons: We want to help them in any way<br>6. School and home: "Caught in the middle"<br>7. Intergenerational conflict: "It's hard for the kids"<br>8. The Cambodian	>Awareness of transcultural reciprocity and the importance of establishing trust may help guide other nurses in the development of meaningful relationships with Cambodian refugee children and families.<br>> Transcultural nursing care should be incorporated into all stages of the nursing process when caring for Cambodians.<br>> In partnership with the Cambodian community, interventions that target Cambodian refugee children with direct services, as well as indirect services through support of their families, are needed.<br>>Collaboration with others outside the school setting is vital to creating a cross-cultural team approach of coordinated and comprehensive service to Cambodian refugee children and families.<br>>Individualize care based on family's background and refugee history.<br>>Keep reaching out; trust takes time.<br>>Take a slow, friendly, no direct spiralling approach.

For peer review only - http://bmjopen.bmj.com/site/about/guidelines.xhtml

Study Reference	Country of study	Study type	Total Participants in Study	Health professional practice setting	Service users	Data collection method	Analysis method	Study aims/purpose (Author's words)	Reported results (Themes/main headings)	Recommendations/applications (Author's words)
									refugee experience: "Left for dead"<br>9. Spiritual healing: "It lifts your spirits"<br>10. Cultural strengths: Carried across the ocean"	>Gently probe.<br>>Suspend assumptions and worldview.<br>>Look beyond the behavior to understand the underlying dynamic.<br>>Support cultural traditions and share your interest<br>>Elicit explanatory models for illness.<br>>Incorporate spiritual healing practices and the temple into delivery of health services.<br>>Encourage and mentor Cambodian role models.<br>>Provide health education: family planning, nutrition, safety, and routine check-ups.<br>>Assist with access to care.<br>>Provide support to parents and elders.<br>>Assess refugee risk factors as part of special education process.<br>>Monitor medications.
Tobin C.L., Murphy-Lawless J. Irish midwives' experiences of providing maternity care to non-Irish women seeking asylum. International Journal of Women's Health 2014 31 Jan 2014;6(1):159-169.	Ireland	Qualitative	10 midwives	Maternity hospitals	Female asylum seekers	In-depth unstructured interviews	Content analysis	To explore midwives' perceptions and experiences of providing care to women in the asylum process and to gain insight into how midwives can be equipped and supported to provide more effective care to this group in the future.	1. Barriers to communication<br>2. Understanding cultural difference<br>3. Challenges of caring for women who were unbooked<br>4. The emotional cost of caring,<br>5. Structural barriers to effective care.	>For women in the asylum process, having access to dedicated community-based services would begin to address the problems of access, late booking, and development of midwife/client relationships which in turn would help to decrease fear and anxiety for both the women themselves and the midwives who care for them.<br>>Cultural competency training: When considering how best to educate midwives to provide culturally competent care, the most important focus should be on using a framework of cultural humility.<br>> There is an urgent need for increased clinical support for midwives who care for traumatized women.<br>>Access to continuing education is also essential, along with debriefing and clinical supervision in order to maintain providers' own health and well-being.<br>> Trained interpreter service should be embedded within hospitals.<br>>dedicated community-based services that provide the possibility of continuity of care, make access to care easier for women, and provide the possibility of good midwife/client relationships and trust building.<br>> Revision of the government policy of forced dispersal for women in the asylum process who are pregnant or in the early postpartum period is urgently needed.
Twohig PL, Burge F, MacLachlan R. Pod people. Response of family physicians and family practice nurses to Kosovar refugees in Greenwood, NS. Canadian Family Physician 2000 Nov;46:2220-2225.	Canada	Qualitative	6 family practice nurses<br>10 family physicians	Clinic in refugee processing centre	Refugees	Semi-structured interviews	Textual analysis	To explore roles of family physicians and family practice nurses who provided care to Kosovar refugees at Greenwood, NS.	1. Clinical encounter<br>2. Expectation and experience<br>3. Roles and team functioning<br>4. Response	> Future responses to emergency situations might benefit from clearer descriptions of individual roles within the team.
Yelland J, Riggs E, Wahidi S, Fouladi F, Casey S, Szwarc J, et al. How do Australian	Australia	Mixed Methods. Interviews conducted	30 Afghan parents*<br>10 midwives<br>5 medical	Mixed Methods. Interviews conducted with Afghan parents	Refugee background	Interviews and focus groups	Thematic analysis	(1) investigate Afghan women and men's experience of the way that health professionals	1. Language services in the context of care<br>2. Women and men's experience of being	>Our findings support calls for standardised procedures to improve identification of people of refugee background in clinical settings.<br>>Building an understanding of the refugee experience,

Study Reference	Country of study	Study type	Total Participants in Study	Health professional practice setting	Service users	Data collection method	Analysis method	Study aims/purpose (Author's words)	Reported results (Themes/main headings)	Recommendations/applications (Author's words)
maternity and early childhood health services identify and respond to the settlement experience and social context of refugee background families?. BMC Pregnancy & Childbirth 2014;14:348.		with Afghan parents contained a quantitative element. No reported quantitative element in interviews with health professionals.	practitioners* 19 Community based health professionals*	contained a quantitative element. No reported quantitative element in interviews with health professionals.				approach inquiry about social factors affecting families having a baby in a new country, and (2) investigate how health professionals identify and respond to the settlement experience and social context of families of refugee background.	asked about social health issues 3. Identifying and responding to social health issues: the experience of health professionals	what health care providers need to be mindful of in providing care to families of refugee background, and knowledge of services for referral, is likely to go some way in building workforce capacity to assess and respond to the social circumstances of refugees. >Interactive training opportunities incorporating knowledge of the refugee and asylum seeker experience and ways of working with these families is a strategy to enhance health professionals understanding and skills. >Any attempts to improve the responsiveness of health services to the needs of families of refugee background need to consider innovative ways to work within system constraints.
Yelland J, Riggs E, Szwarc J, Casey S, Duell-Piening P, Chesters D, et al. Compromised communication: a qualitative study exploring Afghan families and health professionals' experience of interpreting support in Australian maternity care. BMJ Qual Saf 2016 Apr;25(4):e1-2014-003837. Epub 2015 Jun 18	Australia	Qualitative	30 Afghan parents* 10 midwives 5 medical practitioners* 19 Community based health professionals*	Various maternity care services	Refugee background	Interviews and focus groups	Thematic analysis	(1) describe Afghan women's and men's experiences of language support during pregnancy check-ups, labour and birth; (2) explore health professionals' experiences of communicating with Afghan and other refugee clients with low English proficiency; and (3) consider implications for health services and health policy.	1. The use of accredited interpreters in maternity care 2. Family members interpreting during pregnancy, labour and birth	> Improving identification of language needs at point of entry into healthcare, developing innovative ways to engage interpreters as integral members of multidisciplinary healthcare teams and building health professionals' capacity to respond to language needs, especially when clients' have experienced trauma that is likely to impact on their capacity to engage with healthcare, are critical to reducing social inequalities in maternal and child health outcomes for refugee and other migrant populations. >Potential 'solutions' in the context of maternity care include community and language-specific group pregnancy care sessions combining antenatal check-ups with information and support provided by a multidisciplinary team of health professionals including an accredited interpreter.

* These participants are not within the study definition of primary health care professionals and therefore their data have not been included in the thematic synthesis.

For peer review only

Supplement 4: Quality assessment of studies included in the thematic synthesis

Study reference*	Aims & methods	Research design	Sampling†	Data collection	Reflexivity	Ethical issues	Data analysis†	Discussion of findings	Value
Begg H, Gill PS. Views of general practitioners towards refugees and asylum seekers: an interview study. Diversity Health Soc Care 2005 12;2(4):299-305 7p.	Research aims not clearly articulated. Importance and relevance considered. Qualitative methodology is appropriate to capture General practitioner's views.	Use of qualitative design not explicitly justified.	17 general practitioners. "Purposeful sampling was used to recruit GP's, with more or less than 10% of the area population from the black and minority ethnic communities" and areas cross checked with the Refugee council. Age and ethnicity were not controlled for. One hundred GPs were randomly selected from the target locations using computer generated numbers, and approached via post and a follow-up phone call. Of these, 20 GPs volunteered to participate but 17 were actually interviewed as three opted out at the last minute due to work priorities.	Semi-structured Interviews conducted at GP practices by the author. No justification given for methods or setting of data collection. A previously piloted and refined topic guide was utilised with topics listed No detail on how data was recorded. Data collection terminated upon saturation of emergent themes.	Researcher considered the potential influence of her age (medical student), sex (female) ethnicity (as from ethnic minority) in the openness of participants. In addition, recruitment bias was considered (people with stronger opinions more likely to respond). Also discussed the reasons for volunteers opting out not being related to study aims.	No detail on how the study was explained to participants. Written consent was obtained prior to the commencement of each interview, and confidentiality maintained throughout. Lacking details on how researchers handled issues raised for participants by the study. Ethical approval was obtained from North West Multisite Research Ethics Committee.	A thematic framework analysis was conducted. "Data collection and analysis proceeded simultaneously, incorporating emergent themes into subsequent interviews. Emergent themes were compared by HB and PG independently before agreement and refinement of the themes." Did not contain a description of how data presented was selected. Sufficient data were presented to support the findings. Contradictory data were taken into account Researcher highlights the use of multiple coding to reduce bias in the analysis along with respondent validation.	The findings were explicit and clearly discussed. As mentioned in Q1, the research question is not clearly defined. The findings are discussed in the context of the wider literature. Credibility enhanced by respondent validation and multiple analysts.	Briefly considered the value of the study and contribution to research (highlighted some important issues surrounding the delivery of care to refugees and asylum seekers) identified areas for further research (lack of time, support, education, training and, financial resources) Acknowledges the limitations in generalisability as conducted in one metropolitan area.
Bennett S, Scammell J. Midwives caring for asylum-seeking women: research findings. Pract Midwife 2014 Jan;17(1):9-12.	Aims clearly stated with explanation of how the findings would be used to inform policy, education and practice. Qualitative methodology is appropriate for exploration of midwives experiences of caring for asylum seekers.	Use of qualitative design not explicitly justified.	10 midwives. The study was targeted at qualified midwives who had practised for a minimum of one year and had some experience of working with asylum-seeking women. Midwives were recruited via an email sent by the Head of midwifery; 10 volunteered to participate. All those who volunteered were included in the sample. Not clear whether there was a process to check eligibility of volunteers. Non-participation was not discussed.	Semi-structured interviews. Lacking details about the setting and who conducted the interviews. No justification given for methods or setting of data collection. No explicit reporting of how the interviews were conducted and the areas of enquiry. Interviews were audio recorded and transcribed. Data saturation not discussed.	The researcher's role and potential bias in the formulation of questions or data collection was not discussed. An 'audit trail' was kept, capturing influences, events, actions and decisions taken during the conduct of the study.	All participants were provided with information about the study and gave written consent. States that all the participants were volunteers and free to withdraw at any time. Confidentiality was not discussed. Lacking details on how researchers handled issues raised for participants by the study. Ethical approval was gained from the trust and NHS National Research Ethics Service.	"A thematic analysis was used to capture emerging patterns of data. These were reviewed and grouped into two overarching themes and four interconnected sub-themes. Rigour was maintained through a systematic process of enquiry, sampling and analysis." No indication of involvement of multiple researchers in the analysis. Sufficient data were presented to support the findings. Contradictory data not discussed. No examination of researcher's role, potential bias and influence during the analysis and in presentation of the data. Only some of the themes from the analysis are reported in this paper.	The findings were explicit and clearly discussed in relation to the original research question and within the context of the wider research literature. No discussion of the credibility of the research and did not report whether multiple researchers were involved in coding transcripts or interpretation of findings.	Considered the value of the study and the contribution of the research. Did not make suggestions for future research. Considered the generalisability of the findings. Provided a number of recommendations for practice, education and policy.
Burchill J. Safeguarding vulnerable families: work with refugees and asylum seekers.	No clear statement of research aims. Importance and relevance of	Use of qualitative design not explicitly	14 health visitors. Purposive sampling was used in which participants were selected for their	In-depth interviews were conducted at multiple health centres across the borough (number not	Author acknowledges that there may have been bias related to	Research aims were explained at a professional meeting of health	A thematic framework method was utilised that involved a constant comparative approach in which codes and transcripts	The findings were explicit and clearly discussed in relation to the original research	Considered the value of the study in raising awareness of commissioners to

For peer review only - http://bmjopen.bmj.com/site/about/guidelines.xhtml

Study reference*	Aims & methods	Research design	Sampling†	Data collection	Reflexivity	Ethical issues	Data analysis†	Discussion of findings	Value
Community Practitioner 2011 Feb;84(2):23-26.	research adequately stated. Qualitative methodology is appropriate for understanding health visitor's experiences of working with refugees and asylum seekers.	justified.	ability to contribute to the data. Recruitment was conducted by approaching potential participants through a presentation at a professional meeting. Sample was approximately 1/3 of all health visitors in the borough. Participants required to have worked for 2yrs as would be highly likely to have worked with refugees and asylum seekers. No discussion about the reasons why some health visitors chose not to participate.	specified), but unclear who conducted the interviews. No justification given for methods or setting of data collection. A topic guide used that had been developed from a literature review. Participants were asked primarily to describe their experiences of working with refugees and asylum seekers and what problems/difficulties they faced. Method of recording interview not described but states that interviews were transcribed. Data saturation not discussed.	the fact that he worked in the same workplace (colleagues) as the participants. Participants may not have been as open or willing to tell the truth in interviews.	visitors. Lacking discussion about how consent was gained, confidentiality maintained and how issues raised by the study were handled by researchers. Approval to proceed with the study was granted by the Primary Care Trust research and development team and the Local Research Ethics Committee.	were constantly reassessed and re-interpreted. Themes identified were compared across the data and interpretations discussed with external researchers. No reported duplicate coding. Quotations were chosen to illustrate the particular issues described Sufficient data were presented to support the findings. Contradictory findings were not presented. No examination of researcher's role, potential bias and influence during the analysis and in presentation of the data.	question. Limited discussion of findings in the context of the wider literature. No explicit discussion of the credibility of the research, but methods report that interpretation was discussed with external researchers.	provide appropriate services for refugees and asylum seekers. No further research areas suggested. No explicit discussion of transferability to other populations but suggests the findings will be useful for commissioners in other settings and that the study adds to literature that can inform policy and practice.
Burchill J, Pevalin D. Barriers to effective practice for health visitors working with asylum seekers and refugees. Community Practitioner 2012 Jul;85(7):20-23.	Research aims clearly stated. Importance and relevance were articulated. Qualitative methodology is appropriate for understanding barriers to effective practice for health visitors working with refugees and asylum seekers.	Use of qualitative design not explicitly justified.	14 health visitors. Purposive sampling was used in which participants were selected for their ability to contribute to the data. Recruitment was conducted by approaching potential participants through a presentation at a professional meeting. Participants required to have worked for 2yrs as would be highly likely to have worked with refugees and asylum seekers. No discussion about the reasons why some health visitors chose not to participate.	In-depth interviews were conducted at multiple health centres across the borough in which the participants worked (Number of centres not specified). Unclear who conducted the interviews. No justification given for methods or setting of data collection. A topic guide used that had been developed from a literature review and consisted of a number of broad statements that would help guide the interview. The interviews were taped and transcribed verbatim. Data saturation not discussed.	No critical examination of the researcher's role, potential bias and influence in research question formulation or data collection.	Research aims were explained at a professional meeting of health visitors. Lacking discussion about how consent was gained, confidentiality maintained and how issues raised by the study were handled by researchers. The Primary Care Trust and the Local Research and Ethics Committee granted approval for this study.	A framework method was used that involved a constant comparative approach in which the codes were continually reassessed and interpreted. The themes that were identified were compared across the data and discussed with external researchers. Quotations were chosen to illustrate the particular issues described. Sufficient data were presented to support the findings. Contradictory data were not presented. No examination of researcher's role, potential bias and influence during the analysis and in presentation of the data.	The findings were explicit and clearly discussed in relation to the original research question and within the context of the wider research literature. No discussion of the credibility of the research.	Discusses the contribution of the study in increasing awareness in primary health care staff of health service entitlements of refugees and asylum seekers. Also raises awareness for commissioners of barriers to effective services when deciding how to invest in appropriate services.
Burchill J, Pevalin DJ. Demonstrating cultural competence within health-visiting practice: working with refugee and asylum-seeking families. Diversity Equality Health Care 2014 06;11(2):151-159 9p.	The aims of the research clearly stated The importance and relevance of the research were articulated Qualitative methodology is appropriate to explore health visitor's experiences of working with	Use of qualitative design not explicitly justified. Authors describe the purpose and key features of the Framework approach that they have	14 health visitors. A presentation was given at the health visitors' main professional meeting with details of the study and an invitation to participate. Participants had to be qualified health visitors and worked in the borough for over 2 years - ensuring that they had enough experience. Sample size was 14/42 health visitors	In-depth interviews were conducted at multiple health centres across the borough in which the participants worked (Number of centres not specified). Unclear who conducted the interviews. A topic guide used that had been developed from a literature review and consisted of 10 broad open-ended	No critical examination of the researcher's role, potential bias and influence in research question formulation or data collection.	Potential participants approached at a professional meeting of health visitors. All confirmed participants were sent an information letter and consent form to be signed before	Framework analysis. "Each interview was first transcribed and then analysed using Framework. This involved a constant comparative approach throughout. The themes that were identified were compared across the data, and interpretations were discussed between the interviewer (JB) and external researchers consisting of an academic supervisor and a doctoral	The findings were explicit and discussed in relation to the research question. Findings not discussed in the context of the wider literature. No explicit discussion of the credibility of the research, but methods report that interpretation was discussed with	The author discusses the contribution of the study to existing knowledge. Concludes that aspects of cultural competence are lacking, but are being addressed at the local level. Identifies the need for research into models of cultural competence in a variety of primary

For peer review only - http://bmjopen.bmj.com/site/about/guidelines.xhtml

Study reference*	Aims & methods	Research design	Sampling†	Data collection	Reflexivity	Ethical issues	Data analysis†	Discussion of findings	Value
	refugees and asylum seekers.	chosen for the analysis.	working in the borough.<br>No discussion about the reasons why some health visitors chose not to participate.	statements. The topic guide was given to participants prior to the interview.<br>No justification given for methods or setting of data collection.<br>A tape recorder was used to record the interview, which was transcribed for the analysis.<br>Data saturation not discussed.		participation in the study. Lacking discussion of how confidentiality was maintained. "Each participant was offered debriefing at the end of the interview session to discuss any issues that might have arisen, particularly if any difficult experiences were referred to."<br>Ethical approval was granted by the local NHS Research Ethics Committee, and research governance permission was gained from the Primary Care Trust Research and Development Team.	student."<br>Sufficient data are presented to support the findings.<br>Contradictory data were taken into account.<br>No examination of researchers own role, potential bias and influence during the analysis and in presentation of the data.	external researchers.	care settings.<br>Discusses the generalisability of the results and highlights the limitations of the model used for this research study for other health care settings.
Carolan M, Cassar L. Pregnancy care for African refugee women in Australia: attendance at antenatal appointments. EVID BASED MIDWIFERY 2007 2007;5(2):54-58 5p.	The aims of the research clearly stated.<br>The importance and relevance of the research were articulated.<br>A qualitative methodology is appropriate for understanding factors that facilitate or impede uptake of antenatal care among refugee communities.	Researchers justified their choice of study methods.<br>The use of observational methods before the semi structured interviews could help the researcher gain cultural understandin g and build trust with the participants.	10 African women, 2 midwives, 1 family reproductive rights education program worker, 1 interpreter.<br>African women: Recruitment was facilitated by the midwife, who asked women attending the clinic if they were interested in the study. Those indicating an interest were approached by the researcher and the nature of the study, time requirements and study purpose were explained. Women who were still interested were invited to participate.<br>No discussion about the reasons why some people chose not to participate<br>Clinic staff:<br>No description of how the clinic staff were selected for interview.<br>No explanation as to why this clinic was an appropriate place to	Data were collected in two phases. Phase 1 was 40 hours of observation at the women's clinic by a researcher. Phase 2 employed semi-structured interviews with staff and refugee women.<br>Setting of data collection was African Women's Clinic. Unclear who conducted the interviews.<br>No justification given for methods or setting of data collection.<br>Areas of enquiry in the interviews are described.<br>Researchers modified the questions asked in the interviews with attending women when it became apparent they did not understand questions.<br>Field notes were used to record observation element. Specific method of data recording during interviews not	No critical examination of the researcher's role, potential bias and influence in research question formulation or data collection.	Potential participants were approached by the researcher, who explained the nature and purpose of the research and the time commitment.<br>Participant's names were changed in the reporting of the study, but not clear whether this was explained to the participants.<br>No discussion of informed consent or how researchers handled issues raised by the study for participants.<br>The project was approved by university and hospital ethics committees.	Exact method used for data analysis not specified. Brief description of analysis process. "data analysis then proceeded through the following stages: Organising the data; Immersion in the data; Generating categories and themes; Coding the data; Offering interpretations; Seeking alternative explanations. Notes of analytical understandings and decisions were made throughout the process.<br>Trustworthiness of findings was enhanced by asking two academic colleagues to independently generate a theme list."<br>No explanation of how the data presented were selected from original sample.<br>Sufficient data are presented to support the findings.<br>Contradictory data are taken into account in the findings.<br>No examination of researcher's role, potential bias and influence during the analysis and in presentation of the data.	The findings were explicit, discussed with reference to the research question and set within the context of the wider literature.<br>The authors state that the trustworthiness of the findings are enhanced by asking two academic colleagues to independently generate a theme list during the analysis.	Authors suggest that community midwifery clinics might offer a solution for providing acceptable and sensitive services to refugee African women.<br>Findings considered in relation to relevant research-based literature.<br>No further research areas are suggested.<br>Transferability not discussed, but implied that similar healthcare services could be effective in other settings with refugee women.

For peer review only - http://bmjopen.bmj.com/site/about/guidelines.xhtml

Study reference*	Aims & methods	Research design	Sampling†	Data collection	Reflexivity	Ethical issues	Data analysis†	Discussion of findings	Value
			sample.<br>Non-participation not discussed	stated but transcription is mentioned.<br>Data saturation not discussed.					
Crowley P. The mental health needs of adult asylum seekers in Newcastle upon Tyne. Journal of Public Mental Health 2005;4(1):17-23.	Aims of the study were clearly stated and its importance and relevance articulated.<br>The qualitative element of this study was an appropriate methodology to explore perceptions of the causes of mental ill health among asylum seekers and investigate issues in delivering services to this group.	The use of qualitative methodology was not explicitly justified, but the purpose of interviews and focus groups was explained.	10 general practitioners and unspecified numbers of other participants (asylum seekers, managers, mental health service providers, housing support, agency staff, voluntary sector service providers, Interpreters)<br>Exact numbers of participants not reported.<br>No details given about how participants were selected for focus groups or interviews.<br>No justification given for the choice of these participants	Interviews, telephone interviews and focus groups were used to collect qualitative data, but no details about the interviewer(s). Lacking details of the setting of data collection, but some participants were interviewed by telephone.<br>Researcher justifies the use of some of the focus groups and interviews, but not the setting of data collection.<br>No details about how the interviews were conducted.<br>No details about how data were recorded during the interviews/focus groups.<br>No discussion of data saturation.	The researcher's role and potential bias in the formulation of questions or data collection was not discussed.	No details given about how the research was explained to participants.<br>No discussion of informed consent, confidentiality, or how issues raised in the course of study were handled by researchers.<br>Approval from an ethics committee is not reported.	No description given of the analysis process or whether multiple researchers were involved in the analysis.<br>Not clear how findings were derived from the data<br>Insufficient data are presented to support the findings.<br>Contradictory data were not taken into account.<br>No examination of researcher's role, potential bias and influence during the analysis and in presentation of the data.	The findings are explicit and discussed in relation to the research question<br>The findings are discussed in the context of the wider literature.<br>The credibility of the findings are not discussed	The author discusses the contribution of the study to existing knowledge, practice and policy.<br>No identification of new areas for research.<br>No discussion of whether the findings can be transferred to other populations.
Drennan VM, Joseph J. Health visiting and refugee families: issues in professional practice. J Adv Nurs 2005 01/15;49(2):155-163 9p.	The aims of the research clearly stated.<br>The importance and relevance of the research were articulated.<br>A qualitative methodology is appropriate to understand the perceptions of health visitors working with refugees and asylum seekers.	Authors had formulated a hypothesis that health visitors framed their work with refugee and asylum seeking women using Maslow's hierarchy of need. The study was undertaken to explore this hypothesis.<br>No justification of the specific qualitative methods employed.	13 health visitors.<br>The participants were recruited by purposive sampling. Health visitors who identified themselves as having a significant number of refugees and asylum seekers on their caseloads and had worked in inner London for more than 5 years and were currently working with refugees and asylum seekers.<br>No discussion about whether some people chose not to participate and their reasons.	Data were collected through semi-structured interviews, conducted at the health visitor's places of work. Unclear who conducted the interviews No justification given for methods or setting of data collection.<br>"Broad, open ended questions were used in the interview, inviting informants to be discursive and reflective in recounting their experiences.". Areas of enquiry in the interviews are described.<br>Interviews lasted 45min-1hr.<br>Interviews were tape-recorded and subsequently transcribed.<br>Data saturation was not discussed.	No critical examination of the researcher's role, potential bias and influence in research question formulation or data collection.	"Participants…given a full information sheet about the purpose, methods and use of the study". "Formal written consent was obtained and participants were assured that their data would be anonymized and deleted after transcription. "Participants were sent draft copies of the report to demonstrate that anonymity had been preserved".<br>Lacking details on how researchers handled issues raised for participants by the study.<br>Ethical approval was obtained from	Framework method was used to analyse data. "The theoretical issues identified in the literature were used to devise the coding framework. The interviewer and second author independently coded the transcripts against the framework; using word processing and spreadsheet functions software. Additional codes were assigned as the data suggested new themes and issues. A small number of discrepancies in coding between the two analyses were resolved through subsequent discussion. The coded material was then analysed for: (a) Commonalities between informants, (b) conflicting perceptions between informants and (c) evidence to support or disprove the use of a hierarchy of needs in framing practice."<br>Sufficient data are presented to support the findings.<br>Contradictory data are taken into account.<br>No examination of researcher's	The findings were explicit and discussed with reference to the research question.<br>Minimal discussion of the findings in relation to the wider literature.<br>The credibility of the research is not explicitly discussed, but the two authors independently coded transcripts against the framework with discrepancies resolved through discussion. In addition, participants were sent draft copies of the report for comment.	Briefly considers the value of the study. The author acknowledges that the single geographical setting and small sample size limit the conclusions.<br>The contribution of the study to existing knowledge and understanding is discussed.<br>Identified one possible avenue for further research - whether prioritization of children's needs over mothers could be another issue related to Maslow's pyramid.<br>They suggest that although the study was UK based, the issues raised in the study will likely resonate for public health nurses working in other countries.

Study reference*	Aims & methods	Research design	Sampling†	Data collection	Reflexivity	Ethical issues	Data analysis†	Discussion of findings	Value
						the local Research Ethics Committee.	role, potential bias and influence during the analysis and in presentation of the data.		
Farley R, Askew D, Kay M. Caring for refugees in general practice: perspectives from the coalface. Australian Journal of Primary Health 2014;20(1):85-91.	The aims of the research clearly stated.<br>The importance and relevance of the research were articulated.<br>Qualitative methodology is appropriate for exploring the experiences of primary health care providers working with refugees.	Use of qualitative design not explicitly justified.	20 general practitioners, 5 practice nurses, 11 administrators.<br>Researchers explain how the participants were selected. 6 general practices were purposively selected on the basis that they had received newly arrived refugees in the past 6 months. Purposive sampling ensured that participating practices had experience of caring for refugees.<br>Practices were approached by a researcher to discuss involvement in the project, which was followed up by a phone call to clarify involvement.<br>No discussion of the proportion of practice staff that agreed to participate in the research or any reasons for non-participation.	5 Focus groups and 4 semi-structured interviews were used.<br>The exact setting for data collection is not clear, but occurred during staff lunch breaks. RF facilitated the focus groups and conducted the semi-structured interviews.<br>Authors justified the use of some semi-structured interviews as a way of overcoming time constraints for some participants and for testing whether focus groups were effective in surfacing the key themes. The setting was not justified.<br>A standard introduction and interview schedule informed by the literature was used to stimulate conversation and discussion, but unclear whether this was for the focus groups, interviews or both. Brief description of the types of questions used. Authors report modification of methods in the study. Semi-structured interviews were used when time constraints prevented a focus group occurring and when a participant missed a focus group.<br>Focus groups and interviews were audio recorded and transcribed.<br>Data saturation is discussed.	It was acknowledged that personal relationships and power differentials in the workplace may have impacted on individual's freedom to express opinions in the focus groups.<br>The authors were aware of this potential and took steps to minimise this. (offering opportunity to provide confidential feedback).<br>Both researchers were working in refugee health and were aware of potential for influencing data collection and interpretation. To minimise this, a clear statement of the role of the researcher was explained to participants in the preamble to data collection.	Practices were provided with information sheets, confidentiality agreement and consent forms.<br>Informed consent was obtained from each participant before involvement.<br>Lacking details on how researchers handled issues raised for participants by the study.<br>Ethical approval was granted by the Mater Health Services Human Research Ethics Committee.	"Key themes were identified using inductive thematic analysis and NVivo software was used to assist with data management. Analysis was iterative and data collection ceased when no new issues emerged, suggesting data saturation. RF and MK read each transcript and independently coded data, identifying a preliminary list of themes. RF produced a refined list of major themes and subthemes; MK endorsed these themes. Because similar themes were identified during the focus groups and interviews, the data were considered comparable and therefore analysed together."<br>Sufficient data were presented to support the findings.<br>Some Contradictory data were presented in the findings.<br>Authors were aware of the potential bias in data analysis and stated that they critically reflected on how their own views and differing perspectives were influencing interpretation.<br>One of the authors worked outside the field and was able to bring more objectivity.	The findings were explicit and clearly discussed in relation to the research question.<br>Adequate discussion of the findings in relation to the wider literature.<br>The researchers discuss the use of more than one analyst enhancing the credibility of the study.<br>In addition, anonymised transcripts were provided to participants to give an opportunity for any further feedback.	The researcher provides an extensive list of recommendations for practice in relation to each of the main themes identified in the study.<br>The research builds on the body of literature that focusses on the refugee perspective.<br>Further areas for research are identified<br>It is implied that this research will be able to help inform refugee healthcare on a national level although it is acknowledged that this research was carried out in one healthcare model.
Feldmann CT, Bensing JM, de Ruijter A. Worries are the mother of many diseases: General practitioners and refugees in the Netherlands on stress, being ill and prejudice.	The aims of the research clearly stated.<br>The importance and relevance of the research were articulated.<br>Qualitative	Authors state that "we set up an open ended, explorative study to learn about their frames of	66 refugees, 24 general practitioners.<br>Refugee participants were approached through refugee initiated community organisations, Dutch Council for Refugees and personal networks (at least	Refugees: In-depth interviews were conducted by the first author (female former GP) with the help of female Somali or Afghan researchers. Setting for collection of data not	Researcher's role, potential bias and influence in research question formulation or data collection.	Lacking details about how the research was explained to participants.<br>Interviews were conducted with consent from the	Refugees: "The first author analysed and coded the transcripts of the refugee interviews, using the WinMAX software program to organise the data and facilitate retrieval... After initial coding and cross-sectional comparison, a	The findings are explicit and discussed in relation to the research question.<br>Authors discuss the findings in relation to the wider literature.<br>No discussion of the	The contribution of the study to inform healthcare practice is discussed. A number of practice implications are given.<br>Potential new areas of research are

For peer review only - http://bmjopen.bmj.com/site/about/guidelines.xhtml

Study reference*	Aims & methods	Research design	Sampling†	Data collection	Reflexivity	Ethical issues	Data analysis†	Discussion of findings	Value
Patient Educ Couns Mar 2007;65(3):369-380	methodology is appropriate for investigating the views of refugee patients and general practitioners about medically unexplained physical symptoms.	reference, expectations and experiences concerning health and healthcare."	partially purposive). Most GPs were a convenience sample from a letter sent to 325 GPs. 3 GPs were selected through personal contacts. Not clear what criteria were applied at the recruitment stage, but the refugees sample was shown to be diverse and representative. GPs had significant experience of caring for this group (21 had > 5 years' experience caring for Somali and Afghan refugees).<br>No discussion around non-participation.	described.<br>No justification given for methods or setting of data collection.<br>Topic list used that was developed in consultation with refugee experts and used in a flexible way. It was adapted during data collection, adding issues that seemed important. Areas of enquiry are described.<br>Data recorded on tape and transcribed verbatim.<br>Data saturation not discussed.<br>GPs: Semi-structured interviews with open ended questions were conducted by a medical student (22) and the first author (3). Setting is not fully described, but reported that 12 were conducted on the telephone and 12 face-to-face at a place of participants' preference. States that the GP participants were likely to give a more positive response towards refugees as they were willing to make time for the interview.<br>No justification given for methods or setting of data collection.<br>Not clear whether a topic list was used for these interviews or the areas of enquiry covered.<br>3 interviews were tape recorded and transcribed verbatim. 21 were recorded through note-taking with the interviewer conscientiously elaborating on them immediately afterwards.<br>Data saturation not discussed.		participants. No discussion about how confidentiality was maintained or how issues raised through the study were handled by researchers.<br>No reference to ethics committee reported.	schematic presentation in short quotes was made of each refugee interview"<br>GPs: "The GP interviews were analysed and coded in the same way. A short profile was written for each doctor, linking interview results to doctor and practice variables. In an initial analysis, rough codes were assigned for the doctors' perceptions of the refugee groups, the problems the refugees presented to them, the way they dealt with these problems, and the constraints they met."<br>A secondary analysis was performed on both refugee and GP data with further content analysis, which formed the body of the article.<br>Sufficient data are presented to support the findings.<br>Contradictory data were taken into account.<br>No examination of researcher's role, potential bias and influence during the analysis and in presentation of the data.	credibility of the findings.	suggested.<br>No discussion of transferability to other populations.
Furler J, Kokanovic R, Dowrick C, Newton D, Gunn J, May C. Managing depression among ethnic	The aims of the research were stated.<br>Research question does not define	Use of qualitative design not Justified, however, in	8 family physicians. Participants were included as part of a larger study known as 'Re-order', but lacking details on how they	Semi-structured interviews conducted by one of the authors (RK) and a research assistant. Lacking details about the	No critical examination of the researcher's role, potential bias and influence in research	Insufficient details about how the research was explained to participants.	"Three authors read transcripts and analysed them independently to identify themes and categories. Results were compared and discrepancies	The findings were explicit and clearly discussed in relation to the research question. The evidence from the	Discusses the findings in relation to practice of physicians and their approach to working with depressed patients

For peer review only

Study reference*	Aims & methods	Research design	Sampling†	Data collection	Reflexivity	Ethical issues	Data analysis†	Discussion of findings	Value
communities: A qualitative study. Annals of Family Medicine May-Jun 2010;8(3):231-236.	participants as refugees, but throughout the study it is apparent that they are refugees. The importance and relevance of the research were articulated. Qualitative methodology is appropriate to understand experiences of family physicians that work with patients with depression.	the discussion section, Authors state that the findings would not be found through conventional studies of medical records, billing records or patient reports.	were recruited. Explained that the participants were chosen because they were known to work extensively with a range of refugee and migrant communities (Table 1 displays length of time they had worked with these communities). No discussion about reasons for non-participation.	exact location of data-collection No justification given for methods or setting of data collection. Brief explanation of the areas covered in the interviews, but the full interview schedule is provided in an on-line appendix. Interviews lasted 1-1.5 hours and were audio-recorded and transcribed. Data saturation not discussed.	question formulation or data collection.	Lacking discussion about how consent was gained, confidentiality maintained and how issues raised by the study were handled by researchers. Ethical approval for the study was granted by the University of Melbourne Human Research Ethics Committee.	discussed with the wider group, and concepts were further refined. Additional thematic categories were added as the analysis developed." Authors emphasise that transparency in analysis and reporting was achieved by providing extensive verbatim quotes and independent assessments of transcripts and themes. Sufficient data were presented to support the findings. Contradictory data were not presented in the findings No examination of researcher's role, potential bias and influence during the analysis and in presentation of the data.	wider literature is discussed in relation to the findings of the study. Authors acknowledge that the sample was small and that the physicians were working with specific cultural groups. They also mention that 3 authors were involved in the thematic analysis and themes were discussed with the wider group.	in ethnic communities. Suggest areas for future research Lacking discussion about the transferability of the findings of the study or other ways the research could be used.
Griffiths R, Emrys E, Lamb CF, Eagar S, Smith M. Operation Safe Haven: The needs of nurses caring for refugees. Int J Nurs Pract Jun 2003;9(3):183-190.	The aims of the research clearly stated. The importance and relevance of the research were articulated. Qualitative research is an appropriate methodology to ascertain the needs of nurses that worked with refugees arriving from conflict areas.	Use of qualitative design not explicitly justified.	13 nurses, 1 medical records clerk, 2 nurse managers. Researcher explains that all the nurses and midwives employed at the centre during its 14-month operation were invited to participate in focus group discussions (Convenience sampling). 14 positive responses were received, which included a medical records clerk. Unclear how the two nurse managers were chosen for semi-structured interviews. Unclear why some people did not participate in the study, but the authors hypothesise that it could have been due to the distance from residence to study location, nurses no longer working in the same workplace or unable/unwilling to participate.	Data was collected through 2 focus groups (13 nurses and 1 medical records clerk) and 2 semi-structured interviews (Nurse managers). No information is given about the settings of data collection or the researcher(s) that conducted interviews. No justification given for methods or setting of data collection. For focus groups, an interview schedule developed by the researchers was used to guide discussion. 5 areas of discussion were described that were triggered by interview questions. Semi-structured interviews lasted 60-9-0 min and followed another format developed by the researchers, but lacking detail on the areas of discussion. Data were audio-recorded and transcribed Data saturation not discussed.	No critical examination of the researcher's role, potential bias and influence in research question formulation or data collection.	Lacking details about how the research was explained to participants and how consent was gained. To protect confidentiality, all participants were assigned a pseudonym. Lacking details on how researchers handled issues raised for participants by the study. Ethics approval was obtained from the South Western Sydney Area Health Service Research Ethics Committee and the University of Western Sydney Ethics Review Committee.	"Thematic analysis of focus group and in-depth interview transcripts was undertaken by a multidisciplinary research team, who re-read them several times to become immersed in the data. The team, drawing upon informants' stories of their experiences, then generated broad themes common throughout the text. Themes and emerging subthemes identified by the research team were then coded from the transcripts using a qualitative data management program (QSR Nvivo, QSR International)." Sufficient data were presented to support the findings, however the authors did not include many quotations. Some contradictory data are presented in the findings No examination of researcher's role, potential bias and influence during the analysis and in presentation of the data.	The findings are explicit and discussed in relation to the original research question. The findings are discussed within the context of the wider evidence in the literature. No explicit discussion of the credibility of the results, but authors report that a team conducted the thematic analysis implying multiple researchers involved in generating themes from the data.	The contribution of the study to practice within similar settings is discussed. Several recommendations are given for health care providers to improve support for nurses caring for refugees. Authors discussed how the findings might be relevant in other contexts and further research areas are suggested.
Jensen NK, Norredam M, Priebe S, Krasnik A. How do general	The aims of the research clearly stated.	Use of qualitative design not	9 general practitioners. The participants were purposively selected based	Semi-structured interviews took place at the workplace of the	No critical examination of the researcher's role,	The research was explained to the participants in a	Qualitative content analysis was undertaken. "The interviews were read several times to	The findings are explicit and discussed in relation to the	The researchers briefly consider the findings in the context of national

For peer review only

Study reference*	Aims & methods	Research design	Sampling†	Data collection	Reflexivity	Ethical issues	Data analysis†	Discussion of findings	Value
practitioners experience providing care to refugees with mental health problems? A qualitative study from Denmark. BMC Family Practice 2013;14:17.	The importance and relevance of the research were articulated. Qualitative methodology is appropriate for exploring general practitioner's experiences of providing care for refugees with mental health problems.	explicitly justified.	on working in clinics with high proportions of immigrants and were expected to have a high experience of working with immigrant and refugee patients. The research was explained to participants in a letter which was followed up with a phone call with further details and to inquire about their interest in taking part in the study. No discussion of non-participation.	professionals and carried out by the first author. No justification given for methods or setting of data collection Methods for data collection are described. An interview guide was developed by a project coordinating group in London (study was part of a broader EU project) and translated into Danish for use in this study. The first part of the interview included questions around delivery of care to immigrants in general. The second part began with a vignette (scenario of a refugee patient consultation), with pre-prepared questions to begin discussion. Interviews were recorded on a Dictaphone and transcribed. Data saturation was not discussed.	potential bias and influence in research question formulation or data collection.	letter, with more details being given in a phone call. Informed consent was obtained orally from all participants and they were ensured anonymity. Lacking details on how researchers handled issues raised for participants by the study. Ethical permission for this study has been waived by the Ethical Committee of the Capital Region of Denmark as Danish legislation does not require ethical approval for this type of study.	obtain a sense of the whole. The text was then divided into meaning units, which were then condensed and assigned categories and themes in a process moving towards a higher level of abstraction. The creation of categories and themes took place as an iterative process with ongoing reflection and revision of categories and themes. The whole context of the interviews was considered concurrently throughout this process. The initial analysis was carried out by the first author, but presented to and discussed with co-authors and other researchers with a background in public health, medicine and anthropology as part of the analytic process." Sufficient data are presented to support the findings. Contradictory data are presented and discussed. No examination of researcher's role, potential bias and influence during the analysis and in presentation of the data.	original research question. Findings are discussed within the context of evidence in the wider literature. Credibility of findings not explicitly discussed, but the author mentions that the initial stages of the analysis were conducted by the lead author and as themes emerged, they were discussed with the wider group including members from different discipline backgrounds.	policy for health care management of refugees. Briefly suggests ways to improve practice. Suggest the development of conversational models for general practitioners with points to be aware of in consultations with refugees. There is some discussion of the transferability of the results. The authors acknowledge that the participants had high levels of knowledge about refugees and asylum seekers, which is not true of many general practitioners. In addition, the vignette used for the interview gave a theoretical, isolated situation, which they acknowledge may limit generalisability.
Johnson D.R., Ziersch A.M., Burgess T. I don't think general practice should be the front line: Experiences of general practitioners working with refugees in South Australia. Australia and New Zealand Health Policy 2008;5(pagination):Arte Number: 20. ate of Pubaton: 08 Aug 2008.	The aims of the research clearly stated. The importance and relevance of the research were articulated. Qualitative methodology is appropriate to explore the challenges for GPs working with refugees.	Researchers justify the use of qualitative methodology. A qualitative approach was taken in order to gain a deeper understandin g of the challenges faced by general practitioners in private practice when providing care to refugees.	12 general practitioners and 3 medical directors of divisions. Potential participants were identified through a database of GPs who could be identified as having accepted refugee referrals. One of the authors also used his personal knowledge from previous related work. Further GPs were identified after interviews with medical directors of divisions. An introductory letter/invitation was sent to 77 potential GP participants, providing 6 participants. the remaining six were recruited through follow up phone calls. Medical directors of divisions were contacted by email with 2 agreeing to participate with a further participant agreed after a follow up phone call. These	Data were collected through semi-structured interviews. Most were conducted individually, but 3 of the GPs were conducted together in a group setting. No description of the setting for data collection or who conducted interviews. Use of semi-structured interviews was justified. They were able to examine challenges already identified in the literature as well as allowing new themes to emerge. No justification of setting. Lacking detail on how the interviews were conducted, but does briefly outline the general focus of the questions for the GPs and the medical directors of divisions. The interviews were tape recorded and transcribed	No critical examination of the researcher's role, potential bias and influence in research question formulation or data collection.	Lacking details about how the research was explained to participants and how consent was gained. Confidentiality was protected by assigning participants random numbers in the coding process. Lacking details on how researchers handled issues raised for participants by the study The study was approved by the University of Adelaide Human Research Ethics Committee.	"A template analysis approach was adopted where a coding template was developed which included a priori themes in addition to new themes identified from initial reading and analysis of the transcripts. Final thematic templates for both the GP and Division transcripts were agreed upon by the Project Team and then all data was coded according to these themes, with DJ undertaking the bulk of the coding. Two transcripts were also independently coded by the other members of the Project Team. Following this, comparisons were made and a consensus reached on how the remaining data was to be coded." Sufficient data are presented to support the findings. Contradictory data not presented No examination of researcher's role, potential bias and influence	The findings are explicit and discussed in relation to the original research question. Findings are discussed within the context of evidence in the wider literature. Lacking discussion of the credibility of the findings 2 out of 15 transcripts were independently coded by multiple researchers.	Considered the findings of the study in relation to practice and policy. Suggested that to provide more generalisable results a quantitative study should be conducted, but does not give any information about the aims of such a study. The authors discuss the transferability of the study and state that the small numbers limit its generalisability.

For peer review only - http://bmjopen.bmj.com/site/about/guidelines.xhtml

Study reference*	Aims & methods	Research design	Sampling†	Data collection	Reflexivity	Ethical issues	Data analysis†	Discussion of findings	Value
			were contacted because their areas were thought to contain high levels of refugee settlement.<br>Discusses the low response rate and some of the potential bias around those who did participate (i.e. participants more likely to be dissatisfied with current system of provision), however the researchers believe that there were also limited numbers of GPs with experience working with refugees.	verbatim.<br>Researchers state that data saturation was reached.			during the analysis and in presentation of the data.		
Kokanovic R, May C, Dowrick C, Furler J, Newton D, Gunn J. Negotiations of distress between East Timorese and Vietnamese refugees and their family doctors in Melbourne. Sociol Health Illn May 2010;32(4):511-527.	The aims of the research clearly stated.<br>The importance and relevance of the research were articulated.<br>Qualitative methodology is an appropriate methodology to explore how migration experiences are manifested in the lives of the participants and how resulting distress is negotiated and contested in their interaction with family doctors.	Use of qualitative design not explicitly justified, however the choice of in-depth interviews was justified as it allowed enough time for respondents to talk about their lives in their own words and focus on issues that were important to them.	5 general practitioners, 24 refugees from Vietnam and East Timor.<br>The refugee participants were purposively selected to include patients who had experienced depression and had used health services for depression care. They were recruited if they had been diagnosed with depression or prescribed antidepressants in the past year.<br>Lacking details about how refugee participants were first contacted, but the initial approach involved use of interpreters to explain the study. Those agreeing to be contacted by the research team were telephoned by a bilingual researcher with more information and to arrange a time for the interview.<br>Unclear how the GPs were selected or recruited to the study.<br>Authors give characteristics of the participants that suggest that these were an appropriate sample (10-25 years' experience).<br>No discussion around non-participation.	In-depth interviews were conducted. Most interviews were conducted in the Community Health Centre, but some (number not reported) of the refugees were interviewed in their homes. Interviews conducted by experienced qualitative researchers (first author and research fellow)<br>Researchers justified use of in-depth interviews as it allowed enough time for respondents to talk about their lives in their own words and focus on issues that were important to them.<br>Setting not justified<br>Authors report the use of an interview guide for a section of the interviews, but unclear about the full methods used with the refugees and the GP's.<br>The differing areas of discussion with refugees and GP's were outlined.<br>Interpreters were utilised for the majority of the interviews with refugees.<br>Data were audio-recorded and research notes were kept by the interviewer and interpreter. These were translated and transcribed in duplicate	Researchers discuss the complexities of interviewing using translators and the impact on researcher-interviewee communication. It is uncertain whether interpreters may have summarised or modified questions, answers and meanings.	Research was explained to refugees in their own language (through interpreters) at the initial contact and then in more detail in a telephone call. Unclear how the research was explained to GPs.<br>Consent was gained from all participants using consent forms in English or translations into relevant languages. No discussion of confidentiality or how issues raised through the stud for participants were handled by researchers.<br>Ethical approval was given by the University of Melbourne Human Ethics Research Committee.	An inductive thematic approach was taken.<br>The themes from the preliminary coding were used to create a coding frame which was applied to the data across all transcripts. The transcripts were marked and annotated, and emerging themes were then discussed among authors.<br>Unclear who and how many people coded the transcripts.<br>Sufficient data are presented to support the findings.<br>Researchers refer to contradictory data within their dataset.<br>No examination of researcher's role, potential bias and influence during the analysis and in presentation of the data.	The findings of the study were explicit, discussed in relation to the research question and set in context of the wider literature.<br>No explicit discussion of the credibility of the findings, but it is reported that transcripts were translated in duplicate and multiple researchers were involved in developing emerging themes.	Limited discussion of the contribution of the findings to practice or policy. Authors do suggest a reinvestigation of the way of conducting research on depression in a cross-cultural context.<br>Researchers point out that the issues around negotiation of distress investigated in this paper are broadly relevant in (cross-cultural) clinical practice.

For peer review only

For peer review only - http://bmjopen.bmj.com/site/about/guidelines.xhtml

Study reference*	Aims & methods	Research design	Sampling†	Data collection	Reflexivity	Ethical issues	Data analysis†	Discussion of findings	Value
				(to maximise legitimacy). Data saturation was not discussed.					
Kurth E, Jaeger FN, Zemp E, Tschudin S, Bischoff A. Reproductive health care for asylum-seeking women - a challenge for health professionals. BMC Public Health 2010;10:659.	Aims of the study were clearly stated and its importance and relevance articulated.<br>A qualitative study is appropriate to explore the perceptions of health care professionals providing health care to asylum seeking women.	Use of qualitative design not explicitly justified.	80 asylum seekers, 3 physicians, 3 nurse/ midwife, 1 psychologist, 3 interpreters<br>Purposive sample.<br>The people who were invited to participate were those who had been most involved with the asylum-seeking patients insured in the Health Maintenance Organisation (HMO) model - a service specifically set up for asylum seekers in Basil University Hospital.<br>All the professionals invited agreed to participate.	Semi-structured interviews were conducted in a quiet hospital room of the participant's choice.<br>Information about interviewers is not reported.<br>No justification given for methods or setting of data collection.<br>An interview guide was used. Areas of enquiry are described. Additional questions were asked to physicians about roles specific to them.<br>The interviews were audio recorded and transcribed verbatim.<br>Data saturation was not discussed.	The researcher's role and potential bias in the formulation of questions or data collection was not discussed.	Participants were informed about the aims of the study and they signed a consent form.<br>Lacking details about how confidentiality was maintained or how researchers handled issues raised for participants by the study<br>The study was approved by the joint ethical committee of the Cantons of Basel Stadt and Basel Land (Ethikkommission beider Basel).	Analysis followed steps of grounded theory methodology. "We started the process by open coding, which means that we categorized text segments into broad categories or themes … We continued with axial coding which included examining relationships between categories and connecting them accordingly…Finally, selective coding included the organisation of the diverse categories into a framework to explain the phenomenon under study. This framework is depicted and explained in details in the result section. To strengthen the rigour of the analysis, we discussed the results with experts in women's health, ethics and research."<br>Sufficient data are presented to support the findings.<br>Contradictory data not taken into account<br>No critical examination of researcher's role and influence in the analysis	The findings of the study were explicit, discussed in relation to the research question and in context of the wider research literature<br>Cross validation of quantitative and qualitative elements was thought to add to credibility.	Considered the value of the study and the findings in relation to practice and policy.<br>Suggests areas for further research.<br>Researchers discussed the limitations of the small sample size and the research being conducted in a hospital setting with highly developed services.<br>Authors suggest that challenges could be greater in other settings.
Lawrence J, Kearns R. Exploring the 'fit' between people and providers: refugee health needs and health care services in Mt Roskill, Auckland, New Zealand. Health & Social Care in the Community 2005 Sep;13(5):451-461.	Aims of the study were clearly stated and its importance and relevance articulated.<br>Qualitative methodology is appropriate for investigating barriers faced by refugees in accessing health services and challenges faced by providers to meet their needs.	Use of qualitative design not explicitly justified, however the authors explain why they chose in-depth interviews as a data collection method.	5 Community representatives, 9 Refugee group representatives, 5 Medical practitioners, 1 Manager, 1 Administrator.<br>Participants were purposively selected in consultation with staff at the clinic. Community representatives selected based on length of involvement in the Mt Roskill community.<br>Refugee representatives were representative of ethnic groups in the area and chosen based on involvement with the community.<br>All seven members of staff at the clinic were sampled.<br>No discussion around non-participation of community representatives.	In-depth interviews were conducted with participants with most taking place at the clinic and some in the workplace of representatives. All interviews were conducted by the first author.<br>Authors justify their use of in-depth interviews: "Our rationale for this approach is that experience is constituted in participants' accounts as they talk about their surroundings and reactions to them in ways which others can accept and understand. In-depth interviews are a suitable way of gathering and accessing such talk".<br>Setting justified on	The researcher's role and potential bias in the formulation of questions or data collection was not discussed.	Not clear how the study was explained to participants.<br>Respondents gave permission in accordance with agreed ethics protocols, but no further details.<br>No discussion of how confidentiality was maintained or how issues raised in the study were handled by researchers.<br>No reference to ethics committee reported.	A thematic analysis process is described, but it isn't clear whether this applied to all participant groups.<br>"we used a research framework that was built on a critical realist theoretical base, which assumes that realities are socially, culturally and historically situated, but are, nevertheless, experienced as material, objective and stable by participants ...After a period of familiarisation with the transcribed narratives, key themes were identified with reference to topics discussed in the interviews. Indicative narratives identified through this exercise are used to illustrate themes in this paper." No indication of involvement of multiple researchers in the analysis.<br>Sufficient data are presented	The findings of the study were explicit, and discussed in relation to the research question.<br>Limited discussion of the findings in the context of the wider literature.<br>No discussion of the credibility of the findings.	Authors discuss the contribution the study makes to existing knowledge and understanding. The findings are discussed in relation to practice and policy.<br>It is acknowledged that the study focussed on one clinic in one city.<br>Suggestion of conducting further similar studies in other locations to increase generalisability.

For peer review only - http://bmjopen.bmj.com/site/about/guidelines.xhtml

Study reference*	Aims & methods	Research design	Sampling†	Data collection	Reflexivity	Ethical issues	Data analysis†	Discussion of findings	Value
				grounds of convenience<br>A list of topics covered by the interviews is included.<br>Data were audio taped and transcribed.<br>Data saturation is not discussed.			support the some of the findings, however the section reporting health practitioner's experiences did not provide supporting quotations for some of the key challenges presented.<br>Contradictory data are not presented<br>No examination of researcher's role, potential bias and influence during the analysis and in presentation of the data.		
Riggs E, Davis E, Gibbs L, Block K, Szwarc J, Casey S, et al. Accessing maternal and child health services in Melbourne, Australia: Reflections from refugee families and service providers. BMC Health Serv Res 2012 01;12(1):117-117 1p.	Aims of the study were clearly stated and its importance and relevance articulated.<br>Qualitative methodology is appropriate to explore perspectives of parents from refugee backgrounds and service providers.	Qualitative research design is justified and the use of focus groups was justified on the grounds that some of the participants favoured this approach.	87 refugee background mothers, 12 nurses, 1 community worker, 1 community liaison, 5 bilingual workers,<br>3 community representatives, 2 managers of bilingual workers<br>The refugees were recruited by convenience sampling at locations where they were known to attend - playgroups, kindergarten, peer education programme, English language organisation. They were invited to participate through a bilingual worker/health worker who was known to them.<br>Researchers took measures to recruit a more representative sample when it became apparent that initial focus groups were not representative.<br>Researchers sought to understand the depth of experiences of refugee background parents had when engaging with MCH services.<br>Healthcare service providers were recruited through purposive sampling. Lacking information about how they were recruited or why they were an appropriate sample.<br>No discussion of reasons of non-participation.	7 Focus groups were used to collect data from refugees and 4 focus groups were used to collect data from service providers. Interviews used with community representatives/managers of bilingual workers. All focus groups were conducted by ER with assistance from KB or ED The setting of data collection is not described.<br>Use of focus groups and interviews was justified, but setting not described or justified.<br>Focus group guides were used and the areas of questioning were described. Modifications were made to the questions to maximise relevance for different groups.<br>Unclear what methods were used for the interviews<br>Focus groups and interviews were digitally recorded and transcribed, including interpreter translations.<br>Data saturation was not discussed.	The researcher's role and potential bias in the formulation of questions or data collection was not discussed.	Lacking details about how the research was explained to participants. A plain language statement and consent form were provided. No discussion around confidentiality or how issues raised throughout the study were handled by the researchers.<br>Ethical approval was given by the University of Melbourne and the Department of Education and Early Child Development.	Thematic analysis was used.<br>"ER listened to all voice recordings, read and coded all transcripts, and developed categories to organise the data. ED and LG also read a sub-sample of transcripts and coded them. The coding was found to be very similar with any differences discussed by the researchers to arrive at a consensus about final codes. The researchers also discussed patterns, consistencies and contradictions within the data to develop the main themes. ER then refined the themes in consideration of their alignment with the existing literature. All research investigators and the study advisory group came together to discuss the themes, further interpret and explain the results and the implications and applications of the findings."<br>Sufficient data are presented to support the findings, however not all subthemes are supported with direct quotations from participants.<br>contradictory data were taken into account in the analysis.<br>No examination of researcher's role, potential bias and influence during the analysis and in presentation of the data.	The findings of the study were explicit, discussed in relation to the research question and extensively discussed within the context of wider literature.<br>Researchers discussed the credibility of the findings. They discuss triangulation when combining data from the focus groups and interviews, which they suggest can lead to an enhanced description of the phenomenon being explored.<br>Although not explicitly discussed in terms of credibility, the involvement of multiple researchers in coding a sample of transcripts and development of themes enhances the credibility of the findings.	Considered the value of the study and the findings in relation to practice and policy. Suggested areas of further research. In particular, to assess the 'refugee mentor' model described as a potential way to promote access to MCH services.<br>The authors discuss the generalisability of the findings. They comment that as the study was conducted in outer urban areas of Melbourne, the findings may not be applicable to other locations in Victoria (e.g. rural and regional areas).
Samarasinghe K, Fridlund B, Arvidsson B.	Aims of the study were clearly stated	The authors discussed	34 PHCNs. Purposive sampling was used to	Interviews were conducted at the	Authors critically examine the	Research was explained to the	Contextual analysis with reference to phenomenography	The findings are explicit and discussed	The authors discuss the contribution the study

For peer review only - http://bmjopen.bmj.com/site/about/guidelines.xhtml

Study reference*	Aims & methods	Research design	Sampling†	Data collection	Reflexivity	Ethical issues	Data analysis†	Discussion of findings	Value
Primary health care nurses' promotion of involuntary migrant families' health. Int Nurs Rev 2010;57(2):224-231.	and its importance and relevance articulated.<br>Qualitative methodology is appropriate for exploring the experiences of primary health care nurses (PHCN) in health promotion with involuntary migrants.	their reasons for using a phenomenographic approach.	select participants for the study, which sought a wide spectrum of participants (sex, age, ethnicity, specialist education, length of primary health care nursing practice).<br>It is stated that each PHCN nurse had worked with approximately 200 involuntary migrant families, indicating that they would have the knowledge required for the studies aims.<br>No discussion about non-participation.	participant's workplace and were all conducted by the first author.<br>Methods not explicitly justified, however researchers explain that they piloted the interview questions beforehand to test the relevance of the questions (these pilots were included in the analysis). No justification given for choice of setting.<br>Areas of enquiry in the interview are described and the interviews lasted approximately 60 min each.<br>No modifications in the methods were necessary.<br>Interviews were tape recorded and transcribed verbatim.<br>Data saturation was not discussed.	influence of the researcher during the interview. "The first author, being a former PHCN herself, may have contributed to a common bond between the participants and the author, making the PHCNs able to freely express their thoughts throughout the interviews, which is crucial in a qualitative study"	participants through verbal and written information, including their right to withdraw from the study at any time.<br>Participants gave written consent and were assured of confidentiality (data being unidentifiable)<br>Lacking details on how researchers handled issues raised for participants by the study.<br>The study was approved by the university ethics committee of Sweden.	was used.<br>"The first author with experience of working within PHC carried out the analysis, while the two co-authors with specialized knowledge of the methodology served as additional evaluators in the categorization procedure.…The analysis was carried out in six steps: (1) the transcribed interviews were read several times to obtain a sense of the whole; (2) the interviews were processed, and descriptive statements relating to the aim of the study were identified, delimited, analysed and structured into an overview of concepts and keywords; (3) a comparative reduction of the data was commenced by giving a summarized description of each interview from this overview; (4) the summarized descriptions were differentiated by comparisons in relation to similarities and differences of the summarized descriptions, and were grouped together in three qualitatively distinct groups; (5) the underlying structure of the grouped descriptions was identified and described by going back and forth between the grouped descriptions and the original interviews;<br>(6) and the transcribed interviews of the 34 participant PHCNs were finally allocated to the three qualitatively distinct groups of these descriptions."<br>Sufficient data are presented to support the findings.<br>No examination of researcher's role, potential bias and influence during the analysis and in presentation of the data.	in relation to the original research question.<br>Findings are discussed in relation to the wider literature.<br>Authors discuss the process of re-evaluating data in validating the descriptive categories, including the choice of quotations.<br>The analysis was conducted by one person (lead author), but was evaluated by two other co-authors.	makes to informing clinical practice and policy.<br>Recommendations are given to improve the training of nurses, to equip them to work with involuntary migrant families.<br>Further research is suggested to determine how to facilitate cultural transition for involuntary migrants.<br>Transferability of the findings is discussed.
Suurmond J, Rupp I, Seeleman C, Goosen S, Stronks K. The first contacts between healthcare providers and newly-arrived asylum seekers: A qualitative study about which issues need to be addressed. Public Health Jul	Aims of the study were clearly stated and its importance and relevance articulated.<br>Qualitative methodology is appropriate for investigating the issues that healthcare providers	Use of qualitative design not explicitly justified.	36 nurse practitioners and 10 public health physicians.<br>Participants were a purposive sample of nurse practitioners and public health physicians from different asylum seeker centres (from across the Netherlands). They were approached by the coordinator to ascertain if	7 group interviews were used to collect data and were conducted by two specified researchers (IR and CS). The setting was not described.<br>No justification given for methods or setting of data collection.<br>A topic list was used, which had been	The researcher's role and potential bias in the formulation of questions or data collection was not discussed.	Written information about the study was given to all participants who were assured of confidentiality and anonymity (Anonymity was assured by the use of codes).	Data was analysed by Framework approach.<br>Interviews were analysed, starting with the familiarization stage. "Short notes were made to identify themes. This resulted in a thematic framework. The framework was systematically applied to the material, and all interviews were reread and annotated accordingly. Charts	The findings are explicit and discussed in relation to the original research question.<br>There is adequate discussion of the findings in context of the wider literature.<br>No discussion of the credibility of the	The authors discuss the findings in relation to practice and policy, providing perspectives and models that can inform service provision for this group.<br>Authors sought to provide a generic model (beyond first contact) for healthcare provision

For peer review only - http://bmjopen.bmj.com/site/about/guidelines.xhtml

Study reference*	Aims & methods	Research design	Sampling†	Data collection	Reflexivity	Ethical issues	Data analysis†	Discussion of findings	Value
2013;127(7):668-673.	need to address in their first contact with asylum seekers.		they were willing to participate in a group interview. The role of these health professionals in caring for asylum seekers was described, giving justification for their selection. No discussion around non-participation.	developed from the results a survey that had previously been sent to a sample of nurse practitioners and public health physicians. Areas of enquiry are described. All interviews were recorded on tape and transcribed. Data saturation was not discussed.		Informed consent was tape-recorded a priori the interviews. Lacking details on how researchers handled issues raised for participants by the study. The employer of the nurse practitioners and public health physicians (Community Health Services for Asylum Seekers) approved the study. Medical-ethical approval of this study was not required, according to the Dutch Medical Research Involving Human Subjects Act as it only involved care providers and it was not an intervention study.	were devised with headings (and sometimes subheadings) for each key theme. Each chart contained entries for several respondents. Finally, these charts were used to describe patterns through an iterative, comparative process of searching, reviewing, and comparing the data." Sufficient data are presented to support the findings." No indication of involvement of multiple researchers in the analysis. Contradictory data were not presented. No examination of researcher's role, potential bias and influence during the analysis and in presentation of the data.	findings.	for asylum seekers, to increase generalisability to other settings.
Suurmond J, Seeleman C, Rupp I, Goosen S, Stronks K. Cultural competence among nurse practitioners working with asylum seekers. Nurse Educ Today 2010 11;30(8):821-826 6p.	Aims of the study were clearly stated and its importance and relevance articulated. Qualitative methodology is appropriate to explore the views of nurse practitioners about cultural competencies that are important for working with asylum seekers.	Qualitative methodology not explicitly justified.	89 nurse practitioners completed questionnaires. 36 nurse practitioners participated in group interviews. Not reported whether there was overlap in these two data sources. Participants in the questionnaire were a convenience sample. Those who returned the questionnaire were included. It is not known how many questionnaires were distributed, so a response rate cannot be given. Participants for the group interviews were a purposive sample, selected by local coordinators in order to increase representation from different asylum seeker centres and maximise variation in experiences.	89 questionnaires and 7 group interviews were used to collect data., which were conducted by 2 named researchers. The setting for data collection is not clearly described The combination of questionnaires and group interviews (triangulation) was put forward as a way of increasing credibility. No justification of setting No discussion of how the questionnaires were developed. A topic guide was used for group interviews; however, the questions were not focussed on this particular research question. Data about cultural competence emerged in the course of the discussions.	The researcher's role and potential bias in the formulation of questions or data collection was not discussed.	Information about the study was given in the form of a flyer as well as in a letter accompanying the questionnaire. Lacking details about how the research was explained to participants in group interviews. Consent was gained from all participants and they were assured of confidentiality. Lacking details on how researchers handled issues raised for participants by the study. According to the Medical Research	Framework approach was used to analyse the data. "After familiarisation with the data, a coding framework was identified. The questionnaires were then systematically coded using this framework. Data were subsequently charted and three major charts were constructed: educational background, important cultural competences in connection with asylum seekers, and ideas about how cultural competences may be improved. The transcription of each group interview was read carefully to gain an overall impression before being coded and analysed. One chart was designed on the basis of different cultural competences that were mentioned in the interviews. Using this chart, patterns and connections could be described." Not clear how the two sources of data were	The findings are explicit and discussed in relation to the original research question. There is adequate discussion of the findings in context of the wider literature. Authors suggest that credibility is enhanced by having two data sources (questionnaires and group interviews), which allows triangulation.	The contribution to existing knowledge and understanding is discussed. The authors state that the results of the study can be used for training and education of health care professionals. They believe that the results are relevant to other care providers who work with asylum seekers (generalisable) Further areas of research are not discussed.

For peer review only - http://bmjopen.bmj.com/site/about/guidelines.xhtml

Study reference*	Aims & methods	Research design	Sampling†	Data collection	Reflexivity	Ethical issues	Data analysis†	Discussion of findings	Value
			The role of these health professionals in caring for asylum seekers was described, giving justification for their selection. No details given about reasons for non-participation.	Group interviews were recorded on tape and transcribed. Data saturation was not discussed.		Involving Human Subjects Act, medical–ethical approval of this study was not required in the Netherlands (only care providers involved and not an intervention study). approval was obtained from the Community Health Services for Asylum Seekers, the employer of the nurse practitioners.	synthesised. No indication of involvement of multiple researchers in the analysis. Sufficient data are presented to support the findings, however only 2 direct quotations are used in the entire findings section, which had 9 headings. Contradictory data were not presented. No examination of researcher's role, potential bias and influence during the analysis and in presentation of the data.		
Tellep TL, Chim M, Murphy S, Cureton VY. Great suffering, great compassion: A transcultural opportunity for school nurses caring for Cambodian refugee children. Journal of Transcultural Nursing Oct 2001;12(4):261-274.	Aims of the study were clearly stated and its importance and relevance articulated. Qualitative methodology is appropriate to explore experiences of school nurses and Cambodian liaisons that provide care for refugee families.	Qualitative methodology not explicitly justified, although it is mentioned that the focus groups were conducted to gain insight into the concepts of transcultural and intracultural reciprocity as experienced by school nurses in their relationships with Cambodian refugees.	6 school nurses, 2 Cambodian liaisons. "A purposive sample of school nurses and Cambodian liaisons was recruited from a school district serving a large population of Cambodian children in California. Six of the district's eight school nurses volunteered as well as two of the three Cambodian liaisons." Invitation was through a phone call or letter. No reasons given for why these participants were chosen, although it is clear that the nurses had a high level of experience working with Cambodian refugees (6-15 years' experience). No discussion of the reasons for non-participation of those approached, that did not volunteer.	Focus group with Cambodian liaisons was held in the home of a non-Cambodian school nurse. Focus group with school nurses was held in their school district conference room. Focus groups were moderated by two of the authors. No justification for the methods Setting of the groups with Cambodians was justified based on wanting to provide a friendly atmosphere and authors explain the cultural reasons for tea/coffee and relational time before the interviews. No justification of the setting of nurse interviews. A semi-structured interview guide was used in the focus group. Broad areas of enquiry are described, but specific questions not stated. Data were tape recorded and transcribed verbatim. field notes were reviewed. Data saturation was not discussed.	The researchers critically examined their roles and potential bias in the data collection. "Through directing the research to look for insights into the concepts of transcultural and intracultural reciprocity, the authors may not have been as open to other concepts arising from the data regarding the nature of the participants' interactions with Cambodian refugee families. In retrospect, serving Cambodian refreshments at the school nurse focus group relayed the school nurse moderator's bias of transcultural interest and empathy toward Cambodians. This bias may have limited the types of information and viewpoints shared by the nurses. In addition, they may have been hesitant to share issues in the presence of the	Lacking details about how the research was explained to participants. Consent was gained before conducting the focus groups and issues of confidentiality were considered. Lacking details on how researchers handled issues raised for participants by the study. "San Jose State University's Human Subjects Institutional Review Board approved the study's research protocol."	Limited details about the analysis methodology or process. "Tapes were listened to and transcripts were reviewed several times by the moderators individually and together. The data were grouped and categorized into emergent issues and themes and also reviewed in light of Dobson's (1989) conceptual framework of transcultural health visiting." No indication of involvement of multiple researchers in the analysis. Sufficient data were presented to support the findings. Contradictory data were not presented. No examination of researcher's role, potential bias and influence during the analysis and in presentation of the data.	The findings are explicit and discussed in relation to the original research question. Little discussion of the findings in the context of the wider literature. As already noted in the reflexivity section, authors acknowledge potential bias in the interview process, which could impact the credibility of the findings.	Authors provide a number of recommendations for nursing practice Areas for further research are suggested. Authors discuss generalisation of findings, pointing out that focus group research results are not meant to be generalised. They refer readers to Kruger's concept of transferability when reflecting on using these findings in other settings.

For peer review only - http://bmjopen.bmj.com/site/about/guidelines.xhtml

Study reference*	Aims & methods	Research design	Sampling†	Data collection	Reflexivity	Ethical issues	Data analysis†	Discussion of findings	Value
					Cambodian nurse assistant moderator. A similar inhibitor may have existed in the presence of the non-Cambodian school nurse assistant moderator with the Cambodian liaison focus group.				
Tobin C.L., Murphy-Lawless J. Irish midwives' experiences of providing maternity care to non-Irish women seeking asylum. International Journal of Women's Health 2014 31 Jan 2014;6(1):159-169.	Aims of the study were clearly stated and its importance and relevance articulated. Qualitative methods are appropriate to explore midwives' perceptions of caring for women in the asylum process and gain insights into how they can be equipped to provide effective care to this group.	Use of qualitative methodology not explicitly justified.	10 midwives. The participants were purposively selected to ensure that they had experience providing care to asylum seekers. They were chosen from two different sites (an urban hospital and a rural hospital) to gain a wider variety of experiences. Information packs describing the study were distributed by researchers to the two sites; the researchers then followed up with visits to the sites to hold information sessions about the study and to answer questions. Demographic information about participants is included, demonstrating the appropriateness of the sample. No discussion around non-participation.	Data were collected by in-depth, unstructured interviews at a place convenient for participants (usually at home of at a private office space). Interviewer not reported. No justification given for methods or setting of data collection Interviews were launched with one open-ended question and ranged from 26-70 minutes. Data were audio recorded and later transcribed verbatim. Data saturation was not discussed.	Extensive field notes and reflective journals were kept, that provide an audit trail of decision-making and an aid for the qualitative researcher to deepen awareness of their own bias, reactions, and emotions to the data as they emerge. Clinical and peer supervision was used throughout the data collection process.	Information packs describing the study were distributed by researchers to the two sites; the researchers then followed up with visits to the sites to hold information sessions about the study and to answer questions. Informed consent, voluntary participation, and assurance of confidentiality were made explicit. Lacking details on how researchers handled issues raised for participants by the study. Article states that ethical approval was gained from relevant institutions, but details not provided.	Data were analysed using content analysis. "The analysis was undertaken by hand, and involved several readings of transcripts, followed by coding of data and grouping coded material based on shared content or concepts to identify common themes. Transcripts were also read in their entirety by a second researcher to confirm the themes that were identified and add to the validity of the findings." Sufficient data is presented to support the findings. Contradictory data are considered No examination of researcher's role, potential bias and influence during the analysis and in presentation of the data.	The findings are explicit and discussed in relation to the original research question. There is adequate discussion of the findings in context of the wider literature. No explicit discussion of the credibility of the findings.	The contribution of the study to existing knowledge and understanding was discussed. highlights the continued difficulties midwives experience in achieving effective communication, understanding difference, and coping with the emotional cost of caring within a hospital-based technological model of maternity care. Recommends ways that service delivery to asylum seekers could be improved. New areas for research are identified. Authors acknowledge that the study is small scale, and cannot be generalised to the whole population.
Twohig PL, Burge F, MacLachlan R. Pod people. Response of family physicians and family practice nurses to Kosovar refugees in Greenwood, NS. Canadian Family Physician 2000 Nov;46:2220-2225.	Aims of the study were clearly stated and its importance and relevance articulated. Qualitative methodology is appropriate for exploring the experiences of family practice nurses and family physicians that cared for refugees in a refugee	Use of qualitative methodology not explicitly justified.	6 family practice nurses, 10 family physicians. Participants were purposively sampled from the service roster to enrol different kinds of family practice nurses and family physicians. Lacking details about how participants were invited to participate. All the participants had worked at the centre that was the focus of the study. No discussion about non-	Data were collected through semi-structured interviews at a 'private setting' (no further details) and were conducted by one team member (PT). No justification given for methods, but does justify setting as a private place to allow the participants to freely and openly share experiences. Lacking explicit details about the methods (no	The researcher's role and potential bias in the formulation of questions or data collection was not discussed.	Lacking details about how the research was explained to participants. Written consents were obtained, but lacked details about confidentiality and how issues raise for participants in the study were handled by researchers.	A form of textual analysis was applied. "For each interview, key words or phrases were identified and compared with subsequent inter views until no significant new ideas emerged. Once researchers were satisfied that saturation had been achieved, words and phrases were grouped into larger conceptual categories. A second researcher reviewed a subset of transcripts and critiqued and confirmed the preliminary categories. This	The findings are explicit and discussed in relation to the original research question. Lacking discussion of the findings in the context of the wider literature. No explicit discussion of the credibility of the findings although researchers report that a second researcher critiqued categories	The contribution made by the study to existing knowledge and understanding is discussed. Teamwork in emergency response is suggested as a possible avenue for further research. Authors acknowledge that the findings of the study cannot be generalised to other relief settings, but

For peer review only - http://bmjopen.bmj.com/site/about/guidelines.xhtml

Study reference*	Aims & methods	Research design	Sampling†	Data collection	Reflexivity	Ethical issues	Data analysis†	Discussion of findings	Value
	processing centre.		participation.	description of interview guide, format, areas of enquiry) Data were audiotaped and transcribed verbatim. Data saturation was discussed in the analysis process. Comparisons of key words and phrases were made across interviews until no new themes emerged.		Ethics approval was obtained from Dalhousie's Faculty of Medicine.	process was repeated until the categories were clear. These categories became the basis for a coding structure within QSR NUD*IST, software designed for textual analysis." Sufficient data were presented to support the findings. Contradictory data were taken into account. No examination of researcher's role, potential bias and influence during the analysis and in presentation of the data.	that emerged in the analysis	suggest that they could offer insights to generate other research questions.
Yelland J, Riggs E, Wahidi S, Fouladi F, Casey S, Szwarc J, et al. How do Australian maternity and early childhood health services identify and respond to the settlement experience and social context of refugee background families?. BMC Pregnancy & Childbirth 2014;14:348.	The aims of the study were clearly stated and its importance and relevance articulated. Qualitative methodology is appropriate to explore experiences of Afghan parents accessing maternity services and health professional's views on/experiences of identification of refugee background.	Authors state that the methods were informed by community and service provider consultations.	30 Afghan parents, 10 midwives, 5 medical practitioners, 19 Community based health professionals. Afghan men/women: "Purposive recruitment methods and multiple initial contacts were used to optimise recruitment and ensure diversity of potential participants." Inclusion criteria was women and men born in Afghanistan ≥ 18 years old and had a baby that was around 4-12 months old. Also an element of convenience sample. "A postcard with information about the study and details about how to take part, in Dari and English, was distributed to local groups and services, and the postcard was printed in the Afghan community newspaper. Potential participants were provided with a telephone number to contact the community researchers to register their interest in participating in an interview." No discussion around non-participation. Health professionals: Purposive sample. Key informants invited to participate after identification by researchers, with further participants identified through initial participants. All provided care for	Afghan parents: Semi-structured interviews were conducted by community researchers. Setting of data collection was described. Participants were given a choice of location and language preference for interview No justification given for methods or setting of data collection. Interview schedule was designed based in information from a previous community consultation. Areas on enquiry are described. Authors report that the interview schedule was modified after piloting with 6 participants. Interviews were recorded on audio tape. Those conducted in Afghan language (80%) were translated into English and transcribed by community researchers. Data saturation not discussed. Health professionals: A mixture of focus groups and interviews were used with the majority being conducted by one author (ER); one was conducted by another author (JY). The setting of interviews/focus groups not reported. No justification given for methods or setting of	The researcher's role and potential bias in the formulation of questions or data collection was not discussed.	Afghan parents: Potential participants were provided with verbal information and given a copy of the study information in Dari or English and were asked to consent in writing or verbally. Confidentiality, or how issues raised in the study for participants were handled by researchers, are not discussed. Health professionals: Lacking detail about how the research was explained to these participants. No discussion around consent, confidentiality or how issues raised through the study were handled by researchers. "The project was approved by the research ethics committees of the Victorian Foundation for Survivors of Torture and the Royal Children's Hospital."	A thematic analysis approach was taken and the analysis process is described for analysing qualitative data from Afghan parents and health professionals. Afghans: "Analysis began after the first three interviews with women which were coded, informing the coding manual. A coding manual was developed using some a priori codes from the interview schedule; an iterative process was used to add additional codes to the manual (undertaken by ER, JY, FF,SW). This coding manual was used to code all women and men's interviews. JY and ER cross-checked the coding of all interview transcripts, providing an opportunity to discuss differences in the interpretation of the data. Codes were then grouped into logical categories which then provided the overarching themes." Health professionals: "All transcripts were read (by ER, JY) and imported and stored in NVivo10 [26]. Coding and categorising of data was undertaken (by ER), and key themes identified." Authors state that the paper does not report all the themes. Quotations were selected to illustrate the themes identified in the analysis. Sufficient data were presented to support the findings. Contradictory data were taken into account. No examination of researcher's role, potential bias and influence	The findings are explicit and discussed in relation to the original research question. Authors discuss the findings in relation to the wider research literature. Authors discuss the strength of having two components of the study - afghan community and health professionals. Thematic analysis of afghan participant data involved multiple analysts. Analysis of health professional data was primarily completed by one author.	Authors discuss the contribution of the study to existing knowledge and understanding. Further areas for research are not discussed. The transferability of the results is discussed. The authors acknowledge that this study included one community group in one region of Melbourne, so may not be generalisable to other groups. However, authors suggest that the stories told here may resonate with other groups in other settings.

For peer review only - http://bmjopen.bmj.com/site/about/guidelines.xhtml

Study reference*	Aims & methods	Research design	Sampling†	Data collection	Reflexivity	Ethical issues	Data analysis†	Discussion of findings	Value
			families of refugee background.<br>No discussion around non-participation.	data collection.<br>An interview schedule was used with areas of enquiry described.<br>Interviews were digitally recorded and transcribed by an outside agency.<br>Data saturation was not discussed.			during the analysis and in presentation of the data.		
Yelland J, Riggs E, Szwarc J, Casey S, Duell-Piening P, Chesters D, et al. Compromised communication: a qualitative study exploring Afghan families and health professionals' experience of interpreting support in Australian maternity care. BMJ Qual Saf 2016 Apr;25(4):e1-2014-003837. Epub 2015 Jun 18	Aims of the study were clearly stated and its importance and relevance articulated.<br>Qualitative methodology is appropriate to explore experiences of Afghan parents and health professionals.	Research methodology not explicitly justified, but authors state that the data collection methods were informed by consultation with Afghan community members and health professionals working in the area.	Afghan women and men:<br>Potential participants were identified through consultation with community groups, community leaders and the project's advisory group.<br>Not clear how individuals were approached.<br>Inclusion criteria was women and men born in Afghanistan ≥ 18 years old and had a baby that was around 4-12 months old.<br>No discussion around non-participation<br>Heath professionals:<br>Mixed purposive/convenience sample. The research was promoted within organisations that health professionals worked.<br>Those interested in participating responding.<br>Others personally recommended by key stakeholders.<br>Participants were eligible if they had provided services to Afghan families.<br>No discussion around non-participation.	Afghan participants:<br>Interviews were used to collect data and were conducted by Afghan background researchers (one woman, one man).<br>The setting of data collection was not described.<br>No justification given for methods or setting of data collection.<br>An interview schedule that had been developed with input from a previous population-based survey and was translated into Dari and piloted with 6 community members. Areas of enquiry are described.<br>Interviews were audio-taped and transcribed into English.<br>Data saturation was not discussed.<br>Health professionals:<br>Focus groups and interviews were used, but the setting or the interviewer(s) for data collection are not described.<br>An interview schedule was used and areas of enquiry are described.<br>No justification given for methods or setting of data collection.<br>Interviews were digitally recorded and transcribed by an external agency.<br>Data saturation was not discussed.	No explicit critical examination of the researcher's role and potential bias in formulating the research question or data collection. The Authors employed a participatory approach, which enhanced their capability to engage with the community (involved community members in recruitment and conducting interviews)	Afghan participants:<br>Lacking details on how the research was explained to potential participants.<br>Permission was given for audio-recording, but unclear whether consent was given for participation in the study.<br>No discussion of how confidentiality was maintained or how issues raised through the study were handled by researchers.<br>Health professionals:<br>Lacking details about how the research was explained to potential participants.<br>No details given about how participants consented, how confidentiality was maintained or how issues raised through the study were handled by researchers.<br>"The project was approved by the research ethics committees of the Victorian Foundation for the Survivors of Torture and The Royal Children's Hospital."	Afghan participants:<br>A thematic approach was taken. "All transcripts were coded manually by the community researchers and cross-checked (by FF, SW, ER, JY) and entered into NVivo10. Based on the completed coding of the first four transcripts (two women and two men) a coding manual was developed and used to code remaining transcripts. Discussion among the research team was done to place all codes into logical categories. From this seven, major themes were identified and the theme of 'language services and communication' is reported in this paper"<br>Health professionals:<br>Thematic approach was taken. "JY read all of the transcripts. The data were analysed thematically. All transcripts were coded using NVivo software (by ER) into practical categories and overarching themes."<br>Authors discuss how data were selected for this publication and that it does not represent all the themes, which were published elsewhere.<br>Sufficient data are presented to support the findings.<br>Contradictory data are taken into account<br>No examination of researcher's role, potential bias and influence during the analysis and in presentation of the data.	The findings are explicit and discussed in relation to the research question<br>The findings are discussed in the context of the wider literature.<br>Lacking explicit discussion of the credibility of the findings, however authors discuss the merits of using a participatory approach to engage refugees and it is apparent that more than one analyst was involved in defining themes from data from Afghan participants.	Authors discuss the contribution of the study to existing knowledge and understanding.<br>Further areas for research are not discussed.<br>The transferability of the results is discussed.<br>The authors acknowledge that this study included one community group in one region of Melbourne, so may not be generalisable to other groups. However, authors suggest that the stories told here may resonate with other groups in other settings.

For peer review only

Study reference*	Aims & methods	Research design	Sampling†	Data collection	Reflexivity	Ethical issues	Data analysis†	Discussion of findings	Value
* Direct quotations from articles in this table are presented within quotation marks.<br>† Sampling and analysis methods are as reported by the authors.									

For peer review only

ENREQ Reporting Checklist

Robertshaw et al. Challenges and facilitators for health professionals providing primary healthcare to refugees and asylum seekers in high-income countries: A systematic review and thematic synthesis of qualitative research.

No	item	Guide & Description	Included	Page
1	Aim	State the research question the synthesis addresses.	✓	5
2	Synthesis methodology	Identify the synthesis methodology or theoretical framework which underpins the synthesis, and describe the rationale for choice of methodology (e.g. meta-ethnography, thematic synthesis, critical interpretive synthesis, grounded theory synthesis, realist synthesis, meta-aggregation, meta-study, framework synthesis).	✓	6
3	Approach to searching	Indicate whether the search was pre-planned (comprehensive search strategies to seek all available studies) or iterative (to seek all available concepts until they theoretical saturation is achieved).	✓	6
4	Inclusion criteria	Specify the inclusion/exclusion criteria (e.g. in terms of population, language, year limits, type of publication, study type).	✓	7
5	Data sources	Describe the information sources used (e.g. electronic databases (MEDLINE, EMBASE, CINAHL, psycINFO, Econlit), grey literature databases (digital thesis, policy reports), relevant organisational websites, experts, information specialists, generic web searches (Google Scholar) hand searching, reference lists) and when the searches conducted; provide the rationale for using the data sources.	✓	6
6	Electronic Search strategy	Describe the literature search (e.g. provide electronic search strategies with population terms, clinical or health topic terms, experiential or social phenomena related terms, filters for qualitative research, and search limits).	✓	6
7	Study screening methods	Describe the process of study screening and sifting (e.g. title, abstract and full text review, number of independent reviewers who screened studies).	✓	6
8	Study characteristics	Present the characteristics of the included studies (e.g. year of publication, country, population, number of participants, data collection, methodology, analysis, research questions).	✓	11
9	Study selection results	Identify the number of studies screened and provide reasons for study exclusion (e,g, for comprehensive searching, provide numbers of studies screened and reasons for exclusion indicated in a figure/flowchart; for iterative searching describe reasons for study exclusion and inclusion based on modifications to the research question and/or contribution to theory development).	✓	9-10
10	Rationale for appraisal	Describe the rationale and approach used to appraise the included studies or selected findings (e.g. assessment of conduct (validity and robustness), assessment of reporting (transparency), assessment of content and utility of the findings).	✓	8
11	Appraisal items	State the tools, frameworks and criteria used to appraise the studies or selected findings (e.g. Existing tools: CASP, QARI, COREQ, Mays and Pope [25]; reviewer developed tools; describe the domains assessed: research team, study design, data analysis and interpretations, reporting).	✓	8
12	Appraisal process	Indicate whether the appraisal was conducted independently by more than one reviewer and if consensus was required.	✓	8
13	Appraisal results	Present results of the quality assessment and indicate which articles, if any, were weighted/excluded based on the assessment and give the rationale.	✓	17-18
14	Data extraction	Indicate which sections of the primary studies were analysed and how were the data extracted from the primary studies? (e.g. all text under the headings "results /conclusions" were extracted electronically and entered into a computer software).	✓	8
15	Software	State the computer software used, if any.	✓	8
16	Number of reviewers	Identify who was involved in coding and analysis.	✓	8
17	Coding	Describe the process for coding of data (e.g. line by line coding to search for concepts).	✓	8
18	Study comparison	Describe how were comparisons made within and across studies (e.g. subsequent studies were coded into pre-existing concepts, and new concepts were created when deemed necessary).	✓	8
19	Derivation of themes	Explain whether the process of deriving the themes or constructs was inductive or deductive.	✓	8
20	Quotations	Provide quotations from the primary studies to illustrate themes/constructs, and identify whether the quotations were participant quotations of the author's interpretation.	✓	22-23
21	Synthesis output	Present rich, compelling and useful results that go beyond a summary of the primary studies (e.g. new interpretation, models of evidence, conceptual models, analytical framework, development of a new theory or construct).	✓	19-31

This checklist was taken from Tong et al. BMC Medical Research Methodology 2012, 12:181

For peer review only

BMJ Open

Challenges and Facilitators for Health Professionals Providing Primary Healthcare for Refugees and Asylum Seekers in High-Income Countries: A Systematic Review and Thematic Synthesis of Qualitative Research

Journal:	*BMJ Open*
Manuscript ID	bmjopen-2017-015981.R1
Article Type:	Research
Date Submitted by the Author:	30-Mar-2017
Complete List of Authors:	Robertshaw, Luke; University of Birmingham, Public Health, Epidemiology & Biostatistics<br>Dhesi, Surindar; University of Birmingham, School of Geography, Earth and Environmental Sciences<br>Jones, Laura; University of Birmingham, Public Health, Epidemiology & Biostatistics
**Primary Subject Heading**:	Public health
Secondary Subject Heading:	Qualitative research, Health services research, Health policy, Global health, General practice / Family practice
Keywords:	Asylum seeker, Refugee, Primary healthcare, PRIMARY CARE

SCHOLARONE™
Manuscripts

For peer review only

Challenges and Facilitators for Health Professionals Providing Primary Healthcare for Refugees and Asylum Seekers in High-Income Countries: A Systematic Review and Thematic Synthesis of Qualitative Research

Corresponding Author:

Dr Laura L Jones, Institute for Applied Health Research, University of Birmingham, Birmingham, UK

l.l.jones@bham.ac.uk

+44 (0)121 414 3024

First Author:

Luke Robertshaw, Institute for Applied Health Research, University of Birmingham, Birmingham, UK

Second Author:

Surindar Dhesi, School of Geography, Earth and Environmental Sciences, University of Birmingham, Birmingham, UK

Third Author:

Dr Laura L Jones, Institute for Applied Health Research, University of Birmingham, Birmingham, UK

Word count: Abstract 234, Main article: 6246

For peer review only

Abstract

Objectives: To thematically synthesise primary qualitative studies that explore challenges and facilitators for health professionals providing primary healthcare for refugees and asylum seekers in high-income countries.

Design: Systematic review and qualitative thematic synthesis.

Methods: Searches of MEDLINE, EMBASE, PsycINFO, CINAHL and Web of Science. Search terms were combined for qualitative research, primary healthcare professionals, refugees and asylum seekers, and were supplemented by searches of reference lists and citations. Study selection was conducted by two researchers using pre-specified selection criteria. Data extraction and quality assessment using the CASP tool was conducted by the first author. A thematic synthesis was undertaken to develop descriptive themes and analytical constructs.

Results: Twenty-six articles reporting on 21 studies and involving 357 participants were included. Eleven descriptive themes were interpreted, embedded within three analytical constructs: Healthcare encounter (trusting relationship, communication, cultural understanding, health and social conditions, time); Healthcare system (training and guidance, professional support, connecting with other services, organisation, resources and capacity); Asylum and resettlement. Challenges and facilitators were described within these themes.

Conclusions: A range of challenges and facilitators have been identified for health professionals providing primary healthcare for refugees and asylum seekers that are experienced in the dimensions of the healthcare encounter, the healthcare system and wider asylum and resettlement situation. Comprehensive understanding of these challenges and facilitators is important to shape policy, improve the quality of services and provide more equitable health services for this vulnerable group.

For peer review only - http://bmjopen.bmj.com/site/about/guidelines.xhtml

Strengths and limitations of this study

- This is the first review to systematically identify and synthesise qualitative research exploring challenges and facilitators for health professionals providing primary healthcare for refugees and asylum seekers.
- Thematic synthesis of studies from a range of countries and primary healthcare settings allows identification of common, generalisable themes with potential to influence policy and practice.
- The review was limited to English language studies, which may have led to over-representation of studies conducted in English-speaking high-income countries.
- The review was limited to core, clinical health professionals: doctors nurses and midwives.

Background and introduction

Throughout human history, countless people have been forced to flee from their homes and countries due to violence or threats of violence. Other nations may provide refuge for those seeking a safe haven, and in 1950, the Office of the United Nations High Commissioner for Refugees (UNHCR) was established to provide international leadership and coordination for the protection of refugees and promotion of their wellbeing.[1] The UNHCR convention defines refugees as persons who have a "well-founded fear of being persecuted for reasons of race, religion, nationality, membership of a particular social group or political opinion, is outside the country of his nationality and is unable or, owing to such fear, is unwilling to avail himself of the protection of that country; or who, not having a nationality and being outside the country of his former habitual residence as a result of such events, is unable or, owing to such fear, is unwilling to return to it."[2] Those in the application process to be granted refugee status are referred to as 'asylum seekers'. By the end of 2015 there were an estimated 65.3 million forcibly displaced people worldwide, including

For peer review only - http://bmjopen.bmj.com/site/about/guidelines.xhtml

40.8 million internally displaced people, 21.3 million refugees and 3.2 million asylum seekers.[3]

Refugees and asylum seekers are a vulnerable group with significant and complex health needs.[4] A survey by the UK Border Agency in 2010 showed refugees to be in poorer health than the general population.[5] As most refugees and asylum seekers originate from low-mid income countries, there are, accordingly, higher prevalences of pre-existing infectious diseases such as Hepatitis B, TB and HIV compared to host populations.[6] The risk of contracting infectious diseases may be increased by poor hygiene conditions during flight from conflict, coupled with insufficient vaccine coverage.[7] Studies have also highlighted the sexual and reproductive health needs of this group,[8] with high levels of sexual gender based violence (SGBV) being reported along with limited access to contraception.[8, 9] Refugees and asylum seekers also suffer from non-communicable diseases such as hypertension, musculoskeletal disease, chronic respiratory disease and diabetes, which may be under-managed and exacerbated when they are forced to flee their countries.[10]

A further concern for refugee and asylum seeker populations is their mental health. Violence experienced in countries of origin, including war, sexual abuse and torture are reported, that may lead to psychological and physical trauma.[11] These pre-migration traumas are compounded by post-migration stressors such as loss of social networks, shifting societal roles and cross-cultural stress while integrating into countries of settlement.[12] Fazel et al [13] estimated that 9% of adult refugees may suffer with post-traumatic stress disorder (PTSD), which is approximately ten times estimates in an age-matched American population.[13]

Primary healthcare teams are on the front-line of healthcare provision for refugees and asylum seekers that arrive in high-income countries.[14] These teams may include a variety of professional backgrounds, clinical and non-clinical, but typically include a core of general practitioners, community based nurses and midwives.[15, 16] These health professionals face significant challenges when caring for refugees and asylum seekers.[17-19] They must

For peer review only - http://bmjopen.bmj.com/site/about/guidelines.xhtml

address their complex health and social needs, often in cross-cultural interactions, and operate within health systems that may not be structurally configured or politically favourable towards this group.[17-20] These challenges impact on their ability to provide the same quality of care as the general population, leading to healthcare inequalities.[20, 21]

Experiences of health professionals caring for refugees and asylum seekers in high-income countries have been investigated through a range of qualitative research studies conducted across several countries and primary healthcare settings. A recent systematic review by Suphanchaimat et al [22] synthesised challenges providing healthcare services to migrants from a provider perspective. The review included a minority of studies that had refugees and asylum seekers as service users, focussed purely on challenges of healthcare provision, and adopted a limited, purposive search strategy. To our knowledge, this present review is the first to synthesise experiences of health provision for migrants defined specifically as refugees and asylum seekers; synthesise both challenges and facilitators for health professionals; and adopt a systematic approach to identification of qualitative research. Therefore, this review aims to systematically identify and thematically synthesise challenges and facilitators experienced by health professionals that provide primary healthcare for refugees and asylum seekers in high-income countries.

For peer review only

For peer review only - http://bmjopen.bmj.com/site/about/guidelines.xhtml

Methods

This systematic review sought qualitative research studies as they are the appropriate design for understanding perceptions and experiences of healthcare provision.[23, 24] Systematic identification and synthesis of these studies may consolidate the current evidence-base, increase the breadth and depth of understanding and provide more generalisable conclusions than individual primary studies.[25, 26]

This review was guided by established methodology for systematic review and thematic synthesis of qualitative research, outlined by Thomas and Harden.[27] Thematic synthesis of data, applied in this methodology, is suited to development of recommendations for practice and policy and provides a transparent link between conclusions and the primary studies synthesised.[27, 28] Reporting of this review has been guided by Enhancing Transparency of Reporting the Synthesis of Qualitative Research (ENTREQ) framework.[29]

Search strategy

The following databases were searched from inception until week 3 of March 2016: MEDLINE, EMBASE, PsycINFO, CINAHL and Web of Science. The search strategy was based on the SPIDER (Sample, Phenomenon of interest, Design, Evaluation, Research type) tool, which has been developed as an alternative to PICO (Population, Intervention, Comparison, Outcome) to optimise identification of qualitative studies for evidence syntheses.[30] Search terms were combined for primary health professionals/healthcare, refugees and asylum seekers, and qualitative research. No language or date limits were applied. The full detailed search strategy is documented in online supplement 1. Further hand-searches were conducted based on included studies' reference lists and citations (in Google Scholar).

After removal of duplicates, titles and abstracts were screened by one researcher (LR), excluding articles that clearly did not meet the inclusion criteria. Full-texts of remaining articles were obtained and assessed by two independent researchers, according to pre-specified study selection criteria (detailed below). Disagreements were resolved via discussion.

For peer review only - http://bmjopen.bmj.com/site/about/guidelines.xhtml

Selection criteria

This review included peer reviewed, qualitative primary research studies that met the following criteria: English language; explored challenges or facilitators (defined in Box 1) for health professionals providing primary healthcare to refugees and asylum seekers (including forced migrants, involuntary migrants or refugee claimants); and were conducted in a high-income country as defined by the World Bank country classification 2015.[31] Studies were limited to those from high-income countries because of the authors' interest in the developing of recommendations for policy and practice applicable to advanced primary healthcare systems.

Box 1: Definitions of challenge and facilitator

Challenge: A factor that inhibits, obstructs or creates difficulties for health professionals when providing primary healthcare.

Facilitator: A factor that promotes, enables or assists health professionals when providing primary healthcare

Mixed-methods studies were included if the qualitative element's methods and results could be isolated for synthesis. As definitions of health professionals in primary healthcare teams are diverse, [16] this review was limited to articles that interviewed core clinical healthcare professionals including: general practitioners, nurses, pharmacists and midwives working in primary healthcare settings. Articles were excluded if: they were not based on peer reviewed primary qualitative studies (i.e. reviews, case studies, reports, opinion pieces); or were conducted in a secondary care setting. Articles that had referred to service users as 'migrants' or 'immigrants' were excluded, as these terms have a broader meaning including economic migrants, students and family unification.[32] Those that referred to 'illegal immigrants' or 'undocumented migrants' were also excluded as they are known to have unique characteristics (e.g ineligible for free healthcare) that would not be typical of refugees and

For peer review only - http://bmjopen.bmj.com/site/about/guidelines.xhtml

asylum seekers.[33] Articles interviewing mental health professionals were excluded as this clinical area has specific characteristics. Where studies contained a mixture of eligible and ineligible participants, they were only included if data for eligible participants could be isolated for synthesis. Studies were also excluded if the full text articles could not be obtained through institutional access or requests sent to authors through Research Gate. The full inclusion and exclusion criteria applied in this review are documented in online supplement 2.

Data extraction

Study characteristics were extracted by one author (LR) using a data extraction proforma. Characteristics included aims, setting, participants, methodology, results and recommendations/applications. Findings (results) and discussion sections from included articles were imported into NVivo 11 software (NVivo qualitative data analysis Software; QSR International Pty Ltd. Version 11, 2016) for analysis.

Assessment of quality

Included articles were assessed by one author (LR) using the Critical Appraisal Skills Programme (CASP) tool for appraisal of qualitative research.[34] Articles were not excluded from the synthesis or given weighting based on this assessment as there is currently no accepted method for this in syntheses of qualitative research.[35] All articles were included irrespective of their reporting quality given that they contributed to the conceptual richness of the synthesis. Where articles used mixed-methods, only the qualitative element was appraised.

Data synthesis

A thematic synthesis was conducted broadly following the methodology outlined by Thomas and Harden.[27] An article, considered data-rich (containing numerous challenges and facilitators), was selected as an index-article and uploaded into NVivo 11 software. The findings (results) and discussion sections were coded inductively within the two categories of 'challenges' and 'facilitators', as defined by the review question. This approach of inductive coding within a

For peer review only - http://bmjopen.bmj.com/site/about/guidelines.xhtml

priori categories follows established methodology seen in similar qualitative syntheses.[36] Primary quotations, author's commentary and author's interpretations were coded. Sections were only coded if they contained challenges or facilitators (Box 1), and referred to the health professionals defined for this review. Following the index-article, subsequent articles were coded using the same method in approximate order of descending data-richness. Concepts in each article were coded to iteratively develop and refine a codebook, with each article having an ability to contribute new codes. Once all articles had been coded, the finalised codebook was applied across all articles. The final codebook was analysed to inform descriptive themes closely resembling the prevailing concepts across primary studies. These themes were discussed and agreed within the research team. An analytical model was then developed to create higher-order constructs within which descriptive themes were located.

For peer review only

For peer review only - http://bmjopen.bmj.com/site/about/guidelines.xhtml

Results

Systematic search and selection

Systematic database searches identified 5970 articles. A further 16 articles were identified through hand-searching of reference lists and citations. After removal of duplicates, 3571 articles remained. 3493 articles were excluded based on the title and abstract. Full-texts of the remaining 78 articles were sought for detailed assessment against the inclusion criteria. Nine of these articles could not be obtained. In addition, due to resource limitations, four non-English language studies were unable to be translated and assessed against the selection criteria. After reviewing the 65 available full-text papers and applying the full selection criteria, 26 articles were included in the thematic synthesis (Figure 1).

For peer review only

For peer review only - http://bmjopen.bmj.com/site/about/guidelines.xhtml

Characteristics of included studies

The 26 articles included were based on 21 primary studies of which 19 were qualitative studies [17, 18, 37-58] and two were mixed-methods.[19, 59] Nine articles were from Australia,[41, 43, 45-48, 50, 57, 58] seven from the United Kingdom,[18, 37-40, 42, 59] three from the Netherlands [44, 52, 53] and one from each of Denmark,[17] Switzerland,[19] New Zealand,[49] Sweden,[51] the United States,[54] Ireland [55] and Canada.[56] All articles were published between 1999 and 2016. Service users were described as 'refugees' in 11 articles,[17, 41, 43-49, 54, 56] 'asylum seekers' in six articles,[19, 38, 52, 53, 55, 59] 'refugees and asylum seekers' in five articles,[18, 37, 39, 40, 42] 'of refugee background' in three articles,[50, 57, 58] and 'involuntary migrants' in one article.[51]

Qualitative data extracted for this synthesis were derived from 357 participants with a combined sample of 194 nurses, 35 midwives and 128 doctors. None included pharmacists. Data collection methods varied across the 21 primary studies represented, with 14 solely using individual interviews (including in-depth, semi-structured, unstructured),[17-19, 37-40, 42, 44, 45, 47-49, 51, 55, 56, 59] One employed group interviews only,[54] and four combined individual and group interviews.[43, 46, 50, 57, 58] One study used observational methods and individual interviews,[41] and one combined group interviews and qualitative questionnaires.[52, 53] Table 1 summarises characteristics of included articles and online supplement 3 contains the complete data extraction.

For peer review only

Table 1 Characteristics of articles included in the thematic synthesis

First author	Publication year	Country	Eligible participants[a] and practice setting	Service users[b]	Data collection method	Analysis methodology	Study aims/objectives[c]
Begg, H.[37]	2005	United Kingdom	17 general practitioners<br><br>General practice	Refugees & asylum seekers	Semi-structured interviews	Thematic framework	To identify some of the concerns of 17 general Practitioners (GPs) working in an urban environment.
Bennett, S.[38]	2014	United Kingdom	10 midwives<br><br>Community, rotational, specialist and delivery suite midwives	Female asylum seekers	Semi-structured interviews	Thematic analysis	To gain an in depth analysis of the experiences of midwives and their understanding of the specific needs of asylum-seeking women. The findings would be used to inform education, practice and policy to enable more effective delivery of woman-centred care for this group locally.
Burchill, J.[39][d]	2011	United Kingdom	14 health visitors<br><br>London borough	Refugees and asylum seekers	In-depth interviews	Framework	Not clearly stated.
Burchill, J.[18][d]	2012	United Kingdom	14 health visitors<br><br>London borough	Refugees and asylum seekers	In-depth interviews	Framework	To determine the barriers to effective practice that health visitors when working with refugees and asylum seekers.
Burchill, J.[40][d]	2014	United Kingdom	14 health visitors<br><br>London borough	Refugees and asylum seekers	In-depth interviews	Framework	Explored the experiences of health visitors working with refugee and asylum-seeking families in central London, and assessed the dimensions of their cultural competency using Quickfall's model.
Carolan, M.[41]	2008	Australia	2 midwives<br><br>African women's clinic (community health centre)	Female African refugees	Observational methods and semi-structured interviews	Thematic analysis	To explore factors that facilitate or impede the uptake of antenatal care among African refugee women.
Crowley, P.[59][e]	2005	United Kingdom	10 general practitioners<br><br>General practice	Asylum seekers	Telephone interviews	Not specified	To assess the mental health care needs of adult asylum seekers in Newcastle upon Tyne.

For peer review only - http://bmjopen.bmj.com/site/about/guidelines.xhtml

First author	Publication year	Country	Eligible participants[a] and practice setting	Service users[b]	Data collection method	Analysis methodology	Study aims/objectives[c]
Drennan, V.[42]	2005	United Kingdom	13 health visitors<br><br>2 London borough's	Refugees and asylum seekers	Semi-structured interviews	Framework	Describe health visitors' experiences working in Inner London and identifying and addressing the health needs of refugee woman in the first 3 months after the birth of a baby.<br>Investigate health visitors' perceptions of effective and ineffective strategies in identifying and addressing health needs of these women.<br>Investigate whether health visitors used a framework corresponding to Maslow's theory of a hierarchy of needs to prioritize their public health work.
Farley, R.[43]	2014	Australia	20 general practitioners<br>5 practice nurses<br><br>General practice	Newly arrived refugees	Focus groups and Semi-structured interviews	Thematic analysis	Explored the experiences of primary health care providers working with newly arrived refugees in Brisbane...focusing on the barriers and enablers they continue to experience in providing care to refugees.
Feldmann, C.[44]	2007	Netherlands	24 general practitioners<br><br>General practice	Refugees (Afghan/Somali)	In-depth interviews	Thematic analysis	To confront the views of refugee patients and general practitioners in the Netherlands, focusing on medically unexplained physical symptoms (MUPS).
Furler, J.[45][†]	2010	Australia	8 family physicians<br><br>Community health centre	Refugees with depression	Semi-structured interviews	Thematic analysis	This study explores the complexities of this work [clinical care for depression] through a study of how family physicians experience working with different ethnic minority communities in recognizing, understanding, and caring for patients with depression.
Griffiths, R.[46]	2003	Australia	13 nurses<br>2 nurse managers<br><br>Refugee reception centre	Refugees	Focus groups and semi-structured interviews	Thematic analysis	To identify the skills, knowledge and support nurses require to provide holistic and competent care to refugee children and their families and the nature of support that is required to assist their transition back to mainstream health services.

For peer review only - http://bmjopen.bmj.com/site/about/guidelines.xhtml

First author	Publication year	Country	Eligible participants[a] and practice setting	Service users[b]	Data collection method	Analysis methodology	Study aims/objectives[c]
Jensen, N.[17]	2013	Denmark	9 general practitioners<br><br>Medical clinics	Refugees	Semi-structured interviews	Content analysis	To qualitatively explore issues identified by general practitioners as important in their experiences of providing care for refugees with mental health problems.
Johnson, D.[47]	2008	Australia	12 general practitioners<br><br>General practice	Refugees	Semi-structured interviews	Template analysis	To document the existence and nature of challenges for GPs who do this work in SA.<br>To explore the ways in which these challenges could be reduced.<br>To discuss the policy implications of this in relation to optimising the initial health care for refugees.
Kokanovic, R.[48][†]	2010	Australia	5 general practitioners<br><br>Community health centre	Refugees with depression	In-depth interviews	Thematic analysis	We explore a set of cultural boundaries across which depression is contested: between recent migrants to Australia from East Timor and Vietnam, and their white 'Anglo' family doctors.
Kurth, E.[19][e]	2010	Switzerland	3 physicians<br>3 nurses/midwives<br><br>Women's clinic	Female asylum seekers	Semi-structured interviews	Grounded theory	To investigate the reproductive health care provided for women asylum-seekers attending the Women's Clinic of the University Hospital in the city of Basel, Switzerland. To identify the health needs of asylum seekers attending the Women's Clinic and to investigate the health care they received in a Health maintenance organisation (HMO) specifically established for asylum seekers…Explored the perceptions of the health care professionals involved, about providing health care for this group in this setting.
Lawrence, J.[49]	2005	New Zealand	5 medical practitioners<br><br>Community health centre	Refugees	In-depth interviews	Thematic analysis	This paper reports on research that sought to reveal the barriers faced by refugees in accessing health services, and the challenges faced by providers in endeavouring to meet needs in an effective and culturally appropriate manner.

For peer review only - http://bmjopen.bmj.com/site/about/guidelines.xhtml

First author	Publication year	Country	Eligible participants[a] and practice setting	Service users[b]	Data collection method	Analysis methodology	Study aims/objectives[c]
Riggs, E.[50]	2012	Australia	12 nurses<br><br>Maternal and child health services	Refugee background mothers	Focus groups and Interviews	Thematic analysis	To explore the utilisation and experience of MCH services in Melbourne, Victoria for parents of refugee background from the perspective of users and providers.
Samarasinghe, K.[51]	2010	Sweden	34 primary health care nurses<br><br>Various: Maternity, child, school, community health care, nurse-led clinics.	Involuntary migrant families	Interviews	Contextual analysis	The aim of this study was to describe the promotion of health in involuntary migrant families in cultural transition as conceptualized by Swedish PHCNs.
Suurmond, J.[53][g]	2013	Netherlands	36 nurse practitioners<br>10 public health physicians<br><br>Asylum seeker centres	Newly arrived asylum seekers	Group interviews	Framework	To describe the tacit knowledge of Dutch healthcare providers about the care to newly arrived asylum seekers and to give insight into the specific issues that healthcare providers need to address in the first contacts with newly arrived asylum seekers.
Suurmond, J.[52][g]	2010	Netherlands	89 nurse practitioners (questionnaires)<br>36 nurse practitioners (group interviews)<br><br>Asylum seeker centres	Asylum seekers	Questionnaires and group interviews	Framework	We explored the cultural competences that nurse practitioners working with asylum seekers thought were important.
Tellep, T.[54]	2001	United States	6 school nurses<br><br>Schools	Refugees	Focus group	Unspecified	To describe the nature and meaning of school nurses' and Cambodian liaisons' experiences of caring for Cambodian refugee children and families and to explore whether those meanings validated Dobson's conceptual framework of transcultural health visiting.

For peer review only - http://bmjopen.bmj.com/site/about/guidelines.xhtml

First author	Publication year	Country	Eligible participants[a] and practice setting	Service users[b]	Data collection method	Analysis methodology	Study aims/objectives[c]
Tobin, C.[55]	2014	Ireland	10 midwives<br><br>Maternity hospitals	Female asylum seekers	In depth unstructured interviews	Content analysis	To explore midwives' perceptions and experiences of providing care to women in the asylum process and to gain insight into how midwives can be equipped and supported to provide more effective care to this group in the future.
Twohig, P.[56]	1999	Canada	6 family practice nurses<br>10 family physicians<br><br>Clinic at refugee processing centre	Refugees	Semi-structured interviews	Textual analysis	To explore roles of family physicians and family practice nurses who provided care to Kosovar refugees at Greenwood, NS.
Yelland, J.[57][h]	2014	Australia	10 Midwives<br><br>Maternity services	Refugee background families	Interviews and focus groups	Thematic analysis	(1) investigate Afghan women and men's experience of the way that health professionals approach inquiry about social factors affecting families having a baby in a new country, and (2) investigate how health professionals identify and respond to the settlement experience and social context of families of refugee background.
Yelland, J.[58][h]	2016	Australia	10 Midwives<br><br>Maternity services	Refugee background families	Interviews and focus groups	Thematic analysis	(1) describe Afghan women's and men's experiences of language support during pregnancy check-ups, labour and birth; (2) explore health professionals' experiences of communicating with Afghan and other refugee clients with low English proficiency; and (3) consider implications for health services and health policy.

[a]Some studies included some participants not eligible for this review. These participants have not been included on this table.
[b]Service users as described by the authors.
[c]The aims and objectives are from the author (i.e. extracted directly from papers.)
[d,h]These articles were based on data from the same sample, but reported different aspects.
[e]Mixed-methods were utilised in these studies. This table only includes characteristics of the qualitative element relevant to this review.
[f]The 5 GP's in Kokanovic 2010 are included within the 8 physicians in Furler 2010 but report different aspects.
[g]The 36 nurse practitioners are common between articles, but report different aspects.

For peer review only - http://bmjopen.bmj.com/site/about/guidelines.xhtml

Quality assessment

Application of the CASP critical appraisal tool revealed variable results across the 26 articles assessed. All except one article [39] gave a clear statement of the research aims. The majority (21 articles) [17-19, 37-40, 42-44, 46, 48, 49, 51-58] sufficiently described the sampling strategy and provided some rationale for participants' selection. Possible reasons for non-participation were discussed in only four articles.[19, 37, 46, 47] The data collection method was stated in all articles, however the extent of information provided about interview schedule's content was variable. A significant number did not describe the setting of data collection (13 articles) [38, 43-47, 50, 52, 53, 56-59] or the identities of interviewers (12 articles).[18, 19, 38-42, 46, 47, 55, 58, 59] Only eight articles [43, 47-50, 52, 56, 59] gave justification for chosen data collection methods or interview settings. Data saturation was rarely discussed, featuring in five articles.[37, 43, 47, 48, 56]

Reflexivity was particularly poorly discussed across articles. Only seven [37, 39, 43, 48, 51, 54, 55] reflected on potential bias and influence of researchers at any stage in the study (formulation of review question, sampling, data collection or analysis).

Ethical approval was described in the majority of articles (23 articles),[17-19, 37-43, 45-48, 50-58] but they often lacked sufficient information to judge whether ethical standards had been followed. Thirteen articles [17-19, 38-43, 48, 51, 53, 55] described how participants were informed about the nature and purpose of the study, 17 articles [17, 19, 37, 38, 40, 42, 43, 48-56, 60] described obtaining consent and 12 articles [17, 37, 41-43, 46, 47, 51-55] discussed how confidentiality was assured or maintained.

The approach to data analysis was described to some extent in all but one article,[59] however there was variation in the level of detail given. Involvement of multiple researchers in the analysis process was reported in 19 articles.[17-19, 37, 39-43, 45-48, 50, 51, 55-58] The majority (25 articles) [17-19, 37-58] gave support for findings with references to primary data (e.g. quotations from participants). Findings were generally clearly presented and discussed in

For peer review only - http://bmjopen.bmj.com/site/about/guidelines.xhtml

context of wider research literature, policy and practice, although a few (six articles) [39, 40, 42, 49, 54, 56] were limited in this area. Ten articles [19, 37, 41, 43, 45, 50-53, 57] explicitly reflected on the credibility of their findings.

Full details of the CASP assessment are provided in online supplement 4.

For peer review only

For peer review only - http://bmjopen.bmj.com/site/about/guidelines.xhtml

Thematic synthesis findings

Challenges and facilitators for health professionals providing primary healthcare to refugees and asylum seekers were interpreted within 11 descriptive themes, embedded in 3 analytical constructs: healthcare encounter (trusting relationship, communication, cultural understanding, health and social conditions, time), healthcare system (training and guidance, professional support, connecting with other services, organisation, resourcing and capacity), and asylum and resettlement. Figure 2 illustrates the relationships between analytical constructs and descriptive themes. Healthcare encounters occur within the environment of healthcare systems, both of which operate within wider asylum and resettlement policies and processes. Table 2 provides a taxonomy of challenges and facilitators and Table 3 contains illustrative quotations from primary studies for each descriptive theme.

For peer review only - http://bmjopen.bmj.com/site/about/guidelines.xhtml

Table 2 Taxonomy of challenges and facilitators

Analytical construct	Descriptive theme	Challenges	Facilitators
Healthcare encounter	Trusting relationship	-Transience of refugees/ asylum seekers<br>-Suspicion of authorities	-Continuity of care<br>-Assisting with wider needs<br>-Taking an interest<br>-Compassion/empathy<br>-Explaining role
	Communication	-Language: assessing case history/gaining consent/ ensuring patient understanding<br>-Interpreters: additional time/ expense, unavailability, inaccuracy/imposition of own views<br>-Telephone interpreters: impersonal, technological failures<br>-Illiteracy<br>-Lack of language specific resources	-Interpreters: professionally trained, continuity<br>-Telephone interpreters: increased availability<br>-Visual aides
	Cultural understanding	-Different understandings of health concepts/terminology/ healthcare systems<br>-Understanding patient's symptoms<br>-High expectations of patients<br>-Different cultural values	-Knowledge of other cultures: values, health practices, body language<br>-Personal qualities - sensitivity, empathy, cultural humility
	Health and social conditions	- Physical: communicable diseases, FGM, Injuries<br>-Unusual diseases<br>-Psychological: torture, abuse, social difficulties, somatisation<br>-Lacking skills, knowledge, support	-Training<br>-Guidance<br>-Professional support<br>-History taking
	Time	-Increased time requirement<br>-Increased duration/occurrences of appointments<br>-Insufficient time – rushed appointments<br>-Time taken away from other patient groups	

For peer review only - http://bmjopen.bmj.com/site/about/guidelines.xhtml

Table 2 (continued)

Analytical construct	Descriptive theme	Challenge	Facilitator
The healthcare system	Training and guidance	-Lack of training/guidance<br>-Lack of awareness of available resources<br>-Time constraints	-Cultural competency training<br>-Orientation to services/resources/asylum process<br>-Culture specific information
	Professional support	-Deficiency of professional support<br>-Supporting traumatised patients without support<br>-Isolation	
	Connecting with other services	-Referral difficulties; services not present/not suitable<br>-Difficulty understanding/ navigating healthcare system	-Establishing referral pathways – health system/civil society<br>-Accompanying refugees and asylum seekers<br>-Communication/coordination /collaboration with other services<br>-Co-delivery of services<br>-Multi-agency teams
	Organisation		-Flexibility of primary healthcare system: innovation/adaptation<br>-Specialised services
	Resourcing and capacity	-Increased costs<br>-Funding shortages<br>-Workforce shortages<br>-Inflexibility/unsuitability of interpreter services	
Asylum and resettlement	Asylum and resettlement	-Policy restrictions<br>-Conflicts of interest<br>-Understanding changing policy environment and healthcare provisions<br>-Perceived abuses of system	-Training in asylum and resettlement policy/process

For peer review only - http://bmjopen.bmj.com/site/about/guidelines.xhtml

Table 3 Illustrative quotations

Theme		Quotation and reference[a]
**Healthcare encounter**		
Trusting relationship	Challenge:	'… you put your mind around trying to sort things out, the dreadful things that have happened to them, and then the next week it will be a different family there and you start the whole process all over again, trying to build up some sort of trust…'[42]
	Facilitator:	'Creating trust is an important aspect, to show that you are interested in the person, not only in the disease; to show that you want to know something about the context. Sometimes it is difficult to find time for it in a busy practice, but I see it is a worthwhile investment'.[44]
Communication	Challenge:	'I've had some pretty bad examples recently of interpreters where they have actually started giving their opinion, which has been a nightmare, …they start adding their points of view.'[40]<br>'The phone interpreter is too impersonal. And I found that a lot of them use mobile phones so you're constantly cutting out...'[50]
	Facilitator:	'Everything comes down to communication. To know what's going on, what they need, what you need, because it's a partnership, isn't it?' [38]<br>'... this [telephone interpreting] is available 24 hours and is instantaneous ... it's revolutionised, all the doctors use it, the receptionists, the nurses….' [37]
Cultural understanding	Challenge:	'…they have a different culture, so their cultural perception of symptoms and what they mean . . . trying to interpret the difference between a bloated abdomen and a painful abdomen, just becomes an impossible task.'[43]<br>'I sometimes say, 'I am only a doctor'. Sometimes there are far greater expectations than what you can honour'[17]<br>'I think most people understand sadness…but in some cultures, they don't understand [depression] as a condition that requires treatment'[45]
	Facilitator:	'…there were specialized nurses who had worked overseas, who gave workshops for us, and explained much of the history, and explained some of the conflicts which they bring over here.'[54]
Health and social conditions	Challenge:	'I am quite overwhelmed at times as to how complex these ladies' lives are….'[38]<br>'I guess it is out of our comfort zone, because our medical experience doesn't include the exotic illnesses that they front up with...'[47]<br>'Midwives spoke of the emotional impact of working with women with trauma histories: "How does it affect me, you just feel sad you know, but you just do the best that you can and that's all you can do'[55]
	Facilitator:	'[Specialist team teaching sessions] is the sort of thing that people need to help give them a baseline of knowledge, and I suppose, the support to realise that there are other people they can talk to, to help them and signpost, or help them to signpost their clients in the right direction.'[40]<br>'We don't need to know the whole lot; we don't need the whole case history […] to have a bit more understanding.'[38]
Time	Challenge	'... generally speaking a consultation with a refugee will take twice as long [as with] a local patient.'[37]<br>'…providing care with interpreters was more time consuming than without, meaning that midwives had to 'juggle their time' to facilitate good care.'[38]

For peer review only

Table 3 (continued)

Theme		Quotation and reference[a]
**Healthcare system**		
Organisation	Facilitator	'The flexibility of the general practice setting enabled providers to act on their commitment to provide refugee health care, allowing them to be responsive and innovative in their approach to caring for refugees and also providing flexibility in the hours they work.'[43] Participants felt that significant gains had been made to the refugee health care system, with the establishment of a specialised service. One provider working in the field for some time described thinking, *'. . . fantastic, finally'[43]*
Resourcing and capacity	Challenge	*'... [asylum seekers] should be budgeted for ... they're actually slightly harder work than somebody else [this] needs to be acknowledged.'[37]* *'But I was more angry that I just needed more hands to help. So, for me it was about practical support.'[46]*
Training and guidance	Challenge	*'Even when we called . . . the* [Division of General Practice] *. . . they didn't know how to guide us . . . I think we didn't have a guideline ...*'[43]
	Facilitator	'The specialist team facilitated a rolling programme of training for frontline staff working with refugees and asylum seekers, and this was regarded as an effective way of sharing knowledge.'[39]
Professional support	Challenge	'…lack of institutional support all contributed to varying feelings of powerlessness on the part of the midwives themselves.'[55]
	Facilitator	'They described the value of currently available external supports, including language classes, translation and interpreting services, and specialised refugee health services, particularly in the area of mental health.'[43]
Connecting with other services	Challenges	*'She explained she had seen a lot of problems…I put her touch with a voluntary [nationality specific] counselling organization to then discover she had to pay and she can't afford it.'[42]*
	Facilitator	*'so I referred her to ... and we went together for a joint meeting ... FORWARD [a women's campaign and support charity] specialises in FGM and I set her up for an appointment there and she was referred to a specialist nurse ... who was able to look at potentially reversing part of the FGM and the client was happy for this to happen and actually did attend.*'[40]
**Asylum and resettlement**		
	Challenge	*'These requirements differed: on the one hand to be the care giver, to be the patient's advocate in fact, and on the other to act as advocate of the Federal Office for Refugees, and thirdly to be responsible for the organisation, to save costs for the health insurance. But that is simply not possible.'[19]* *'I don't know if there is some sort of system that they go through, or some sort of protocol that they, medically, have to go through before they are granted visas...'[47]*

[a] Participant's quotations are in italics, study authors text is normal typeface.

For peer review only - http://bmjopen.bmj.com/site/about/guidelines.xhtml

The healthcare encounter

Challenges and facilitators for healthcare provision to refugees and asylum seekers were experienced within the healthcare encounter. This is the milieu of personal engagement between health professionals and service users. Five inter-related factors influenced health professionals' practice: Trusting relationship, communication, cultural understanding, health and social conditions, and time.

Trusting relationship

Building trusting relationships with refugees or asylum seekers featured in 15 of the articles.[18, 40-42, 44-46, 48, 50-55, 57] Facilitators included; continuity of the attending care provider;[42, 50, 52, 54, 57] taking an active interest in their background, language and culture;[40, 44, 54, 57] and assisting them with their wider needs.[18, 40, 50] Having a compassionate and empathetic disposition was also seen as important in relationship building.[41, 51, 52, 55, 57] The transient nature of some service users made building relationships challenging [42] and trust was threatened when refugees or asylum seekers thought that healthcare professionals were associated with immigration authorities.[38, 42, 52] Health professionals found that clearly explaining their role and confidentiality brought reassurance and allayed suspicions.[42, 52] Some benefits of establishing trusting relationships were said to be increased engagement with the healthcare service by refugees and asylum seekers [18, 40, 41, 50] and greater levels of disclosure about their health and social concerns.[42, 48, 50, 52, 57]

Communication

Communication was a theme found in 22 included articles.[17-19, 37, 38, 40, 42-45, 47-58] The language barrier was widely cited as challenging while caring for refugees and asylum seekers.[17-19, 37, 38, 40, 43, 44, 47-51, 53-58] Individual articles elaborated that language barriers presented difficulties in assessing case histories,[19] gaining consent [55] and ensuring patients understood treatment.[42]

For peer review only - http://bmjopen.bmj.com/site/about/guidelines.xhtml

Utilising interpreters was considered a major facilitator in communication [17, 37, 38, 40, 43, 45, 50, 51, 57] and was maximised when interpreters were well-trained and familiar with medical terminology.[17, 45] Continuity of the interpreter was deemed important in fostering good communication and increased confidence in the integrity of translation.[38, 40, 45, 50] There were, however, challenges associated with interpreter use.[17-19, 37, 38, 40, 42, 43, 45, 47, 49, 50, 52, 55, 56, 58] Communicating through interpreters required additional time [38, 47] and financial expense.[55] Suitable interpreters were not always available at the appropriate time,[17, 38, 42, 43, 47, 55] which could lead to delayed, extended or rearranged appointments.[17, 38, 47] This led, in some cases, to family or other community members being asked to translate instead of professional interpreters.[42, 55] Participants were also concerned that interpreters did not always accurately communicate [37, 40, 43, 45, 55, 56] and may impose their own views.[40, 43] The use of telephone interpreters received mixed opinions. Advocates welcomed the increased availability of interpreters at any time of the day,[37] but others felt they were more impersonal [50, 58] and pointed to technological failures that hindered communication.[50, 58]

Further communication challenges included unavailability of written health information in service users' languages [53, 57] and in some cases patients were unable to read or write.[43] To improve communication with those with limited language skills, some participants used objects or other visual aids.[51]

Cultural understanding

Cultural understanding was a theme described across 21 articles.[17, 18, 37, 38, 40-49, 51-57] Healthcare provision could be challenging, when there were different understandings of health, illness or healthcare.[17, 18, 40, 44-49, 51, 53, 55] Health literacy could be limited [43, 47, 53] and different terms could be used to refer to health conditions.[18, 45, 48, 57] Healthcare concepts such as preventative care (e.g. screening),[47, 49] mental healthcare [48, 57] and self-management [51] were sometimes unfamiliar. Service users also lacked understanding host country's healthcare systems,[37, 40, 42, 43, 45, 49]

For peer review only - http://bmjopen.bmj.com/site/about/guidelines.xhtml

making them prone to miss appointments,[43] and attempt to inappropriately access services.[37]

Differences in health culture presented difficulties for health professionals' understanding of patient's symptoms [45] and required additional time and effort explaining health conditions, healthcare concepts or health systems.[42, 47, 51] It was also reported that some refugees or asylum seekers had very high, and sometimes unrealistic, expectations of health services or health professionals,[17, 37, 40, 52, 53] which needed to be counteracted by participants. [17, 53] Disparities in cultural values such as gender roles, decision-making, social taboos and time-orientation were also mentioned as challenges,[41, 47, 48, 53] with some health professionals expressing uncertainty about approaching some clinical tasks such as physical examinations.[47]

Gaining knowledge and understanding about cultures of refugees and asylum seekers was viewed as an important facilitator in cross-cultural care.[38, 40, 42, 47, 52, 54, 55, 57] This included understanding differences in values,[42] body language,[52] health practices [42] and health presentations[52]. Cultural understanding allowed health professionals to adjust their healthcare practice accordingly.[40, 45, 48, 49, 51, 55, 56] Personal qualities in health professionals that were deemed to enhance cross cultural interactions were sensitivity,[49, 52, 54] empathy [40, 41, 54] and cultural humility.[54, 55]

Health and social conditions

Health professionals spoke of challenges in dealing with physical, psychological and social problems that were typically presented by refugees and asylum seekers.[17, 37, 40, 43, 44, 46, 47, 52, 53, 55-57]

Physical conditions presented challenges[37, 40, 43, 44, 47] and included: tropical diseases such as malaria and schistosomiasis;[43] other communicable diseases such as TB and HIV;[37, 40, 44] and nutritional deficiencies.[37, 40, 44] Physical injuries were also encountered, such as female genital mutilation (FGM) [40, 55] and injuries inflicted from conflict or torture.[40] Health

For peer review only - http://bmjopen.bmj.com/site/about/guidelines.xhtml

professionals did not always feel prepared or equipped to deal with these conditions [43, 47] and there were concerns from general practitioners that some conditions could remain undiagnosed.[43, 44, 47]

Psychological conditions were considered challenging to deal with,[17, 37, 40, 43, 46, 52, 53, 55-57] and were frequently seen among refugees and asylum seekers.[37, 43] These included psychological trauma related to war,[17] torture [40, 43, 46] and other abuses.[17, 38, 40] Post-migration stresses were also perceived to impact negatively on their mental health such as the asylum and resettlement process,[17, 40, 47] social isolation,[17, 45, 55] and other social vulnerabilities.[40, 50, 57] Health professionals found engaging with these service users emotionally difficult,[37, 55] and distressing when hearing their disturbing stories.[40, 42, 46, 55] They also expressed feelings of powerlessness [17, 46, 55] believing they lacked required skills, knowledge and support to respond to their complex psychological needs.[43, 57]

A further challenge noted by health professionals across four articles was the manifestation of medically unexplained symptoms (somatisation) among some refugees and asylum seekers,[18, 43, 44, 48] which could be frustrating [43] and time consuming to address.[43, 48]

Several facilitators were identified that could help deal with complex physical and psychological conditions. Careful history-taking of medical, social and migration background was helpful [38, 44, 50, 53, 57] and could identify possible risk-factors.[53, 57] Training in conditions common among refugees and asylum seekers was deemed valuable,[37, 38, 40, 46, 52, 53, 55] increasing confidence in care delivery [40] and resulting in 'more effective, evidence based care'.[38] Clinical guidelines for refugee healthcare were considered beneficial [37, 47] although these were often unavailable.[37, 47] Professional support was regarded as a facilitator,[37, 38, 42, 43, 46, 51, 55] provided within services [42] or from external organisations specialising in refugee healthcare.[43, 46] The importance of psychological support for those working with traumatised patients was highlighted,[46, 51, 55] such as

For peer review only - http://bmjopen.bmj.com/site/about/guidelines.xhtml

counselling or debriefing.[46, 51] Challenges around training, guidance and professional support are described in 'The healthcare system' section.

Time

A significant challenge faced by health professionals was the time required to provide healthcare for refugees and asylum seekers.[18, 37, 38, 40, 43, 47, 49-51, 55, 56, 59] More time was necessary due to the aforementioned challenges around building relationships,[18, 38, 40] communication,[38, 50, 55, 59] achieving cultural understanding,[47] and dealing with complex health conditions.[18, 38, 47, 50, 51] This additional time demand meant that appointments needed to be extended in duration [37, 47] or occur more frequently.[18, 49] Health professionals were concerned that time limitations could lead to 'rushed consultations' [59] and the potential to miss some conditions.[59] Some also commented that the extra time spent caring for refugees and asylum seekers drew them away from other patient groups.[40, 43]

The healthcare system

Health systems have been defined as "the combination of resources, organization, financing and management that culminate in the delivery of health services to the population".[61] They are the environment in which healthcare encounters take place. Healthcare professionals described health system related challenges and facilitators within 5 areas: training and guidance, professional support, connecting with other services, organisation, and resourcing and capacity.

Training and guidance

As already described in 'health and social conditions', health professionals thought that specific training and guidance would facilitate their clinical practice, improving their competence and confidence. Positive examples of training delivered were: orientation to services and resources available for refugees and asylum seekers;[40] culture specific information;[42, 54] engaging with women about FGM;[40] and trauma-sensitive care.[46] Despite this, a broad base of

For peer review only - http://bmjopen.bmj.com/site/about/guidelines.xhtml

For peer review only

participants identified lack of training, education or guidance as detrimental for practice.[17, 37, 38, 42, 43, 46, 49, 50, 55] Even when available, training may be inaccessible due to lack of awareness or time constraints.[43] Participants called for more training, guidance or information regarding integration with other health and social care services,[37, 42, 50] health profiles of specific groups,[46] cultural awareness/competence,[42, 46, 47, 49, 50] and the wider process of asylum.[37, 42]

Professional support

As reported in the earlier section 'health and social conditions', professional support was needed by health professionals working with refugees and asylum seekers. However professional support was identified as deficient in healthcare systems.[37, 43, 46, 55] Participants in one study described 'isolation' [43] that they felt within the healthcare system and another study described support networks as 'non-existent'.[37] Concerns were raised that health professionals exposed to distressing stories were not provided with sufficient psychological support.[46, 55]

Connecting with other services

Connecting with other health and social care services was another important facilitator for health professionals.[17, 18, 38, 40, 42, 47, 49-52, 54] Establishing referral pathways to different services in the healthcare system [40, 42, 47, 51, 52] and services within civil society [40, 42, 47] could direct refugees and asylum seekers to appropriate care. Some health visitors described accompanying refugees and asylum seekers to support groups to help with introductions.[40, 42] Good communication and cooperation between services was helpful [38] and fruitful collaborations with other services were recognised, such as delivering services together [50, 51] and working in multiagency teams to deliver holistic healthcare.[38, 51, 54]

Health professionals spoke of some difficulties referring refugees and asylum seekers to other health or social services.[17, 18, 39, 40, 50, 55] Some, services were not set up to meet their needs,[17, 40] others would not receive

For peer review only - http://bmjopen.bmj.com/site/about/guidelines.xhtml

referrals because they were operating at full capacity [18, 39] and sometimes services were simply not present.[18, 55] These challenges could be accentuated when health professionals found it difficult to navigate complex healthcare systems themselves.[43]

Organisation

Some articles highlighted flexibility in primary healthcare systems as beneficial in practice among refugees and asylum seekers.[40, 41, 43, 49, 50] This allowed for innovative approaches to optimise service delivery [40, 43] such as relocating services to more accessible places [18, 40, 42, 50] and adaptation of working patterns to better suit service users' needs.[43, 50]

Provision of specialised services for refugees and asylum seekers was supported across some studies,[37, 40, 43, 47] including initial health assessment services,[47] specialist teams [40, 47] and specialist centres.[37, 47] However, it was emphasised that these should integrate well into mainstream healthcare services.[37, 40]

Resourcing and capacity

Longer, more frequent appointments and utilisation of interpreters led to additional costs being incurred,[18, 19, 37, 43, 47, 49, 51] which some felt was not taken into account in health system financing models.[43, 47, 49] Some participants did not think that they could deliver adequate care as a result of funding shortages,[37, 55] with one study citing an example where interpreters were not able to be utilised because of lack of finance.[55]

Shortages in workforces were reported in some articles,[46, 47, 49] putting additional workload and stress onto health professionals.[46, 49] Reported consequences of this were closures of services to new patients [47, 49] and health professionals leaving their posts, further exacerbating the problem.[49] Interpreter shortages were also mentioned as a difficulty [46, 49, 56] along with inflexibility of their service operations.[37, 42, 55]

Asylum and resettlement

For peer review only - http://bmjopen.bmj.com/site/about/guidelines.xhtml

Further challenges were associated with the immigration status of, and legislative policy towards, refugees and asylum seekers.[18, 19, 37, 39, 40, 46, 47, 59] In some instances, health professionals were hindered in meeting health needs due to policy restrictions.[40] Difficulties understanding the frequently-changing policies towards, and entitlements for, refugees and asylum seekers were reported [39, 40] and uncertainty was expressed about healthcare pathways for this group upon arrival in the host country.[47] Some health professionals described conflicts in their professional duty to act as an advocate for their patients whilst requirements were placed on them to conduct assessments used to inform the asylum process.[19, 46] Another concern raised was a perception that service users were abusing the health and welfare systems,[18, 37, 40, 59] such as feigning symptoms of post-traumatic stress disorder to further their asylum claims [37] or illegal benefit claims.[18]

For peer review only

For peer review only - http://bmjopen.bmj.com/site/about/guidelines.xhtml

Discussion

Three analytical constructs containing 11 descriptive themes were interpreted in the thematic synthesis. Challenges and facilitators were located within the healthcare encounter (trusting relationships; communication; cultural understanding; health and social conditions; time), working within the healthcare system (training and guidance; professional support; connecting with other services; organisation; resourcing and capacity) and asylum and resettlement.

The growing research field of 'cultural competence' identifies components that can be incorporated into practice to enhance quality of care towards ethnic minority groups and reduce healthcare inequalities.[62, 63] Betancourt et al [62] defined cultural competence in healthcare as "the ability of systems to provide care to patients with diverse values, beliefs and behaviors, including tailoring delivery to meet patients' social, cultural, and linguistic needs".[62] This literature mirrors themes interpreted in the current review, including trusting relationships, communication and cultural understanding, as key components that may be optimised to improve healthcare and reduce inequalities.[62, 63]

Trusting relationships are essential for effective healthcare delivery.[64-66] Murray et al [67] identified continuity of relationship, time, interpersonal skills and 'getting to know patients' as enhancers of trust between health professionals and patients. The current review likewise recognised these elements, and it can be argued that even greater attention to trust-building is needed for refugees and asylum seekers, a vulnerable and ethnically diverse group who may be apprehensive about engagement with healthcare systems.[68, 69]

Communication between health professionals and patients is also regarded as essential.[70] Language discordance may compromise the quality of healthcare, lessening detection of ill health and referral to further healthcare.[71, 72] Health professionals in the current review consistently thought language barriers hindered their work with refugees and asylum seekers. The main strategy used to overcome language barriers was communication through

For peer review only - http://bmjopen.bmj.com/site/about/guidelines.xhtml

interpreters, as is recommended in the wider literature.[73-75] However, concerns were raised about the quality and availability of interpreters. Generally, it is recommended that professional interpreters are used, as they have been trained in professional standards, medical terminology and ethical issues.[75] Ad-hoc interpreters such as family or community members may be used pragmatically, although this may diminish the quality of interpretation and threaten patient confidentiality.[74, 75] Remote interpretation, such as telephone or video services have been developed to provide more efficient and timely services.[76, 77] The merits of such services have been debated [76, 77] and conflicting opinions were likewise given in this review. A systematic review [77] reported no significant difference in patient and provider satisfaction between remote and face-to-face interpreters, although subsequent primary studies have suggested a significant preference for in-person interpreters.[76]

Consistent with other research,[6-8, 11-13] health professionals encountered challenges dealing with complex physical, psychological and social problems of refugees and asylum seekers and did not always feel prepared to meet their needs. They also reported challenges in cross-cultural care such as different understandings of health, healthcare and healthcare systems, which introduced complications.

Participants in this review saw opportunities for improving care by working together with other health services and civil society. Identifying these organisations and possible areas of collaboration such as information sharing, referral pathways and joint service delivery may benefit health providers, health professionals and service users.

The organisation and delivery of primary healthcare services to refugees and asylum seekers is a growing research area, with service models being developed that integrate specialised components with existing structures.[78, 79] A model innovated in Australia established ‘Beacon practices’, which have expanded capacity for refugee care and may flexibly resource local services.[79] Such integrated services provide specialised resources without

For peer review only - http://bmjopen.bmj.com/site/about/guidelines.xhtml

isolating refugees and asylum seekers from general practice, which was a concern raised by some participants in this review.

Health professionals and health services operate within, and are influenced by, the wider healthcare policy environment. Decisions made at a political and health system levels invariably impact on front-line clinical practice in areas such as resourcing priorities, health professional roles and healthcare access.[80] Health professionals in this review recognised associated challenges, particularly when healthcare pathways were unclear and changeable. This emphasises the need for policy-makers to provide consistent, clear and up-to-date guidance on asylum and resettlement health policy for health professionals.

Public health implications

A central concern in public health is reduction of inequalities in health and healthcare.[81, 82] The WHO has established a commission on the social determinants of health that recommends actions addressing inequalities in health.[82] Healthcare inequalities exist when certain groups systematically receive lower quality care than the general population, resulting in poorer health outcomes.[80, 83] These inequalities have been widely observed in healthcare provision to ethnic minority groups across a broad range of health services [80] and has been highlighted as an issue for refugees and asylum seekers in the UK.[21] However, through knowledge translation, where evidence is moved into practice, challenges and facilitators identified in this review may be mapped onto components of healthcare interventions that may minimise such healthcare inequalities.[84]

Reduction in healthcare inequalities will likely require targeting healthcare resources towards disadvantaged groups.[79] For example, health professionals in this review highlighted the need for additional resources such as interpreter services, training and professional support to improve quality of care for refugees and asylum seekers.

For peer review only - http://bmjopen.bmj.com/site/about/guidelines.xhtml

Recommendations

Practice

Health professionals should be sufficiently resourced to meet the complex needs of refugees and asylum seekers. This should include provision of appropriate training on areas of cultural competence, asylum policies and process and health conditions. It is recommended that specific clinical guidelines are developed for provision of care to refugees and asylum seekers, drawing on the best available evidence. Further professional support should be given to those working with patients who present with complex psychological and social difficulties. Relevant, up to date information should be made available to inform health professionals about the needs of current waves of refugees and asylum seekers and other available services for referral and collaboration. Health providers should ensure adequate time is allocated for appointments with refugees and asylum seekers allowing space for trust building, communication and cultural understanding and develop infrastructure to ensure that trained interpreters are provided in a timely manner for appointments. Where resources permit, trained interpreters should be available with face-to-face and remote options (e.g. via phone), depending on patients' preferences.

Policy

Healthcare policy makers and commissioners should recognise the complex needs of refugees and asylum seekers, providing enhanced resources for quality and equitable service provision. Adequate human resourcing would allow health professionals to spend the necessary time to follow best practice. Integration of specialised components with existing general practice may facilitate care. Asylum and resettlement policy makers should seek to promote continuity of relationship with healthcare providers, limiting relocations.

Research

Primary qualitative research could explore other healthcare professionals' experiences of caring for refugees and asylum seekers. For example, no

For peer review only - http://bmjopen.bmj.com/site/about/guidelines.xhtml

studies of pharmacists' experiences were identified in this review. Further systematic reviews could be conducted to investigate experiences of health professionals working with refugees and asylum seekers in other areas of the healthcare system. A systematic review of challenges and facilitators for mental health professionals providing services to refugees and asylum seekers could inform service delivery for this group and searches in for this current review identified primary studies that could be included.

The outputs from this review may be used to inform service models for refugees and asylum seekers. Healthcare evaluations may be conducted to evaluate these models and identify areas that are able to improve quality of care

Strengths and limitations

An extensive and systematic search that was carried out across four databases complemented by reference and citation searches and it is therefore unlikely that published studies would have been overlooked. The inclusion of only English language studies may have led to under-representation of health professionals working in non-English speaking countries leading to a greater applicability to healthcare policy and practice in English speaking high-income countries. It is also possible that the database searches may not have identified studies where refugees and asylum seekers were referred to as 'migrants' or 'immigrants'; however, the additional hand-searches conducted would likely have identified any further key studies relevant for this review.

In study selection, titles and abstracts were screened by one reviewer, giving potential for selection bias or for relevant studies to be missed. By involving a second reviewer at the full-text selection stage, the study team sought to minimise bias, and supplementary searches of reference lists and citations reduced the potential for missing key studies. A second reviewer in data extraction could have reduced possibility of transcription errors, and in the quality appraisal stage could have minimised potential for biased assessment. Ideally, the analysis process would also have involved multiple reviewers in coding and formation of descriptive and analytical themes, bringing a wider perspective to interpretation.

For peer review only - http://bmjopen.bmj.com/site/about/guidelines.xhtml

Participants in this review were limited to the core clinical professions of nurses, primary care doctors and midwives. Other professionals, that may be part of primary healthcare teams, such as mental health workers, counsellors, physiotherapists and other community workers, were not included, raising a question about the transferability to more diverse primary healthcare teams. Studies including other professional groups report similar themes to the present review; however, those including mental health professionals may have a greater emphasis on secondary stress experienced when working with traumatised patients.[85, 86] A further consideration for transferability of these findings is the combining of data from the three clinical professions as they have different care practices, interaction with patients and support networks, giving the potential to introduce imprecision to the findings.

A strength of syntheses of qualitative research is that concepts are translated across studies, with common themes described that may be more transferable to other contexts and a greater ability to inform policy and practice.[26, 87] This contrasts with primary qualitative studies that are tied to their context and transference of findings is treated with caution.[26, 87] On the other hand, a perceived limitation of thematic syntheses is that they introduce a greater degree of abstraction from original experiences, sacrificing thickness of data and details found within the primary studies.[88] In this case, given that refugees are not a homogeneous group, it is perhaps acceptable to emphasise only the more generalised themes that transcend the contexts of individual studies.

Conclusions

Many people continue to be displaced due to conflict and persecution, seeking sanctuary in high-income countries. Health professionals experience a range of challenges and facilitators providing primary healthcare for this vulnerable group within the healthcare encounter, the environment of the healthcare system and in the broader context of asylum and resettlement policy and process. These challenges and facilitators provide valuable insight to inform practice and policy,

For peer review only - http://bmjopen.bmj.com/site/about/guidelines.xhtml

supporting quality healthcare and minimising healthcare inequalities for refugees and asylum seekers.

For peer review only

For peer review only - http://bmjopen.bmj.com/site/about/guidelines.xhtml

Footnotes

Contributors

LR designed the study, undertook the searches, data extraction, appraisal, synthesis and wrote the first draft of the manuscript. LLJ and SD supported study design, analysis, synthesis and interpretation, and provided critical revisions to the manuscript. LLJ had senior oversight and is the study guarantor. All authors revised the manuscript critically for important intellectual content and approved the final version for publication. All authors agree to be accountable for all aspects of the work.

Acknowledgements

The authors acknowledge the contribution of Madeline Flawn who was a second reviewer in study selection stage of the review.

Funding

This research received no specific grant from any funding agency in the public, commercial or not-for-profit sectors.

Competing interests

None

Data sharing

No additional data are available.

For peer review only

References

1 United Nations High Commissioner for Refugees. History of UNHCR. UNHCR 2016. http://www.unhcr.org/pages/49c3646cbc.html (accessed Jan 2016).

2 United Nations High Commissioner for Refugees. Convention and Protocol Relating to the Status of Refugees. UNHCR 2010. http://www.unhcr.org/protection/basic/3b66c2aa10/convention-protocol-relating-status-refugees.html (accessed Jan 2016).

3 United Nations High Commissioner for Refugees. Global Trends; Forced Displacement in 2015. UNHCR 2016. http://www.unhcr.org/statistics/unhcrstats/576408cd7/unhcr-global-trends-2015.html (accessed Aug 2016).

4 Aspinall P. Hidden Needs. Identifying Key Vulnerable Groups in Data Collections: Vulnerable Migrants, Gypsies and Travellers, Homeless People, and Sex Workers. Inclusion Health 2014. https://www.gov.uk/government/uploads/system/uploads/attachment_data/file/287805/vulnerable_groups_data_collections.pdf (accessed Jan 2016).

5 Daniel M, Devine C, Gillespie R, et al. Helping new refugees integrate into the UK: baseline data analysis from the Survey of New Refugees. UK Border Agency 2010. https://www.gov.uk/government/uploads/system/uploads/attachment_data/file/116069/horr36-report.pdf (accessed Jan 2016).

6 Clark RC, Mytton J. Estimating infectious disease in UK asylum seekers and refugees: a systematic review of prevalence studies. *J Public Health (Oxf)* 2007;29:420-8.

7 Allotey P. The health of refugees: public health perspectives from crisis to settlement. Oxford: Oxford University Press 2003.

8 Keygnaert I, Vettenburg N, Temmerman M. Hidden violence is silent rape: sexual and gender-based violence in refugees, asylum seekers and undocumented migrants in Belgium and the Netherlands. *Cult Health Sex* 2012;14:505-20.

9 Aptekman M, Rashid M, Wright V, et al. Unmet contraceptive needs among refugees. *Can Fam Physician* 2014;60:e613-9.

10 Amara AH, Aljunid SM. Noncommunicable diseases among urban refugees and asylum-seekers in developing countries: a neglected health care need. *Global Health* 2014;10:24.

For peer review only - http://bmjopen.bmj.com/site/about/guidelines.xhtml

11 Kalt A, Hossain M, Kiss L, et al. Asylum seekers, violence and health: a systematic review of research in high-income host countries. *Am J Public Health* 2013;103:30-42.

12 Miller KE, Worthington GJ, Muzurovic J, et al. Bosnian refugees and the stressors of exile: a narrative study. *Am J Orthopsychiatry* 2002;72:341-54.

13 Fazel M, Wheeler J, Danesh J. Prevalence of serious mental disorder in 7000 refugees resettled in western countries: a systematic review. *Lancet* 2005;365:1309-14.

14 Starfield B. Is primary care essential? *Lancet* 1994;344:1129-33.

15 World Health Organization. WHO Nursing and Midwifery Progress Report 2008-2012. Geneva: WHO 2013. http://www.who.int/hrh/nursing_midwifery/NursingMidwiferyProgressReport.pdf?ua=1 (accessed Jan 2016).

16 Burke M. The perceived experiences of primary healthcare professionals in Ireland: interprofessional teamwork in practice. Doctoral dissertation 2016.

17 Jensen NK, Norredam M, Priebe S, et al. How do general practitioners experience providing care to refugees with mental health problems? A qualitative study from Denmark. *BMC Fam Pract* 2013;14:17.

18 Burchill J, Pevalin D. Barriers to effective practice for health visitors working with asylum seekers and refugees. *Community Pract* 2012;85:20-3.

19 Kurth E, Jaeger FN, Zemp E, et al. Reproductive health care for asylum-seeking women - a challenge for health professionals. *BMC Public Health* 2010;10:659.

20 O'Donnell CA, Burns N, Mair FS, et al. Reducing the health care burden for marginalised migrants: the potential role for primary care in Europe. *Health Policy* 2016;120:495-508.

21 Jones D, Gill PS. Refugees and primary care: tackling the inequalities. *BMJ* 1998;317:1444-6.

22 Suphanchaimat R, Kantamaturapoj K, Putthasri W, et al. Challenges in the provision of healthcare services for migrants: a systematic review through providers' lens. *BMC Health Serv Res* 2015;15:390.

23 Holloway I. Qualitative Research in Nursing and Healthcare. Somerset: Wiley 2013.

24 Pope C, van Royen P, Baker R. Qualitative methods in research on healthcare quality. *Qual Saf Health Care* 2002;11:148-52.

For peer review only - http://bmjopen.bmj.com/site/about/guidelines.xhtml

25 Harden A, Garcia J, Oliver S, et al. Applying systematic review methods to studies of people's views: an example from public health research. *J Epidemiol Community Health* 2004;58:794-800.

26 Finfgeld-Connett D. Generalizability and transferability of meta-synthesis research findings. *J Adv Nurs* 2010;66:246-54.

27 Thomas J, Harden A. Methods for the thematic synthesis of qualitative research in systematic reviews. *BMC Med Res Methodol* 2008;8:45.

28 Barnett-Page E, Thomas J. Methods for the synthesis of qualitative research: a critical review. *BMC Med Res Methodol* 2009;9:1.

29 Tong A, Flemming K, McInnes E, et al. Enhancing transparency in reporting the synthesis of qualitative research: ENTREQ. *BMC Med Res Methodol* 2012;12:1.

30 Cooke A, Smith D, Booth A. Beyond PICO: the SPIDER tool for qualitative evidence synthesis. *Qual Health Res* 2012;22:1435-43.

31 The World Bank. World Bank list of economies 2015 [Data File]. The World Bank 2016. https://siteresources.worldbank.org/DATASTATISTICS/Resources/CLASS.XLS (accessed Jan 2016).

32 The Migration Observatory. Who Counts as a Migrant? Definitions and their Consequences. The Migration Observatory 2017. http://www.migrationobservatory.ox.ac.uk/resources/briefings/who-counts-as-a-migrant-definitions-and-their-consequences (accessed Mar 2017).

33 Hacker K, Anies M, Folb BL, et al. Barriers to health care for undocumented immigrants: a literature review. *Risk Manag Healthc Policy* 2015;8:175-83.

34 Critical Appraisal Skills Programme. Qualitative Research Checklist. Critical Appraisal Skills Programme 2014. http://media.wix.com/ugd/dded87_25658615020e427da194a325e7773d42.pdf (accessed Jan 2016).

35 Dixon-Woods M, Bonas S, Booth A, et al. How can systematic reviews incorporate qualitative research? A critical perspective. *Qual Res* 2006;6:27-44.

36 Passey ME, Longman JM, Robinson J, et al. Smoke-free homes: what are the barriers, motivators and enablers? A qualitative systematic review and thematic synthesis. *BMJ Open* 2016;6:e010260,2015-010260.

37 Begg H, Gill PS. Views of general practitioners towards refugees and asylum seekers: an interview study. *Diversity Health Soc Care* 2005;2:299-305.

For peer review only

For peer review only - http://bmjopen.bmj.com/site/about/guidelines.xhtml

38 Bennett S, Scammell J. Midwives caring for asylum-seeking women: research findings. *Pract Midwife* 2014;17:9-12.

39 Burchill J. Safeguarding vulnerable families: work with refugees and asylum seekers. *Community Pract* 2011;84:23-6.

40 Burchill J, Pevalin DJ. Demonstrating cultural competence within health-visiting practice: working with refugee and asylum-seeking families. *Divers Equal Health Care* 2014;11:151-9.

41 Carolan M, Cassar L. Pregnancy care for African refugee women in Australia: attendance at antenatal appointments. *Evid Based Midwifery* 2007;5:54-8.

42 Drennan VM, Joseph J. Health visiting and refugee families: issues in professional practice. *J Adv Nurs* 2005;49:155-63.

43 Farley R, Askew D, Kay M. Caring for refugees in general practice: perspectives from the coalface. *Aust J Prim Health* 2014;20:85-91.

44 Feldmann CT, Bensing JM, de Ruijter A. Worries are the mother of many diseases: General practitioners and refugees in the Netherlands on stress, being ill and prejudice. *Patient Educ Couns* 2007;65:369-80.

45 Furler J, Kokanovic R, Dowrick C, et al. Managing depression among ethnic communities: A qualitative study. *Ann Fam Med* 2010;8:231-6.

46 Griffiths R, Emrys E, Lamb CF, et al. Operation Safe Haven: The needs of nurses caring for refugees. *Int J Nurs Pract* 2003;9:183-90.

47 Johnson D.R., Ziersch A.M., Burgess T. I don't think general practice should be the front line: Experiences of general practitioners working with refugees in South Australia. *Aust New Zealand Health Policy* 2008;5:20.

48 Kokanovic R, May C, Dowrick C, et al. Negotiations of distress between East Timorese and Vietnamese refugees and their family doctors in Melbourne. *Sociol Health Illn* May 2010;32:511-27.

49 Lawrence J, Kearns R. Exploring the 'fit' between people and providers: refugee health needs and health care services in Mt Roskill, Auckland, New Zealand. *Health Soc Care Community* 2005;13:451-61.

50 Riggs E, Davis E, Gibbs L, et al. Accessing maternal and child health services in Melbourne, Australia: Reflections from refugee families and service providers. *BMC Health Serv Res* 2012;12:117-.

51 Samarasinghe K, Fridlund B, Arvidsson B. Primary health care nurses' promotion of involuntary migrant families' health. *Int Nurs Rev* 2010;57:224-31.

For peer review only - http://bmjopen.bmj.com/site/about/guidelines.xhtml

52 Suurmond J, Seeleman C, Rupp I, et al. Cultural competence among nurse practitioners working with asylum seekers. *Nurse Educ Today* 2010;30:821-6.

53 Suurmond J, Rupp I, Seeleman C, et al. The first contacts between healthcare providers and newly-arrived asylum seekers: A qualitative study about which issues need to be addressed. *Public Health* 2013;127:668-73.

54 Tellep TL, Chim M, Murphy S, et al. Great suffering, great compassion: A transcultural opportunity for school nurses caring for Cambodian refugee children. *J Transcult Nurs* 2001;12:261-74.

55 Tobin C.L., Murphy-Lawless J. Irish midwives' experiences of providing maternity care to non-Irish women seeking asylum. *Int J Womens Health* 2014;6:159-69.

56 Twohig PL, Burge F, MacLachlan R. Pod people. Response of family physicians and family practice nurses to Kosovar refugees in Greenwood, NS. *Can Fam Physician* 2000;46:2220-5.

57 Yelland J, Riggs E, Wahidi S, et al. How do Australian maternity and early childhood health services identify and respond to the settlement experience and social context of refugee background families? *BMC Pregnancy Childbirth* 2014;14:348.

58 Yelland J, Riggs E, Szwarc J, et al. Compromised communication: a qualitative study exploring Afghan families and health professionals' experience of interpreting support in Australian maternity care. *BMJ Qual Saf* 2016;25:e1.

59 Crowley P. The mental health needs of adult asylum seekers in Newcastle upon Tyne. *Public Ment Health* 2005;4:17-23.

60 Feldmann T. What do refugees and general practitioners say about medically unexplained physical symptoms? Medical errors undermine trust in the GP. *Huisarts Wet* 2007;50:381-4.

61 Roemer MI. National health systems of the world. Oxford: Oxford University Press 1993.

62 Betancourt J, Green A, Carillo J. Cultural competence in health care: Emerging frameworks and practical approaches. The Commonwealth Fund 2002. http://www.commonwealthfund.org/usr_doc/betancourt_culturalcompetence_576.pdf (accessed Aug 2016).

63 Saha S, Beach MC, Cooper LA. Patient centeredness, cultural competence and healthcare quality. *J Natl Med Assoc* 2008;100:1275-85.

For peer review only - http://bmjopen.bmj.com/site/about/guidelines.xhtml

64 Hall MA, Dugan E, Zheng B, et al. Trust in physicians and medical institutions: what is it, can it be measured, and does it matter? *Milbank Q* 2001;79:613-39.

65 Mainous AG, Baker R, Love MM, et al. Continuity of care and trust in one's physician: evidence from primary care in the United States and the United Kingdom. *Fam Med* 2001;33:22-7.

66 Mechanic D. The functions and limitations of trust in the provision of medical care. *J Health Polit Policy Law* 1998;23:661-86.

67 Murray B, McCrone S. An integrative review of promoting trust in the patient–primary care provider relationship. *J Adv Nurs* 2015;71:3-23.

68 Duncan GF. Refugee Healthcare: Towards Healing Relationships. *Canadian Social Science* 2015;11:158-68.

69 Peterson P, Sackey D, Correa-Velez I, et al. Building trust: delivering health care to newly arrived refugees. Mater UQ Centre for Primary Health Care Innovation 2011. http://www.materonline.org.au/services/refugee-services/pdfs/building-trust_-delivering-health-care-to-newl-(1) (accessed Aug 2016).

70 Ong LM, De Haes JC, Hoos AM, et al. Doctor-patient communication: a review of the literature. *Soc Sci Med* 1995;40:903-18.

71 Bischoff A, Bovier PA, Isah R, et al. Language barriers between nurses and asylum seekers: their impact on symptom reporting and referral. *Soc Sci Med* 2003;57:503-12.

72 Timmins CL. The impact of language barriers on the health care of Latinos in the United States: a review of the literature and guidelines for practice. *Midwifery Womens Health* 2002;47:80-96.

73 Flores G. The impact of medical interpreter services on the quality of health care: a systematic review. *Med Care Res Rev* 2005;62:255-99.

74 Karliner LS, Jacobs EA, Chen AH, et al. Do professional interpreters improve clinical care for patients with limited English proficiency? A systematic review of the literature. *Health Serv Res* 2007;42:727-54.

75 Mayo R, Parker VG, Sherrill WW, et al. Cutting Corners: Provider Perceptions of Interpretation Services and Factors Related to Use of an Ad Hoc Interpreter. *Hisp Health Care Int* 2016;14:73-80.

76 Locatis C, Williamson D, Gould-Kabler C, et al. Comparing in-person, video, and telephonic medical interpretation. *J Gen Intern Med* 2010;25:345-50.

For peer review only - http://bmjopen.bmj.com/site/about/guidelines.xhtml

77 Azarmina P, Wallace P. Remote interpretation in medical encounters: a systematic review. *J Telemed Telecare* 2005;11:140-5.

78 Feldman R. Primary health care for refugees and asylum seekers: A review of the literature and a framework for services. *Public Health* 2006;120:809-16.

79 Kay M, Jackson C, Nicholson C. Refugee health: a new model for delivering primary health care. *Aust J Prim Health* 2010;16:98-103.

80 Smedley BD, Stith AY, Nelson AR. Unequal treatment: Confronting racial and ethnic disparities in health care. Washington, DC: National Academies Press 2002.

81 Arcaya MC, Arcaya AL, Subramanian S. Inequalities in health: definitions, concepts, and theories. *Rev Panam Salud Publica* 2015;38:261-71.

82 World Health Organization. Closing the gap in a generation: health equity through action on the social determinants of health. Final Report of the Commission on Social Determinants of Health. Geneva: World Health Organization 2008. http://www.who.int/social_determinants/final_report/csdh_finalreport_2008.pdf (accessed Aug 2016).

83 Betancourt JR, Green AR, Carrillo JE, et al. Defining cultural competence: a practical framework for addressing racial/ethnic disparities in health and health care. *Public Health Rep* 2003;118:293-302.

84 Colquhoun H, Grimshaw J, Wensing M. Chapter 3.3b Mapping KT interventions to barriers and facilitators. In: Straus S, Tetroe J, Graham I, eds. Knowledge translation in health care: moving from evidence to practice. Oxford: Wiley-Blackwell 2013:137-49.

85 Puvimanasinghe T, Denson L, Augoustinos M, et al. Vicarious resilience and vicarious traumatisation: Experiences of working with refugees and asylum seekers in South Australia. *Transcult Psychiatry* 2015;52:743-65.

86 Priebe S, Sandhu S, Dias S, et al. Good practice in health care for migrants: views and experiences of care professionals in 16 European countries. *BMC Public Health* 2011;11:1.

87 Levack WM. The role of qualitative metasynthesis in evidence-based physical therapy. *Phys Ther Rev* 2012;17:390-7.

88 Sandelowski M, Docherty S, Emden C. Qualitative metasynthesis: issues and techniques. *Res Nurs Health* 1997;20:365-72.

For peer review only - http://bmjopen.bmj.com/site/about/guidelines.xhtml

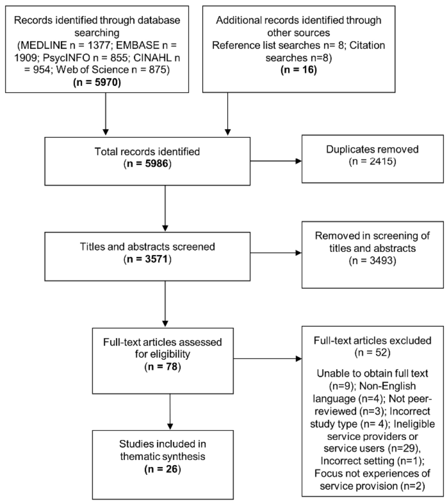

Figure 1 Flow diagram of systematic search and study selection

338x190mm (300 x 300 DPI)

review only

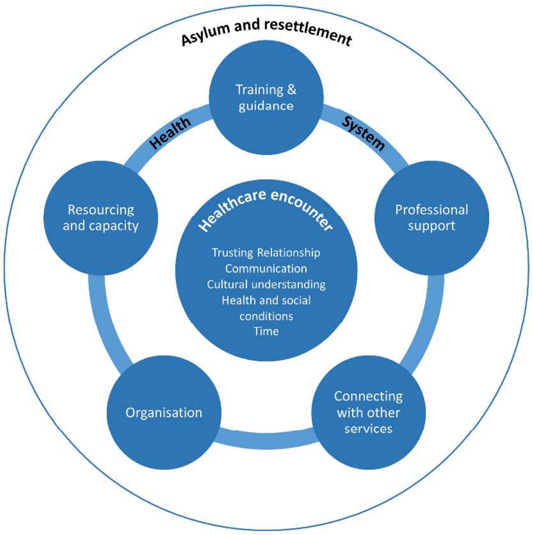

Figure 2: Model illustrating analytical constructs and descriptive themes

355x266mm (300 x 300 DPI)

For peer review only

Supplement 1: Database search strategy

MEDLINE	EMBASE	CINAHL	WEB OF SCIENCE	PSYCINFO
1. refugee/<br>2. asylum Seek$.mp.<br>3. refugee$.mp.	1. refugee/<br>2. asylum seeker/<br>3. asylum seek*.mp.<br>4. refugee*.mp.	1. MH "Refugees"<br>2. "refugee*"<br>3. "asylum seek*"	1. refugee*<br>2. asylum seek*	1. exp refugees/<br>2. asylum seek*.mp.<br>3. refugee*.mp.
4. 1 or 2 or 3	5. 1 or 2 or 3 or 4	4. 1 or 2 or 3	3. 1 or 2	4. 1 or 2 or 3
5. exp Primary Healthcare/<br>6. exp health services/<br>7. exp health personnel/<br>8. nurs$.mp.<br>9. pharmacist$.mp.<br>10. health care.mp.<br>11. midwi$.mp.<br>12. general practi$.mp.<br>13. service provi$.mp.<br>14. care prov$.mp.<br>15. healthcare.mp.	6. exp primary health care/<br>7. exp health service/<br>8. exp health care personnel/<br>9. healthcare.mp.<br>10. health care.mp.<br>11. nurs*.mp.<br>12. pharmacist*.mp.<br>13. midwi*.mp.<br>14. general practi*.mp.<br>15. service prov*.mp.<br>16. care prov*.mp.	5. MH "Facilities Manpower and Services+"<br>6. MH "Health Personnel+"<br>7. "healthcare"<br>8. "health care"<br>9. "service prov*"<br>10. "care prov*"<br>11. "nurs*"<br>12. "pharmacist*"<br>13. "midwi*"<br>14. "general practi*"	4. healthcare<br>5. health care<br>6. service prov*<br>7. care prov*<br>8. nurs*<br>9. pharmacist*<br>10. midwi*<br>11. general practi*	5. exp Health Care Services/<br>6. exp primary health care/<br>7. exp Health Personnel/<br>8. health care.mp.<br>9. healthcare.mp.<br>10. care prov*.mp.<br>11. service prov*.mp.<br>12. nurs*.mp.<br>13. pharmacist*.mp.<br>14. midwi*.mp.<br>15. general practi*.mp.
16. 5 or 6 or 7 or 8 or 9 or 10 or 11 or 12 or 13 or 14 or 15	17. 6 or 7 or 8 or 9 or 10 or 11 or 12 or 13 or 14 or 15 or 16	15. 5 or 6 or 7 or 8 or 9 or 10 or 11 or 12 or 13 or 14	12. 4 or 5 or 6 or 7 or 8 or 9 or 10 or 11	16. 5 or 6 or 7 or 8 or 9 or 10 or 11 or 12 or 13 or 14 or 15
17. qualitative.mp.<br>18. qualitative research/<br>19. mixed method$.mp.<br>20. experienc$.mp.<br>21. perception$.mp.<br>22. attitude$.mp.<br>23. Perspective$.mp.<br>24. challenge$.mp.<br>25. barrier$.mp.<br>26. facilitator$.mp.	18. qualitative research/<br>19. qualitative.mp.<br>20. mixed method*.mp.<br>21. experienc*.mp.<br>22. perception*.mp.<br>23. attitude*.mp.<br>24. perspective*.mp.<br>25. challeng*.mp.<br>26. facilitator*.mp.<br>27. barrier*.mp.	16. MH "Qualitative Studies+"<br>17. "qualitative*"<br>18. "mixed method*"<br>19. "experienc*"<br>20. "perception*"<br>21. "attitude*"<br>22. "perspective*"<br>23. "challeng*"<br>24. "facilitator*"<br>25. "barrier*"	13. qualitative<br>14. mixed method*<br>15. experienc*<br>16. perception*<br>17. attitude*<br>18. perspective*<br>19. challeng*<br>20. facilitator*<br>21. barrier*	17. exp Qualitative Research/<br>18. qualitative.mp.<br>19. mixed method*.mp.<br>20. experienc*.mp.<br>21. perception*.mp.<br>22. attitude*.mp.<br>23. perspective*.mp.<br>24. challeng*.mp.<br>25. facilitator*.mp.<br>26. barrier*.mp.
27. 17 or 18 or 19 or 20 or 21 or 22 or 23 or 24 or 25 or 26	28. 18 or 19 or 20 or 21 or 22 or 23 or 24 or 25 or 26 or 27	26. 16 or 17 or 18 or 19 or 20 or 21 or 22 or 23 or 24 or 25	22. 13 or 14 or 15 or 16 or 17 or 18 or 19 or 20 or 21	27. 17 or 18 or 19 or 20 or 21 or 22 or 23 or 24 or 25 or 26
28. 4 and 16 and 27	29. 5 and 17 and 28	27. 4 and 15 and 26	23. 3 and 12 and 22	28. 4 and 16 and 27
**1377**	**1909**	**954**	**875**	**855**

**Total from database searches**	**5,970**

Robertshaw et al 2017

Supplement 2: Selection criteria

Study type:

Include	Exclude
Primary qualitative research studies<br>Interviews/focus groups<br>Peer reviewed	Theses/Dissertations<br>Opinion articles<br>Case studies<br>Surveys (quantitative)<br>Organisation reports<br>Reviews

Primary health care professionals:

Include	Exclude
Nurses<br>General practitioners<br>Midwives<br>Health visitors (nurse/midwives)<br>Pharmacists	Obstetricians<br>Psychologists<br>Psychotherapists<br>Physiotherapists<br>Counsellors<br>Social workers<br>Managers<br>Interpreters<br>Volunteers<br>Unqualified health professional (e.g student nurse)<br>Unspecified staff within service providers

Health care service users:

Include	Exclude
Refugees<br>Asylum seekers<br>Forced/Involuntary migrants<br>Refugee claimant	Migrants<br>Immigrants<br>Undocumented migrant<br>Illegal immigrant

Setting of practice of health professionals:

Include	Exclude
Community<br>Community health centres<br>General practices<br>Community clinics<br>Refugee/asylum centres	Asylum seeker detention centre<br>Hospitals- acute care<br>Specialist centres: referral from primary care

For peer review only - http://bmjopen.bmj.com/site/about/guidelines.xhtml

For peer review only

High-income countries (World Bank classification 2015[1]):

Include:

Andorra
Antigua and Barbuda
Argentina
Aruba
Australia
Austria
Bahamas, The
Bahrain
Barbados
Belgium
Bermuda
Brunei Darussalam
Canada
Cayman Islands
Channel Islands
Chile
Croatia
Curaçao
Cyprus
Czech Republic
Denmark
Equatorial Guinea
Estonia
Faeroe Islands
Finland
France
French Polynesia
Germany
Greece
Greenland
Guam
Hong Kong SAR, China
Hungary
Iceland
Ireland
Isle of Man
Israel
Italy
Japan
Korea, Rep.
Kuwait
Latvia
Liechtenstein
Lithuania
Luxembourg
Macao SAR, China
Malta
Monaco
Netherlands
New Caledonia
New Zealand
Northern Mariana Islands
Norway
Oman
Poland
Portugal
Puerto Rico
Qatar
Russian Federation
San Marino
Saudi Arabia
Seychelles
Singapore
Sint Maarten (Dutch part)
Slovak Republic
Slovenia
Spain
St. Kitts and Nevis
St. Martin (French part)
Sweden
Switzerland
Taiwan, China
Trinidad and Tobago
Turks and Caicos Islands
United Arab Emirates
United Kingdom
United States
Uruguay
Venezuela, RB
Virgin Islands (U.S.)

Focus of study:

Include	Exclude
Experiences providing primary healthcare for refugees and asylum seekers	Experiences treating a specific condition common in refugees and asylum seekers, but no focus on healthcare interactions.<br>Experiences of a particular service or organisation for refugees and asylum seekers<br>HCP's perspectives on refugees and asylum seekers' experiences

[1] The World Bank, World Bank list of economies (July 2015) [Data file]. Retrieved from http://data.worldbank.org/about/country-and-lending-groups

Robertshaw et al 2017

For peer review only - http://bmjopen.bmj.com/site/about/guidelines.xhtml

Supplement 3: Data extraction of studies included in the thematic synthesis

Study Reference	Country of study	Study type	Total Participants in Study	Health professional practice setting	Service users	Data collection method	Analysis method	Study aims/purpose (Author's words)	Reported results (Themes/main headings)	Recommendations/applications (Author's words)
Begg H, Gill PS. Views of general practitioners towards refugees and asylum seekers: an interview study. Diversity Health Soc Care 2005 12;2(4):299-305 7p.	United Kingdom	Qualitative	17 general practitioners	General practice	Refugees and asylum seekers	Semi-structured interviews	Thematic framework (Ritchie and Spencer, 1993)	To identify some of the concerns of 17 general Practitioners working in an urban environment.	1.Political logistics and the asylum process<br>2 Community issues<br>3 Impact upon primary care<br>4 Resources and resource management<br>5 Training needs within primary care	> Guidelines and protocols for practice ...GPs would welcome those that might help them to deliver healthcare to refugees and asylum seekers.<br>>Primary care trusts need to liaise with local authorities and the Home Office to identify areas to which large numbers of asylum seekers are dispersed.
Bennett S, Scammell J. Midwives caring for asylum-seeking women: research findings. Pract Midwife 2014 Jan;17(1):9-12.	United Kingdom	Qualitative	10 midwives	Setting unclear, but includes community, rotational, specialist and delivery suite midwives.	Asylum seeking women	Semi-structured interviews	Thematic analysis (Bryman 2008)	The aim of this research was to gain an in depth analysis of the experiences of midwives and their understanding of the specific needs of asylum-seeking women. The findings would be used to inform education, practice and policy to enable more effective delivery of woman-centred care for this group locally.	1. Time<br>2. Communication	>Midwives deserve support in practice and enhanced education, and policy around asylum-seeking women would facilitate more effective, evidence-based care.<br>>It is essential that midwives (and other members of the multi-disciplinary team) have access to and training in the use of interpreting services.<br>>The additional time required to provide care to women seeking asylum should be factored into midwives' workloads.<br>>Education programmes to prepare/enhance knowledge and skills in caring for asylum seekers<br>>Web based resource with information about asylum seekers.
Burchill J. Safeguarding vulnerable families: work with refugees and asylum seekers. Community Practitioner 2011 Feb;84(2):23-26.	United Kingdom	Qualitative	14 health visitors	London borough	Refugees and asylum seekers	In-depth interviews	Thematic framework (Ritchie & Spencer, 1994)	Not clearly stated	1. Complexity of safeguarding-related needs<br>2. Sole support agent<br>3. Cultural challenges<br>4. Cycle of abuse<br>5. Disappearing from the system	> Increase awareness for effective commissioning of appropriate services for this group.<br>> Joint working may prevent the difficulties that health visitors face when working with vulnerable populations such as asylum seekers and refugees.<br>> Health visitors working with vulnerable populations need to explore opportunities to highlight concerns with their managers and commissioners.
Burchill J, Pevalin D. Barriers to effective practice for health visitors working with asylum seekers and refugees. Community Practitioner 2012 Jul;85(7):20-23.	United Kingdom	Qualitative	14 health visitors	London borough	Refugees and asylum seekers	In-depth interviews	Thematic framework (Ritchie & Spencer, 1994)	To determine the barriers to effective practice that health visitors when working with refugees and asylum seekers.	1.Ineffective engagement<br>2.Stretched resources	> Health professionals share innovative ways of working to in order to reduce the barriers experienced by refugees and asylum seekers.<br>> Increase awareness among primary health care staff of entitlement to health services for this particular client group.<br>> Commissioners should have an awareness of barriers to effective practice when deciding how to invest in services for vulnerable populations.
Burchill J, Pevalin DJ. Demonstrating cultural competence within health-visiting practice: working with refugee and asylum-seeking families. Diversity Equality Health Care 2014 06;11(2):151-159 9p.	United Kingdom	Qualitative	14 health visitors	London borough	Refugees and asylum seekers	In-depth interviews	Thematic framework (Ritchie & Spencer, 1994)	Explored the experiences of health visitors working with refugee and asylum-seeking families in central London, and assessed the dimensions of their cultural competency using Quickfall's model (Quickfall, 2004, 2010)	1.Institutional regard<br>2.Cultural awareness<br>3.Cultural sensitivity<br>4.Cultural knowledge<br>5.Cultural competence	> Health visitors need to be able to demonstrate cultural competence in their practice with refugee and asylum-seeking families.

For peer review only - http://bmjopen.bmj.com/site/about/guidelines.xhtml

For peer review only

Study Reference	Country of study	Study type	Total Participants in Study	Health professional practice setting	Service users	Data collection method	Analysis method	Study aims/purpose (Author's words)	Reported results (Themes/main headings)	Recommendations/applications (Author's words)
Carolan M, Cassar L. Pregnancy care for African refugee women in Australia: attendance at antenatal appointments. Evid Based Midwifery 2007 2007;5(2):54-58 5p.	Australia	Qualitative	2 midwives<br>10 African women*<br>1 community worker*<br>1 interpreter*<br>1 family and reproductive rights education program worker*	African women's clinic within a community health centre	African refugee women	Observational methods and Semi-structured interviews	Not explicitly stated, but think perhaps Thematic Analysis	To explore factors that facilitate or impede the uptake of antenatal care among African refugee women.	1.Staff attitudes<br>2.Availability of interpreters<br>3.Knowledge about the clinic at community level<br>4.Convenient location of the clinic	>Community midwifery clinics might offer a solution in terms of providing an acceptable and sensitive service to refugee African women. This familiar service would allow the women to meet the same carers on each visit, which would facilitate the development of trust.<br>>Opportunity for the clinic staff to tailor services to identified needs, such as the provision of interpreters in specific languages, liaison with medical and midwifery specialists with a knowledge of African disease and access to social and community workers.
Crowley P. The mental health needs of adult asylum seekers in Newcastle upon Tyne. Journal of Public Mental Health 2005;4(1):17-23.	United Kingdom	Mixed methods	10 general practitioners<br>67 asylum seekers (quantitative)*<br>? asylum seekers (qualitative)*<br>? managers*<br>? mental health service providers*<br>? housing support*<br>? agency staff*<br>? voluntary sector service providers*<br>? interpreters*	General practice and community	Asylum seekers	Interviews<br>Telephone interviews<br>Focus groups	Unspecified	To assess the mental health care needs of adult asylum seekers in Newcastle upon Tyne.	A. Quantitative<br>1. Demographic information<br>2. Mental illness prevalence in primary care<br>3. Mental illness prevalence in the general population<br>4. Mental health service use<br>B. Qualitative<br>1. Asylum seekers<br>2. Housing support workers and interpreters<br>3. Voluntary sector service providers<br>4. GP practices<br>5. Mental health service providers and managers<br>6. Regional and national agencies	> Increase opportunities for self-sufficiency; developing social support; developing peer groups; strengthening links with the host community; tackling racial harassment; improving economic well-being, and facilitating communication with families.<br>> Primary care practices need more education, training, support and resource to meet the needs of asylum seekers effectively, and to address the issue of hostility from other patients.<br>> There is a need both to improve mental health services and to strengthen social and other forms of support both within the communities to which asylum seekers belong and within host communities.<br>>In Newcastle, weaknesses in policy and practice in the mental health trust require attention in the light of the overall need to develop mental health services that best meet the need of the whole population.<br>> A greater level of sensitivity to the mental health needs of asylum seekers is required across the public sector, together with recognition of the major impact that experience in the host country has on their mental health and well-being.
Drennan VM, Joseph J. Health visiting and refugee families: issues in professional practice. J Adv Nurs 2005 01/15;49(2):155-163 9p.	United Kingdom	Qualitative	13 health visitors	2 inner London borough's	Refugees and asylum seekers	Semi-structured interviews	Framework method (Ritchie and Spencer 1994)	Describe health visitors' experiences working in Inner London and identifying and addressing the health needs of refugee woman in the first 3 months after the birth of a baby.<br>Investigate health visitors' perceptions of effective and ineffective strategies in identifying and addressing health needs of these women.<br>Investigate whether health visitors used a framework corresponding to Maslow's theory of a hierarchy of needs to prioritize their public health work.	1. Complexity of the relationship between health visitors and clients who are refugees.<br>2. Identification and prioritization of the health needs of the asylum seeking and refugee families.<br>3. Health visitors' perceptions of successful outcomes of their work.<br>4. Impact on health visitors of working with asylum seekers and refugees.	> There is a service and professional responsibility to ensure that health visiting and public health nursing practice is developed from the best evidence available and that collective knowledge and expertise are shared, rather than left for each practitioner to discover through trial and error.<br>> Both professional education providers and service providers need to pay attention to the specific health and social needs of asylum seeking women, who will unfortunately continue to arrive in the UK and other parts of the world.
Farley R, Askew D, Kay M. Caring for refugees in	Australia	Qualitative	20 general practitioners	General practice	Newly arrived refugees	Focus groups and	Thematic analysis	Explored the experiences of primary health care	1. Communication<br>2. Knowledge	> Increase range of resources available in languages other than English. Support English education for

For peer review only - http://bmjopen.bmj.com/site/about/guidelines.xhtml

Study Reference	Country of study	Study type	Total Participants in Study	Health professional practice setting	Service users	Data collection method	Analysis method	Study aims/purpose (Author's words)	Reported results (Themes/main headings)	Recommendations/applications (Author's words)
general practice: perspectives from the coalface. Australian Journal of Primary Health 2014;20(1):85-91.			5 practice Nurses<br>11 administrators*			semi-structured interviews		providers working with newly arrived refugees in Brisbane...focusing on the barriers and enablers they continue to experience in providing care to refugees.	3. Practice and health care systems	refugees. Support providers in understanding the linguistic backgrounds of their patients. Consider the importance of literacy in English education for refugees Improve availability and quality of visual resources. Raise awareness of refugees' limited literacy among providers. Increase interpreter service availability across all health care sectors (including allied health). Improve medical interpreter training. Provide information for providers regarding cultural differences in communication and the impact this can have on a consultation.<br>> Provide focussed education and training around important refugee health issues.<br>> Provide mental health training for providers, particularly in relation to caring for victims of past torture and trauma Improve supports available to providers working in this area, through access to trained psychologist and bicultural workers. Enhance psychologists' access to interpreters.<br>> Provide initial refugee health care in a specialised refugee health setting and ensure effective communication and support at the time of referral and beyond. Provide a forum for the exchange and transfer of experiences, information and resources between providers working in this area. Provide focussed education and training for providers, regarding the health care system as it pertains to refugee health care. Identify and adequately resource relevant support organisations. Consider methods to adequately remunerate providers (e.g. Medicare payments when interpreters are used). Provide case workers with appropriate training to assist in coordinating care.<br>> Provide education for refugees around health care within the Australian health care system.
Feldmann CT, Bensing JM, de Ruijter A. Worries are the mother of many diseases: General practitioners and refugees in the Netherlands on stress, being ill and prejudice. Patient Educ Couns Mar 2007;65(3):369-380	Netherlands	Qualitative	66 refugees*<br>24 general practitioners	General practice	Refugees (Afghan & Somali)	In-depth interviews	Not specified	To confront the views of refugee patients and general practitioners in the Netherlands, focusing on medically unexplained physical symptoms (MUPS).	1. Perspectives of refugees<br>-General narrative versus personal narratives<br>-Refugees' concepts of health and illness<br>-Causes of illness—mental worries<br>-Personal responsibility—strategies to stay healthy<br>-Expectations from doctors<br>-Refugees' problems with doctors<br>2. The general practitioners' perspective<br>-General practitioners on refugee problems<br>-How doctors deal with refugee problems<br>-Human interest strategy	>For a fruitful cooperation to develop, based on trust, GPs need to invest in the relationship with individual refugees, and avoid statements or actions based on stereotypes and prejudice. There is a heartening parallel between refugees' expectations and GPs' best practices.<br>> Direct observation, visual registration and later (qualitative) analysis of consultations between general practitioners and refugee patients, combined with eliciting refugees' expectations and level of trust before the consultation, and both the GPs' and the refugees' assessments afterwards, can help to raise awareness of possibilities for improvement in specific practices.<br>>Early investment in the relationship with new refugee patients may be crucial to establishing a basis of trust and dealing with unexplained physical symptoms effectively.<br>>Asking (refugee) patients about their situation and the way they are dealing with it, separate from the complaint that is being presented, helps to create an atmosphere of joint responsibility.

For peer review only - http://bmjopen.bmj.com/site/about/guidelines.xhtml

Study Reference	Country of study	Study type	Total Participants in Study	Health professional practice setting	Service users	Data collection method	Analysis method	Study aims/purpose (Author's words)	Reported results (Themes/main headings)	Recommendations/applications (Author's words)
									-Technical strategy<br>-Elements that occur in both 'human interest' and 'technical' strategies	>A physical complaint always deserves a thorough physical examination.<br>>The tendency to stereotype refugee patients may be a serious pitfall for practitioners.<br>>Critical reflection by practitioners is needed on strategies they employ for dealing with unexplained physical symptoms.<br>>Professional errors by medical practitioners have a long life circulating as part of the 'general narrative' in refugee communities, undermining trust. A more open climate when dealing with professional mistakes, especially towards the patients involved and their relatives, may help to address this phenomenon.
Furler J, Kokanovic R, Dowrick C, Newton D, Gunn J, May C. Managing depression among ethnic communities: A qualitative study. Annals of Family Medicine May-Jun 2010;8(3):231-236.	Australia	Qualitative	8 family physicians	Community health centre	Refugees with depression	Semi-structured interviews	Thematic analysis (Mays & Pope 1995)	Explores the complexities of this work [clinical care for depression] through a study of how family physicians experience working with different ethnic minority communities in recognizing, understanding, and caring for patients with depression.	1. Understanding and negotiating the problem of depression<br>2. Managing the depression<br>3.Working with the interpreter	>Highlight the need for more detailed observational research of clinical care for depression across a range of primary care settings and contexts.
Griffiths R, Emrys E, Lamb CF, Eagar S, Smith M. Operation Safe Haven: The needs of nurses caring for refugees. Int J Nurs Pract Jun 2003;9(3):183-190.	Australia	Qualitative	13 nurses<br>1 medical records clerk*<br>2 nursing managers	Refugee reception centre	Refugees	2 focus groups (13 nurses + 1 clerk), Semi-structured interviews (2 nurse managers)	Thematic analysis	To identify the skills, knowledge and support nurses require to provide holistic and competent care to refugee children and their families and the nature of support that is required to assist their transition back to mainstream health services.	1. Clinical skills and knowledge required by Safe Haven nursing staff.<br>2. Cultural competency skills<br>3. Trauma-sensitive care<br>4. Stressors impacting on Safe Haven nurses<br>5. Sources of support for Safe Haven nurses<br>6. Rewards<br>7. Return to work	>Counselling (for Nurses) should be provided by qualified, on-site counsellors with good understanding of trauma-related issues.<br>>Nursing workforce planners need to be able to employ appropriate numbers of permanent staff for extended disaster operations, avoiding the need for excessive work hours or the unsustainable practice of 'partial secondment', where nurses are expected to carry out disaster-type work and maintain their existing work responsibilities.<br>>Nursing workforce planners should undertake strategic recruitment during extended disaster operations, identifying appropriately skilled workers to form a stable workforce offering continuity of care.<br>>Disaster planners at the Area Health Service level should identify appropriate external agencies and designated health providers to assist with clinical management during extended operations, where nurses work with increased autonomy.
Jensen NK, Norredam M, Priebe S, Krasnik A. How do general practitioners experience providing care to refugees with mental health problems? A qualitative study from Denmark. BMC Family Practice 2013;14:17.	Denmark	Qualitative	9 general practitioners	Medical clinics with high proportion of immigrants	Refugees	Semi-structured interviews	Content analysis (Graneheim and Lundman 2004)	To qualitatively explore issues identified by general practitioners as important in their experiences of providing care for refugees with mental health problems.	1. Communication<br>2. Quality of care<br>3. Referral pathways<br>4. Understandings of disease and expectations of treatment.	>The findings from this study suggest that there is an increased need for general practitioners to be aware of potential traumas experienced by refugee patients, but also leave room for taking individual differences into account in the consultation. This could be attained by the development of conversational models for general practitioners including points to be aware of in the treatment of refugee patients. This may serve as a support in the health care management of refugee patients, but at the same time does not disregard the resources of individual refugee patients.

For peer review only - http://bmjopen.bmj.com/site/about/guidelines.xhtml

Study Reference	Country of study	Study type	Total Participants in Study	Health professional practice setting	Service users	Data collection method	Analysis method	Study aims/purpose (Author's words)	Reported results (Themes/main headings)	Recommendations/applications (Author's words)
Johnson D.R., Ziersch A.M., Burgess T. I don't think general practice should be the front line: Experiences of general practitioners working with refugees in South Australia. Australia and New Zealand Health Policy 2008;5(pagination):Arte Number: 20. ate of Pubaton: 08 Aug 2008.	Australia	Qualitative	12 general practitioners<br>3 medical directors of divisions of general practice*	General practice	Refugees	Semi-structured interviews	Template analysis	To document the existence and nature of challenges for GPs who do this work in SA.<br>To explore the ways in which these challenges could be reduced.<br>To discuss the policy implications of this in relation to optimising the initial health care for refugees.	1. Challenges for GPs<br>a)Refugee health issues<br>-GP knowledge of previous health assessments<br>- GP awareness of and experience managing health conditions unique to refugees<br>- The multiple and complex nature of refugee health conditions<br>b) GP-refugee interaction<br>- Issues related to culture<br>- Issues related to language<br>- Refugee knowledge of the Australian healthcare system<br>c) Structure of general practice<br>- GP workforce shortages<br>- Referral systems<br>- Remuneration<br>- Infrastructure supports to perform initial assessments<br>2. Challenges for Divisions assisting GPs<br>3. Ways GPs could be better supported<br>a) Providing GPs with more resources<br>b) Providing initial refugee health care via a specialist service	>Utilise a specialist service for refugees in refugees' resettlement period, which could provide initial health assessments and expertise in working with this population.<br>> If initial health assessments are provided by a specialist service, it is important that a clear, transparent and effective referral system to a nominated general practice is part of this process when initial health care needs have been met.
Kokanovic R, May C, Dowrick C, Furler J, Newton D, Gunn J. Negotiations of distress between East Timorese and Vietnamese refugees and their family doctors in Melbourne. Sociol Health Illn May 2010;32(4):511-527.	Australia	Qualitative	5 general practitioners<br>24 refugees from Vietnam and East Timor*	Community health centre	Refugees	In depth interviews	Thematic analysis	We explore a set of cultural boundaries across which depression is contested: between recent migrants to Australia from East Timor and Vietnam, and their white 'Anglo' family doctors. We are concerned with the ways that the experiences of migration and its aftermath are manifest in the lives of people from these ethnic groups; how their consequent distress is negotiated and contested in their interactions with family doctors; and how the	1. The journey and the arrival are important<br>2. Home and family: here and there<br>3.The naming of parts: manifestations of and bringing distress into the medical encounter<br>4. Illness Labels: naming distress	> Reinvestigate the way of conducting research on depression in a cross-cultural context.

For peer review only

Study Reference	Country of study	Study type	Total Participants in Study	Health professional practice setting	Service users	Data collection method	Analysis method	Study aims/purpose (Author's words)	Reported results (Themes/main headings)	Recommendations/applications (Author's words)
								resulting collisions affect the meaningfulness of the concept of depression.		
Kurth E, Jaeger FN, Zemp E, Tschudin S, Bischoff A. Reproductive health care for asylum-seeking women - a challenge for health professionals. BMC Public Health 2010;10:659.	Switzerland	Mixed-Methods. Quantitative element, based on patient files, explored frequencies of diagnoses and medical interventions. Qualitative element analysed data from asylum seekers patient notes and interviews with health professionals.	80 asylum seekers*<br>3 physicians<br>3 nurse/ midwife<br>1 psychologist*<br>3 interpreter*	Women's clinic	Female asylum seekers	Semi-structured Interviews with the 10 health professionals. Textual data was extracted from the 80 asylum seeker's patient files. The quantitative element extracted data from hospital electronic database and patient files.	Grounded theory methodology	The aim of the present study was to investigate the reproductive health care provided for women asylum seekers attending the Women's Clinic of the University Hospital in the city of Basel, Switzerland. To identify the health needs of asylum seekers attending the Women's Clinic and to investigate the health care they received in a Health maintenance organisation (HMO) specifically established for asylum seekers. Explored the perceptions of the health care professionals Involved about providing health care for this group in this setting.	1. Language and cultural barriers<br>2. Conflicting roles of physicians<br>Unclear how these themes were chosen from all of the data	> Specific training and support for health care providers.<br>> Training and support are needed not only because of the emotional challenges resulting from the situation, but because the patients do not only need medical care, but very often suffer from severe psychosocial problems arising from the stressful situation they are in.<br>>Attention should also be paid to stressors that could potentially affect health professionals and their work: the need for support and training of health care providers caring for vulnerable populations should be investigated further.<br>> The effect on health care providers of working in a restrictive HMO setting, where they do not only have to carry out their traditional clinical tasks but must also cope with increasing managerial responsibilities and financial restrictions, may also warrant further study.<br>> Language barriers can be overcome with the use of well-trained professional interpreters - both for the patients' sake and to avoid frustration in health care providers.
Lawrence J, Kearns R. Exploring the 'fit' between people and providers: refugee health needs and health care services in Mt Roskill, Auckland, New Zealand. Health & Social Care in the Community 2005 Sep;13(5):451-461.	New Zealand	Qualitative	5 community representatives*<br>9 refugee group representatives*<br>5 medical practitioners<br>1 manager*<br>1 administrator*	Community health centre	Refugees	Semi-structured Interviews	Thematic analysis	This paper reports on research that sought to reveal the barriers faced by refugees in accessing health services, and the challenges faced by providers in endeavouring to meet needs in an effective and culturally appropriate manner.	1. Population change within the Roskill area<br>2. Refugee perspectives on barriers to accessing health services<br>- Resettlement issues<br>-Differing cultural understanding of illnesses and health care systems<br>-Distrust of others<br>- Difficulties in communication<br>-Cost<br>-Physical access difficulties<br>3. Experiences of health practitioners in delivering health services to refugees	>The changing social landscape of larger Western cities...demands a greater attentiveness to the health needs of a population and the health services in place at a neighbourhood level<br>>In Mt Roskill...further adjustments in terms of funding, staffing, training and the style of patient/professional contact seem a necessary prerequisite for advancing health and social care in the community.<br>>There is clear need for funded health educators to provide a comprehensive orientation on such matters at the time of their registration at a service like HoP.<br>>Many of the delays and frustrations experienced by both the users and providers of services would be addressed by the funding of appropriate translation services.<br>>We advocate an enhanced commitment to developing cultural awareness through incorporating social-scientific perspectives to complement biomedical knowledge in medical education.<br>>To achieve this responsiveness [to community demographics], maintaining an elected board comprising both community and clinic representatives, as well as developing relationships with sympathetic researchers, can assist in bridging what otherwise might be a gulf between clinic and community.
Riggs E, Davis E, Gibbs L, Block K, Szwarc J,	Australia	Qualitative	87 refugee background	Maternal and child health (MCH)	Refugee background	Focus groups	Thematic analysis	This study aims to explore the utilisation	1. Facilitating access to MCH services.	> Provision of refugee focussed training for service providers and a strategically coordinated approach is

For peer review only - http://bmjopen.bmj.com/site/about/guidelines.xhtml

Study Reference	Country of study	Study type	Total Participants in Study	Health professional practice setting	Service users	Data collection method	Analysis method	Study aims/purpose (Author's words)	Reported results (Themes/main headings)	Recommendations/applications (Author's words)
Casey S, et al. Accessing maternal and child health services in Melbourne, Australia: Reflections from refugee families and service providers. BMC Health Serv Res 2012 01;12(1):117-117 1p.			mothers*<br>12 nurses<br>1 community worker*<br>1 community liaison*<br>5 bilingual workers*<br>3 community representatives*<br>2 managers of bilingual workers*	service	mothers	(refugees, nurses, bilingual workers, community worker, community liaison) and individual interviews (community representatives, managers of bilingual workers)		and experience of MCH services in Melbourne, Victoria for parents of refugee background from the perspective of users and providers.	2. Promoting continued engagement with the MCH service.<br>3. Language challenges.<br>4. What is working well and what could be done better?	likely to facilitate access, build rapport and ongoing engagement and retention to the service for families of refugee background.<br>> Innovative culturally competent strategies to organise individual MCH service appointments should be trialled and evaluated to develop a MCH system that promotes refugee maternal and child health.<br>> Trial a model where MCH nurses attend venues where refugees already gather to promote MCH services, provide information and build trust.<br>> The role played by bicultural workers should be recognised and utilised in a way that benefits clients and service providers.<br>> MCH services could proactively work in partnership with bilingual community workers to call clients directly to make appointments. Where these workers are not available, interpreters could also be utilised for this purpose.
Samarasinghe K, Fridlund B, Arvidsson B. Primary health care nurses' promotion of involuntary migrant families' health. Int Nurs Rev 2010;57(2):224-231.	Sweden	Qualitative	34 primary health care nurses	Various primary health care settings: maternity, child, school and community health care, and nurse-led clinics covering asthma, allergy, diabetes and hypertension	Involuntary migrants	Interviews	Contextual analysis (Phenomenography)	The aim of this study was to describe the promotion of health in involuntary migrant families in cultural transition as conceptualized by Swedish PHCNs.	1. Category I. An ethnocentric approach focusing on the physical health of the individual<br>2. Category II. An empathic approach focusing on the mental health of the individual in a family context<br>3. Category III. A holistic approach empowering the family to function well in everyday life	> In orientating families to cultural values of host country, teaching new cultural behaviours must be carried out in a respectful way so that the families do not feel subjected to forced assimilation.<br>> having family conversations with the entire family about the impact of acculturation on interpersonal relationships may be helpful in strengthening family relations.<br>> To enhance family health and family cohesion, nurses need to facilitate involuntary migrant families' cultural transition by empowering the family to be in control of acculturation.<br>>For nurses to enhance family health during cultural transition, adequate education encompassing the development of intercultural communication skills and cultural self-awareness must be available at both undergraduate as well as post-graduate level on a national basis.<br>>In clinical practice, the implementation of family-focused nursing incorporating supportive conversations about acculturation and adaptation will be useful.
Suurmond J, Rupp I, Seeleman C, Goosen S, Stronks K. The first contacts between healthcare providers and newly-arrived asylum seekers: A qualitative study about which issues need to be addressed. Public Health Jul 2013;127(7):668-673.	Netherlands	Qualitative	36 nurse practitioners<br>10 public health physicians	Asylum seeker centres	Newly arrived asylum seekers	Group interviews	Framework	To describe the tacit knowledge of Dutch healthcare providers about the care to newly arrived asylum seekers and to give insight into the specific issues that healthcare providers need to address in the first contacts with newly arrived asylum seekers.	1. Investigation of the current health condition of asylum seekers<br>2. Assessment of health risks<br>3. Providing information about the health care system<br>4.Health education	> In education and training this rough framework thus can be used as a means to reflect upon priorities in health care to asylum seekers as well as being aware of possible pitfalls, dilemmas and difficulties.<br>> Potential aspects of training: the need for good communication skills (including the skill to work with a professional interpreter) to deal with cultural differences and to deal with possible high expectations of asylum seekers.<br>>Training may help care providers reflect upon their own boundaries of their medical profession: for example, should they be the ones to assess mental health problems of asylum seekers or is it better to refer to another institution with more relevant competencies?<br>>Sufficient time is needed for a consultation when all four elements are included.

For peer review only - http://bmjopen.bmj.com/site/about/guidelines.xhtml

For peer review only

Study Reference	Country of study	Study type	Total Participants in Study	Health professional practice setting	Service users	Data collection method	Analysis method	Study aims/purpose (Author's words)	Reported results (Themes/main headings)	Recommendations/applications (Author's words)
										>Reference to other types of care, such as mental health care, need to be ascertained, before care providers assess asylum seekers' needs such as mental health needs.<br>>Different issues may be addressed by different professionals (for example, assessing mental health problems may be done by a psychologist, health education may be done by a health educator).
Suurmond J, Seeleman C, Rupp I, Goosen S, Stronks K. Cultural competence among nurse practitioners working with asylum seekers. Nurse Educ Today 2010 11;30(8):821-826 6p.	Netherlands	Qualitative	89 nurse practitioners for survey element. 36 nurse practitioners in group interviews.	Asylum seeker centres	Asylum seekers	Questionnaires and group interviews	Framework	We explored the cultural competences that nurse practitioners working with asylum seekers thought were important.	1. Training and education in cultural competence<br>2. Knowledge of the political and humanitarian situation in the country of origin<br>3. Knowledge of epidemiology and the manifestation of diseases in asylum seekers' countries of origin<br>4. Knowledge of the effects of refugeehood on health<br>5. Awareness of the juridical context in which asylum seekers live<br>6. Skills to develop a trustful relationship with an asylum seeker<br>7. Ability to ask delicate questions about traumatic events and personal problems.<br>8. Ability to explain what can be expected from health care<br>9. Improving cultural competence	> These results add more specific competences to the cultural competences that have been described in other studies.<br>> It is not merely education or training that helps nurse practitioners feel culturally competent. Equally significant is the concrete experience of working with asylum seekers. This suggests that 'learning in action' by way of adequate supervision, mutual peer supervision, and systematic feedback on the work floor may also be a key teaching instrument. Thus, experiential and didactic learning may be integrated in order to develop relevant cultural competences.<br>> Cultural competences should not be seen as a list of skills that are acquired and ticked off one at a time, resulting in a person who is culturally competent. Acquiring cultural competence is an ongoing process, driven by the practitioners' self-reflection.
Tellep TL, Chim M, Murphy S, Cureton VY. Great suffering, great compassion: A transcultural opportunity for school nurses caring for Cambodian refugee children. Journal of Transcultural Nursing Oct 2001;12(4):261-274.	United States	Qualitative	6 school nurses 2 Cambodian liaisons*	Schools	Refugees	Focus group	Not specified	To describe the nature and meaning of school nurses' and Cambodian liaisons' experiences of caring for Cambodian refugee children and families and to explore whether those meanings validated Dobson's (1989) conceptual framework of transcultural health visiting.	1. Transcultural health-visiting education<br>2. Intracultural reciprocity<br>3. Transcultural reciprocity<br>4. Goal of maximising health and wellbeing: Letting go of one's own views<br>5. Multifaceted roles of Cambodian liaisons: We want to help them in any way<br>6. School and home: "Caught in the middle"<br>7. Intergenerational conflict: "It's hard for the kids"<br>8. The Cambodian	>Awareness of transcultural reciprocity and the importance of establishing trust may help guide other nurses in the development of meaningful relationships with Cambodian refugee children and families.<br>> Transcultural nursing care should be incorporated into all stages of the nursing process when caring for Cambodians.<br>> In partnership with the Cambodian community, interventions that target Cambodian refugee children with direct services, as well as indirect services through support of their families, are needed.<br>>Collaboration with others outside the school setting is vital to creating a cross-cultural team approach of coordinated and comprehensive service to Cambodian refugee children and families.<br>>Individualize care based on family's background and refugee history.<br>>Keep reaching out; trust takes time.<br>>Take a slow, friendly, no direct spiralling approach.

For peer review only

Study Reference	Country of study	Study type	Total Participants in Study	Health professional practice setting	Service users	Data collection method	Analysis method	Study aims/purpose (Author's words)	Reported results (Themes/main headings)	Recommendations/applications (Author's words)
									refugee experience: "Left for dead"<br>9. Spiritual healing: "It lifts your spirits"<br>10. Cultural strengths: Carried across the ocean"	>Gently probe.<br>>Suspend assumptions and worldview.<br>>Look beyond the behavior to understand the underlying dynamic.<br>>Support cultural traditions and share your interest<br>>Elicit explanatory models for illness.<br>>Incorporate spiritual healing practices and the temple into delivery of health services.<br>>Encourage and mentor Cambodian role models.<br>>Provide health education: family planning, nutrition, safety, and routine check-ups.<br>>Assist with access to care.<br>>Provide support to parents and elders.<br>>Assess refugee risk factors as part of special education process.<br>>Monitor medications.
Tobin C.L., Murphy-Lawless J. Irish midwives' experiences of providing maternity care to non-Irish women seeking asylum. International Journal of Women's Health 2014 31 Jan 2014;6(1):159-169.	Ireland	Qualitative	10 midwives	Maternity hospitals	Female asylum seekers	In-depth unstructure d interviews	Content analysis	To explore midwives' perceptions and experiences of providing care to women in the asylum process and to gain insight into how midwives can be equipped and supported to provide more effective care to this group in the future.	1. Barriers to communication<br>2. Understanding cultural difference<br>3. Challenges of caring for women who were unbooked<br>4. The emotional cost of caring,<br>5. Structural barriers to effective care.	>For women in the asylum process, having access to dedicated community-based services would begin to address the problems of access, late booking, and development of midwife/client relationships which in turn would help to decrease fear and anxiety for both the women themselves and the midwives who care for them.<br>>Cultural competency training: When considering how best to educate midwives to provide culturally competent care, the most important focus should be on using a framework of cultural humility.<br>> There is an urgent need for increased clinical support for midwives who care for traumatized women.<br>>Access to continuing education is also essential, along with debriefing and clinical supervision in order to maintain providers' own health and well-being.<br>> Trained interpreter service should be embedded within hospitals.<br>>dedicated community-based services that provide the possibility of continuity of care, make access to care easier for women, and provide the possibility of good midwife/client relationships and trust building.<br>> Revision of the government policy of forced dispersal for women in the asylum process who are pregnant or in the early postpartum period is urgently needed.
Twohig PL, Burge F, MacLachlan R. Pod people. Response of family physicians and family practice nurses to Kosovar refugees in Greenwood, NS. Canadian Family Physician 2000 Nov;46:2220-2225.	Canada	Qualitative	6 family practice nurses<br>10 family physicians	Clinic in refugee processing centre	Refugees	Semi-structured interviews	Textual analysis	To explore roles of family physicians and family practice nurses who provided care to Kosovar refugees at Greenwood, NS.	1. Clinical encounter<br>2. Expectation and experience<br>3. Roles and team functioning<br>4. Response	> Future responses to emergency situations might benefit from clearer descriptions of individual roles within the team.
Yelland J, Riggs E, Wahidi S, Fouladi F, Casey S, Szwarc J, et al. How do Australian	Australia	Mixed Methods. Interviews conducted	30 Afghan parents*<br>10 midwives<br>5 medical	Mixed Methods. Interviews conducted with Afghan parents	Refugee background	Interviews and focus groups	Thematic analysis	(1) investigate Afghan women and men's experience of the way that health professionals	1. Language services in the context of care<br>2. Women and men's experience of being	>Our findings support calls for standardised procedures to improve identification of people of refugee background in clinical settings.<br>>Building an understanding of the refugee experience,

For peer review only

Study Reference	Country of study	Study type	Total Participants in Study	Health professional practice setting	Service users	Data collection method	Analysis method	Study aims/purpose (Author's words)	Reported results (Themes/main headings)	Recommendations/applications (Author's words)
maternity and early childhood health services identify and respond to the settlement experience and social context of refugee background families?. BMC Pregnancy & Childbirth 2014;14:348.		with Afghan parents contained a quantitative element. No reported quantitative element in interviews with health professionals.	practitioners* 19 Community based health professionals*	contained a quantitative element. No reported quantitative element in interviews with health professionals.				approach inquiry about social factors affecting families having a baby in a new country, and (2) investigate how health professionals identify and respond to the settlement experience and social context of families of refugee background.	asked about social health issues 3. Identifying and responding to social health issues: the experience of health professionals	what health care providers need to be mindful of in providing care to families of refugee background, and knowledge of services for referral, is likely to go some way in building workforce capacity to assess and respond to the social circumstances of refugees. >Interactive training opportunities incorporating knowledge of the refugee and asylum seeker experience and ways of working with these families is a strategy to enhance health professionals understanding and skills. >Any attempts to improve the responsiveness of health services to the needs of families of refugee background need to consider innovative ways to work within system constraints.
Yelland J, Riggs E, Szwarc J, Casey S, Duell-Piening P, Chesters D, et al. Compromised communication: a qualitative study exploring Afghan families and health professionals' experience of interpreting support in Australian maternity care. BMJ Qual Saf 2016 Apr;25(4):e1-2014-003837. Epub 2015 Jun 18	Australia	Qualitative	30 Afghan parents* 10 midwives 5 medical practitioners* 19 Community based health professionals*	Various maternity care services	Refugee background	Interviews and focus groups	Thematic analysis	(1) describe Afghan women's and men's experiences of language support during pregnancy check-ups, labour and birth; (2) explore health professionals' experiences of communicating with Afghan and other refugee clients with low English proficiency; and (3) consider implications for health services and health policy.	1. The use of accredited interpreters in maternity care 2. Family members interpreting during pregnancy, labour and birth	> Improving identification of language needs at point of entry into healthcare, developing innovative ways to engage interpreters as integral members of multidisciplinary healthcare teams and building health professionals' capacity to respond to language needs, especially when clients' have experienced trauma that is likely to impact on their capacity to engage with healthcare, are critical to reducing social inequalities in maternal and child health outcomes for refugee and other migrant populations. >Potential 'solutions' in the context of maternity care include community and language-specific group pregnancy care sessions combining antenatal check-ups with information and support provided by a multidisciplinary team of health professionals including an accredited interpreter.

* These participants are not within the study definition of primary health care professionals and therefore their data have not been included in the thematic synthesis.

For peer review only

Supplement 4: Quality assessment of studies included in the thematic synthesis

Study reference*	Aims & methods	Research design	Sampling†	Data collection	Reflexivity	Ethical issues	Data analysis†	Discussion of findings	Value
Begg H, Gill PS. Views of general practitioners towards refugees and asylum seekers: an interview study. Diversity Health Soc Care 2005 12;2(4):299-305 7p.	Research aims not clearly articulated. Importance and relevance considered. Qualitative methodology is appropriate to capture General practitioner's views.	Use of qualitative design not explicitly justified.	17 general practitioners. "Purposeful sampling was used to recruit GP's, with more or less than 10% of the area population from the black and minority ethnic communities" and areas cross checked with the Refugee council. Age and ethnicity were not controlled for. One hundred GPs were randomly selected from the target locations using computer generated numbers, and approached via post and a follow-up phone call. Of these, 20 GPs volunteered to participate but 17 were actually interviewed as three opted out at the last minute due to work priorities.	Semi-structured Interviews conducted at GP practices by the author. No justification given for methods or setting of data collection. A previously piloted and refined topic guide was utilised with topics listed No detail on how data was recorded. Data collection terminated upon saturation of emergent themes.	Researcher considered the potential influence of her age (medical student), sex (female) ethnicity (as from ethnic minority) in the openness of participants. In addition, recruitment bias was considered (people with stronger opinions more likely to respond). Also discussed the reasons for volunteers opting out not being related to study aims.	No detail on how the study was explained to participants. Written consent was obtained prior to the commencement of each interview, and confidentiality maintained throughout. Lacking details on how researchers handled issues raised for participants by the study. Ethical approval was obtained from North West Multisite Research Ethics Committee.	A thematic framework analysis was conducted. "Data collection and analysis proceeded simultaneously, incorporating emergent themes into subsequent interviews. Emergent themes were compared by HB and PG independently before agreement and refinement of the themes." Did not contain a description of how data presented was selected. Sufficient data were presented to support the findings. Contradictory data were taken into account Researcher highlights the use of multiple coding to reduce bias in the analysis along with respondent validation.	The findings were explicit and clearly discussed. As mentioned in Q1, the research question is not clearly defined. The findings are discussed in the context of the wider literature. Credibility enhanced by respondent validation and multiple analysts.	Briefly considered the value of the study and contribution to research (highlighted some important issues surrounding the delivery of care to refugees and asylum seekers) identified areas for further research (lack of time, support, education, training and, financial resources) Acknowledges the limitations in generalisability as conducted in one metropolitan area.
Bennett S, Scammell J. Midwives caring for asylum-seeking women: research findings. Pract Midwife 2014 Jan;17(1):9-12.	Aims clearly stated with explanation of how the findings would be used to inform policy, education and practice. Qualitative methodology is appropriate for exploration of midwives experiences of caring for asylum seekers.	Use of qualitative design not explicitly justified.	10 midwives. The study was targeted at qualified midwives who had practised for a minimum of one year and had some experience of working with asylum-seeking women. Midwives were recruited via an email sent by the Head of midwifery; 10 volunteered to participate. All those who volunteered were included in the sample. Not clear whether there was a process to check eligibility of volunteers. Non-participation was not discussed.	Semi-structured interviews. Lacking details about the setting and who conducted the interviews. No justification given for methods or setting of data collection. No explicit reporting of how the interviews were conducted and the areas of enquiry. Interviews were audio recorded and transcribed. Data saturation not discussed.	The researcher's role and potential bias in the formulation of questions or data collection was not discussed. An 'audit trail' was kept, capturing influences, events, actions and decisions taken during the conduct of the study.	All participants were provided with information about the study and gave written consent. States that all the participants were volunteers and free to withdraw at any time. Confidentiality was not discussed. Lacking details on how researchers handled issues raised for participants by the study. Ethical approval was gained from the trust and NHS National Research Ethics Service.	"A thematic analysis was used to capture emerging patterns of data. These were reviewed and grouped into two overarching themes and four interconnected sub-themes. Rigour was maintained through a systematic process of enquiry, sampling and analysis." No indication of involvement of multiple researchers in the analysis. Sufficient data were presented to support the findings. Contradictory data not discussed. No examination of researcher's role, potential bias and influence during the analysis and in presentation of the data. Only some of the themes from the analysis are reported in this paper.	The findings were explicit and clearly discussed in relation to the original research question and within the context of the wider research literature. No discussion of the credibility of the research and did not report whether multiple researchers were involved in coding transcripts or interpretation of findings.	Considered the value of the study and the contribution of the research. Did not make suggestions for future research. Considered the generalisability of the findings. Provided a number of recommendations for practice, education and policy.
Burchill J. Safeguarding vulnerable families: work with refugees and asylum seekers.	No clear statement of research aims. Importance and relevance of	Use of qualitative design not explicitly	14 health visitors. Purposive sampling was used in which participants were selected for their	In-depth interviews were conducted at multiple health centres across the borough (number not	Author acknowledges that there may have been bias related to	Research aims were explained at a professional meeting of health	A thematic framework method was utilised that involved a constant comparative approach in which codes and transcripts	The findings were explicit and clearly discussed in relation to the original research	Considered the value of the study in raising awareness of commissioners to

For peer review only - http://bmjopen.bmj.com/site/about/guidelines.xhtml

Study reference*	Aims & methods	Research design	Sampling†	Data collection	Reflexivity	Ethical issues	Data analysis†	Discussion of findings	Value
Community Practitioner 2011 Feb;84(2):23-26.	research adequately stated. Qualitative methodology is appropriate for understanding health visitor's experiences of working with refugees and asylum seekers.	justified.	ability to contribute to the data. Recruitment was conducted by approaching potential participants through a presentation at a professional meeting. Sample was approximately 1/3 of all health visitors in the borough. Participants required to have worked for 2yrs as would be highly likely to have worked with refugees and asylum seekers. No discussion about the reasons why some health visitors chose not to participate.	specified), but unclear who conducted the interviews. No justification given for methods or setting of data collection. A topic guide used that had been developed from a literature review. Participants were asked primarily to describe their experiences of working with refugees and asylum seekers and what problems/difficulties they faced. Method of recording interview not described but states that interviews were transcribed. Data saturation not discussed.	the fact that he worked in the same workplace (colleagues) as the participants. Participants may not have been as open or willing to tell the truth in interviews.	visitors. Lacking discussion about how consent was gained, confidentiality maintained and how issues raised by the study were handled by researchers. Approval to proceed with the study was granted by the Primary Care Trust research and development team and the Local Research Ethics Committee.	were constantly reassessed and re-interpreted. Themes identified were compared across the data and interpretations discussed with external researchers. No reported duplicate coding. Quotations were chosen to illustrate the particular issues described Sufficient data were presented to support the findings. Contradictory findings were not presented. No examination of researcher's role, potential bias and influence during the analysis and in presentation of the data.	question. Limited discussion of findings in the context of the wider literature. No explicit discussion of the credibility of the research, but methods report that interpretation was discussed with external researchers.	provide appropriate services for refugees and asylum seekers. No further research areas suggested. No explicit discussion of transferability to other populations but suggests the findings will be useful for commissioners in other settings and that the study adds to literature that can inform policy and practice.
Burchill J, Pevalin D. Barriers to effective practice for health visitors working with asylum seekers and refugees. Community Practitioner 2012 Jul;85(7):20-23.	Research aims clearly stated. Importance and relevance were articulated. Qualitative methodology is appropriate for understanding barriers to effective practice for health visitors working with refugees and asylum seekers.	Use of qualitative design not explicitly justified.	14 health visitors. Purposive sampling was used in which participants were selected for their ability to contribute to the data. Recruitment was conducted by approaching potential participants through a presentation at a professional meeting. Participants required to have worked for 2yrs as would be highly likely to have worked with refugees and asylum seekers. No discussion about the reasons why some health visitors chose not to participate.	In-depth interviews were conducted at multiple health centres across the borough in which the participants worked (Number of centres not specified). Unclear who conducted the interviews. No justification given for methods or setting of data collection. A topic guide used that had been developed from a literature review and consisted of a number of broad statements that would help guide the interview. The interviews were taped and transcribed verbatim. Data saturation not discussed.	No critical examination of the researcher's role, potential bias and influence in research question formulation or data collection.	Research aims were explained at a professional meeting of health visitors. Lacking discussion about how consent was gained, confidentiality maintained and how issues raised by the study were handled by researchers. The Primary Care Trust and the Local Research and Ethics Committee granted approval for this study.	A framework method was used that involved a constant comparative approach in which the codes were continually reassessed and interpreted. The themes that were identified were compared across the data and discussed with external researchers. Quotations were chosen to illustrate the particular issues described. Sufficient data were presented to support the findings. Contradictory data were not presented. No examination of researcher's role, potential bias and influence during the analysis and in presentation of the data.	The findings were explicit and clearly discussed in relation to the original research question and within the context of the wider research literature. No discussion of the credibility of the research.	Discusses the contribution of the study in increasing awareness in primary health care staff of health service entitlements of refugees and asylum seekers. Also raises awareness for commissioners of barriers to effective services when deciding how to invest in appropriate services.
Burchill J, Pevalin DJ. Demonstrating cultural competence within health-visiting practice: working with refugee and asylum-seeking families. Diversity Equality Health Care 2014 06;11(2):151-159 9p.	The aims of the research clearly stated The importance and relevance of the research were articulated Qualitative methodology is appropriate to explore health visitor's experiences of working with	Use of qualitative design not explicitly justified. Authors describe the purpose and key features of the Framework approach that they have	14 health visitors. A presentation was given at the health visitors' main professional meeting with details of the study and an invitation to participate. Participants had to be qualified health visitors and worked in the borough for over 2 years - ensuring that they had enough experience. Sample size was 14/42 health visitors	In-depth interviews were conducted at multiple health centres across the borough in which the participants worked (Number of centres not specified). Unclear who conducted the interviews. A topic guide used that had been developed from a literature review and consisted of 10 broad open-ended	No critical examination of the researcher's role, potential bias and influence in research question formulation or data collection.	Potential participants approached at a professional meeting of health visitors. All confirmed participants were sent an information letter and consent form to be signed before	Framework analysis. "Each interview was first transcribed and then analysed using Framework. This involved a constant comparative approach throughout. The themes that were identified were compared across the data, and interpretations were discussed between the interviewer (JB) and external researchers consisting of an academic supervisor and a doctoral	The findings were explicit and discussed in relation to the research question. Findings not discussed in the context of the wider literature. No explicit discussion of the credibility of the research, but methods report that interpretation was discussed with	The author discusses the contribution of the study to existing knowledge. Concludes that aspects of cultural competence are lacking, but are being addressed at the local level. Identifies the need for research into models of cultural competence in a variety of primary

For peer review only - http://bmjopen.bmj.com/site/about/guidelines.xhtml

Study reference*	Aims & methods	Research design	Sampling†	Data collection	Reflexivity	Ethical issues	Data analysis†	Discussion of findings	Value
	refugees and asylum seekers.	chosen for the analysis.	working in the borough.<br>No discussion about the reasons why some health visitors chose not to participate.	statements. The topic guide was given to participants prior to the interview.<br>No justification given for methods or setting of data collection.<br>A tape recorder was used to record the interview, which was transcribed for the analysis.<br>Data saturation not discussed.		participation in the study. Lacking discussion of how confidentiality was maintained. “Each participant was offered debriefing at the end of the interview session to discuss any issues that might have arisen, particularly if any difficult experiences were referred to.”<br>Ethical approval was granted by the local NHS Research Ethics Committee, and research governance permission was gained from the Primary Care Trust Research and Development Team.	student.”<br>Sufficient data are presented to support the findings.<br>Contradictory data were taken into account.<br>No examination of researchers own role, potential bias and influence during the analysis and in presentation of the data.	external researchers.	care settings.<br>Discusses the generalisability of the results and highlights the limitations of the model used for this research study for other health care settings.
Carolan M, Cassar L. Pregnancy care for African refugee women in Australia: attendance at antenatal appointments. EVID BASED MIDWIFERY 2007 2007;5(2):54-58 5p.	The aims of the research clearly stated.<br>The importance and relevance of the research were articulated.<br>A qualitative methodology is appropriate for understanding factors that facilitate or impede uptake of antenatal care among refugee communities.	Researchers justified their choice of study methods.<br>The use of observational methods before the semi structured interviews could help the researcher gain cultural understandin g and build trust with the participants.	10 African women, 2 midwives, 1 family reproductive rights education program worker, 1 interpreter.<br>African women:<br>Recruitment was facilitated by the midwife, who asked women attending the clinic if they were interested in the study. Those indicating an interest were approached by the researcher and the nature of the study, time requirements and study purpose were explained. Women who were still interested were invited to participate.<br>No discussion about the reasons why some people chose not to participate<br>Clinic staff:<br>No description of how the clinic staff were selected for interview.<br>No explanation as to why this clinic was an appropriate place to	Data were collected in two phases. Phase 1 was 40 hours of observation at the women's clinic by a researcher. Phase 2 employed semi-structured interviews with staff and refugee women. Setting of data collection was African Women's Clinic. Unclear who conducted the interviews.<br>No justification given for methods or setting of data collection.<br>Areas of enquiry in the interviews are described. Researchers modified the questions asked in the interviews with attending women when it became apparent they did not understand questions.<br>Field notes were used to record observation element. Specific method of data recording during interviews not	No critical examination of the researcher's role, potential bias and influence in research question formulation or data collection.	Potential participants were approached by the researcher, who explained the nature and purpose of the research and the time commitment.<br>Participant's names were changed in the reporting of the study, but not clear whether this was explained to the participants.<br>No discussion of informed consent or how researchers handled issues raised by the study for participants.<br>The project was approved by university and hospital ethics committees.	Exact method used for data analysis not specified. Brief description of analysis process. “data analysis then proceeded through the following stages: Organising the data; Immersion in the data; Generating categories and themes; Coding the data; Offering interpretations; Seeking alternative explanations. Notes of analytical understandings and decisions were made throughout the process. Trustworthiness of findings was enhanced by asking two academic colleagues to independently generate a theme list.”<br>No explanation of how the data presented were selected from original sample.<br>Sufficient data are presented to support the findings.<br>Contradictory data are taken into account in the findings.<br>No examination of researcher’s role, potential bias and influence during the analysis and in presentation of the data.	The findings were explicit, discussed with reference to the research question and set within the context of the wider literature. The authors state that the trustworthiness of the findings are enhanced by asking two academic colleagues to independently generate a theme list during the analysis.	Authors suggest that community midwifery clinics might offer a solution for providing acceptable and sensitive services to refugee African women. Findings considered in relation to relevant research-based literature.<br>No further research areas are suggested. Transferability not discussed, but implied that similar healthcare services could be effective in other settings with refugee women.

For peer review only - http://bmjopen.bmj.com/site/about/guidelines.xhtml

Study reference*	Aims & methods	Research design	Sampling†	Data collection	Reflexivity	Ethical issues	Data analysis†	Discussion of findings	Value
			sample.<br>Non-participation not discussed	stated but transcription is mentioned.<br>Data saturation not discussed.					
Crowley P. The mental health needs of adult asylum seekers in Newcastle upon Tyne. Journal of Public Mental Health 2005;4(1):17-23.	Aims of the study were clearly stated and its importance and relevance articulated.<br>The qualitative element of this study was an appropriate methodology to explore perceptions of the causes of mental ill health among asylum seekers and investigate issues in delivering services to this group.	The use of qualitative methodology was not explicitly justified, but the purpose of interviews and focus groups was explained.	10 general practitioners and unspecified numbers of other participants (asylum seekers, managers, mental health service providers, housing support, agency staff, voluntary sector service providers, Interpreters)<br>Exact numbers of participants not reported.<br>No details given about how participants were selected for focus groups or interviews.<br>No justification given for the choice of these participants	Interviews, telephone interviews and focus groups were used to collect qualitative data, but no details about the interviewer(s). Lacking details of the setting of data collection, but some participants were interviewed by telephone.<br>Researcher justifies the use of some of the focus groups and interviews, but not the setting of data collection.<br>No details about how the interviews were conducted.<br>No details about how data were recorded during the interviews/focus groups.<br>No discussion of data saturation.	The researcher's role and potential bias in the formulation of questions or data collection was not discussed.	No details given about how the research was explained to participants.<br>No discussion of informed consent, confidentiality, or how issues raised in the course of study were handled by researchers.<br>Approval from an ethics committee is not reported.	No description given of the analysis process or whether multiple researchers were involved in the analysis.<br>Not clear how findings were derived from the data<br>Insufficient data are presented to support the findings.<br>Contradictory data were not taken into account.<br>No examination of researcher's role, potential bias and influence during the analysis and in presentation of the data.	The findings are explicit and discussed in relation to the research question<br>The findings are discussed in the context of the wider literature.<br>The credibility of the findings are not discussed	The author discusses the contribution of the study to existing knowledge, practice and policy.<br>No identification of new areas for research.<br>No discussion of whether the findings can be transferred to other populations.
Drennan VM, Joseph J. Health visiting and refugee families: issues in professional practice. J Adv Nurs 2005 01/15;49(2):155-163 9p.	The aims of the research clearly stated.<br>The importance and relevance of the research were articulated.<br>A qualitative methodology is appropriate to understand the perceptions of health visitors working with refugees and asylum seekers.	Authors had formulated a hypothesis that health visitors framed their work with refugee and asylum seeking women using Maslow's hierarchy of need. The study was undertaken to explore this hypothesis.<br>No justification of the specific qualitative methods employed.	13 health visitors.<br>The participants were recruited by purposive sampling. Health visitors who identified themselves as having a significant number of refugees and asylum seekers on their caseloads and had worked in inner London for more than 5 years and were currently working with refugees and asylum seekers.<br>No discussion about whether some people chose not to participate and their reasons.	Data were collected through semi-structured interviews, conducted at the health visitor's places of work. Unclear who conducted the interviews<br>No justification given for methods or setting of data collection.<br>"Broad, open ended questions were used in the interview, inviting informants to be discursive and reflective in recounting their experiences.". Areas of enquiry in the interviews are described.<br>Interviews lasted 45min-1hr.<br>Interviews were tape-recorded and subsequently transcribed.<br>Data saturation was not discussed.	No critical examination of the researcher's role, potential bias and influence in research question formulation or data collection.	"Participants…given a full information sheet about the purpose, methods and use of the study". "Formal written consent was obtained and participants were assured that their data would be anonymized and deleted after transcription.<br>"Participants were sent draft copies of the report to demonstrate that anonymity had been preserved".<br>Lacking details on how researchers handled issues raised for participants by the study.<br>Ethical approval was obtained from	Framework method was used to analyse data. "The theoretical issues identified in the literature were used to devise the coding framework. The interviewer and second author independently coded the transcripts against the framework; using word processing and spreadsheet functions software. Additional codes were assigned as the data suggested new themes and issues. A small number of discrepancies in coding between the two analyses were resolved through subsequent discussion. The coded material was then analysed for: (a) Commonalities between informants, (b) conflicting perceptions between informants and (c) evidence to support or disprove the use of a hierarchy of needs in framing practice."<br>Sufficient data are presented to support the findings.<br>Contradictory data are taken into account.<br>No examination of researcher's	The findings were explicit and discussed with reference to the research question.<br>Minimal discussion of the findings in relation to the wider literature.<br>The credibility of the research is not explicitly discussed, but the two authors independently coded transcripts against the framework with discrepancies resolved through discussion. In addition, participants were sent draft copies of the report for comment.	Briefly considers the value of the study. The author acknowledges that the single geographical setting and small sample size limit the conclusions.<br>The contribution of the study to existing knowledge and understanding is discussed.<br>Identified one possible avenue for further research - whether prioritization of children's needs over mothers could be another issue related to Maslow's pyramid.<br>They suggest that although the study was UK based, the issues raised in the study will likely resonate for public health nurses working in other countries.

For peer review only - http://bmjopen.bmj.com/site/about/guidelines.xhtml

Study reference*	Aims & methods	Research design	Sampling†	Data collection	Reflexivity	Ethical issues	Data analysis†	Discussion of findings	Value
						the local Research Ethics Committee.	role, potential bias and influence during the analysis and in presentation of the data.		
Farley R, Askew D, Kay M. Caring for refugees in general practice: perspectives from the coalface. Australian Journal of Primary Health 2014;20(1):85-91.	The aims of the research clearly stated. The importance and relevance of the research were articulated. Qualitative methodology is appropriate for exploring the experiences of primary health care providers working with refugees.	Use of qualitative design not explicitly justified.	20 general practitioners, 5 practice nurses, 11 administrators. Researchers explain how the participants were selected. 6 general practices were purposively selected on the basis that they had received newly arrived refugees in the past 6 months. Purposive sampling ensured that participating practices had experience of caring for refugees. Practices were approached by a researcher to discuss involvement in the project, which was followed up by a phone call to clarify involvement. No discussion of the proportion of practice staff that agreed to participate in the research or any reasons for non-participation.	5 Focus groups and 4 semi-structured interviews were used. The exact setting for data collection is not clear, but occurred during staff lunch breaks. RF facilitated the focus groups and conducted the semi-structured interviews. Authors justified the use of some semi-structured interviews as a way of overcoming time constraints for some participants and for testing whether focus groups were effective in surfacing the key themes. The setting was not justified. A standard introduction and interview schedule informed by the literature was used to stimulate conversation and discussion, but unclear whether this was for the focus groups, interviews or both. Brief description of the types of questions used. Authors report modification of methods in the study. Semi-structured interviews were used when time constraints prevented a focus group occurring and when a participant missed a focus group. Focus groups and interviews were audio recorded and transcribed. Data saturation is discussed.	It was acknowledged that personal relationships and power differentials in the workplace may have impacted on individual's freedom to express opinions in the focus groups. The authors were aware of this potential and took steps to minimise this. (offering opportunity to provide confidential feedback). Both researchers were working in refugee health and were aware of potential for influencing data collection and interpretation. To minimise this, a clear statement of the role of the researcher was explained to participants in the preamble to data collection.	Practices were provided with information sheets, confidentiality agreement and consent forms. Informed consent was obtained from each participant before involvement. Lacking details on how researchers handled issues raised for participants by the study. Ethical approval was granted by the Mater Health Services Human Research Ethics Committee.	"Key themes were identified using inductive thematic analysis and NVivo software was used to assist with data management. Analysis was iterative and data collection ceased when no new issues emerged, suggesting data saturation. RF and MK read each transcript and independently coded data, identifying a preliminary list of themes. RF produced a refined list of major themes and subthemes; MK endorsed these themes. Because similar themes were identified during the focus groups and interviews, the data were considered comparable and therefore analysed together." Sufficient data were presented to support the findings. Some Contradictory data were presented in the findings. Authors were aware of the potential bias in data analysis and stated that they critically reflected on how their own views and differing perspectives were influencing interpretation. One of the authors worked outside the field and was able to bring more objectivity.	The findings were explicit and clearly discussed in relation to the research question. Adequate discussion of the findings in relation to the wider literature. The researchers discuss the use of more than one analyst enhancing the credibility of the study. In addition, anonymised transcripts were provided to participants to give an opportunity for any further feedback.	The researcher provides an extensive list of recommendations for practice in relation to each of the main themes identified in the study. The research builds on the body of literature that focusses on the refugee perspective. Further areas for research are identified It is implied that this research will be able to help inform refugee healthcare on a national level although it is acknowledged that this research was carried out in one healthcare model.
Feldmann CT, Bensing JM, de Ruijter A. Worries are the mother of many diseases: General practitioners and refugees in the Netherlands on stress, being ill and prejudice.	The aims of the research clearly stated. The importance and relevance of the research were articulated. Qualitative	Authors state that "we set up an open ended, explorative study to learn about their frames of	66 refugees, 24 general practitioners. Refugee participants were approached through refugee initiated community organisations, Dutch Council for Refugees and personal networks (at least	Refugees: In-depth interviews were conducted by the first author (female former GP) with the help of female Somali or Afghan researchers. Setting for collection of data not	Researcher's role, potential bias and influence in research question formulation or data collection.	Lacking details about how the research was explained to participants. Interviews were conducted with consent from the	Refugees: "The first author analysed and coded the transcripts of the refugee interviews, using the WinMAX software program to organise the data and facilitate retrieval... After initial coding and cross-sectional comparison, a	The findings are explicit and discussed in relation to the research question. Authors discuss the findings in relation to the wider literature. No discussion of the	The contribution of the study to inform healthcare practice is discussed. A number of practice implications are given. Potential new areas of research are

For peer review only

Study reference*	Aims & methods	Research design	Sampling†	Data collection	Reflexivity	Ethical issues	Data analysis†	Discussion of findings	Value
Patient Educ Couns Mar 2007;65(3):369-380	methodology is appropriate for investigating the views of refugee patients and general practitioners about medically unexplained physical symptoms.	reference, expectations and experiences concerning health and healthcare."	partially purposive). Most GPs were a convenience sample from a letter sent to 325 GPs. 3 GPs were selected through personal contacts. Not clear what criteria were applied at the recruitment stage, but the refugees sample was shown to be diverse and representative. GPs had significant experience of caring for this group (21 had > 5 years' experience caring for Somali and Afghan refugees).<br>No discussion around non-participation.	described.<br>No justification given for methods or setting of data collection.<br>Topic list used that was developed in consultation with refugee experts and used in a flexible way. It was adapted during data collection, adding issues that seemed important.<br>Areas of enquiry are described.<br>Data recorded on tape and transcribed verbatim.<br>Data saturation not discussed.<br>GPs: Semi-structured interviews with open ended questions were conducted by a medical student (22) and the first author (3). Setting is not fully described, but reported that 12 were conducted on the telephone and 12 face-to-face at a place of participants' preference. States that the GP participants were likely to give a more positive response towards refugees as they were willing to make time for the interview.<br>No justification given for methods or setting of data collection.<br>Not clear whether a topic list was used for these interviews or the areas of enquiry covered.<br>3 interviews were tape recorded and transcribed verbatim. 21 were recorded through note-taking with the interviewer conscientiously elaborating on them immediately afterwards.<br>Data saturation not discussed.		participants. No discussion about how confidentiality was maintained or how issues raised through the study were handled by researchers.<br>No reference to ethics committee reported.	schematic presentation in short quotes was made of each refugee interview"<br>GPs: "The GP interviews were analysed and coded in the same way. A short profile was written for each doctor, linking interview results to doctor and practice variables. In an initial analysis, rough codes were assigned for the doctors' perceptions of the refugee groups, the problems the refugees presented to them, the way they dealt with these problems, and the constraints they met."<br>A secondary analysis was performed on both refugee and GP data with further content analysis, which formed the body of the article.<br>Sufficient data are presented to support the findings.<br>Contradictory data were taken into account.<br>No examination of researcher's role, potential bias and influence during the analysis and in presentation of the data.	credibility of the findings.	suggested.<br>No discussion of transferability to other populations.
Furler J, Kokanovic R, Dowrick C, Newton D, Gunn J, May C. Managing depression among ethnic	The aims of the research were stated.<br>Research question does not define	Use of qualitative design not Justified, however, in	8 family physicians.<br>Participants were included as part of a larger study known as 'Re-order', but lacking details on how they	Semi-structured interviews conducted by one of the authors (RK) and a research assistant. Lacking details about the	No critical examination of the researcher's role, potential bias and influence in research	Insufficient details about how the research was explained to participants.	"Three authors read transcripts and analysed them independently to identify themes and categories. Results were compared and discrepancies	The findings were explicit and clearly discussed in relation to the research question. The evidence from the	Discusses the findings in relation to practice of physicians and their approach to working with depressed patients

For peer review only

Study reference*	Aims & methods	Research design	Sampling†	Data collection	Reflexivity	Ethical issues	Data analysis†	Discussion of findings	Value
communities: A qualitative study. Annals of Family Medicine May-Jun 2010;8(3):231-236.	participants as refugees, but throughout the study it is apparent that they are refugees. The importance and relevance of the research were articulated. Qualitative methodology is appropriate to understand experiences of family physicians that work with patients with depression.	the discussion section, Authors state that the findings would not be found through conventional studies of medical records, billing records or patient reports.	were recruited. Explained that the participants were chosen because they were known to work extensively with a range of refugee and migrant communities (Table 1 displays length of time they had worked with these communities). No discussion about reasons for non-participation.	exact location of data-collection No justification given for methods or setting of data collection. Brief explanation of the areas covered in the interviews, but the full interview schedule is provided in an on-line appendix. Interviews lasted 1-1.5 hours and were audio-recorded and transcribed. Data saturation not discussed.	question formulation or data collection.	Lacking discussion about how consent was gained, confidentiality maintained and how issues raised by the study were handled by researchers. Ethical approval for the study was granted by the University of Melbourne Human Research Ethics Committee.	discussed with the wider group, and concepts were further refined. Additional thematic categories were added as the analysis developed." Authors emphasise that transparency in analysis and reporting was achieved by providing extensive verbatim quotes and independent assessments of transcripts and themes. Sufficient data were presented to support the findings. Contradictory data were not presented in the findings No examination of researcher's role, potential bias and influence during the analysis and in presentation of the data.	wider literature is discussed in relation to the findings of the study. Authors acknowledge that the sample was small and that the physicians were working with specific cultural groups. They also mention that 3 authors were involved in the thematic analysis and themes were discussed with the wider group.	in ethnic communities. Suggest areas for future research Lacking discussion about the transferability of the findings of the study or other ways the research could be used.
Griffiths R, Emrys E, Lamb CF, Eagar S, Smith M. Operation Safe Haven: The needs of nurses caring for refugees. Int J Nurs Pract Jun 2003;9(3):183-190.	The aims of the research clearly stated. The importance and relevance of the research were articulated. Qualitative research is an appropriate methodology to ascertain the needs of nurses that worked with refugees arriving from conflict areas.	Use of qualitative design not explicitly justified.	13 nurses, 1 medical records clerk, 2 nurse managers. Researcher explains that all the nurses and midwives employed at the centre during its 14-month operation were invited to participate in focus group discussions (Convenience sampling). 14 positive responses were received, which included a medical records clerk. Unclear how the two nurse managers were chosen for semi-structured interviews. Unclear why some people did not participate in the study, but the authors hypothesise that it could have been due to the distance from residence to study location, nurses no longer working in the same workplace or unable/unwilling to participate.	Data was collected through 2 focus groups (13 nurses and 1 medical records clerk) and 2 semi-structured interviews (Nurse managers). No information is given about the settings of data collection or the researcher(s) that conducted interviews. No justification given for methods or setting of data collection. For focus groups, an interview schedule developed by the researchers was used to guide discussion. 5 areas of discussion were described that were triggered by interview questions. Semi-structured interviews lasted 60-9-0 min and followed another format developed by the researchers, but lacking detail on the areas of discussion. Data were audio-recorded and transcribed Data saturation not discussed.	No critical examination of the researcher's role, potential bias and influence in research question formulation or data collection.	Lacking details about how the research was explained to participants and how consent was gained. To protect confidentiality, all participants were assigned a pseudonym. Lacking details on how researchers handled issues raised for participants by the study. Ethics approval was obtained from the South Western Sydney Area Health Service Research Ethics Committee and the University of Western Sydney Ethics Review Committee.	"Thematic analysis of focus group and in-depth interview transcripts was undertaken by a multidisciplinary research team, who re-read them several times to become immersed in the data. The team, drawing upon informants' stories of their experiences, then generated broad themes common throughout the text. Themes and emerging subthemes identified by the research team were then coded from the transcripts using a qualitative data management program (QSR Nvivo, QSR International)." Sufficient data were presented to support the findings, however the authors did not include many quotations. Some contradictory data are presented in the findings No examination of researcher's role, potential bias and influence during the analysis and in presentation of the data.	The findings are explicit and discussed in relation to the original research question. The findings are discussed within the context of the wider evidence in the literature. No explicit discussion of the credibility of the results, but authors report that a team conducted the thematic analysis implying multiple researchers involved in generating themes from the data.	The contribution of the study to practice within similar settings is discussed. Several recommendations are given for health care providers to improve support for nurses caring for refugees. Authors discussed how the findings might be relevant in other contexts and further research areas are suggested.
Jensen NK, Norredam M, Priebe S, Krasnik A. How do general	The aims of the research clearly stated.	Use of qualitative design not	9 general practitioners. The participants were purposively selected based	Semi-structured interviews took place at the workplace of the	No critical examination of the researcher's role,	The research was explained to the participants in a	Qualitative content analysis was undertaken. "The interviews were read several times to	The findings are explicit and discussed in relation to the	The researchers briefly consider the findings in the context of national

For peer review only - http://bmjopen.bmj.com/site/about/guidelines.xhtml

Study reference*	Aims & methods	Research design	Sampling†	Data collection	Reflexivity	Ethical issues	Data analysis†	Discussion of findings	Value
practitioners experience providing care to refugees with mental health problems? A qualitative study from Denmark. BMC Family Practice 2013;14:17.	The importance and relevance of the research were articulated. Qualitative methodology is appropriate for exploring general practitioner's experiences of providing care for refugees with mental health problems.	explicitly justified.	on working in clinics with high proportions of immigrants and were expected to have a high experience of working with immigrant and refugee patients. The research was explained to participants in a letter which was followed up with a phone call with further details and to inquire about their interest in taking part in the study. No discussion of non-participation.	professionals and carried out by the first author. No justification given for methods or setting of data collection Methods for data collection are described. An interview guide was developed by a project coordinating group in London (study was part of a broader EU project) and translated into Danish for use in this study. The first part of the interview included questions around delivery of care to immigrants in general. The second part began with a vignette (scenario of a refugee patient consultation), with pre-prepared questions to begin discussion. Interviews were recorded on a Dictaphone and transcribed. Data saturation was not discussed.	potential bias and influence in research question formulation or data collection.	letter, with more details being given in a phone call. Informed consent was obtained orally from all participants and they were ensured anonymity. Lacking details on how researchers handled issues raised for participants by the study. Ethical permission for this study has been waived by the Ethical Committee of the Capital Region of Denmark as Danish legislation does not require ethical approval for this type of study.	obtain a sense of the whole. The text was then divided into meaning units, which were then condensed and assigned categories and themes in a process moving towards a higher level of abstraction. The creation of categories and themes took place as an iterative process with ongoing reflection and revision of categories and themes. The whole context of the interviews was considered concurrently throughout this process. The initial analysis was carried out by the first author, but presented to and discussed with co-authors and other researchers with a background in public health, medicine and anthropology as part of the analytic process." Sufficient data are presented to support the findings. Contradictory data are presented and discussed. No examination of researcher's role, potential bias and influence during the analysis and in presentation of the data.	original research question. Findings are discussed within the context of evidence in the wider literature. Credibility of findings not explicitly discussed, but the author mentions that the initial stages of the analysis were conducted by the lead author and as themes emerged, they were discussed with the wider group including members from different discipline backgrounds.	policy for health care management of refugees. Briefly suggests ways to improve practice. Suggest the development of conversational models for general practitioners with points to be aware of in consultations with refugees. There is some discussion of the transferability of the results. The authors acknowledge that the participants had high levels of knowledge about refugees and asylum seekers, which is not true of many general practitioners. In addition, the vignette used for the interview gave a theoretical, isolated situation, which they acknowledge may limit generalisability.
Johnson D.R., Ziersch A.M., Burgess T. I don't think general practice should be the front line: Experiences of general practitioners working with refugees in South Australia. Australia and New Zealand Health Policy 2008;5(pagination):Arte Number: 20. ate of Pubaton: 08 Aug 2008.	The aims of the research clearly stated. The importance and relevance of the research were articulated. Qualitative methodology is appropriate to explore the challenges for GPs working with refugees.	Researchers justify the use of qualitative methodology. A qualitative approach was taken in order to gain a deeper understandin g of the challenges faced by general practitioners in private practice when providing care to refugees.	12 general practitioners and 3 medical directors of divisions. Potential participants were identified through a database of GPs who could be identified as having accepted refugee referrals. One of the authors also used his personal knowledge from previous related work. Further GPs were identified after interviews with medical directors of divisions. An introductory letter/invitation was sent to 77 potential GP participants, providing 6 participants. the remaining six were recruited through follow up phone calls. Medical directors of divisions were contacted by email with 2 agreeing to participate with a further participant agreed after a follow up phone call. These	Data were collected through semi-structured interviews. Most were conducted individually, but 3 of the GPs were conducted together in a group setting. No description of the setting for data collection or who conducted interviews. Use of semi-structured interviews was justified. They were able to examine challenges already identified in the literature as well as allowing new themes to emerge. No justification of setting. Lacking detail on how the interviews were conducted, but does briefly outline the general focus of the questions for the GPs and the medical directors of divisions. The interviews were tape recorded and transcribed	No critical examination of the researcher's role, potential bias and influence in research question formulation or data collection.	Lacking details about how the research was explained to participants and how consent was gained. Confidentiality was protected by assigning participants random numbers in the coding process. Lacking details on how researchers handled issues raised for participants by the study The study was approved by the University of Adelaide Human Research Ethics Committee.	"A template analysis approach was adopted where a coding template was developed which included a priori themes in addition to new themes identified from initial reading and analysis of the transcripts. Final thematic templates for both the GP and Division transcripts were agreed upon by the Project Team and then all data was coded according to these themes, with DJ undertaking the bulk of the coding. Two transcripts were also independently coded by the other members of the Project Team. Following this, comparisons were made and a consensus reached on how the remaining data was to be coded." Sufficient data are presented to support the findings. Contradictory data not presented No examination of researcher's role, potential bias and influence	The findings are explicit and discussed in relation to the original research question. Findings are discussed within the context of evidence in the wider literature. Lacking discussion of the credibility of the findings 2 out of 15 transcripts were independently coded by multiple researchers.	Considered the findings of the study in relation to practice and policy. Suggested that to provide more generalisable results a quantitative study should be conducted, but does not give any information about the aims of such a study. The authors discuss the transferability of the study and state that the small numbers limit its generalisability.

For peer review only - http://bmjopen.bmj.com/site/about/guidelines.xhtml

Study reference*	Aims & methods	Research design	Sampling†	Data collection	Reflexivity	Ethical issues	Data analysis†	Discussion of findings	Value
			were contacted because their areas were thought to contain high levels of refugee settlement.<br>Discusses the low response rate and some of the potential bias around those who did participate (i.e. participants more likely to be dissatisfied with current system of provision), however the researchers believe that there were also limited numbers of GPs with experience working with refugees.	verbatim.<br>Researchers state that data saturation was reached.			during the analysis and in presentation of the data.		
Kokanovic R, May C, Dowrick C, Furler J, Newton D, Gunn J. Negotiations of distress between East Timorese and Vietnamese refugees and their family doctors in Melbourne. Sociol Health Illn May 2010;32(4):511-527.	The aims of the research clearly stated.<br>The importance and relevance of the research were articulated.<br>Qualitative methodology is an appropriate methodology to explore how migration experiences are manifested in the lives of the participants and how resulting distress is negotiated and contested in their interaction with family doctors.	Use of qualitative design not explicitly justified, however the choice of in-depth interviews was justified as it allowed enough time for respondents to talk about their lives in their own words and focus on issues that were important to them.	5 general practitioners, 24 refugees from Vietnam and East Timor.<br>The refugee participants were purposively selected to include patients who had experienced depression and had used health services for depression care. They were recruited if they had been diagnosed with depression or prescribed antidepressants in the past year.<br>Lacking details about how refugee participants were first contacted, but the initial approach involved use of interpreters to explain the study. Those agreeing to be contacted by the research team were telephoned by a bilingual researcher with more information and to arrange a time for the interview.<br>Unclear how the GPs were selected or recruited to the study.<br>Authors give characteristics of the participants that suggest that these were an appropriate sample (10-25 years' experience).<br>No discussion around non-participation.	In-depth interviews were conducted. Most interviews were conducted in the Community Health Centre, but some (number not reported) of the refugees were interviewed in their homes. Interviews conducted by experienced qualitative researchers (first author and research fellow)<br>Researchers justified use of in-depth interviews as it allowed enough time for respondents to talk about their lives in their own words and focus on issues that were important to them.<br>Setting not justified<br>Authors report the use of an interview guide for a section of the interviews, but unclear about the full methods used with the refugees and the GP's.<br>The differing areas of discussion with refugees and GP's were outlined.<br>Interpreters were utilised for the majority of the interviews with refugees.<br>Data were audio-recorded and research notes were kept by the interviewer and interpreter. These were translated and transcribed in duplicate	Researchers discuss the complexities of interviewing using translators and the impact on researcher-interviewee communication. It is uncertain whether interpreters may have summarised or modified questions, answers and meanings.	Research was explained to refugees in their own language (through interpreters) at the initial contact and then in more detail in a telephone call. Unclear how the research was explained to GPs.<br>Consent was gained from all participants using consent forms in English or translations into relevant languages. No discussion of confidentiality or how issues raised through the stud for participants were handled by researchers.<br>Ethical approval was given by the University of Melbourne Human Ethics Research Committee.	An inductive thematic approach was taken.<br>The themes from the preliminary coding were used to create a coding frame which was applied to the data across all transcripts. The transcripts were marked and annotated, and emerging themes were then discussed among authors.<br>Unclear who and how many people coded the transcripts.<br>Sufficient data are presented to support the findings.<br>Researchers refer to contradictory data within their dataset.<br>No examination of researcher's role, potential bias and influence during the analysis and in presentation of the data.	The findings of the study were explicit, discussed in relation to the research question and set in context of the wider literature.<br>No explicit discussion of the credibility of the findings, but it is reported that transcripts were translated in duplicate and multiple researchers were involved in developing emerging themes.	Limited discussion of the contribution of the findings to practice or policy. Authors do suggest a reinvestigation of the way of conducting research on depression in a cross-cultural context.<br>Researchers point out that the issues around negotiation of distress investigated in this paper are broadly relevant in (cross-cultural) clinical practice.

For peer review only

For peer review only - http://bmjopen.bmj.com/site/about/guidelines.xhtml

Study reference*	Aims & methods	Research design	Sampling†	Data collection	Reflexivity	Ethical issues	Data analysis†	Discussion of findings	Value
				(to maximise legitimacy). Data saturation was not discussed.					
Kurth E, Jaeger FN, Zemp E, Tschudin S, Bischoff A. Reproductive health care for asylum-seeking women - a challenge for health professionals. BMC Public Health 2010;10:659.	Aims of the study were clearly stated and its importance and relevance articulated.<br>A qualitative study is appropriate to explore the perceptions of health care professionals providing health care to asylum seeking women.	Use of qualitative design not explicitly justified.	80 asylum seekers, 3 physicians, 3 nurse/ midwife, 1 psychologist, 3 interpreters<br>Purposive sample.<br>The people who were invited to participate were those who had been most involved with the asylum-seeking patients insured in the Health Maintenance Organisation (HMO) model - a service specifically set up for asylum seekers in Basil University Hospital.<br>All the professionals invited agreed to participate.	Semi-structured interviews were conducted in a quiet hospital room of the participant's choice.<br>Information about interviewers is not reported.<br>No justification given for methods or setting of data collection.<br>An interview guide was used. Areas of enquiry are described. Additional questions were asked to physicians about roles specific to them.<br>The interviews were audio recorded and transcribed verbatim.<br>Data saturation was not discussed.	The researcher's role and potential bias in the formulation of questions or data collection was not discussed.	Participants were informed about the aims of the study and they signed a consent form.<br>Lacking details about how confidentiality was maintained or how researchers handled issues raised for participants by the study<br>The study was approved by the joint ethical committee of the Cantons of Basel Stadt and Basel Land (Ethikkommission beider Basel).	Analysis followed steps of grounded theory methodology. “We started the process by open coding, which means that we categorized text segments into broad categories or themes … We continued with axial coding which included examining relationships between categories and connecting them accordingly…Finally, selective coding included the organisation of the diverse categories into a framework to explain the phenomenon under study. This framework is depicted and explained in details in the result section. To strengthen the rigour of the analysis, we discussed the results with experts in women’s health, ethics and research.”<br>Sufficient data are presented to support the findings.<br>Contradictory data not taken into account<br>No critical examination of researcher's role and influence in the analysis	The findings of the study were explicit, discussed in relation to the research question and in context of the wider research literature<br>Cross validation of quantitative and qualitative elements was thought to add to credibility.	Considered the value of the study and the findings in relation to practice and policy. Suggests areas for further research.<br>Researchers discussed the limitations of the small sample size and the research being conducted in a hospital setting with highly developed services.<br>Authors suggest that challenges could be greater in other settings.
Lawrence J, Kearns R. Exploring the 'fit' between people and providers: refugee health needs and health care services in Mt Roskill, Auckland, New Zealand. Health & Social Care in the Community 2005 Sep;13(5):451-461.	Aims of the study were clearly stated and its importance and relevance articulated.<br>Qualitative methodology is appropriate for investigating barriers faced by refugees in accessing health services and challenges faced by providers to meet their needs.	Use of qualitative design not explicitly justified, however the authors explain why they chose in-depth interviews as a data collection method.	5 Community representatives, 9 Refugee group representatives, 5 Medical practitioners, 1 Manager, 1 Administrator.<br>Participants were purposively selected in consultation with staff at the clinic. Community representatives selected based on length of involvement in the Mt Roskill community.<br>Refugee representatives were representative of ethnic groups in the area and chosen based on involvement with the community.<br>All seven members of staff at the clinic were sampled.<br>No discussion around non-participation of community representatives.	In-depth interviews were conducted with participants with most taking place at the clinic and some in the workplace of representatives. All interviews were conducted by the first author.<br>Authors justify their use of in-depth interviews: "Our rationale for this approach is that experience is constituted in participants’ accounts as they talk about their surroundings and reactions to them in ways which others can accept and understand. In-depth interviews are a suitable way of gathering and accessing such talk".<br>Setting justified on	The researcher's role and potential bias in the formulation of questions or data collection was not discussed.	Not clear how the study was explained to participants.<br>Respondents gave permission in accordance with agreed ethics protocols, but no further details.<br>No discussion of how confidentiality was maintained or how issues raised in the study were handled by researchers.<br>No reference to ethics committee reported.	A thematic analysis process is described, but it isn't clear whether this applied to all participant groups.<br>"we used a research framework that was built on a critical realist theoretical base, which assumes that realities are socially, culturally and historically situated, but are, nevertheless, experienced as material, objective and stable by participants ...After a period of familiarisation with the transcribed narratives, key themes were identified with reference to topics discussed in the interviews. Indicative narratives identified through this exercise are used to illustrate themes in this paper." No indication of involvement of multiple researchers in the analysis.<br>Sufficient data are presented	The findings of the study were explicit, and discussed in relation to the research question.<br>Limited discussion of the findings in the context of the wider literature.<br>No discussion of the credibility of the findings.	Authors discuss the contribution the study makes to existing knowledge and understanding. The findings are discussed in relation to practice and policy.<br>It is acknowledged that the study focussed on one clinic in one city.<br>Suggestion of conducting further similar studies in other locations to increase generalisability.

For peer review only - http://bmjopen.bmj.com/site/about/guidelines.xhtml

Study reference*	Aims & methods	Research design	Sampling†	Data collection	Reflexivity	Ethical issues	Data analysis†	Discussion of findings	Value
				grounds of convenience<br>A list of topics covered by the interviews is included.<br>Data were audio taped and transcribed.<br>Data saturation is not discussed.			support the some of the findings, however the section reporting health practitioner's experiences did not provide supporting quotations for some of the key challenges presented.<br>Contradictory data are not presented<br>No examination of researcher's role, potential bias and influence during the analysis and in presentation of the data.		
Riggs E, Davis E, Gibbs L, Block K, Szwarc J, Casey S, et al. Accessing maternal and child health services in Melbourne, Australia: Reflections from refugee families and service providers. BMC Health Serv Res 2012 01;12(1):117-117 1p.	Aims of the study were clearly stated and its importance and relevance articulated.<br>Qualitative methodology is appropriate to explore perspectives of parents from refugee backgrounds and service providers.	Qualitative research design is justified and the use of focus groups was justified on the grounds that some of the participants favoured this approach.	87 refugee background mothers, 12 nurses, 1 community worker, 1 community liaison, 5 bilingual workers,<br>3 community representatives, 2 managers of bilingual workers<br>The refugees were recruited by convenience sampling at locations where they were known to attend - playgroups, kindergarten, peer education programme, English language organisation. They were invited to participate through a bilingual worker/health worker who was known to them.<br>Researchers took measures to recruit a more representative sample when it became apparent that initial focus groups were not representative.<br>Researchers sought to understand the depth of experiences of refugee background parents had when engaging with MCH services.<br>Healthcare service providers were recruited through purposive sampling. Lacking information about how they were recruited or why they were an appropriate sample.<br>No discussion of reasons of non-participation.	7 Focus groups were used to collect data from refugees and 4 focus groups were used to collect data from service providers. Interviews used with community representatives/managers of bilingual workers. All focus groups were conducted by ER with assistance from KB or ED The setting of data collection is not described.<br>Use of focus groups and interviews was justified, but setting not described or justified.<br>Focus group guides were used and the areas of questioning were described. Modifications were made to the questions to maximise relevance for different groups.<br>Unclear what methods were used for the interviews<br>Focus groups and interviews were digitally recorded and transcribed, including interpreter translations.<br>Data saturation was not discussed.	The researcher's role and potential bias in the formulation of questions or data collection was not discussed.	Lacking details about how the research was explained to participants. A plain language statement and consent form were provided. No discussion around confidentiality or how issues raised throughout the study were handled by the researchers.<br>Ethical approval was given by the University of Melbourne and the Department of Education and Early Child Development.	Thematic analysis was used. "ER listened to all voice recordings, read and coded all transcripts, and developed categories to organise the data. ED and LG also read a sub-sample of transcripts and coded them. The coding was found to be very similar with any differences discussed by the researchers to arrive at a consensus about final codes. The researchers also discussed patterns, consistencies and contradictions within the data to develop the main themes. ER then refined the themes in consideration of their alignment with the existing literature. All research investigators and the study advisory group came together to discuss the themes, further interpret and explain the results and the implications and applications of the findings."<br>Sufficient data are presented to support the findings, however not all subthemes are supported with direct quotations from participants.<br>contradictory data were taken into account in the analysis.<br>No examination of researcher's role, potential bias and influence during the analysis and in presentation of the data.	The findings of the study were explicit, discussed in relation to the research question and extensively discussed within the context of wider literature.<br>Researchers discussed the credibility of the findings. They discuss triangulation when combining data from the focus groups and interviews, which they suggest can lead to an enhanced description of the phenomenon being explored.<br>Although not explicitly discussed in terms of credibility, the involvement of multiple researchers in coding a sample of transcripts and development of themes enhances the credibility of the findings.	Considered the value of the study and the findings in relation to practice and policy.<br>Suggested areas of further research. In particular, to assess the 'refugee mentor' model described as a potential way to promote access to MCH services.<br>The authors discuss the generalisability of the findings. They comment that as the study was conducted in outer urban areas of Melbourne, the findings may not be applicable to other locations in Victoria (e.g. rural and regional areas).
Samarasinghe K, Fridlund B, Arvidsson B.	Aims of the study were clearly stated	The authors discussed	34 PHCNs. Purposive sampling was used to	Interviews were conducted at the	Authors critically examine the	Research was explained to the	Contextual analysis with reference to phenomenography	The findings are explicit and discussed	The authors discuss the contribution the study

For peer review only - http://bmjopen.bmj.com/site/about/guidelines.xhtml

Study reference*	Aims & methods	Research design	Sampling†	Data collection	Reflexivity	Ethical issues	Data analysis†	Discussion of findings	Value
Primary health care nurses' promotion of involuntary migrant families' health. Int Nurs Rev 2010;57(2):224-231.	and its importance and relevance articulated.<br>Qualitative methodology is appropriate for exploring the experiences of primary health care nurses (PHCN) in health promotion with involuntary migrants.	their reasons for using a phenomenographic approach.	select participants for the study, which sought a wide spectrum of participants (sex, age, ethnicity, specialist education, length of primary health care nursing practice).<br>It is stated that each PHCN nurse had worked with approximately 200 involuntary migrant families, indicating that they would have the knowledge required for the studies aims.<br>No discussion about non-participation.	participant's workplace and were all conducted by the first author.<br>Methods not explicitly justified, however researchers explain that they piloted the interview questions beforehand to test the relevance of the questions (these pilots were included in the analysis). No justification given for choice of setting.<br>Areas of enquiry in the interview are described and the interviews lasted approximately 60 min each.<br>No modifications in the methods were necessary.<br>Interviews were tape recorded and transcribed verbatim.<br>Data saturation was not discussed.	influence of the researcher during the interview. "The first author, being a former PHCN herself, may have contributed to a common bond between the participants and the author, making the PHCNs able to freely express their thoughts throughout the interviews, which is crucial in a qualitative study"	participants through verbal and written information, including their right to withdraw from the study at any time.<br>Participants gave written consent and were assured of confidentiality (data being unidentifiable)<br>Lacking details on how researchers handled issues raised for participants by the study.<br>The study was approved by the university ethics committee of Sweden.	was used.<br>"The first author with experience of working within PHC carried out the analysis, while the two co-authors with specialized knowledge of the methodology served as additional evaluators in the categorization procedure.…The analysis was carried out in six steps: (1) the transcribed interviews were read several times to obtain a sense of the whole; (2) the interviews were processed, and descriptive statements relating to the aim of the study were identified, delimited, analysed and structured into an overview of concepts and keywords; (3) a comparative reduction of the data was commenced by giving a summarized description of each interview from this overview; (4) the summarized descriptions were differentiated by comparisons in relation to similarities and differences of the summarized descriptions, and were grouped together in three qualitatively distinct groups; (5) the underlying structure of the grouped descriptions was identified and described by going back and forth between the grouped descriptions and the original interviews;<br>(6) and the transcribed interviews of the 34 participant PHCNs were finally allocated to the three qualitatively distinct groups of these descriptions."<br>Sufficient data are presented to support the findings.<br>No examination of researcher's role, potential bias and influence during the analysis and in presentation of the data.	in relation to the original research question.<br>Findings are discussed in relation to the wider literature.<br>Authors discuss the process of re-evaluating data in validating the descriptive categories, including the choice of quotations.<br>The analysis was conducted by one person (lead author), but was evaluated by two other co-authors.	makes to informing clinical practice and policy.<br>Recommendations are given to improve the training of nurses, to equip them to work with involuntary migrant families.<br>Further research is suggested to determine how to facilitate cultural transition for involuntary migrants.<br>Transferability of the findings is discussed.
Suurmond J, Rupp I, Seeleman C, Goosen S, Stronks K. The first contacts between healthcare providers and newly-arrived asylum seekers: A qualitative study about which issues need to be addressed. Public Health Jul	Aims of the study were clearly stated and its importance and relevance articulated.<br>Qualitative methodology is appropriate for investigating the issues that healthcare providers	Use of qualitative design not explicitly justified.	36 nurse practitioners and 10 public health physicians. Participants were a purposive sample of nurse practitioners and public health physicians from different asylum seeker centres (from across the Netherlands). They were approached by the coordinator to ascertain if	7 group interviews were used to collect data and were conducted by two specified researchers (IR and CS). The setting was not described.<br>No justification given for methods or setting of data collection.<br>A topic list was used, which had been	The researcher's role and potential bias in the formulation of questions or data collection was not discussed.	Written information about the study was given to all participants who were assured of confidentiality and anonymity (Anonymity was assured by the use of codes).	Data was analysed by Framework approach.<br>Interviews were analysed, starting with the familiarization stage. "Short notes were made to identify themes. This resulted in a thematic framework. The framework was systematically applied to the material, and all interviews were reread and annotated accordingly. Charts	The findings are explicit and discussed in relation to the original research question.<br>There is adequate discussion of the findings in context of the wider literature.<br>No discussion of the credibility of the	The authors discuss the findings in relation to practice and policy, providing perspectives and models that can inform service provision for this group.<br>Authors sought to provide a generic model (beyond first contact) for healthcare provision

For peer review only - http://bmjopen.bmj.com/site/about/guidelines.xhtml

Study reference*	Aims & methods	Research design	Sampling†	Data collection	Reflexivity	Ethical issues	Data analysis†	Discussion of findings	Value
2013;127(7):668-673.	need to address in their first contact with asylum seekers.		they were willing to participate in a group interview.<br>The role of these health professionals in caring for asylum seekers was described, giving justification for their selection.<br>No discussion around non-participation.	developed from the results a survey that had previously been sent to a sample of nurse practitioners and public health physicians. Areas of enquiry are described. All interviews were recorded on tape and transcribed.<br>Data saturation was not discussed.		Informed consent was tape-recorded a priori the interviews.<br>Lacking details on how researchers handled issues raised for participants by the study.<br>The employer of the nurse practitioners and public health physicians (Community Health Services for Asylum Seekers) approved the study. Medical-ethical approval of this study was not required, according to the Dutch Medical Research Involving Human Subjects Act as it only involved care providers and it was not an intervention study.	were devised with headings (and sometimes subheadings) for each key theme. Each chart contained entries for several respondents. Finally, these charts were used to describe patterns through an iterative, comparative process of searching, reviewing, and comparing the data."<br>Sufficient data are presented to support the findings." No indication of involvement of multiple researchers in the analysis.<br>Contradictory data were not presented.<br>No examination of researcher's role, potential bias and influence during the analysis and in presentation of the data.	findings.	for asylum seekers, to increase generalisability to other settings.
Suurmond J, Seeleman C, Rupp I, Goosen S, Stronks K. Cultural competence among nurse practitioners working with asylum seekers. Nurse Educ Today 2010 11;30(8):821-826 6p.	Aims of the study were clearly stated and its importance and relevance articulated.<br>Qualitative methodology is appropriate to explore the views of nurse practitioners about cultural competencies that are important for working with asylum seekers.	Qualitative methodology not explicitly justified.	89 nurse practitioners completed questionnaires. 36 nurse practitioners participated in group interviews. Not reported whether there was overlap in these two data sources.<br>Participants in the questionnaire were a convenience sample. Those who returned the questionnaire were included. It is not known how many questionnaires were distributed, so a response rate cannot be given.<br>Participants for the group interviews were a purposive sample, selected by local coordinators in order to increase representation from different asylum seeker centres and maximise variation in experiences.	89 questionnaires and 7 group interviews were used to collect data., which were conducted by 2 named researchers.<br>The setting for data collection is not clearly described<br>The combination of questionnaires and group interviews (triangulation) was put forward as a way of increasing credibility.<br>No justification of setting<br>No discussion of how the questionnaires were developed. A topic guide was used for group interviews; however, the questions were not focussed on this particular research question. Data about cultural competence emerged in the course of the discussions.	The researcher's role and potential bias in the formulation of questions or data collection was not discussed.	Information about the study was given in the form of a flyer as well as in a letter accompanying the questionnaire.<br>Lacking details about how the research was explained to participants in group interviews.<br>Consent was gained from all participants and they were assured of confidentiality.<br>Lacking details on how researchers handled issues raised for participants by the study.<br>According to the Medical Research	Framework approach was used to analyse the data. "After familiarisation with the data, a coding framework was identified. The questionnaires were then systematically coded using this framework. Data were subsequently charted and three major charts were constructed: educational background, important cultural competences in connection with asylum seekers, and ideas about how cultural competences may be improved. The transcription of each group interview was read carefully to gain an overall impression before being coded and analysed. One chart was designed on the basis of different cultural competences that were mentioned in the interviews. Using this chart, patterns and connections could be described." Not clear how the two sources of data were	The findings are explicit and discussed in relation to the original research question.<br>There is adequate discussion of the findings in context of the wider literature. Authors suggest that credibility is enhanced by having two data sources (questionnaires and group interviews), which allows triangulation.	The contribution to existing knowledge and understanding is discussed. The authors state that the results of the study can be used for training and education of health care professionals. They believe that the results are relevant to other care providers who work with asylum seekers (generalisable) Further areas of research are not discussed.

For peer review only

For peer review only - http://bmjopen.bmj.com/site/about/guidelines.xhtml

Study reference*	Aims & methods	Research design	Sampling†	Data collection	Reflexivity	Ethical issues	Data analysis†	Discussion of findings	Value
			The role of these health professionals in caring for asylum seekers was described, giving justification for their selection. No details given about reasons for non-participation.	Group interviews were recorded on tape and transcribed. Data saturation was not discussed.		Involving Human Subjects Act, medical–ethical approval of this study was not required in the Netherlands (only care providers involved and not an intervention study). approval was obtained from the Community Health Services for Asylum Seekers, the employer of the nurse practitioners.	synthesised. No indication of involvement of multiple researchers in the analysis. Sufficient data are presented to support the findings, however only 2 direct quotations are used in the entire findings section, which had 9 headings. Contradictory data were not presented. No examination of researcher's role, potential bias and influence during the analysis and in presentation of the data.		
Tellep TL, Chim M, Murphy S, Cureton VY. Great suffering, great compassion: A transcultural opportunity for school nurses caring for Cambodian refugee children. Journal of Transcultural Nursing Oct 2001;12(4):261-274.	Aims of the study were clearly stated and its importance and relevance articulated. Qualitative methodology is appropriate to explore experiences of school nurses and Cambodian liaisons that provide care for refugee families.	Qualitative methodology not explicitly justified, although it is mentioned that the focus groups were conducted to gain insight into the concepts of transcultural and intracultural reciprocity as experienced by school nurses in their relationships with Cambodian refugees.	6 school nurses, 2 Cambodian liaisons. "A purposive sample of school nurses and Cambodian liaisons was recruited from a school district serving a large population of Cambodian children in California. Six of the district's eight school nurses volunteered as well as two of the three Cambodian liaisons." Invitation was through a phone call or letter. No reasons given for why these participants were chosen, although it is clear that the nurses had a high level of experience working with Cambodian refugees (6-15 years' experience). No discussion of the reasons for non-participation of those approached, that did not volunteer.	Focus group with Cambodian liaisons was held in the home of a non-Cambodian school nurse. Focus group with school nurses was held in their school district conference room. Focus groups were moderated by two of the authors. No justification for the methods Setting of the groups with Cambodians was justified based on wanting to provide a friendly atmosphere and authors explain the cultural reasons for tea/coffee and relational time before the interviews. No justification of the setting of nurse interviews. A semi-structured interview guide was used in the focus group. Broad areas of enquiry are described, but specific questions not stated. Data were tape recorded and transcribed verbatim. field notes were reviewed. Data saturation was not discussed.	The researchers critically examined their roles and potential bias in the data collection. "Through directing the research to look for insights into the concepts of transcultural and intracultural reciprocity, the authors may not have been as open to other concepts arising from the data regarding the nature of the participants' interactions with Cambodian refugee families. In retrospect, serving Cambodian refreshments at the school nurse focus group relayed the school nurse moderator's bias of transcultural interest and empathy toward Cambodians. This bias may have limited the types of information and viewpoints shared by the nurses. In addition, they may have been hesitant to share issues in the presence of the	Lacking details about how the research was explained to participants. Consent was gained before conducting the focus groups and issues of confidentiality were considered. Lacking details on how researchers handled issues raised for participants by the study. "San Jose State University's Human Subjects Institutional Review Board approved the study's research protocol."	Limited details about the analysis methodology or process. "Tapes were listened to and transcripts were reviewed several times by the moderators individually and together. The data were grouped and categorized into emergent issues and themes and also reviewed in light of Dobson's (1989) conceptual framework of transcultural health visiting." No indication of involvement of multiple researchers in the analysis. Sufficient data were presented to support the findings. Contradictory data were not presented. No examination of researcher's role, potential bias and influence during the analysis and in presentation of the data.	The findings are explicit and discussed in relation to the original research question. Little discussion of the findings in the context of the wider literature. As already noted in the reflexivity section, authors acknowledge potential bias in the interview process, which could impact the credibility of the findings.	Authors provide a number of recommendations for nursing practice Areas for further research are suggested. Authors discuss generalisation of findings, pointing out that focus group research results are not meant to be generalised. They refer readers to Kruger's concept of transferability when reflecting on using these findings in other settings.

For peer review only - http://bmjopen.bmj.com/site/about/guidelines.xhtml

Study reference*	Aims & methods	Research design	Sampling†	Data collection	Reflexivity	Ethical issues	Data analysis†	Discussion of findings	Value
					Cambodian nurse assistant moderator. A similar inhibitor may have existed in the presence of the non-Cambodian school nurse assistant moderator with the Cambodian liaison focus group.				
Tobin C.L., Murphy-Lawless J. Irish midwives' experiences of providing maternity care to non-Irish women seeking asylum. International Journal of Women's Health 2014 31 Jan 2014;6(1):159-169.	Aims of the study were clearly stated and its importance and relevance articulated. Qualitative methods are appropriate to explore midwives' perceptions of caring for women in the asylum process and gain insights into how they can be equipped to provide effective care to this group.	Use of qualitative methodology not explicitly justified.	10 midwives. The participants were purposively selected to ensure that they had experience providing care to asylum seekers. They were chosen from two different sites (an urban hospital and a rural hospital) to gain a wider variety of experiences. Information packs describing the study were distributed by researchers to the two sites; the researchers then followed up with visits to the sites to hold information sessions about the study and to answer questions. Demographic information about participants is included, demonstrating the appropriateness of the sample. No discussion around non-participation.	Data were collected by in-depth, unstructured interviews at a place convenient for participants (usually at home of at a private office space). Interviewer not reported. No justification given for methods or setting of data collection Interviews were launched with one open-ended question and ranged from 26-70 minutes. Data were audio recorded and later transcribed verbatim. Data saturation was not discussed.	Extensive field notes and reflective journals were kept, that provide an audit trail of decision-making and an aid for the qualitative researcher to deepen awareness of their own bias, reactions, and emotions to the data as they emerge. Clinical and peer supervision was used throughout the data collection process.	Information packs describing the study were distributed by researchers to the two sites; the researchers then followed up with visits to the sites to hold information sessions about the study and to answer questions. Informed consent, voluntary participation, and assurance of confidentiality were made explicit. Lacking details on how researchers handled issues raised for participants by the study. Article states that ethical approval was gained from relevant institutions, but details not provided.	Data were analysed using content analysis. "The analysis was undertaken by hand, and involved several readings of transcripts, followed by coding of data and grouping coded material based on shared content or concepts to identify common themes. Transcripts were also read in their entirety by a second researcher to confirm the themes that were identified and add to the validity of the findings." Sufficient data is presented to support the findings. Contradictory data are considered No examination of researcher's role, potential bias and influence during the analysis and in presentation of the data.	The findings are explicit and discussed in relation to the original research question. There is adequate discussion of the findings in context of the wider literature. No explicit discussion of the credibility of the findings.	The contribution of the study to existing knowledge and understanding was discussed. highlights the continued difficulties midwives experience in achieving effective communication, understanding difference, and coping with the emotional cost of caring within a hospital-based technological model of maternity care. Recommends ways that service delivery to asylum seekers could be improved. New areas for research are identified. Authors acknowledge that the study is small scale, and cannot be generalised to the whole population.
Twohig PL, Burge F, MacLachlan R. Pod people. Response of family physicians and family practice nurses to Kosovar refugees in Greenwood, NS. Canadian Family Physician 2000 Nov;46:2220-2225.	Aims of the study were clearly stated and its importance and relevance articulated. Qualitative methodology is appropriate for exploring the experiences of family practice nurses and family physicians that cared for refugees in a refugee	Use of qualitative methodology not explicitly justified.	6 family practice nurses, 10 family physicians. Participants were purposively sampled from the service roster to enrol different kinds of family practice nurses and family physicians. Lacking details about how participants were invited to participate. All the participants had worked at the centre that was the focus of the study. No discussion about non-	Data were collected through semi-structured interviews at a 'private setting' (no further details) and were conducted by one team member (PT). No justification given for methods, but does justify setting as a private place to allow the participants to freely and openly share experiences. Lacking explicit details about the methods (no	The researcher's role and potential bias in the formulation of questions or data collection was not discussed.	Lacking details about how the research was explained to participants. Written consents were obtained, but lacked details about confidentiality and how issues raise for participants in the study were handled by researchers.	A form of textual analysis was applied. "For each interview, key words or phrases were identified and compared with subsequent inter views until no significant new ideas emerged. Once researchers were satisfied that saturation had been achieved, words and phrases were grouped into larger conceptual categories. A second researcher reviewed a subset of transcripts and critiqued and confirmed the preliminary categories. This	The findings are explicit and discussed in relation to the original research question. Lacking discussion of the findings in the context of the wider literature. No explicit discussion of the credibility of the findings although researchers report that a second researcher critiqued categories	The contribution made by the study to existing knowledge and understanding is discussed. Teamwork in emergency response is suggested as a possible avenue for further research. Authors acknowledge that the findings of the study cannot be generalised to other relief settings, but

For peer review only - http://bmjopen.bmj.com/site/about/guidelines.xhtml

Study reference*	Aims & methods	Research design	Sampling†	Data collection	Reflexivity	Ethical issues	Data analysis†	Discussion of findings	Value
	processing centre.		participation.	description of interview guide, format, areas of enquiry)<br>Data were audiotaped and transcribed verbatim.<br>Data saturation was discussed in the analysis process. Comparisons of key words and phrases were made across interviews until no new themes emerged.		Ethics approval was obtained from Dalhousie's Faculty of Medicine.	process was repeated until the categories were clear. These categories became the basis for a coding structure within QSR NUD*IST, software designed for textual analysis."<br>Sufficient data were presented to support the findings.<br>Contradictory data were taken into account.<br>No examination of researcher's role, potential bias and influence during the analysis and in presentation of the data.	that emerged in the analysis	suggest that they could offer insights to generate other research questions.
Yelland J, Riggs E, Wahidi S, Fouladi F, Casey S, Szwarc J, et al. How do Australian maternity and early childhood health services identify and respond to the settlement experience and social context of refugee background families?. BMC Pregnancy & Childbirth 2014;14:348.	The aims of the study were clearly stated and its importance and relevance articulated.<br>Qualitative methodology is appropriate to explore experiences of Afghan parents accessing maternity services and health professional's views on/experiences of identification of refugee background.	Authors state that the methods were informed by community and service provider consultations.	30 Afghan parents, 10 midwives, 5 medical practitioners, 19 Community based health professionals.<br>Afghan men/women: "Purposive recruitment methods and multiple initial contacts were used to optimise recruitment and ensure diversity of potential participants." Inclusion criteria was women and men born in Afghanistan ≥ 18 years old and had a baby that was around 4-12 months old.<br>Also an element of convenience sample. "A postcard with information about the study and details about how to take part, in Dari and English, was distributed to local groups and services, and the postcard was printed in the Afghan community newspaper. Potential participants were provided with a telephone number to contact the community researchers to register their interest in participating in an interview."<br>No discussion around non-participation.<br>Health professionals: Purposive sample. Key informants invited to participate after identification by researchers, with further participants identified through initial participants. All provided care for	Afghan parents: Semi-structured interviews were conducted by community researchers. Setting of data collection was described. Participants were given a choice of location and language preference for interview<br>No justification given for methods or setting of data collection.<br>Interview schedule was designed based in information from a previous community consultation. Areas on enquiry are described.<br>Authors report that the interview schedule was modified after piloting with 6 participants.<br>Interviews were recorded on audio tape. Those conducted in Afghan language (80%) were translated into English and transcribed by community researchers.<br>Data saturation not discussed.<br>Health professionals: A mixture of focus groups and interviews were used with the majority being conducted by one author (ER); one was conducted by another author (JY). The setting of interviews/focus groups not reported.<br>No justification given for methods or setting of	The researcher's role and potential bias in the formulation of questions or data collection was not discussed.	Afghan parents: Potential participants were provided with verbal information and given a copy of the study information in Dari or English and were asked to consent in writing or verbally.<br>Confidentiality, or how issues raised in the study for participants were handled by researchers, are not discussed.<br>Health professionals: Lacking detail about how the research was explained to these participants.<br>No discussion around consent, confidentiality or how issues raised through the study were handled by researchers.<br>"The project was approved by the research ethics committees of the Victorian Foundation for Survivors of Torture and the Royal Children's Hospital."	A thematic analysis approach was taken and the analysis process is described for analysing qualitative data from Afghan parents and health professionals.<br>Afghans: "Analysis began after the first three interviews with women which were coded, informing the coding manual. A coding manual was developed using some a priori codes from the interview schedule; an iterative process was used to add additional codes to the manual (undertaken by ER, JY, FF,SW). This coding manual was used to code all women and men's interviews. JY and ER cross-checked the coding of all interview transcripts, providing an opportunity to discuss differences in the interpretation of the data. Codes were then grouped into logical categories which then provided the overarching themes."<br>Health professionals: "All transcripts were read (by ER, JY) and imported and stored in NVivo10 [26]. Coding and categorising of data was undertaken (by ER), and key themes identified."<br>Authors state that the paper does not report all the themes.<br>Quotations were selected to illustrate the themes identified in the analysis.<br>Sufficient data were presented to support the findings.<br>Contradictory data were taken into account.<br>No examination of researcher's role, potential bias and influence	The findings are explicit and discussed in relation to the original research question.<br>Authors discuss the findings in relation to the wider research literature.<br>Authors discuss the strength of having two components of the study - afghan community and health professionals.<br>Thematic analysis of afghan participant data involved multiple analysts. Analysis of health professional data was primarily completed by one author.	Authors discuss the contribution of the study to existing knowledge and understanding.<br>Further areas for research are not discussed.<br>The transferability of the results is discussed. The authors acknowledge that this study included one community group in one region of Melbourne, so may not be generalisable to other groups. However, authors suggest that the stories told here may resonate with other groups in other settings.

For peer review only - http://bmjopen.bmj.com/site/about/guidelines.xhtml

Study reference*	Aims & methods	Research design	Sampling†	Data collection	Reflexivity	Ethical issues	Data analysis†	Discussion of findings	Value
			families of refugee background.<br>No discussion around non-participation.	data collection.<br>An interview schedule was used with areas of enquiry described.<br>Interviews were digitally recorded and transcribed by an outside agency.<br>Data saturation was not discussed.			during the analysis and in presentation of the data.		
Yelland J, Riggs E, Szwarc J, Casey S, Duell-Piening P, Chesters D, et al. Compromised communication: a qualitative study exploring Afghan families and health professionals' experience of interpreting support in Australian maternity care. BMJ Qual Saf 2016 Apr;25(4):e1-2014-003837. Epub 2015 Jun 18	Aims of the study were clearly stated and its importance and relevance articulated.<br>Qualitative methodology is appropriate to explore experiences of Afghan parents and health professionals.	Research methodology not explicitly justified, but authors state that the data collection methods were informed by consultation with Afghan community members and health professionals working in the area.	Afghan women and men:<br>Potential participants were identified through consultation with community groups, community leaders and the project's advisory group.<br>Not clear how individuals were approached.<br>Inclusion criteria was women and men born in Afghanistan ≥ 18 years old and had a baby that was around 4-12 months old.<br>No discussion around non-participation<br>Heath professionals:<br>Mixed purposive/convenience sample. The research was promoted within organisations that health professionals worked. Those interested in participating responding. Others personally recommended by key stakeholders.<br>Participants were eligible if they had provided services to Afghan families.<br>No discussion around non-participation.	Afghan participants:<br>Interviews were used to collect data and were conducted by Afghan background researchers (one woman, one man).<br>The setting of data collection was not described.<br>No justification given for methods or setting of data collection.<br>An interview schedule that had been developed with input from a previous population-based survey and was translated into Dari and piloted with 6 community members. Areas of enquiry are described.<br>Interviews were audio-taped and transcribed into English.<br>Data saturation was not discussed.<br>Health professionals:<br>Focus groups and interviews were used, but the setting or the interviewer(s) for data collection are not described.<br>An interview schedule was used and areas of enquiry are described.<br>No justification given for methods or setting of data collection.<br>Interviews were digitally recorded and transcribed by an external agency.<br>Data saturation was not discussed.	No explicit critical examination of the researcher's role and potential bias in formulating the research question or data collection. The Authors employed a participatory approach, which enhanced their capability to engage with the community (involved community members in recruitment and conducting interviews)	Afghan participants:<br>Lacking details on how the research was explained to potential participants.<br>Permission was given for audio-recording, but unclear whether consent was given for participation in the study.<br>No discussion of how confidentiality was maintained or how issues raised through the study were handled by researchers.<br>Health professionals:<br>Lacking details about how the research was explained to potential participants.<br>No details given about how participants consented, how confidentiality was maintained or how issues raised through the study were handled by researchers.<br>"The project was approved by the research ethics committees of the Victorian Foundation for the Survivors of Torture and The Royal Children's Hospital."	Afghan participants:<br>A thematic approach was taken. "All transcripts were coded manually by the community researchers and cross-checked (by FF, SW, ER, JY) and entered into NVivo10. Based on the completed coding of the first four transcripts (two women and two men) a coding manual was developed and used to code remaining transcripts. Discussion among the research team was done to place all codes into logical categories. From this seven, major themes were identified and the theme of 'language services and communication' is reported in this paper"<br>Health professionals:<br>Thematic approach was taken. "JY read all of the transcripts. The data were analysed thematically. All transcripts were coded using NVivo software (by ER) into practical categories and overarching themes."<br>Authors discuss how data were selected for this publication and that it does not represent all the themes, which were published elsewhere.<br>Sufficient data are presented to support the findings.<br>Contradictory data are taken into account<br>No examination of researcher's role, potential bias and influence during the analysis and in presentation of the data.	The findings are explicit and discussed in relation to the research question<br>The findings are discussed in the context of the wider literature.<br>Lacking explicit discussion of the credibility of the findings, however authors discuss the merits of using a participatory approach to engage refugees and it is apparent that more than one analyst was involved in defining themes from data from Afghan participants.	Authors discuss the contribution of the study to existing knowledge and understanding.<br>Further areas for research are not discussed.<br>The transferability of the results is discussed.<br>The authors acknowledge that this study included one community group in one region of Melbourne, so may not be generalisable to other groups. However, authors suggest that the stories told here may resonate with other groups in other settings.

For peer review only - http://bmjopen.bmj.com/site/about/guidelines.xhtml

Study reference*	Aims & methods	Research design	Sampling†	Data collection	Reflexivity	Ethical issues	Data analysis†	Discussion of findings	Value

* Direct quotations from articles in this table are presented within quotation marks.
† Sampling and analysis methods are as reported by the authors.

For peer review only

ENREQ Reporting Checklist

Robertshaw et al. Challenges and facilitators for health professionals providing primary healthcare to refugees and asylum seekers in high-income countries: A systematic review and thematic synthesis of qualitative research.

No	item	Guide & Description	Included	Page
1	Aim	State the research question the synthesis addresses.	✓	5
2	Synthesis methodology	Identify the synthesis methodology or theoretical framework which underpins the synthesis, and describe the rationale for choice of methodology (e.g. meta-ethnography, thematic synthesis, critical interpretive synthesis, grounded theory synthesis, realist synthesis, meta-aggregation, meta-study, framework synthesis).	✓	6
3	Approach to searching	Indicate whether the search was pre-planned (comprehensive search strategies to seek all available studies) or iterative (to seek all available concepts until they theoretical saturation is achieved).	✓	6
4	Inclusion criteria	Specify the inclusion/exclusion criteria (e.g. in terms of population, language, year limits, type of publication, study type).	✓	7
5	Data sources	Describe the information sources used (e.g. electronic databases (MEDLINE, EMBASE, CINAHL, psycINFO, Econlit), grey literature databases (digital thesis, policy reports), relevant organisational websites, experts, information specialists, generic web searches (Google Scholar) hand searching, reference lists) and when the searches conducted; provide the rationale for using the data sources.	✓	6
6	Electronic Search strategy	Describe the literature search (e.g. provide electronic search strategies with population terms, clinical or health topic terms, experiential or social phenomena related terms, filters for qualitative research, and search limits).	✓	6
7	Study screening methods	Describe the process of study screening and sifting (e.g. title, abstract and full text review, number of independent reviewers who screened studies).	✓	6
8	Study characteristics	Present the characteristics of the included studies (e.g. year of publication, country, population, number of participants, data collection, methodology, analysis, research questions).	✓	11
9	Study selection results	Identify the number of studies screened and provide reasons for study exclusion (e,g, for comprehensive searching, provide numbers of studies screened and reasons for exclusion indicated in a figure/flowchart; for iterative searching describe reasons for study exclusion and inclusion based on modifications to the research question and/or contribution to theory development).	✓	9-10
10	Rationale for appraisal	Describe the rationale and approach used to appraise the included studies or selected findings (e.g. assessment of conduct (validity and robustness), assessment of reporting (transparency), assessment of content and utility of the findings).	✓	8
11	Appraisal items	State the tools, frameworks and criteria used to appraise the studies or selected findings (e.g. Existing tools: CASP, QARI, COREQ, Mays and Pope [25]; reviewer developed tools; describe the domains assessed: research team, study design, data analysis and interpretations, reporting).	✓	8
12	Appraisal process	Indicate whether the appraisal was conducted independently by more than one reviewer and if consensus was required.	✓	8
13	Appraisal results	Present results of the quality assessment and indicate which articles, if any, were weighted/excluded based on the assessment and give the rationale.	✓	17-18
14	Data extraction	Indicate which sections of the primary studies were analysed and how were the data extracted from the primary studies? (e.g. all text under the headings "results /conclusions" were extracted electronically and entered into a computer software).	✓	8
15	Software	State the computer software used, if any.	✓	8
16	Number of reviewers	Identify who was involved in coding and analysis.	✓	8
17	Coding	Describe the process for coding of data (e.g. line by line coding to search for concepts).	✓	8
18	Study comparison	Describe how were comparisons made within and across studies (e.g. subsequent studies were coded into pre-existing concepts, and new concepts were created when deemed necessary).	✓	8
19	Derivation of themes	Explain whether the process of deriving the themes or constructs was inductive or deductive.	✓	8
20	Quotations	Provide quotations from the primary studies to illustrate themes/constructs, and identify whether the quotations were participant quotations of the author's interpretation.	✓	22-23
21	Synthesis output	Present rich, compelling and useful results that go beyond a summary of the primary studies (e.g. new interpretation, models of evidence, conceptual models, analytical framework, development of a new theory or construct).	✓	19-31

This checklist was taken from Tong et al. BMC Medical Research Methodology 2012, 12:181

For peer review only
